# Risk factors for endoscopic postoperative recurrence in patients with Crohn’s Disease: a protocol for systematic review and meta-analysis

**DOI:** 10.1186/s12876-024-03301-z

**Published:** 2024-06-25

**Authors:** Dongchi Ma, Yu Li, Ling Li, Lili Yang

**Affiliations:** 1 School of nursing, Zhejiang Chinese Medical University, 548 Bin-wen Road, Hangzhou, Zhejiang 310053 PR China; 2 School of nursing, Zhejiang Shuren University, 8 Shuren Road, Hangzhou, Zhejiang 310015 PR China

**Keywords:** Crohn’s disease, Postoperative recurrence, Endoscopic recurrence, Risk factors, Meta-analysis, Systematic review

## Abstract

**Background:**

Crohn’s disease (CD) is a chronic condition characterized by a high recurrence rate after surgery, which seriously affects the quality of life of patients. Many studies have explored the risk factors for the recurrence of CD after surgery, there is a lack of meta-analysis focusing on endoscopic postoperative recurrence (ePOR) as a clinical outcome. Therefore, this paper aims to identify the risk factors for ePOR in CD patients through systematic review and meta-analysis.

**Methods:**

PubMed, Embase, Cochrane Library, and Web of Science databases were searched for related literature from inception to 17th October 2023. Two researchers independently screened the literature and extracted information. Data analysis was performed using Stata18.0.

**Results:**

Twenty-three papers were included, with 5 case-control studies and 18 cohort studies. The National Institutes of Health quality assessment tool rated 17 studies as good and 6 studies as fair. The sample size of the 23 studies ranged from 40 to 346, and the number of patients with ePOR ranged from 23 to 169. The results of multivariate meta-analysis showed that smoking [OR = 2.06, 95% CI (1.65, 2.57), *P* = 0.0001], previous ileocolonic resection [OR = 1.71, 95% CI (1.23, 2.38), *P* = 0.002], disease localization at ileocolic resection [OR = 2.68, 95% CI (1.38, 5.22), *P* = 0.004], perianal disease [OR = 1.47, 95% CI (1.07, 2.03), *P* = 0.017], and anastomotic scattered ulcer [OR = 3.39, 95% CI (1.83, 6.28), *P* = 0.001] were risk factors for ePOR in CD patients. Postoperative prophylactic medication [OR = 0.53, 95% CI (0.38,0.75), *P* = 0.0001] was a protective factor for ePOR in CD patients.

**Conclusions:**

This systematic review identified multiple factors for ePOR in CD patients, as well as a protective factor. However, the number of articles included was limited. More high-quality clinical studies are required to further validate the conclusions.

**Trial registration:**

This study was registered in the International Prospective Register of Systematic Reviews (PROSPERO) (CRD42023483671).

**Supplementary Information:**

The online version contains supplementary material available at 10.1186/s12876-024-03301-z.

## Background

Crohn’s disease (CD) is a persistent, non-specific inflammatory condition of the intestinal tract, with the terminal ileum and colon being the most commonly affected areas [1]. This disease is often associated with complications such as intestinal obstruction and intestinal perforation, significantly impacting the individual’s quality of life [2]. Current guidelines suggest that surgical intervention should be considered for CD patients who have not shown improvement with medical therapy, particularly in cases involving fibrous stenosis of the bowel, bowel perforation, and abscess formation in the abdominal cavity. Enterectomy is the most frequently performed surgical procedure in such cases [3]. However, surgery is not a cure for CD. Over 70% of CD patients require surgical intervention during their lifetime and experience endoscopic postoperative recurrence (ePOR) and clinical recurrence, with approximately 50% of patients ultimately requiring re-surgery after 10 years [4, 5]. Repeated surgeries can result in bowel failure or short-bowel syndrome, significantly affecting the quality of life of patients [5].

Currently, the main types of postoperative recurrence in CD patients are ePOR and clinical recurrence [6]. Research indicates that a majority of patients exhibit asymptomatic mucosal lesions during endoscopic examination before clinically symptomatic recurrence, i.e., endoscopic recurrence [7]. Prior research has identified a strong correlation between ePOR and clinical recurrence, suggesting its potential as a primary endpoint in postoperative clinical trials for CD patients [8]. Moreover, ePOR (empirically defined as a Rutgeerts score of ≥ i2 on ileocolonoscopy) is widely regarded as the gold index for assessing the clinical course and severity of postoperative recurrent CD [3]. The statistical data indicate that the ePOR rate in the first year ranges from 30 to 90%, with significant heterogeneity [9].

The precise mechanisms underlying postoperative recurrence of CD is unclear but is currently thought to be related to age and smoking [10]. The American Gastroenterological Association (AGA) stated in 2020 that high-risk factors for recurrence after enterectomy included age at diagnosis of < 30 years, active smoking, and two or more surgeries for penetrating disease with or without a surgical history of perianal disease, while low-risk factors included age at diagnosis of > 50 years, non-smoking, and disease duration of > 10 years [3]. However, the relative impact of individual risk factors is not explained and there is a lack of systematic evaluation and clinical decisions to validate these risk factors.

Despite significant advancements in novel therapeutic techniques regarding the pathogenesis of CD recurrence and prophylactic interventions, the risk factors for CD recurrence remain unclear. There is a lack of systematic review of risk factors for postoperative recurrence with ePOR as an outcome indicator. Therefore, the present study aimed to investigate the ePOR rate and risk factors through meta-analysis and systematic review, hoping to develop preventive interventions for postoperative recurrence and enhance the quality of life and the prognosis of CD patients.

## Methods

The protocol was designed following the Preferred Reporting Items for Systematic Evaluation and Meta-Analysis Protocols (PRISMA) guidelines [11]. This study was registered in the International Prospective Register of Systematic Reviews (PROSPERO) (CRD42023483671).

### Search strategy

Four databases PubMed, Cochrane Library, Embase, and Web of Science were comprehensively searched for English articles from inception to 17 October 2023, supplemented by manual searches. The searches were conducted using a combination of medical subject terms and keywords using (“Crohn Disease*” OR “Crohn’s Enteritis” OR “Crohn’s Disease”) AND (“Recurrence*” OR “Recurrences” OR “Relapse " OR “Relapses”) AND (“Risk Factors*” OR “Factor, Risk " OR “Risk Factor”). The detailed search strategy is exhibited in Supplementary Material 1.

### Article screening

The articles included were independently reviewed by two authors. After importing the retrieved articles into EndNote X9 to remove duplicates, two researchers independently implemented literature screening, data extraction, and cross-checking. A third researcher was involved in resolving disagreement until a consensus was reached. According to the eligibility criteria, the initial screening was conducted by reading the title and abstract. After the removal of irrelevant literature, the full text was read for secondary screening to determine the final inclusion.

### Inclusion criteria

Inclusion criteria were as follows: (1) cohort studies or case-control studies; (2) The exposure group was CD patients who underwent initial radical surgery and had ePOR at any time after surgery. Specific outcome measures were Rutgeerts score ≥ i2 or modified Rutgeerts score ≥ i2b assessed by endoscopy, or recurrence of ulcer, inflammation, and other symptoms detected by other imaging tests; (3) the primary outcome metric was a multivariate analysis of risk factors for ePOR, and the secondary outcome metric was the ePOR rate in CD patients.

### Exclusion criteria

Articles were excluded for the following reasons: (1) conference abstracts, study protocols, or letters; (2) duplicates; (3) incomplete data or unavailable data; (4) with clinical recurrence or reoperation as the outcome metric; (5) with children with CD as the study population.

### Data extraction

Data were extracted independently by 2 evaluators and cross-checked to ensure consistency. A third evaluator was consulted if necessary. The extracted information included first author, publication year, country, study type, sample size, mean age, gender, number of ePOR, and risk factors.

### Quality assessment

The included studies encompassed case-control studies and observational cohort studies. The National Institutes of Health (NIH) quality assessment tool was used to evaluate the quality of included studies, and each of the questions was representative of an aspect of the included study [12]. Scores of “9 to 12”, “5 to 8”, and “0 to 4” indicated “good”, “fair”, and “poor” quality of case-control studies, respectively. And “11–14,” “6–10,” and “0–5” indicated “good”, “fair” and “poor” quality of observational cohort studies [12]. The greater the risk of bias, the lower the quality. Two researchers scored these studies independently, and in case of disagreement, a third researcher determined the score.

### Statistical analyses

Data were statistically analyzed using Stata 18.0. The risk value of each study was described as odd ratios (ORs). ORs and 95% confidence intervals (CI) were computed to summarize the ePOR rate and risk factors in CD patients. Due to the limited articles, subgroup analysis of ePOR rates was only performed for different continents and study types. Based on the results of heterogeneity tests and I^2^ statistics, the corresponding model was adopted to calculate the OR of risk factors for ePOR. The random-effects model was employed if I^2^ > 50%, and the fixed-effects model was adopted if I^2^ ≤ 50%. For I^2^ > 50%, the leave-one-out method was adopted for sensitivity analysis, and publication bias was appraised using the Egger test with a level of α = 0.05. Differences were considered statistically significant at *P* < 0.05.

## Results

### Screening results

**Fig. 1:**
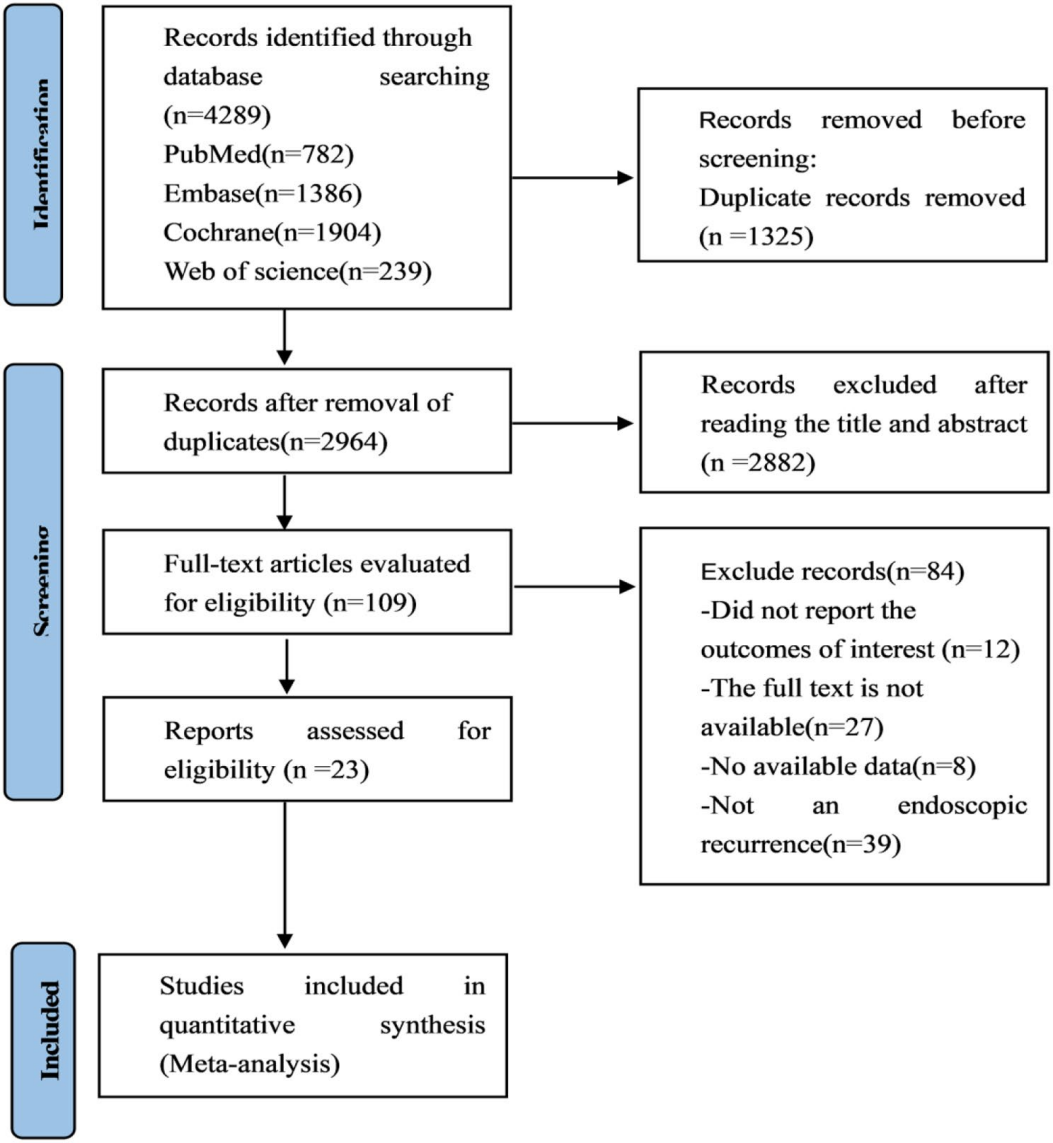
PRISMA flow diagram of the study process

4289 relevant articles were searched from PubMed, Cochrane Library, Embase, and Web of Science databases. After removal of 1325 duplicate, 82 articles were initially obtained, and 23 articles were finally included. The flow diagram of literature screening is shown in Fig. 1.

### Basic features

Of the included 23 studies published from 2015 to 2023, 5 were case-control studies [13–17] and 18 were cohort studies [18–35]. Among them, 3 were from the Netherlands [19, 20, 33], 3 each from Italy [13, 23, 24] and China [15, 28, 31], 4 each from France [18, 25, 30, 32] and the United States [21, 26, 27, 29], and 1 each from Korea [14], Belgium [22], Portugal [16], Japan [34], Israel [35], and Brazil [17]. The sample size of CD patients in each study ranged from 40 to 34, the number of patients with ePOR ranged from 23 to 169, and the age of the study population ranged from 24 to 49 years. Specific literature characteristics are shown in Table 1. Quality assessment revealed that all studies scored ≥ 8, indicating a high overall quality of the included studies. The specific quality assessment is displayed in Table 2.

**Table 1:** Summary of article characteristics

Study	Year	Country	Area	Sample size	Endoscopic Recurrence	Gender (Male/Female)	mean age
Joustra	2022	Netherlands	Europe	142	68	62/80	33
Arkenbosch	2023	Netherlands	Europe	213	64	74/139	34.5
Auzolle	2018	France	Europe	225	107	104/121	34.6
Azzam	2022	America	North America	105	25	56/49	36.99
Bislenghi	2023	Belgium	Europe	127	97	52/75	-
Carvello	2023	Italy	Europe	262	145	125/137	34.75
Coletta	2019	Italy	Europe	193	102	107/86	49
D’Amico	2023	Italy	Europe	141	99	81/60	45
Decousus	2016	France	Europe	75	52	30/45	-
Gaytan	2023	America	North America	107	28	52/55	42.7
Glick	2018	America	North America	70	26	41/29	36.5
Guo	2022	China	Asian	84	37	60/24	-
Hollis	2020	America	North America	193	23	108/85	43
Kim	2021	Korea	Asian	218	110	143/75	-
Li	2015	China	Asian	72	26	49/23	34.18
Maggirori	2019	France	Europe	346	169	200/146	37
Monterio	2017	Portugal	Europe	42	25	21/21	-
Shen	2018	China	Asian	40	/	25/15	-
Tyrode	2023	France	Europe	85	44	48/37	34.9
Wasman	2020	Netherlands	Europe	106	56	36/70	-
Yamada	2021	Japan	Asian	89	80	41/48	36
Yanai	2022	Israel	Asian	297	124	166/131	24
de Barcelos	2015	Brazil	South America	127	43	75/52	33

**Table 2:** Risk of Bias Appraisal for Observational Cohort studies

Study	1	2	3	4	5	6	7	8	9	10	11	12	13	14	Quality Rating
Joustra	Y	Y	Y	Y	N	Y	N	Y	Y	N	Y	N	Y	Y	Fair
Arkenbosch	Y	Y	Y	Y	N	Y	Y	Y	Y	N	Y	Y	Y	N	Good
Auzolle	Y	Y	Y	Y	N	Y	Y	Y	Y	N	Y	Y	Y	Y	Good
Azzam	Y	Y	Y	Y	Y	Y	Y	Y	Y	N	Y	N	Y	Y	Good
Bislenghi	Y	Y	Y	Y	N	Y	Y	Y	Y	N	Y	N	Y	N	Fair
Coletta	Y	Y	Y	Y	N	Y	Y	Y	Y	N	Y	N	N	N	Fair
D’Amico	Y	Y	Y	Y	N	Y	Y	Y	N	N	Y	N	Y	Y	Fair
Decousus	Y	Y	Y	Y	Y	Y	Y	Y	Y	N	Y	N	Y	N	Good
Gaytan	Y	Y	Y	Y	N	Y	Y	Y	Y	N	Y	N	Y	Y	Good
Glick	Y	Y	Y	Y	Y	Y	Y	Y	Y	N	Y	Y	Y	Y	Good
Guo	Y	Y	Y	Y	N	Y	Y	Y	Y	N	Y	Y	NR	Y	Good
Hollis	Y	Y	Y	Y	N	Y	Y	Y	Y	N	Y	N	Y	N	Fair
Maggirori	Y	Y	Y	Y	N	Y	Y	Y	Y	N	Y	Y	Y	N	Good
Shen	Y	Y	Y	Y	N	Y	Y	Y	Y	N	Y	Y	Y	N	Good
Tyrode	Y	Y	Y	Y	N	Y	Y	Y	Y	N	Y	Y	Y	Y	Good
Wasman	Y	Y	Y	Y	N	Y	Y	Y	Y	N	Y	Y	Y	N	Good
Yamada	Y	Y	Y	Y	N	Y	Y	Y	Y	N	Y	Y	Y	N	Good
Yanai	Y	Y	Y	Y	N	Y	Y	Y	Y	N	Y	Y	Y	N	Good

Y: yes; N: no; NR: not recorded

1: Was the research question or objective in this paper clearly stated? 2: Was the study population clearly specified and defined? 3: Was the participation rate of eligible persons at least 50%? 4: Were all the subjects selected or recruited from the same or similar populations? Were inclusion and exclusion criteria for being in the study prespecified and applied uniformly to all participants? 5: Was a sample size justification, power description, or variance and effect estimates provided? 6: For the analyses in this paper, were the exposure(s) of interest measured prior to the outcome(s) being measured? 7: Was the timeframe sufficient so that one could reasonably expect to see an association between exposure and outcome if it existed? 8: For exposures that can vary in amount or level, did the study examine different levels of the exposure as related to the outcome? 9: Were the exposure measures clearly defined, valid, reliable, and implemented consistently across all study participants? 10: Was the exposure(s) assessed more than once over time? 11: Were the outcome measures clearly defined, valid, reliable, and implemented consistently across all study participants? 12: Were the outcome assessors blinded to the exposure status of participants? 13: Was loss to follow-up after baseline 20% or less? 14: Were key potential confounding variables measured and adjusted statistically for their impact on the relationship between exposure(s) and outcome(s)?

### ePOR rate in CD patients and subgroup analysis

#### Meta-analysis of ePOR rate in CD patients

Among the included 23 articles, 22 studies mentioned the ePOR rate. Due to heterogeneity (I^2^ = 96.7%, *P* = 0.000), the random-effects model was utilized. The results showed that the ePOR rate was [ES = 0.48, 95% CI (0.39, 0.57)]. Due to significant heterogeneity, sensitivity analysis was performed by excluding the literature one by one. The results revealed low sensitivity and stable results. Egger’s test indicated a small possibility of publication bias (*P* = 0.322).

#### Meta-analysis of subgroup analysis

Of the 22 articles, 12 were from Europe [13, 18–20, 22–25, 30, 36], 4 from North America [21, 26, 27, 29], 5 from Asia [14, 15, 28, 34, 35], and 1 from South America [17]. The results demonstrated that the ePOR rate was [ES = 0.55, 95% CI (0.47, 0.63)] in Europe, [ES = 0.24, 95% CI (0.13, 0.35)] in North America, [ES = 0.53, 95% CI (0.32, 0.73)] in Asia, and [ES = 0.34, 95% CI (0.26, 0.42)] in South America. As for study types, 17 were observational cohort studies [18–30, 32–35] and 5 were case-control studies [13–17]. The results showed that the ePOR rate of observational cohort studies was [ES = 0.48, 95%CI (0.38, 0.59)] and the ePOR rate of case-control studies was [ES = 0.48, 95%CI (0.39, 0.57)]. The details are presented in Supplementary Material 2.

### Multivariate meta-analysis of risk factors for ePOR

#### Smoking

**Fig. 2:**
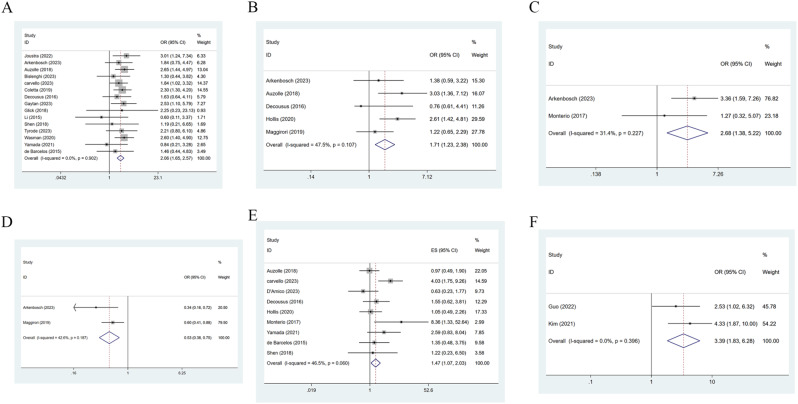
(**A**) Forest plot of smoking; (**B**) Forest plot of previous colonic ileal resection; (**C**) Forest plot of disease localization at ileocolic resection; (**D**) Forest plot of postoperative prophylactic medication; (**E**) Forest plot of Perianal disease; (**F**) Forest plot of anastomotic scattered ulcer

15 studies [13, 15, 17–20, 22, 23, 25–27, 31–34] mentioned smoking. Due to low heterogeneity (I^2^ = 0, *P* = 0.902), a fixed-effects model was applied, and the result suggested that smoking was a risk factor for ePOR [OR = 2.06, 95% CI (1.65, 2.57), *P* = 0.0001] (Fig. 2A; Table 3).

**Table 3:** Risk of Bias Appraisal for Case-Control studies

Study	1	2	3	4	5	6	7	8	9	10	11	12	Quality Rating
Carvello	Y	Y	N	Y	Y	Y	Y	NR	Y	Y	Y	N	Good
Kim	Y	Y	N	Y	Y	Y	Y	N	N	Y	Y	N	Fair
Li	Y	Y	N	Y	Y	Y	Y	NR	Y	Y	Y	Y	Good
Monterio	Y	Y	N	Y	Y	Y	Y	NR	Y	Y	Y	N	Good
de Barcelos	Y	Y	N	Y	Y	Y	Y	NR	Y	Y	Y	N	Good

Y: yes; N: no; NR: not recorded

1: Was the research question or objective in this paper clearly stated and appropriate? 2: Was the study population clearly specified and defined? 3: Did the authors include a sample size justification? 4: Were controls selected or recruited from the same or similar population that gave rise to the cases? 5: Were the definitions, inclusion and exclusion criteria, algorithms or processes used to identify or select cases and controls valid, reliable, and implemented consistently across all study participants? 6: Were the cases clearly defined and differentiated from controls? 7: If less than 100% of eligible cases and/or controls were selected for the study, were the cases and/or controls randomly selected from those eligible? 8: Was there use of concurrent controls? 9: Were the investigators able to confirm that the exposure/risk occurred prior to the development of the condition or event that defined a participant as a case? 10: Were the measures of exposure/risk clearly defined, valid, reliable, and implemented consistently (including the same time period) across all study participants? 11: Were the assessors of exposure/risk blinded to the case or control status of participants? 12: Were key potential confounding variables measured and adjusted statistically in the analyses? If matching was used, did the investigators account for matching during study analysis?

**Table 4:** Multivariate meta-analysis

Risk factors	No. of study	Heterogeneity		OR (95%CI)	*P*	Egger
		I^2^(%)	*P*			
Smoking	15	0	0.902	2.06 (1.65, 2.57)	0.0001	0.012
Postoperative cessation	3	82.8	0.003	1.61 (0.45, 5.75)	0.463	0.030
Previous ileocolonic resection	5	47.5	0.107	1.71 (1.23, 2.38)	0.002	0.525
The age at diagnosis	3	77.5	0.0012	1.53 (0.67, 3.50)	0.315	0.260
Disease localization at ICR	2	31.4	0.227	2.68 (1.38, 5.22)	0.004	-
Penetrating disease behavior	10	70.3	0.0001	1.05 (0.71, 1.57)	0.797	0.614
Postoperative prophylactic medication	2	42.6	0.187	0.53 (0.38, 0.75)	0.0001	-
Gender (Female)	6	62.9	0.019	1.18 (0.76, 1.83)	0.463	0.821
Age	3	28.7	0.246	0.99 (0.97,1.01)	0.476	0.740
Perianal disease	9	46.5	0.060	1.47 (1.07, 2.03)	0.017	0.326
Pre-operative anti‐TNFα	4	52.8	0.096	0.93 (0.49, 1.75)	0.812	0.972
Type of anastomosis	2	0	0.456	0.92 (0.49, 1.74)	0.806	-
End-to-end Anastomosis	2	0	0.655	1.13 (0.60, 2.14)	0.706	-
Handsewn Anastomosis	2	18.4	0.268	1.31 (0.94, 1.82)	0.106	-
Time from diagnosis to surgery	2	75.5	0.043	1.01 (0.94, 1.08)	0.757	-
Fistulizing Disease	2	0	0.906	0.58 (0.33, 1.03)	0.063	0
ASA class	2	0	0.345	0.86 (0.48, 1.56)	0.626	-
Anastomotic scattered ulcer	2	0	0.396	3.39 (1.83, 6.28)	0.001	-
Crohn’s disease-related surgery	3	0	0.729	0.94 (0.42, 2.07)	0.870	0.540

#### Previous ileocolonic resection

5 studies [18, 20, 25, 29, 30] mentioned previous ileocolonic resection. Due to low heterogeneity (I^2^ = 47.5%, *P* = 0.902), a random-effects model was utilized, and the result suggested that previous ileocolonic resection was a risk index for ePOR, with statistical significance [OR = 1.71, 95% CI (1.23, 2.38), *P* = 0.002] (Fig. 2B; Table 3).

#### Disease localization at ileocolic resection

2 studies [16, 20] mentioned disease localization at ileocolic resection. Due to low heterogeneity (I^2^ = 31.4%, *P* = 0.227), a random-effects model was utilized, and the result suggested that disease localization at ileocolic resection was a risk factor for ePOR, with statistical significance [OR = 2.68, 95% CI (1.38, 5.22), *P* = 0.004] (Fig. 2C; Table 3).

#### Postoperative prophylactic medication

2 studies [20, 30] mentioned postoperative prophylactic medication. Due to low heterogeneity (I^2^ = 42.6%, *P* = 0.187), a random-effects model was utilized, and the result revealed that postoperative prophylactic medication was a risk index for ePOR, with marked difference [OR = 0.53, 95% CI (0.38, 0.75), *P* = 0.0001] (Fig. 2D; Table 3).

#### Perianal disease

9 studies [13, 16–18, 24, 25, 29, 31, 34] mentioned perianal disease. Due to low heterogeneity (I^2^ = 46.5%, *P* = 0.060), a random-effects model was utilized, and the result demonstrated that perianal disease was a risk index for ePOR, with marked difference [OR = 1.47, 95% CI (1.07, 2.03), *P* = 0.017] (Fig. 2E; Table 3).

#### Anastomotic scattered ulcer

2 studies [14, 28] mentioned anastomotic scattered ulcers. Due to low heterogeneity (I^2^ = 0, *P* = 0.396), a random-effects model was utilized, and the result suggested that anastomotic scattered ulcer was a risk factor for ePOR, with marked difference [OR = 3.39, 95% CI (1.83, 6.28), *P* = 0.001] (Fig. 2F; Table 3).

#### Other factors

The results showed that postoperative cessation of smoking, age at diagnosis, penetrating disease behavior, female, age, fistulizing disease, preoperative anti-TNFα, type of anastomosis, end-to-end anastomosis, handsewn anastomosis, time from diagnosis to surgery, ASA class, and CD-related surgery were not statistically significant (Table 4).

### Publication bias

Publication bias was evaluated using the Egger test for each multivariate indicator. The P values for smoking and postoperative cessation of smoking were 0.012 and 0.030, which were less than 0.05, suggesting the presence of publication bias. Other risk factors did not exhibit publication bias (Table 3).

## Discussion

CD is a lifelong condition characterized by frequent relapses that significantly impact the daily activities and quality of life of CD patients. This paper summarized the risk factors for ePOR in CD patients through meta-analysis to provide early prevention strategies for high-risk patients, thereby reducing the ePOR rate and improving patients’ quality of life. The quality assessment by the NIH rated 17 studies as good quality and 6 studies as fair, and the meta-analysis results were relatively reliable.

The results suggested that smoking, previous colonic ileal resection, disease localization at ileocolic resection, perianal disease, and anastomotic scattered ulcer were independent risk factors for ePOR. Postoperative prophylactic medication was a protective factor for ePOR. Some independent risk factors and protective factors were identical to the findings of established risk assessment tools, further confirming the validity of the risk assessment tool.

The present systematic review also reconfirmed that smoking shortened the time to ePOR in CD patients, which was consistent with the widely reported finding that smoking was a risk factor for clinical, endoscopic, and surgical recurrence of CD. Auzolle et al. [18] noted that CD patients who smoked had three times the ePOR rate than non-smokers. Passive smoking also increased the risk of ePOR in CD patients [37]. This might be related to microvascular changes in the intestinal mucosa caused by nicotine and carbon monoxide produced in smoke, resulting in ischemia, chronic inflammation, ulceration, and fibrosis in the gut [38]. In addition, nicotine affected intestinal flora and disrupted intestinal homeostasis [39]. Early smoking cessation interventions and preventive education for CD patients could reduce the recurrence rate and improve quality of life. This study identified that previous ileocolonic resection was notably associated with an elevated risk of ePOR. Intestinal surgery might be an indicator of disease invasion, which was related to intestinal involvement due to previous diseases and surgeries [29]. An investigation elicited a higher percentage of recurrence in CD patients who underwent multiple intestinal resections than in the non-recurrence group, consistent with our findings [40]. It is recommended that postoperative monitoring and follow-up of high-risk patients with previous intestinal surgery should be strengthened to emphasize the importance of maintenance therapy and to guide patients to avoid risk factors associated with recurrence, thereby reducing the recurrence rate. In addition, disease localization at ileocolic resection was significantly associated with postoperative recurrence. Studies found that CD patients whose lesions were confined to the ileum had a higher recurrence rate than those whose lesions were in the ileocolic or confined to the colonic site [41]. The ileal CD was more likely to be diffuse and thus involved many regions of the small intestine, resulting in multiple lesions [42]. In contrast, surgical resection for ileocecal lesions was limited in scope, targeting only the site where symptoms appeared, leading to recurrence in the unresected site [42]. Medical practitioners should carefully choose treatment modalities considering the disease characteristics, phenotypes, and patient’s needs, thereby improving the prognosis. The perianal disease was also an independent risk factor for ePOR. Perianal lesions included perianal skin lesions, anal canal lesions, and perianal abscesses, which accelerated the course of CD, thereby increasing the ePOR rate [43]. Previous cohort studies also confirmed a higher rate of ePOR in CD patients with perianal diseases, but with great heterogeneity, which was further supported by the present study [44]. The anastomotic scattered ulcer was also an independent risk factor for ePOR. Anastomotic ulcers were a common complication after surgery in CD patients, mainly due to reduced blood flow at the anastomotic site after surgery or the effect of sutures on the surgical site, resulting in ischemic changes at the anastomotic site [45]. However, the underlying mechanism of anastomotic ulcers in recurrence was currently unknown. It may be related to the surgically-induced decrease in the density of lymphatic vessels in the mucosal and submucosal layers [45, 46]. To address the two risk factors, this study suggested postoperative monitoring for high-risk patients and postoperative endoscopic tests on time to achieve early detection, early prevention, and early treatment. In the future, new surgical methods and biological agents can be used more widely, and multidisciplinary cooperation can be strengthened to bring a brighter future for CD patients.

Our results revealed that postoperative prophylactic medication could protect against ePOR, which was widely established. In the included articles, anti-TNF therapy was the main treatment modality. The meta-analysis by Carla-Moreau et al. [47] also suggested that anti-TNF therapy was the best treatment modality against postoperative recurrence in CD patients. The ECCO guidelines also recommended active prophylactic medication for high-risk CD patients [48]. Therefore, the present study suggested that healthcare professionals should provide early chemoprophylaxis for high-risk individuals according to their postoperative situation.

**Limitation**.

The limitations of this study were as follows: first, the study only performed a meta-analysis of combined risk factors, but other risk factors for ePOR could not be included due to insufficient articles. Second, the diagnosis time of the recurrence rate in the included articles was not consistent, which might partly explain the high variability of recurrence rate. Third, the limited number of articles made it difficult to perform detailed subgroup analyses. Fourth, residual confounders could not be ruled out owing to limited original studies, which may have biased the effect estimates. Fianlly, the language of the included studies was limited to English, which limited the inclusion of studies in other languages, resulting in the limitation of the comprehensiveness of the included literature and increasing the risk of language bias in this study. In the future, more high-quality, prospective, multicenter, large-sample studies should be carried out to verify and enrich the risk factors associated with ePOR in CD patients.

## Conclusion

The results showed that the ePOR rate was higher in CD patients and varied according to the region. In addition, smoking, previous colonic ileal resection, disease localization at ileocolic resection, perianal disease, and anastomotic scattered ulcer were independent risk factors for ePOR in CD patients, and prophylactic medication was a protective factor. Clinicians can further refine current risk assessment tools to incorporate these indicators for early diagnosis and intervention in postoperative CD patients. Our understanding of the causes of CD recurrence is still insufficient, and more prospective, large-sample studies are needed to explore the risk factors for postoperative recurrence.

### Electronic supplementary material

Below is the link to the electronic supplementary material.

Supplementary Material 1

Supplementary Material 2

## Data Availability

The datasets used and analyzed in the current study are available from the corresponding author on reasonable request.

## Abbreviations

CD: Crohn’s disease
ePOR: Endoscopic postoperative recurrence
ORs: Odd ratios
CI: Confidence intervals

## References

[1] Roda G, Chien Ng S, Kotze PG, Argollo M, Panaccione R, Spinelli A, Kaser A, Peyrin-Biroulet L, Danese S (2020). Crohn’s disease. Nat Reviews Disease Primers.

[2] Peyrin-Biroulet L, Loftus EV, Colombel JF, Sandborn WJ (2011). Long-term complications, extraintestinal manifestations, and mortality in adult Crohn’s disease in population-based cohorts. Inflamm Bowel Dis.

[3] Lightner AL, Vogel JD, Carmichael JC, Keller DS, Shah SA, Mahadevan U, Kane SV, Paquette IM, Steele SR, Feingold DL (2020). The American Society of Colon and rectal surgeons Clinical Practice guidelines for the Surgical Management of Crohn’s Disease. Dis Colon Rectum.

[4] Bouguen G (2011). Surgery for adult Crohn’s disease: what is the actual risk?. Gut.

[5] Shinagawa T, Hata K, Ikeuchi H, Fukushima K, Futami K, Sugita A, Uchino M, Watanabe K, Higashi D, Kimura H (2020). Rate of Reoperation decreased significantly after Year 2002 in patients with Crohn’s Disease. Clin Gastroenterol Hepatol.

[6] Candia R, Bravo-Soto GA, Monrroy H, Hernandez C, Nguyen GC (2020). Colonoscopy-guided therapy for the prevention of post-operative recurrence of Crohn’s disease. Cochrane Database Syst Rev.

[7] Rocha CH, Walshe M, Birch S, Sabic K, Korie U, Chasteau C, Miladinova VM, Sabol WB, Mengesha E, Hanna M (2023). Clinical predictors of early and late endoscopic recurrence following ileocolonic resection in Crohn’s disease. J Crohns Colitis.

[8] Ble A, Renzulli C, Cenci F, Grimaldi M, Barone M, Sedano R, Chang J, Nguyen TM, Hogan M, Zou G (2022). The relationship between endoscopic and clinical recurrence in postoperative Crohn’s Disease: a systematic review and Meta-analysis. J Crohns Colitis.

[9] Buisson A, Chevaux JB, Allen PB, Bommelaer G, Peyrin-Biroulet L (2012). Review article: the natural history of postoperative Crohn’s disease recurrence. Aliment Pharmacol Ther.

[10] De Cruz P, Kamm MA, Prideaux L, Allen PB, Desmond PV (2012). Postoperative recurrent luminal Crohn’s disease: a systematic review. Inflamm Bowel Dis.

[11] Liberati A, Altman DG, Tetzlaff J, Mulrow C, Gøtzsche PC, Ioannidis JP, Clarke M, Devereaux PJ, Kleijnen J, Moher D (2009). The PRISMA statement for reporting systematic reviews and meta-analyses of studies that evaluate health care interventions: explanation and elaboration. PLoS Med.

[12] NIH. Study Quality Assessment Tools. 2021. Available online: https://www.nhlbi.nih.gov/health-topics/study-quality-assessment-tools (accessed on 9 April 2024).

[13] Carvello M, D’Hoore A, Maroli A, Cuenca C, Vermeire S, Danese S, Bislenghi G, Spinelli A (2023). Postoperative complications are Associated with an early and increased rate of Disease Recurrence after surgery for Crohn’s Disease. Dis Colon Rectum.

[14] Kim JY, Park SH, Park JC, Noh S, Lee JS, Kim J, Ham NS, Oh EH, Hwang SW, Yang DH (2021). The clinical significance of Anastomotic Ulcers after Ileocolic Resection to predict postoperative recurrence of Crohn’s Disease. Dig Dis Sci.

[15] Li Y, Stocchi L, Liu X, Rui Y, Liu G, Remzi FH, Shen B (2015). Presence of Granulomas in Mesenteric Lymph Nodes is Associated with postoperative recurrence in Crohn’s Disease. Inflamm Bowel Dis.

[16] Monteiro S, Cúrdia Gonçalves T, Boal Carvalho P, Moreira MJ, Cotter J (2017). Updating predictors of endoscopic recurrence after ileocolic resection for Crohn disease. Turk J Gastroenterol.

[17] de Barcelos IF, Kotze PG, Spinelli A, Suzuki Y, Teixeira FV, de Albuquerque IC, Saad-Hossne R, da Silva Kotze LM, Yamamoto T (2017). Factors affecting the incidence of early endoscopic recurrence after ileocolonic resection for Crohn’s disease: a multicentre observational study. Colorectal Dis.

[18] Auzolle C, Nancey S, Tran-Minh ML, Buisson A, Pariente B, Stefanescu C, Fumery M, Marteau P, Treton X, Hammoudi N (2018). Male gender, active smoking and previous intestinal resection are risk factors for post-operative endoscopic recurrence in Crohn’s disease: results from a prospective cohort study. Aliment Pharmacol Ther.

[19] Joustra V, Duijvestein M, Mookhoek A, Bemelman W, Buskens C, Koželj M, Novak G, Hindryckx P, Mostafavi N, D’Haens G (2022). Natural history and risk stratification of recurrent Crohn’s Disease after Ileocolonic Resection: a Multicenter Retrospective Cohort Study. Inflamm Bowel Dis.

[20] Arkenbosch JHC, Beelen EMJ, Dijkstra G, Romberg-Camps M, Duijvestein M, Hoentjen F, van der Marel S, Maljaars PWJ, Jansen S, de Boer NKH (2023). Prophylactic medication for the Prevention of Endoscopic recurrence in Crohn’s Disease: a prospective study based on clinical risk stratification. J Crohns Colitis.

[21] Azzam N, AlRuthia Y, Al Thaher A, Almadi M, Alharbi O, Altuwaijri M, Alshankiti S, Alanazi M, Alanazi A, Aljebreen A (2022). Rate and risk factors of postoperative endoscopic recurrence of moderate- to high-risk Crohn’s disease patients - a real-world experience from a middle eastern cohort. Saudi J Gastroenterol.

[22] Bislenghi G, Vancoillie PJ, Fieuws S, Verstockt B, Sabino J, Wolthuis A, D’Hoore A (2023). Effect of anastomotic configuration on Crohn’s disease recurrence after primary ileocolic resection: a comparative monocentric study of end-to-end versus side-to-side anastomosis. Updates Surg.

[23] Coletta M, Zefelippo A, Mazza S, D’Abrosca V, Botti F, Oreggia B, Prati M, Boni L, Vecchi M, Caprioli F (2019). Previous colonic resection is a risk factor for surgical relapse in Crohn’s disease. Dig Liver Dis.

[24] D’Amico F, Tasopoulou O, Fiorino G, Zilli A, Furfaro F, Allocca M, Sileri P, Spinelli A, Peyrin-Biroulet L, Danese S (2023). Early biological therapy in operated Crohn’s Disease patients is Associated with a lower rate of endoscopic recurrence and Improved Long-Term outcomes: a single-center experience. Inflamm Bowel Dis.

[25] Decousus S, Boucher AL, Joubert J, Pereira B, Dubois A, Goutorbe F, Déchelotte PJ, Bommelaer G, Buisson A (2016). Myenteric plexitis is a risk factor for endoscopic and clinical postoperative recurrence after ileocolonic resection in Crohn’s disease. Dig Liver Dis.

[26] Gaytan-Fuentes IA, Ore AS, Vigna C, Cordova-Cassia CA, Crowell KT, Fabrizio AC, Cataldo TE, Messaris E (2023). Perioperative use of NSAIDs and the risk of short-term endoscopic recurrence in Crohn’s disease patients: a retrospective cohort study. Am J Surg.

[27] Glick LR, Sossenheimer PH, Ollech JE, Cohen RD, Hyman NH, Hurst RD, Rubin DT (2019). Low-dose metronidazole is Associated with a decreased rate of endoscopic recurrence of Crohn’s Disease after Ileal Resection: a retrospective cohort study. J Crohns Colitis.

[28] Guo Z, Zhu Y, Xu Y, Cao L, Li Y, Gong J, Wang Z, Zhu W (2022). Endoscopic evaluation at 1 Month after Ileocolic Resection for Crohn’s Disease predicts future postoperative recurrence and is safe. Dis Colon Rectum.

[29] Hollis RH, Smith N, Sapci I, Click B, Regueiro M, Hull TL, Lightner AL (2022). Small Bowel Crohn’s Disease recurrence is common after total proctocolectomy for Crohn’s colitis. Dis Colon Rectum.

[30] Maggiori L, Brouquet A, Zerbib P, Lefevre JH, Denost Q, Germain A, Cotte E, Beyer-Berjot L, Munoz-Bongrand N, Desfourneaux V (2019). Penetrating Crohn Disease is not Associated with a higher risk of recurrence after surgery: a prospective Nationwide Cohort conducted by the Getaid Chirurgie Group. Ann Surg.

[31] Shen W, Li Y, Cao L, Cai X, Ge Y, Zhu W (2018). Decreased expression of Prox1 is Associated with postoperative recurrence in Crohn’s Disease. J Crohns Colitis.

[32] Tyrode G, Lakkis Z, Vernerey D, Falcoz A, Clairet V, Alibert L, Koch S, Vuitton L (2023). KONO-S anastomosis is not Superior to Conventional Anastomosis for the reduction of postoperative endoscopic recurrence in Crohn’s Disease. Inflamm Bowel Dis.

[33] Wasmann K, van Amesfoort J, van Montfoort ML, Koens L, Bemelman WA, Buskens CJ (2020). The predictive value of inflammation at Ileocecal Resection Margins for Postoperative Crohn’s recurrence: a Cohort Study. Inflamm Bowel Dis.

[34] Yamada A, Komaki Y, Komaki F, Haider H, Micic D, Pekow J, Dalal S, Cohen RD, Cannon L, Umanskiy K (2021). The correlation between Vitamin D Levels and the risk of postoperative recurrence in Crohn’s Disease. Digestion.

[35] Yanai H, Kagramanova A, Knyazev O, Sabino J, Haenen S, Mantzaris GJ, Mountaki K, Armuzzi A, Pugliese D, Furfaro F (2022). Endoscopic postoperative recurrence in Crohn’s Disease after curative Ileocecal resection with early prophylaxis by Anti-TNF, Vedolizumab or Ustekinumab: a real-world multicentre European study. J Crohns Colitis.

[36] de Buck A, Eshuis EJ, Vermeire S, Van Assche G, Ferrante M, D’Haens GR, Ponsioen CY, Belmans A, Buskens CJ, Wolthuis AM (2017). Short- and medium-term outcomes following primary ileocaecal resection for Crohn’s disease in two specialist centres. Br J Surg.

[37] Scharrer S, Lissner D, Primas C, Reinisch W, Novacek G, Reinisch S, Papay P, Dejaco C, Vogelsang H, Miehsler W (2021). Passive Smoking increases the risk for intestinal surgeries in patients with Crohn’s Disease. Inflamm Bowel Dis.

[38] Papoutsopoulou S, Satsangi J, Campbell BJ, Probert CS (2020). Review article: impact of cigarette smoking on intestinal inflammation-direct and indirect mechanisms. Aliment Pharmacol Ther.

[39] Khasawneh M, Spence AD, Addley J, Allen PB (2017). The role of smoking and alcohol behaviour in the management of inflammatory bowel disease. Best Pract Res Clin Gastroenterol.

[40] Erzin Y, Şişman G, Hatemi İ, Baca B, Hamzaoglu İ, Dirican A, Korman U, Çelik AF (2020). Predictors of endoscopic recurrence in resected patients with Crohn’s disease in a long-term follow-up cohort: history of multiple previous resections and residual synchronous disease in the remnant intestine. Turk J Gastroenterol.

[41] Scaringi S, Di Bella A, Boni L, Giudici F, Di Martino C, Zambonin D, Ficari F (2018). New perspectives on the long-term outcome of segmental colectomy for Crohn’s colitis: an observational study on 200 patients. Int J Colorectal Dis.

[42] Bernell O, Lapidus A, Hellers G (2000). Risk factors for surgery and postoperative recurrence in Crohn’s disease. Ann Surg.

[43] Mak WY, Mak OS, Lee CK, Tang W, Leung WK, Wong MTL, Sze ASF, Li M, Leung CM, Lo FH (2018). Significant Medical and Surgical Morbidity in Perianal Crohn’s Disease: results from a territory-wide study. J Crohns Colitis.

[44] Bernell O, Lapidus A, Hellers G (2000). Risk factors for surgery and recurrence in 907 patients with primary ileocaecal Crohn’s disease. Br J Surg.

[45] Hirten RP, Ungaro RC, Castaneda D, Lopatin S, Sands BE, Colombel JF, Cohen BL (2020). Anastomotic ulcers after Ileocolic Resection for Crohn’s Disease are common and predict recurrence. Inflamm Bowel Dis.

[46] Rahier JF, Dubuquoy L, Colombel JF, Jouret-Mourin A, Delos M, Ferrante M, Sokol H, Hertogh GD, Salleron J, Geboes K (2013). Decreased lymphatic vessel density is associated with postoperative endoscopic recurrence in Crohn’s disease. Inflamm Bowel Dis.

[47] Carla-Moreau A, Paul S, Roblin X, Genin C, Peyrin-Biroulet L (2015). Prevention and treatment of postoperative Crohn’s disease recurrence with anti-TNF therapy: a meta-analysis of controlled trials. Dig Liver Dis.

[48] Gionchetti P, Dignass A, Danese S, Magro Dias FJ, Rogler G, Lakatos PL, Adamina M, Ardizzone S, Buskens CJ, Sebastian S (2017). 3rd European evidence-based Consensus on the diagnosis and management of Crohn’s Disease 2016: part 2: Surgical Management and Special situations. J Crohns Colitis.

