# Supplementary material for: Risk factors for endoscopic postoperative recurrence in patients with Crohn’s Disease: a protocol for systematic review and meta-analysis
Source: BMC Gastroenterol. 2024 Jun 25;24:211. doi: 10.1186/s12876-024-03301-z (PMC11197377; doi:10.1186/s12876-024-03301-z)
Supplement: Supplementary file 1 — Supplementary Material 1 [file 12876_2024_3301_MOESM1_ESM.docx]

**Supplementary material 1 Search history**

1. Queries

| No. | Query | Results | Date |
| --- | --- | --- | --- |
| #47 | #17 AND #25 AND #46 | 1386 | 17-Oct-23 |
| #46 | #26 OR #27 OR #28 OR #29 OR #30 OR #31 OR #32 OR #33 OR #34 OR #35 OR #36 OR #37 OR #38 OR #39 OR #40 OR #41 OR #42 OR #43 OR #44 OR #45 | 1810475 | 17-Oct-23 |
| #45 | 'score, risk factor':ab,ti | 16 | 17-Oct-23 |
| #44 | 'risk factor score':ab,ti | 293 | 17-Oct-23 |
| #43 | 'risk factor scores':ab,ti | 145 | 17-Oct-23 |
| #42 | 'score, risk':ab,ti | 1309 | 17-Oct-23 |
| #41 | 'risk score':ab,ti | 47152 | 17-Oct-23 |
| #40 | 'risk scores':ab,ti | 21984 | 17-Oct-23 |
| #39 | 'populations at risk':ab,ti | 3610 | 17-Oct-23 |
| #38 | 'population at risk':ab,ti | 5856 | 17-Oct-23 |
| #37 | 'correlates, health':ab,ti | 12 | 17-Oct-23 |
| #36 | 'health correlates':ab,ti | 610 | 17-Oct-23 |
| #35 | 'social risk factor':ab,ti | 106 | 17-Oct-23 |
| #34 | 'risk factors, social':ab,ti | 141 | 17-Oct-23 |
| #33 | 'risk factor, social':ab,ti | 7 | 17-Oct-23 |
| #32 | 'factors, social risk':ab,ti | 9 | 17-Oct-23 |
| #31 | 'factor, social risk':ab,ti | 0 | 17-Oct-23 |
| #30 | 'social risk factors':ab,ti | 1364 | 17-Oct-23 |
| #29 | 'factor, risk':ab,ti | 335 | 17-Oct-23 |
| #28 | 'risk factors':ab,ti | 850564 | 17-Oct-23 |
| #27 | 'risk factor':ab,ti | 396747 | 17-Oct-23 |
| #26 | 'risk factor'/exp | 1359963 | 17-Oct-23 |
| #25 | #18 OR #19 OR #20 OR #21 OR #22 OR #23 OR #24 | 978527 | 17-Oct-23 |
| #24 | 'relapses':ab,ti | 55286 | 17-Oct-23 |
| #23 | 'relapse':ab,ti | 257252 | 17-Oct-23 |
| #22 | 'recrudescences':ab,ti | 265 | 17-Oct-23 |
| #21 | 'recrudescence':ab,ti | 3835 | 17-Oct-23 |
| #20 | 'recurrences':ab,ti | 86245 | 17-Oct-23 |
| #19 | 'recurrence':ab,ti | 554899 | 17-Oct-23 |
| #18 | 'recurrent disease'/exp | 217370 | 17-Oct-23 |
| #17 | #1 OR #2 OR #3 OR #4 OR #5 OR #6 OR #7 OR #8 OR #9 OR #10 OR #11 OR #12 OR #13 OR #14 OR #15 OR #16 | 116456 | 17-Oct-23 |
| #16 | 'regional ileitis':ab,ti | 291 | 17-Oct-23 |
| #15 | 'ileitis, regional':ab,ti | 6 | 17-Oct-23 |
| #14 | 'terminal ileitis':ab,ti | 741 | 17-Oct-23 |
| #13 | 'ileitis terminal':ab,ti | 2 | 17-Oct-23 |
| #12 | 'granulomatous colitis':ab,ti | 536 | 17-Oct-23 |
| #11 | 'colitis, granulomatous':ab,ti | 12 | 17-Oct-23 |
| #10 | 'ileocolitis':ab,ti | 768 | 17-Oct-23 |
| #9 | 'enteritis, regional':ab,ti | 5 | 17-Oct-23 |
| #8 | 'granulomatous enteritis':ab,ti | 225 | 17-Oct-23 |
| #7 | 'enteritis, granulomatous':ab,ti | 3 | 17-Oct-23 |
| #6 | 'inflammatory bowel disease 1':ab,ti | 162 | 17-Oct-23 |
| #5 | 'crohns disease':ab,ti | 1089 | 17-Oct-23 |
| #4 | 'regional enteritis':ab,ti | 969 | 17-Oct-23 |
| #3 | 'crohns enteritis':ab,ti | 0 | 17-Oct-23 |
| #2 | 'crohn disease':ab,ti | 5997 | 17-Oct-23 |
| #1 | 'crohn disease'/exp | 114460 | 17-Oct-23 |

2. PubMed Search History

| Search number | Query | Sort By | Filters | Search Details | Results | Time |
| --- | --- | --- | --- | --- | --- | --- |
| 11 | ((("Crohn Disease"[Mesh]) OR ((((((((((((((((Crohn Disease[Title/Abstract]) OR (Crohn's Enteritis[Title/Abstract])) OR (Regional Enteritis[Title/Abstract])) OR (Crohn's Disease[Title/Abstract])) OR (Crohns Disease[Title/Abstract])) OR (Inflammatory Bowel Disease 1[Title/Abstract])) OR (Enteritis, Granulomatous[Title/Abstract])) OR (Granulomatous Enteritis[Title/Abstract])) OR (Enteritis, Regional[Title/Abstract])) OR (Ileocolitis[Title/Abstract])) OR (Colitis, Granulomatous[Title/Abstract])) OR (Granulomatous Colitis[Title/Abstract])) OR (Ileitis Terminal[Title/Abstract])) OR (Terminal Ileitis[Title/Abstract])) OR (Ileitis, Regional[Title/Abstract])) OR (Regional Ileitis[Title/Abstract]))) AND (("Recurrence"[Mesh]) OR ((((((Recurrence[Title/Abstract]) OR (Recurrences[Title/Abstract])) OR (Recrudescence[Title/Abstract])) OR (Recrudescences[Title/Abstract])) OR (Relapse[Title/Abstract])) OR (Relapses[Title/Abstract])))) AND (("Risk Factors"[Mesh]) OR (((((((((((((((((((Factor, Risk[Title/Abstract]) OR (Risk Factors[Title/Abstract])) OR (Risk Factor[Title/Abstract])) OR (Social Risk Factors[Title/Abstract])) OR (Factor, Social Risk[Title/Abstract])) OR (Factors, Social Risk[Title/Abstract])) OR (Risk Factor, Social[Title/Abstract])) OR (Risk Factors, Social[Title/Abstract])) OR (Social Risk Factor[Title/Abstract])) OR (Health Correlates[Title/Abstract])) OR (Correlates, Health[Title/Abstract])) OR (Population at Risk[Title/Abstract])) OR (Populations at Risk[Title/Abstract])) OR (Risk Scores[Title/Abstract])) OR (Risk Score[Title/Abstract])) OR (Score, Risk[Title/Abstract])) OR (Risk Factor Scores[Title/Abstract])) OR (Risk Factor Score[Title/Abstract])) OR (Score, Risk Factor[Title/Abstract]))) | Most Recent |  | ("Crohn Disease"[MeSH Terms] OR ("Crohn Disease"[Title/Abstract] OR "crohn s enteritis"[Title/Abstract] OR "regional enteritis"[Title/Abstract] OR "crohn s disease"[Title/Abstract] OR "crohns disease"[Title/Abstract] OR "inflammatory bowel disease 1"[Title/Abstract] OR "enteritis granulomatous"[Title/Abstract] OR "granulomatous enteritis"[Title/Abstract] OR "enteritis regional"[Title/Abstract] OR "Ileocolitis"[Title/Abstract] OR "colitis granulomatous"[Title/Abstract] OR "granulomatous colitis"[Title/Abstract] OR "ileitis terminal"[Title/Abstract] OR "terminal ileitis"[Title/Abstract] OR "ileitis regional"[Title/Abstract] OR "regional ileitis"[Title/Abstract])) AND ("Recurrence"[MeSH Terms] OR ("Recurrence"[Title/Abstract] OR "Recurrences"[Title/Abstract] OR "Recrudescence"[Title/Abstract] OR "Recrudescences"[Title/Abstract] OR "Relapse"[Title/Abstract] OR "Relapses"[Title/Abstract])) AND ("Risk Factors"[MeSH Terms] OR ("factor risk"[Title/Abstract] OR "Risk Factors"[Title/Abstract] OR "risk factor"[Title/Abstract] OR "social risk factors"[Title/Abstract] OR (("Factor"[All Fields] OR "factor s"[All Fields] OR "Factors"[All Fields]) AND "social risk"[Title/Abstract]) OR "factors social risk"[Title/Abstract] OR "risk factor social"[Title/Abstract] OR "risk factors social"[Title/Abstract] OR "social risk factor"[Title/Abstract] OR "health correlates"[Title/Abstract] OR "correlates health"[Title/Abstract] OR "population at risk"[Title/Abstract] OR "populations at risk"[Title/Abstract] OR "risk scores"[Title/Abstract] OR "risk score"[Title/Abstract] OR "score risk"[Title/Abstract] OR "risk factor scores"[Title/Abstract] OR "risk factor score"[Title/Abstract] OR "score risk factor"[Title/Abstract])) | 782 | 23:19:41 |
| 10 | ("Risk Factors"[Mesh]) OR (((((((((((((((((((Factor, Risk[Title/Abstract]) OR (Risk Factors[Title/Abstract])) OR (Risk Factor[Title/Abstract])) OR (Social Risk Factors[Title/Abstract])) OR (Factor, Social Risk[Title/Abstract])) OR (Factors, Social Risk[Title/Abstract])) OR (Risk Factor, Social[Title/Abstract])) OR (Risk Factors, Social[Title/Abstract])) OR (Social Risk Factor[Title/Abstract])) OR (Health Correlates[Title/Abstract])) OR (Correlates, Health[Title/Abstract])) OR (Population at Risk[Title/Abstract])) OR (Populations at Risk[Title/Abstract])) OR (Risk Scores[Title/Abstract])) OR (Risk Score[Title/Abstract])) OR (Score, Risk[Title/Abstract])) OR (Risk Factor Scores[Title/Abstract])) OR (Risk Factor Score[Title/Abstract])) OR (Score, Risk Factor[Title/Abstract])) | Most Recent |  | "Risk Factors"[MeSH Terms] OR ("factor risk"[Title/Abstract] OR "Risk Factors"[Title/Abstract] OR "risk factor"[Title/Abstract] OR "social risk factors"[Title/Abstract] OR (("Factor"[All Fields] OR "factor s"[All Fields] OR "Factors"[All Fields]) AND "social risk"[Title/Abstract]) OR "factors social risk"[Title/Abstract] OR "risk factor social"[Title/Abstract] OR "risk factors social"[Title/Abstract] OR "social risk factor"[Title/Abstract] OR "health correlates"[Title/Abstract] OR "correlates health"[Title/Abstract] OR "population at risk"[Title/Abstract] OR "populations at risk"[Title/Abstract] OR "risk scores"[Title/Abstract] OR "risk score"[Title/Abstract] OR "score risk"[Title/Abstract] OR "risk factor scores"[Title/Abstract] OR "risk factor score"[Title/Abstract] OR "score risk factor"[Title/Abstract]) | 1,416,925 | 23:19:20 |
| 9 | ("Recurrence"[Mesh]) OR ((((((Recurrence[Title/Abstract]) OR (Recurrences[Title/Abstract])) OR (Recrudescence[Title/Abstract])) OR (Recrudescences[Title/Abstract])) OR (Relapse[Title/Abstract])) OR (Relapses[Title/Abstract])) | Most Recent |  | "Recurrence"[MeSH Terms] OR "Recurrence"[Title/Abstract] OR "Recurrences"[Title/Abstract] OR "Recrudescence"[Title/Abstract] OR "Recrudescences"[Title/Abstract] OR "Relapse"[Title/Abstract] OR "Relapses"[Title/Abstract] | 667,180 | 23:19:06 |
| 8 | ("Crohn Disease"[Mesh]) OR ((((((((((((((((Crohn Disease[Title/Abstract]) OR (Crohn's Enteritis[Title/Abstract])) OR (Regional Enteritis[Title/Abstract])) OR (Crohn's Disease[Title/Abstract])) OR (Crohns Disease[Title/Abstract])) OR (Inflammatory Bowel Disease 1[Title/Abstract])) OR (Enteritis, Granulomatous[Title/Abstract])) OR (Granulomatous Enteritis[Title/Abstract])) OR (Enteritis, Regional[Title/Abstract])) OR (Ileocolitis[Title/Abstract])) OR (Colitis, Granulomatous[Title/Abstract])) OR (Granulomatous Colitis[Title/Abstract])) OR (Ileitis Terminal[Title/Abstract])) OR (Terminal Ileitis[Title/Abstract])) OR (Ileitis, Regional[Title/Abstract])) OR (Regional Ileitis[Title/Abstract])) | Most Recent |  | "Crohn Disease"[MeSH Terms] OR "Crohn Disease"[Title/Abstract] OR "crohn s enteritis"[Title/Abstract] OR "regional enteritis"[Title/Abstract] OR "crohn s disease"[Title/Abstract] OR "crohns disease"[Title/Abstract] OR "inflammatory bowel disease 1"[Title/Abstract] OR "enteritis granulomatous"[Title/Abstract] OR "granulomatous enteritis"[Title/Abstract] OR "enteritis regional"[Title/Abstract] OR "Ileocolitis"[Title/Abstract] OR "colitis granulomatous"[Title/Abstract] OR "granulomatous colitis"[Title/Abstract] OR "ileitis terminal"[Title/Abstract] OR "terminal ileitis"[Title/Abstract] OR "ileitis regional"[Title/Abstract] OR "regional ileitis"[Title/Abstract] | 66,337 | 23:18:49 |
| 7 | ((((((((((((((((((Factor, Risk[Title/Abstract]) OR (Risk Factors[Title/Abstract])) OR (Risk Factor[Title/Abstract])) OR (Social Risk Factors[Title/Abstract])) OR (Factor, Social Risk[Title/Abstract])) OR (Factors, Social Risk[Title/Abstract])) OR (Risk Factor, Social[Title/Abstract])) OR (Risk Factors, Social[Title/Abstract])) OR (Social Risk Factor[Title/Abstract])) OR (Health Correlates[Title/Abstract])) OR (Correlates, Health[Title/Abstract])) OR (Population at Risk[Title/Abstract])) OR (Populations at Risk[Title/Abstract])) OR (Risk Scores[Title/Abstract])) OR (Risk Score[Title/Abstract])) OR (Score, Risk[Title/Abstract])) OR (Risk Factor Scores[Title/Abstract])) OR (Risk Factor Score[Title/Abstract])) OR (Score, Risk Factor[Title/Abstract]) | Most Recent |  | "factor risk"[Title/Abstract] OR "risk factors"[Title/Abstract] OR "risk factor"[Title/Abstract] OR "social risk factors"[Title/Abstract] OR (("Factor"[All Fields] OR "factor s"[All Fields] OR "Factors"[All Fields]) AND "social risk"[Title/Abstract]) OR "factors social risk"[Title/Abstract] OR "risk factor social"[Title/Abstract] OR "risk factors social"[Title/Abstract] OR "social risk factor"[Title/Abstract] OR "health correlates"[Title/Abstract] OR "correlates health"[Title/Abstract] OR "population at risk"[Title/Abstract] OR "populations at risk"[Title/Abstract] OR "risk scores"[Title/Abstract] OR "risk score"[Title/Abstract] OR "score risk"[Title/Abstract] OR "risk factor scores"[Title/Abstract] OR "risk factor score"[Title/Abstract] OR "score risk factor"[Title/Abstract] | 819,599 | 23:18:27 |
| 6 | "Risk Factors"[Mesh] | Most Recent |  | "Risk Factors"[MeSH Terms] | 965,289 | 23:16:11 |
| 5 | (((((Recurrence[Title/Abstract]) OR (Recurrences[Title/Abstract])) OR (Recrudescence[Title/Abstract])) OR (Recrudescences[Title/Abstract])) OR (Relapse[Title/Abstract])) OR (Relapses[Title/Abstract]) | Most Recent |  | "Recurrence"[Title/Abstract] OR "Recurrences"[Title/Abstract] OR "Recrudescence"[Title/Abstract] OR "Recrudescences"[Title/Abstract] OR "Relapse"[Title/Abstract] OR "Relapses"[Title/Abstract] | 547,680 | 23:15:41 |
| 4 | "Recurrence"[Mesh] | Most Recent |  | "Recurrence"[MeSH Terms] | 201,390 | 23:14:56 |
| 3 | (((((((((((((((Crohn Disease[Title/Abstract]) OR (Crohn's Enteritis[Title/Abstract])) OR (Regional Enteritis[Title/Abstract])) OR (Crohn's Disease[Title/Abstract])) OR (Crohns Disease[Title/Abstract])) OR (Inflammatory Bowel Disease 1[Title/Abstract])) OR (Enteritis, Granulomatous[Title/Abstract])) OR (Granulomatous Enteritis[Title/Abstract])) OR (Enteritis, Regional[Title/Abstract])) OR (Ileocolitis[Title/Abstract])) OR (Colitis, Granulomatous[Title/Abstract])) OR (Granulomatous Colitis[Title/Abstract])) OR (Ileitis Terminal[Title/Abstract])) OR (Terminal Ileitis[Title/Abstract])) OR (Ileitis, Regional[Title/Abstract])) OR (Regional Ileitis[Title/Abstract]) | Most Recent |  | "crohn disease"[Title/Abstract] OR "crohn s enteritis"[Title/Abstract] OR "regional enteritis"[Title/Abstract] OR "crohn s disease"[Title/Abstract] OR "crohns disease"[Title/Abstract] OR "inflammatory bowel disease 1"[Title/Abstract] OR "enteritis granulomatous"[Title/Abstract] OR "granulomatous enteritis"[Title/Abstract] OR "enteritis regional"[Title/Abstract] OR "Ileocolitis"[Title/Abstract] OR "colitis granulomatous"[Title/Abstract] OR "granulomatous colitis"[Title/Abstract] OR "ileitis terminal"[Title/Abstract] OR "terminal ileitis"[Title/Abstract] OR "ileitis regional"[Title/Abstract] OR "regional ileitis"[Title/Abstract] | 58,181 | 23:14:01 |
| 2 | "Crohn Disease"[Mesh] | Most Recent |  | "Crohn Disease"[MeSH Terms] | 44,706 | 23:00:51 |

3. Web of Science Core set

| Authority | # | Search mode | database | result | date |
| --- | --- | --- | --- | --- | --- |
| - WOS.IC: 1993 to 2023 - WOS.CCR: 1985 to 2023 - WOS.SCI: 1900 to 2023 - WOS.AHCI: 1975 to 2023 - WOS.BHCI: 2005 to 2023 - WOS.BSCI: 2005 to 2023 - WOS.ESCI: 2005 to 2023 - WOS.ISTP: 1990 to 2023 - WOS.SSCI: 1900 to 2023 - WOS.ISSHP: 1990 to 2023 | 1 | TS=(Crohn Disease) OR TS=(Crohn's Enteritis) OR TS=(Regional Enteritis) OR TS=(Crohn's Disease) OR TS=(Crohns Disease) OR TS=(Inflammatory Bowel Disease 1) OR TS=(Enteritis, Granulomatous) OR TS=(Granulomatous Enteritis) OR TS=(Enteritis, Regional) OR TS=(Ileocolitis) OR TS=(Colitis, Granulomatous) OR TS=(Granulomatous Colitis) OR TS=(Ileitis Terminal) OR TS=(Terminal Ileitis) OR TS=(Ileitis, Regional) OR TS=(Regional Ileitis) | Web of Science Core set | 107729 | Tue Oct 17 2023 12:59:34 GMT+0800 (China Standard Time) |
| - WOS.IC: 1993 to 2023 - WOS.CCR: 1985 to 2023 - WOS.SCI: 1900 to 2023 - WOS.AHCI: 1975 to 2023 - WOS.BHCI: 2005 to 2023 - WOS.BSCI: 2005 to 2023 - WOS.ESCI: 2005 to 2023 - WOS.ISTP: 1990 to 2023 - WOS.SSCI: 1900 to 2023 - WOS.ISSHP: 1990 to 2023 | 2 | TS=(Recurrence) OR TS=(Recurrences) OR TS=(Recrudescence) OR TS=(Recrudescences) OR TS=(Relapse) OR TS=(Relapses) | Web of Science Core set | 657403 | Tue Oct 17 2023 13:01:17 GMT+0800 (China Standard Time) |
| - WOS.IC: 1993 to 2023 - WOS.CCR: 1985 to 2023 - WOS.SCI: 1900 to 2023 - WOS.AHCI: 1975 to 2023 - WOS.BHCI: 2005 to 2023 - WOS.BSCI: 2005 to 2023 - WOS.ESCI: 2005 to 2023 - WOS.ISTP: 1990 to 2023 - WOS.SSCI: 1900 to 2023 - WOS.ISSHP: 1990 to 2023 | 3 | TS=(Risk Factors) OR TS=(Factor, Risk) OR TS=(Risk Factor) OR TS=(Social Risk Factors) OR TS=(Factor, Social Risk) OR TS=(Factors, Social Risk) OR TS=(Risk Factor, Social) OR TS=(Risk Factors, Social) OR TS=(Social Risk Factor) OR TS=(Health Correlates) OR TS=(Correlates, Health) OR TS=(Population at Risk) OR TS=(Populations at Risk) OR TS=(Risk Scores) OR TS=(Risk Score) OR TS=(Score, Risk) OR TS=(Risk Factor Scores) OR TS=(Risk Factor Score) OR TS=(Score, Risk Factor) | Web of Science Core set | 2146194 | Tue Oct 17 2023 13:04:56 GMT+0800 (China Standard Time) |
| - WOS.IC: 1993 to 2023 - WOS.CCR: 1985 to 2023 - WOS.SCI: 1900 to 2023 - WOS.AHCI: 1975 to 2023 - WOS.BHCI: 2005 to 2023 - WOS.BSCI: 2005 to 2023 - WOS.ESCI: 2005 to 2023 - WOS.ISTP: 1990 to 2023 - WOS.SSCI: 1900 to 2023 - WOS.ISSHP: 1990 to 2023 | 4 | #3 AND #2 AND #1 | Web of Science Core set | 1904 | Tue Oct 17 2023 13:06:30 GMT+0800 (China Standard Time) |

4. Cohrane

%0 Journal Article

%A Wenzl, H. H.

%A Primas, C.

%A Novacek, G.

%A Teml, A.

%A Öfferlbauer-Ernst, A.

%A Högenauer, C.

%A Vogelsang, H.

%A Petritsch, W.

%A Reinisch, W.

%D 2015

%T Withdrawal of long-term maintenance treatment with azathioprine tends to increase relapse risk in patients with Crohn's disease

%V 60

%N 5

%P 1414‐1423

%8 2015-01-01

%R 10.1007/s10620-014-3419-5

%K Adult; Anti‐Inflammatory Agents [*administration & dosage]; Austria; Azathioprine [*administration & dosage]; Crohn Disease [diagnosis, *drug therapy]; Double‐Blind Method; Drug Administration Schedule; Early Termination of Clinical Trials; Female; Gastrointestinal Agents [*administration & dosage]; Humans; Male; Middle Aged; Patient Selection; Prospective Studies; Recurrence; Remission Induction; Risk Factors; Time Factors; Treatment Outcome

%X BACKGROUND AND AIM: Many patients with quiescent Crohn's disease are maintained on long‐term treatment with azathioprine (AZA), but controlled data are limited. We aimed to evaluate the efficacy of AZA therapy for more than 4 years to maintain clinical remission. METHODS: We performed a randomized double‐blind placebo‐controlled AZA withdrawal trial with a follow‐up period of 24 months. Patients had to have continuous AZA therapy ≥ 4 years without exacerbation of disease during the 12 months before enrollment, and a Crohn's disease activity index < 150 at baseline. Patients were randomized to continue on AZA or switch to placebo. The primary endpoint was time to clinical relapse during follow‐up. RESULTS: After inclusion of 52 patients, the trial was stopped prematurely due to slow recruitment. During the 2‐year follow‐up, clinical relapse occurred in 4 of 26 (15 %) patients on continued AZA and in 8 of 26 (31 %) patients on placebo. Time to clinical relapse averaged 22.3 months (95 % CI 20.6‐24.0) on AZA and 19.2 months (95 % CI 16.4‐22.1) on placebo (p = 0.20). According to life‐table analysis, the proportion of patients in remission after 12 and 24 months was 96 ± 4 and 86 ± 7 % in patients receiving AZA versus 76 ± 8 and 68 ± 9 % in patients receiving placebo (month 12, p = 0.035; month 24, p = 0.30). A higher AZA dose at enrollment was an independent predictor for relapse (p < 0.05). CONCLUSIONS: AZA withdrawal resulted in a significantly increased relapse risk after 1 year and a nonstatistically significant trend for relapse after 2 years. Our results are in line with previous observations.

%Z Digestive diseases and sciences

Journal article

%U https://cochrane.66557.net/central/doi/10.1002/central/CN-01112574/full

%0 Journal Article

%A Louis, E.

%A Resche-Rigon, M.

%A Laharie, D.

%A Satsangi, J.

%A Ding, N.

%A Siegmund, B.

%A D'Haens, G.

%A Picon, L.

%A Bossuyt, P.

%A Vuitton, L.

%A Et, Al.

%D 2023

%T Withdrawal of infliximab or concomitant immunosuppressant therapy in patients with Crohn's disease on combination therapy (SPARE): a multicentre, open-label, randomised controlled trial

%V 8

%N 3

%P 215‐227

%8 2023-01-01

%R 10.1016/S2468-1253(22)00385-5

%K *Crohn disease; *cancer combination chemotherapy; *cancer patient; *erratum; *relapse; *remission; Adult; Article; Australia; Azathioprine [adverse effects]; Cancer recurrence; Clinical assessment; Clinical trial; Controlled study; Crohn Disease [chemically induced, drug therapy]; Disease exacerbation; Drug combination; Drug therapy; Drug withdrawal; Endoscopy; Europe; European Union; Female; Human; Humans; Immunosuppressive Agents [adverse effects]; Infliximab [adverse effects]; Major clinical study; Male; Malignant neoplasm; Mean survival time; Multicenter study; Randomization; Randomized controlled trial; Recurrence; Recurrence risk; Risk assessment; Treatment failure; Tumor Necrosis Factor Inhibitors [therapeutic use]; Ulcer

%X BACKGROUND: The combination of infliximab and immunosuppressant therapy is a standard management strategy for patients with Crohn's disease. Concerns regarding the implications of long‐term combination therapy provided the rationale for a formal clinical trial of treatment de‐escalation. Our aim was to compare the relapse rate and the time spent in remission over 2 years between patients continuing combination therapy and those stopping infliximab or immunosuppressant therapy. METHODS: This multicentre, open‐label, randomised controlled trial was performed in 64 hospitals in seven countries in Europe and Australia. Adult patients with Crohn's disease in steroid‐free clinical remission for more than 6 months, on combination therapy of infliximab and immunosuppressant therapy for at least 8 months were randomly assigned (1:1:1) to either continue combination therapy (combination group), discontinue infliximab (infliximab withdrawal group), or discontinue immunosuppressant therapy (immunosuppressant withdrawal group). Randomisation was stratified according to disease duration before start of first anti‐TNF treatment (≤2 or >2 years), failure of immunosuppressant therapy before start of infliximab, and presence of ulcers at baseline endoscopy. The patient number and group of each stratum were assigned by a central online randomisation website. Treatment was optimised or resumed in case of relapse in all groups. Participants, those assessing outcomes, and those analysing the data were not masked to group assignment. The coprimary endpoints were the relapse rate (superiority analysis) and time in remission over 2 years (non‐inferiority analysis, non‐inferiority margin 35 days). Analyses were done on an intention‐to‐treat basis. This study is registered with ClinicalTrials.gov, NCT02177071, and with EU Clinical Trials Register, EUDRACT 2014‐002311‐41. The trial was completed in April, 2021. FINDINGS: Between Nov 2, 2015, and April 24, 2019, 254 patients were screened. Of these, 211 were randomised and 207 were included in the final analysis (n=67 in the combination group, n=71 in the infliximab withdrawal group, and n=69 in the immunosuppressant withdrawal group). 39 patients had a relapse (eight [12%] of 67 in the combination group, 25 [35%] of 71 in the infliximab withdrawal group, six [9%] of 69 in the immunosuppressant withdrawal group). 2‐year relapse rates were 14% (95% CI 4‐23) in the combination group, 36% (24‐47) in the infliximab withdrawal group, and 10% (2‐18) in the immunosuppressant withdrawal group (hazard ratio [HR] 3·45 [95% CI 1·56‐7·69], p=0·003, for infliximab withdrawal vs combination, and 4·76 [1·92‐11·11], p=0·0004, for infliximab withdrawal vs immunosuppressant withdrawal). Of 28 patients who had a relapse and were retreated or optimised according to protocol, remission was achieved in 25 patients (one of two in the combination group, 22 of 23 in the infliximab withdrawal group, and two of three in the immunosuppressant withdrawal group). The mean time spent in remission over 2 years was 698 days (95% CI 668‐727) in the combination group, 684 days (651‐717) in the infliximab withdrawal group, and 706 days (682‐730) in the immunosuppressant withdrawal group. The difference in restricted mean survival time in remission was ‐14 days (95% CI ‐56 to 27) between the infliximab withdrawal group and the combination group and ‐22 days (‐62 to 16) between the infliximab withdrawal group and the immunosuppressant withdrawal group. The 95% CIs contained the non‐inferiority threshold (‐35 days). We recorded 31 serious adverse events, in 20 patients, with no difference in frequency between groups. The most frequent serious adverse events were infections (four in the combination group, two in the infliximab withdrawal group, and one in the immunosuppressant withdrawal group) and Crohn's disease exacerbation (three in the combination group, four in the infliximab withdrawal group, and one in the immunosuppressant withdrawal group). No death nor malignancy was recorded. INTERPRETATION: In patients with Crohn's disease in sustained steroid‐free remission under combination therapy with infliximab and immunosuppressant therapy, withdrawal of infliximab should only be considered after careful assessment of risks and benefits for each patient, whereas withdrawal of immunosuppressant therapy could generally represent a preferable strategy when considering treatment de‐escalation. FUNDING: European Union's Horizon 2020.

%Z The lancet. Gastroenterology & hepatology

Journal article

%U https://cochrane.66557.net/central/doi/10.1002/central/CN-02516113/full

%0 Journal Article

%A Holt, D. Q.

%A Moore, G. T.

%A Strauss, B. J.

%A Hamilton, A. L.

%A De Cruz, P.

%A Kamm, M. A.

%D 2017

%T Visceral adiposity predicts post-operative Crohn's disease recurrence

%V 45

%N 9

%P 1255‐1264

%8 2017-01-01

%R 10.1111/apt.14018

%K Adiposity; Adult; Biomarkers [metabolism]; Colonoscopy; Crohn Disease [*metabolism, surgery]; Feces [chemistry]; Female; Humans; Intra‐Abdominal Fat [*metabolism]; Leukocyte L1 Antigen Complex [metabolism]; Male; Middle Aged; Postoperative Period; Recurrence; Risk Factors; Young Adult

%X BACKGROUND: Excessive visceral adipose tissue has been associated with poorer outcomes in patients with inflammatory bowel disease. AIM: To determine whether body composition is associated with outcome in a prospective study of post‐operative Crohn's disease patients. METHODS: The POCER study evaluated management strategies for prevention of post‐operative Crohn's disease recurrence; subjects were enrolled after resection of all macroscopic Crohn's disease and were randomised to early endoscopy and possible treatment escalation, or standard care. The primary endpoint was endoscopic recurrence at 18 months. 44 subjects with cross‐sectional abdominal imaging were studied, and body composition analysis performed using established techniques to measure visceral adipose tissue area, subcutaneous adipose tissue area, and skeletal muscle area. RESULTS: The body composition parameter with the greatest variance was visceral adipose tissue. Regardless of treatment, all subjects with visceral adipose tissue/height2 >1.5 times the gender‐specific mean experienced endoscopic recurrence at 18 months (compared to 47%) [relative risk 2.1, 95% CI 1.5‐3.0, P = 0.012]. Waist circumference correlated strongly with visceral adipose tissue area (ρ = 0.840, P < 0.001). Low skeletal muscle was prevalent (41% of patients), but did not predict endoscopic recurrence; however, appendicular skeletal muscle indices correlated inversely with faecal calprotectin (ρ = 0.560, P = 0.046). CONCLUSIONS: Visceral adiposity is an independent risk factor for endoscopic recurrence of Crohn's disease after surgery. Sarcopenia correlates with inflammatory biomarkers. Measures of visceral adipose tissue may help to stratify risk in post‐operative management strategies.

%Z Alimentary pharmacology & therapeutics

Journal article

%U https://cochrane.66557.net/central/doi/10.1002/central/CN-01401223/full

%0 Journal Article

%A Holt, D. Q.

%A Moore, G. T.

%A Strauss, BJG

%A Hamilton, A. L.

%A De Cruz, P.

%A Kamm, M. A.

%D 2017

%T Visceral adiposity predicts post-operative Crohn's disease recurrence

%V (no pagination)

%8 2017-01-01

%R 10.1111/apt.14018

%K *Crohn disease; *intraperitoneal fat; *recurrent disease; Body composition; Calgranulin; Clinical article; Controlled clinical trial; Controlled study; Endogenous compound; Endoscopy; Female; Gender; Height; Human; Male; Postoperative care; Prospective study; Randomized controlled trial; Relapse; Risk factor; Sarcopenia; Skeletal muscle; Subcutaneous fat; Surgery; Treatment outcome; Waist circumference

%X Background: Excessive visceral adipose tissue has been associated with poorer outcomes in patients with inflammatory bowel disease. Aim: To determine whether body composition is associated with outcome in a prospective study of post‐operative Crohn's disease patients. Methods: The POCER study evaluated management strategies for prevention of post‐operative Crohn's disease recurrence; subjects were enrolled after resection of all macroscopic Crohn's disease and were randomised to early endoscopy and possible treatment escalation, or standard care. The primary endpoint was endoscopic recurrence at 18 months. 44 subjects with cross‐sectional abdominal imaging were studied, and body composition analysis performed using established techniques to measure visceral adipose tissue area, subcutaneous adipose tissue area, and skeletal muscle area. Results: The body composition parameter with the greatest variance was visceral adipose tissue. Regardless of treatment, all subjects with visceral adipose tissue/height2 >1.5 times the gender‐specific mean experienced endoscopic recurrence at 18 months (compared to 47%) [relative risk 2.1, 95% CI 1.5‐3.0, P = 0.012]. Waist circumference correlated strongly with visceral adipose tissue area (rho = 0.840, P < 0.001). Low skeletal muscle was prevalent (41% of patients), but did not predict endoscopic recurrence; however, appendicular skeletal muscle indices correlated inversely with faecal calprotectin (rho = 0.560, P = 0.046). Conclusions: Visceral adiposity is an independent risk factor for endoscopic recurrence of Crohn's disease after surgery. Sarcopenia correlates with inflammatory biomarkers. Measures of visceral adipose tissue may help to stratify risk in post‐operative management strategies. Copyright © 2017 John Wiley & Sons Ltd.

%Z Alimentary pharmacology & therapeutics

Journal article

%U https://cochrane.66557.net/central/doi/10.1002/central/CN-01341629/full

%0 Journal Article

%A Liang, C.

%A Chen, P.

%A Tang, Y.

%A Zhang, C.

%A Lei, N.

%A Luo, Y.

%A Duan, S.

%A Zhang, Y.

%D 2022

%T VENLAFAXINE AS AN ADJUVANT THERAPY FOR INFLAMMATORY BOWEL DISEASE PATIENTS WITH ANXIOUS AND DEPRESSIVE SYMPTOMS: a RANDOMIZED CONTROLLED TRIAL

%V 10

%P 706

%8 2022-01-01

%R 10.1002/ueg2.12295

%K *depression; Adult; Anxiety; Blood examination; Blood level; Clinical article; Clinical trial; Conference abstract; Controlled study; Crohn Disease Activity Index; Double blind procedure; Drug therapy; Female; Follow up; Gene expression; Hospital Anxiety and Depression Scale; Human; Human tissue; Inflammatory bowel disease; Male; Mental health; Prospective study; Protein expression; Protein function; Quality of life; Questionnaire; Randomized controlled trial; Recurrence risk; Univariate analysis

%X Introduction: The effect of antidepressant therapy on Inflammatory Bowel Disease (IBD) remains controversial. This trial aimed to assess whether adding venlafaxine to standard therapy for IBD improved the quality of life (QoL), mental health and disease activity in IBD patients with anxious and depressive symptoms. Aims & Methods: A prospective, randomized, double‐blind, placebocontrolled clinical trial was conducted. Participants diagnosed with IBD with symptoms of anxiety/depression were randomly assigned to receive either venlafaxine 150 mg daily or equivalent placebo and followed for 6 months. Inflammatory Bowel Disease Questionnaire (IBDQ), Mayo score, Crohn's disease activity index (CDAI), Hospital Anxiety and Depression Scale (HADS) and blood examination were completed before the enrollment, during and after the follow‐up. Mixed linear models and univariate analyses were used to compare groups. Results: Forty‐five IBD patients were included, of whom 25 were randomized to receive venlafaxine. The mean age was 39.76 (SD=11.30) years old. 25 (55.6%) was male. Venlafaxine showed a significant improvement on QoL (p<0.001) and disease course (p=0.035), a greater reduction in HADS (anxiety: p<0.001, depression: p<0.001), Mayo scores (p<0.001) and CDAI (p=0.006) 6 months. Venlafaxine had no effect on IL‐10 expression, endoscopic scores, relapse rate and use rate of biologics and corticosteroids, but did reduce serum level of ESR (p=0.003), CRP (p<0.001) and TNF‐α (0.009). Conclusion: Venlafaxine has a significantly beneficial effect on QoL, IBD activity and mental health in IBD patients with comorbid anxious or depressive symptoms.

%Z United European gastroenterology journal

Journal article; Conference proceeding

%U https://cochrane.66557.net/central/doi/10.1002/central/CN-02496513/full

%0 Journal Article

%A Liang, C.

%A Chen, P.

%A Tang, Y.

%A Zhang, C.

%A Lei, N.

%A Luo, Y.

%A Duan, S.

%A Zhang, Y.

%D 2022

%T Venlafaxine as an Adjuvant Therapy for Inflammatory Bowel Disease Patients With Anxious and Depressive Symptoms: a Randomized Controlled Trial

%V 13

%P 880058

%8 2022-01-01

%R 10.3389/fpsyt.2022.880058

%K *adjuvant chemotherapy; *anxiety; *cancer patient; *depression; *inflammatory bowel disease; *quality of life; Adult; Article; Blood examination; Cancer recurrence; Clinical article; Clinical trial; Clinical trial registry; Controlled study; Crohn Disease Activity Index; Double blind procedure; Drug therapy; Erythrocyte; Female; Follow up; Gene expression; Hospital Anxiety and Depression Scale; Human; Human cell; Human tissue; Inflammatory Bowel Disease Questionnaire; Male; Mayo score; Mental health; Prospective study; Protein expression; Protein function; Questionnaire; Randomized controlled trial; Recurrence risk; Univariate analysis; adjuvant therapy; adult; age; anxiety disorder; article; biological therapy; blood examination; clinical article; controlled study; corticosteroid therapy; depression; digestive system disease assessment; disease activity; disease course; double blind procedure; erythrocyte sedimentation rate; female; follow up; gastrointestinal endoscopy; gender; human; inflammatory bowel disease [drug therapy]; male; mental health; prospective study; protein blood level; protein expression; quality of life; randomized controlled trial; relapse; treatment duration

%X Background and Aims: The effect of antidepressant therapy on Inflammatory Bowel Disease (IBD) remains controversial. This trial aimed to assess whether adding venlafaxine to standard therapy for IBD improved the quality of life (QoL), mental health, and disease activity of patients with IBD with anxious and depressive symptoms. Methods: A prospective, randomized, double‐blind, and placebo‐controlled clinical trial was conducted. Participants diagnosed with IBD with symptoms of anxiety or depression were randomly assigned to receive either venlafaxine 150 mg daily or equivalent placebo and followed for 6 months. Inflammatory Bowel Disease Questionnaire (IBDQ), Mayo score, Crohn's disease activity index (CDAI), Hospital Anxiety and Depression Scale (HADS), and blood examination were completed before the enrollment, during, and after the follow‐up. Mixed linear models and univariate analyses were used to compare groups. Results: Forty‐five patients with IBD were included, of whom 25 were randomized to receive venlafaxine. The mean age was 40.00 (SD = 13.12) years old and 25 (55.6%) were male. Venlafaxine showed a significant improvement on QoL (p < 0.001) and disease course (p = 0.035), a greater reduction in HADS (anxiety: p < 0.001, depression: p < 0.001), Mayo scores (p < 0.001), and CDAI (p = 0.006) after 6 months. Venlafaxine had no effect on IL‐10 expression, endoscopic scores, relapse rate, and use rate of biologics and corticosteroids, but did reduce serum level of erythrocyte estimation rate (ESR; p = 0.003), C‐reactive protein (CRP; p < 0.001) and tumor necrosis factor‐α (TNF‐α; p = 0.009). Conclusions: Venlafaxine has a significantly beneficial effect on QoL, IBD activity, and mental health in patients with IBD with comorbid anxious or depressive symptoms. (Chinese Clinical Trial Registry, ID: ChiCTR1900021496).

%Z Frontiers in psychiatry

Journal article

%U https://cochrane.66557.net/central/doi/10.1002/central/CN-02414372/full

%0 Journal Article

%A Stevens, B. W.

%A Borren, N. Z.

%A Velonias, G.

%A Conway, G.

%A Cleland, T.

%A Andrews, E.

%A Khalili, H.

%A Garber, J. G.

%A Xavier, R. J.

%A Yajnik, V.

%A Et, Al.

%D 2017

%T Vedolizumab Therapy Is Associated with an Improvement in Sleep Quality and Mood in Inflammatory Bowel Diseases

%V 62

%N 1

%P 197‐206

%8 2017-01-01

%R 10.1007/s10620-016-4356-2

%K *Crohn disease/dm [Disease Management]; *Crohn disease/dt [Drug Therapy]; *anxiety; *general condition improvement; *inflammatory bowel disease; *mood; *sleep quality; *ulcerative colitis/dm [Disease Management]; *ulcerative colitis/dt [Drug Therapy]; *vedolizumab; *vedolizumab/cm [Drug Comparison]; *vedolizumab/dt [Drug Therapy]; Adalimumab/cm [Drug Comparison]; Adalimumab/dt [Drug Therapy]; Adult; Anxiety; Article; Biological therapy; C reactive protein/ec [Endogenous Compound]; Clinical trial; Controlled clinical trial; Controlled study; Crohn disease/dt [Drug Therapy]; Depression; Disease activity; Drug therapy; Endogenous compound; Erythrocyte sedimentation rate; Female; Golimumab/cm [Drug Comparison]; Golimumab/dt [Drug Therapy]; Human; Immunomodulating agent; Infliximab/cm [Drug Comparison]; Infliximab/dt [Drug Therapy]; Major clinical study; Male; Mesalazine; Nih promis questionnaire; Opiate; Priority journal; Prospective study; Protein blood level; Quality of life; Questionnaire; Statistical model; Steroid; Treatment duration; Tumor necrosis factor; Ulcerative colitis/dt [Drug Therapy]

%X Introduction: Poor sleep, depression, and anxiety are common in patients with inflammatory bowel diseases (IBD) and associated with increased risk of relapse and poor outcomes. The effectiveness of therapies in improving such psychosocial outcomes is unclear but is an important question to examine with increasing selectivity of therapeutic agents. Methods: This prospective cohort enrolled patients with moderate‐to‐severe CD or UC starting biologic therapy with vedolizumab or anti‐tumor necrosis factor alpha agents (anti‐TNF). Sleep quality, depression, and anxiety were measured using validated short‐form NIH PROMIS questionnaires assessing sleep and mood quality over the past 7 days. Disease activity was assessed using validated indices. Improvement in sleep and mood scores from baseline was assessed, and regression models were used to identify determinants of sleep quality. Results: Our study included 160 patients with IBD (49 anti‐TNF, 111 Vedolizumab) among whom half were women and the mean age was 40.2 years. In the combined cohort, we observed a statistically significant and meaningful decrease in mean scores from baseline (52.8) by week 6 (49.8, p = 0.002). Among vedolizumab users, sleep T‐score improved from baseline (53.6) by week 6 (50.7) and persisted through week 54 (46.5, p = 0.009). Parallel reductions in depression and anxiety were also noted (p < 0.05 by week 6). We observed no difference in improvement in sleep, depression, and anxiety between vedolizumab and anti‐TNF use at week 6. Conclusions: Both vedolizumab and anti‐TNF biologic therapies were associated with improvement in sleep and mood quality in IBD. Copyright © 2016, Springer Science+Business Media New York.

%Z Digestive diseases and sciences

Journal article

%U https://cochrane.66557.net/central/doi/10.1002/central/CN-01296635/full

%0 Journal Article

%A Hassan, H. A.

%A Obeid, L. M.

%A Aldahash, AAM

%D 2022

%T Value Assessment of Local Treatment (Diltiazem Hydrochloride gel/vs Glycerile Trinitrite ointment) in acute Anal Fissure

%V 12

%N 3

%P 1093‐1096

%8 2022-01-01

%R 10.25258/ijddt.12.3.28

%K *anus fissure; *local therapy; *ointment; Adult; Article; Clinical trial; Comparative effectiveness; Controlled study; Female; Follow up; Human; Major clinical study; Male; Pain; Prospective study; Randomized controlled trial; Recurrence risk; Side effect; Teaching hospital; Topical drug administration; adult; anus cancer; anus fissure; anus fistula; article; bleeding; cardiovascular disease; diabetes mellitus; double blind procedure; female; follow up; headache; hemorrhoidectomy; human; inflammatory bowel disease; information processing; major clinical study; male; migraine; ointment; pain; physical examination; prospective study; pruritus; randomized controlled trial; recurrence risk; side effect; topical drug administration; tuberculosis; ulcer; visual analog scale

%X Background: Anal fissure is a linear tear in the distal anal canal, associated with spasm of internal anal sphincter. Acute anal pain with bleeding on defecation are the main symptoms. Different types of chemical treatment to relax the sphincteric muscles have tried. Objective: Assess the efficacy, side effects and recurrence rate of 0.2% glycerile trinitrate ointment (GTN) and 2% diltiazim hydrochloride gel (DTZ) in treatment of acute anal fissure. Methods: A prospective randomized trial of 112 patient with acute anal fissure over 1.5 years period at Al‐Kindy Teaching Hospital. The diagnosis was entirely clinical. Setting of inclusion and exclusion criteria, ethical consideration respected. Two groups of patients submitted randomly to either 0.2% GTN ointment or 2% DTZ gel twice daily for 8 weeks. Correct pain score by the patient every day, follow up to the end of week 1, 2, 8 and at 6 months. Scientific signs of healing assessed blinded to the mode of treatment. The collected data was analyzed and compared. Results: Complete fissure healing was observed in (83.3%) of the DTZ group and (76%) of the GTN group. Pain response was better in DTZ than GTN patients were. Side effects had reported. The recurrence rate was (15%) in DTZ group and (30%) in GTN after 6 months follow up. Conclusion: Topical Diltiazem is greater than Glyceryl trinitrate in managing acute anal fissure with low side effects and reappearance rate.

%Z International journal of drug delivery technology

Journal article

%U https://cochrane.66557.net/central/doi/10.1002/central/CN-02486918/full

%0 Journal Article

%A DeFor, T. E.

%A Le, C.

%A Smith, A. R.

%A Warlick, E. D.

%A Bejanyan, N.

%A Weisdorf, D. J.

%D 2016

%T Validation of a modified comorbidity index for allogeneic hematopoietic cell transplant

%V 128

%N 22

%8 2016-01-01

%K *comorbidity; *hematopoietic cell; *validation process; Adult; Bootstrapping; Calculation; Cancer susceptibility; Cerebrovascular disease; Clinical trial; Collagen disease; Congenital malformation; Controlled clinical trial; Controlled study; Decision making; Diabetes mellitus; Diagnosis; Female; Graft recipient; Hazard ratio; Human; Infection; Inflammatory bowel disease; Kidney failure; Liver function; Lung; Major clinical study; Male; Mental disease; Middle aged; Mortality; Multicenter study; Obesity; Overall survival; Peptic ulcer; Prediction; Regression analysis; Risk factor; Sibling; Solid tumor; Statistical model; Statistics; Umbilical cord blood; Unrelated donors; Valvular heart disease; adult; bootstrapping; calculation; cancer susceptibility; cerebrovascular disease; clinical trial; collagen disease; comorbidity; congenital malformation; controlled clinical trial; controlled study; decision making; diabetes mellitus; diagnosis; female; graft recipient; hazard ratio; hematopoietic cell; human; infection; inflammatory bowel disease; kidney failure; liver function; lung; major clinical study; male; mental disease; middle aged; mortality; multicenter study; obesity; overall survival; peptic ulcer; prediction; regression analysis; risk factor; sibling; solid malignant neoplasm; statistical model; statistics; umbilical cord blood; unrelated donor; validation process; valvular heart disease

%X INTRODUCTION Among adult allogeneic hematopoietic cell transplant (HCT) recipients, the HCT‐specific comorbidity index (HCT‐CI) is a standard measure of baseline comorbidity. This measure incorporates 17 different comorbidities into a combined, categorically weighted score of standard, intermediate and high risk. Using the specific weights for each comorbidity from the single center analysis, the HCT‐CI has been validated in other studies, most notably in a recent analysis including 8115 HCT recipients from the United States. The HCT‐CI has been useful in controlling for confounding of comorbidities among patients. We previously reported that the efficiency and predictive power could be improved by removing the conversion of adjusted hazard ratios (HR) for non‐relapse mortality (NRM) to three possible weights (1‐3) for each comorbidity. METHODS Because some comorbidities show effects on a continuous scale and others show no effect, we proposed a weighting scheme in which each comorbidity is assigned the natural weight based on Fine and Gray regression analysis on NRM. The final modified comorbidity index (MCI) is based on a multiplicative model controlling for age, disease risk index, donor type and stratified by conditioning intensity. In this current study, we tested validation of calculations for the MCI by randomizing 2/3 of 1114 adult allogeneic patients with prospectively collected (2000‐2015) comorbidities to a training set and 1/3 of patients to a test set. Using weights from the training set, we compared the MCI to the HCT‐CI for the endpoints of NRM and overall survival (OS) in the test set. We did this using regression analysis and bootstrapping the difference in C‐statistics for each method. RESULTS The median patient age was 51 (IQR: 39‐59), 59% were male, donors included 41% HLA‐matched sibling donors, 7% matched unrelated donors (URD) and 52% umbilical cord blood (UCB). Patients had malignant diagnoses with a disease risk index (DRI) of 19% low, 62% intermediate and 19% high or very high. Conditioning intensity included 65% reduced intensity (RIC) regimens. Using the HCT‐CI, 19% were classified as low, 31% as intermediate and 39% as high risk. Based on the MCI, 34% were classified as low, 54% as intermediate and 12% as high risk. After adjusting for other factors, the independent weights for each comorbidity were calculated in our training set. We calculated the MCI by exponentiating the sum of all parameter coefficients from the regression analysis. The revised index score is: MCI = exponent [0.40∗ (binary indicator for cardiac disorders) + 0.85∗(heart valve disease) + 0.05∗(inflammatory bowel disease) + 0.48∗(peptic ulcer) + 0.46∗ (diabetes) + 0.03∗(psychiatric disturbance) + 0.20∗(mild hepatic function) + 0.93∗(moderate/severe hepatic function) + 0.19∗(infection) + 2.00∗(renal insufficiency) + 0.17∗(moderate pulmonary abnormalities) + 0.39∗(severe pulmonary abnormalities) + 0.16∗(prior solid tumor)]. Comorbidities including obesity, cerebrovascular disease and rheumatologic disorders had no influence on NRM. This on‐line calculator facilitates scoring of the modified index‐‐MCI: http://bmt.ahc.umn.edu:8082/hct. In the test set (N=372), MCI was more predictive of NRM (table, fig 1a and 1b) and showed a trend toward increased sensitivity for OS compared to the original HCT‐CI. The HR for intermediate and high risk categories increased (≥60% for NRM and >30% for OS). The adjusted likelihood ratio (showing model fit) increased from 20.3 to 22.5 for NRM and from 38.9 to 40.7 for OS when substituting MCI for HCT‐CI. An increase shows better prediction of the endpoint. The C‐statistic reflecting more NRM with a higher score and worse survival increased from 0.540 to 0.562 for NRM (P=0.02) and increased from 0.567 to 0.594 for OS (P=0.08). DISCUSSION This new MCI showed higher discriminating and predictive power for post‐HCT NRM and a trend towards more predictive power for OS. As many HCT recipients have pre‐existing comorbidities, the greater discrimination in assigning patient comorbidity will better inform decision‐making for HCT recipients and HCT studies by better adjustment of these important risk factors. This MCI methodology should be used to create more efficient and predictive assessments in a larger multi‐center study. (Figure presented).

%Z Blood

Journal article; Conference proceeding

%U https://cochrane.66557.net/central/doi/10.1002/central/CN-01302968/full

%0 Journal Article

%A NCT

%D 2019

%T Vagal Nerve Stimulation for Intestinal Barrier Dysfunction in Healthy Volunteers

%8 2019-01-01

%X Stress ‐ a state which evokes a 'fight or flight' or sympathetic response from the human organism ‐ has been implicated in the propagation of inflammation in patients with inflammatory bowel disease (IBD). Indeed, the risk of relapse in both Crohns disease (CD) and Ulcerative colitis (UC) increases in the presence of both acute and chronic stress. The mechanism proposed for this phenomenon includes the ability of stress to induce a breakdown in intestinal barrier function via mast cell‐ and corticotropin releasing hormone (CRH)‐dependent pathways. This intestinal barrier perturbation has been demonstrated experimentally in humans using stress paradigms which could be equated to the relatively mild stressful events experienced in every‐day life. The autonomic nervous system (ANS) appears to be playing a crucial role in stress‐induced changes in intestinal permeability. Multiple in vitro and in vivo murine model studies have confirmed that both acute and chronic stress (induced in a mouse by various stress paradigms such as restraint and water emersion) can promote increased intestinal permeability. The underlying mechanism seems to be via the disruption of tight junction proteins along the paracellular space of the intestinal epithelium. The disruption of tight junctions in these stress paradigm models are dependent on acetylcholine and CRH. This effect on intestinal permeability appears to recover within 4 days in murine models. Similar changes have been described in human models of acute stress. Barclay and Turnberg first showed that intestinal absorption of salt and water was reduced in healthy participants in response to acute stress induced by a short‐term stressor paradigm. In this study, they used dichotomous listening as the physiological stressor, whereby the participant is asked to listen to two types of different genres of music in each ear whilst they completed a mentally challenging task. These physiological absorption changes to the intestine's barrier function were found to be mediated by a cholinergic‐dependent mechanism. The same authors later showed similar effects with the physical stress paradigm of cold‐induced pain in healthy volunteers. Although actual intestinal permeability changes were not measured at the time, one likely mechanism of permeability changes related to these alterations in electrolyte flux is via the claudin‐2 paracellular channel, which has since been described. The configuration of this channel is related to ionic flux across the intestinal epithelium and, therefore, would render the epithelium more permeable in times of electrolyte flux. In more recent studies utilising the same cold‐pain paradigm, changes in intestinal permeability were paralleled by the release of products of mast cell degranulation, tryptase and histamine. Such changes in intestinal permeability induced by acute stress were mimicked by peripheral administration of CRH and could be reversed by the administration of mast cell stabilisers. The therapeutic effect of vagal nerve stimulation in intestinal barrier dysfunction is an important area of study as it could lead to a novel and in‐expensive method of treatment of conditions associated with intestinal barrier perturbation. In humans, the auricular branch of the vagus nerve is located directly under the skin, making it a suitable target for transcutaneous stimulation. A transcutaneous electrical nerve stimulation (TENS) device is thus a suitable non‐invasive tool for the investigation of transcutaneous vagal nerve stimulation (tVNS) for reduction of intestinal permeability in humans and has been used in a number of studies utilizing the technique of tVNS in our laboratory. A key role for the parasympathetic nervous system in the perturbation of intestinal permeability by severe physiological stress was derived from the effects of vagal nerve stimulation in animal models. Stimulation of the cervical vagal nerve for 10 minutes prior to giving mice a severe burn protected them from intestinal barrier perturbation and increased intestinal glial fibrillary acidic protein (FGAP) expression, a marker of enteric nervous system glial activation, in vitro and in vivo.This effect was likely, at least in part, due to an α7 acetylcholine receptor dependent mechanism that was demonstrated in both cultured intestinal epithelial cells as well as in enteric glial cells. Further studies have since supported this protective effect of vagal nerve stimulation, suggesting a role for the preservation of the number of dendritic cells in the mesenteric lymph as another potential mechanism.Moreover, the protective effect on the epithelial lining seemed to be via a locally‐induced mechanism on the intestine rather than being dependent on a splenic‐mediated process as the protective effect held true in splenectomised mice. Stimulation of the parasympathetic nervous system via the vagal nerve therefore seems to play a protective role in modulation of the intestinal barrier function with experimental evidence hinting at its ability to reverse stress induced changes in intestinal permeability. These results are of significant interest given that the last decade has seen a number of studies supporting the theory of intestinal barrier perturbation in the pathogenesis of IBD.Additionally, the detection of antibodies directed against microbial antigens (such as 'ASCA' ‐ epitopes of Saccharomyces cerevisiae and Flagellin CBir1) are found in the serum of patients prior to the development of IBD ‐ indicating a disruption of the intestinal barrier function prior to the onset of disease. Furthermore, a genetic predisposition to intestinal barrier perturbation is considered to be a predisposing factor in the development of IBD. Given that it seems that the ANS has a significant role to play in the maintenance of the intestinal barrier function, it would be reasonable to conclude that alterations within the ANS in a susceptible individual may, at least in part, be implicated the development and flares of IBD. Corticotropin releasing hormone (CRH) is naturally secreted from the healthy human hypothalamus during periods of stress. Intravenously administered CRH possesses the advantage of having a short half‐life and is routinely clinically utilised in the diagnosis of Cushing's syndrome. Recent evidence has shown that injecting CRH intravenously reliably and significantly increases intestinal permeability in healthy volunteers with no significant adverse effects.This model will be used to induce increased intestinal permeability in healthy human volunteers in order to test the hypothesis that pre‐treatment with vagal nerve stimulation can reduce the degree of intestinal permeability induced by CRH. Several serological and urinary markers have been validated as useful tools in measuring intestinal permeability changes in vivo. These include lipopolysaccharide‐binding protein (LBP), CD‐14 and intestinal‐type fatty acid‐binding protein (I‐FABP) in the serum, as well as an orally‐administered dual sugar solution and its by‐products in serum and urine. Soluble CD‐14 and LBP are part of the LPS inflammatory signalling pathway, thus their levels in serum have been shown to be objective markers for systemic immune activation and damage to gut epithelium. I‐FABP is an intracellular protein expressed in the epithelial cells of the mucosal layer of the small and large intestine tissue and is prone to leakage into the blood stream from the enterocytes when intestinal mucosal damage occurs. The oral dual sugar test uses lactulose and rhamnose that are differentially absorbed in the intestine and excreted in the urine. The ratio of lactulose to rhamnose in the urine and serum can be used as a measure of small intestinal permeability when the urine is collected for two hours post ingestion of the test solution.

%Z https://clinicaltrials.gov/show/NCT04061564

Trial registry record

%U https://cochrane.66557.net/central/doi/10.1002/central/CN-01983569/full

%0 Journal Article

%A Buisson, A.

%A Nancey, S.

%A Manlay, L.

%A Rubin, D. T.

%A Hebuterne, X.

%A Pariente, B.

%A Fumery, M.

%A Laharie, D.

%A Roblin, X.

%A Bommelaer, G.

%A Et, Al.

%D 2021

%T Ustekinumab is more effective than azathioprine to prevent endoscopic postoperative recurrence in Crohn's disease

%V 9

%N 5

%P 552‐560

%8 2021-01-01

%R 10.1002/ueg2.12068

%K *Crohn disease; Adult; Antibodies, Monoclonal, Humanized [administration & dosage, therapeutic use]; Article; Azathioprine [administration & dosage, *therapeutic use]; Clinical article; Clinical trial; Cohort analysis; Comparative effectiveness; Control Groups; Controlled study; Crohn Disease [*prevention & control, surgery]; Drug combination; Drug therapy; Female; Gastrointestinal Agents [administration & dosage, therapeutic use]; Human; Humans; Immunosuppressive Agents [administration & dosage, *therapeutic use]; Intestine resection; Male; Multicenter study; Phenotype; Prevention; Probability; Propensity Score; Propensity score; Randomized controlled trial; Recurrence; Retrospective Studies; Retrospective study; Risk factor; Secondary Prevention [*methods]; Smoking; Surgery; Ustekinumab [administration & dosage, *therapeutic use]

%X Background: Preventing postoperative recurrence (POR) is a major concern in Crohn's disease (CD). While azathioprine is an option, no data is available on ustekinumab efficacy in this situation. Aims: We compared the effectiveness of ustekinumab versus azathioprine in preventing endoscopic POR in CD. Methods: We retrospectively collected data from all consecutive CD patients treated with ustekinumab after intestinal resection in 9 centers. The control group (azathioprine alone) was composed of patients who participated in a randomized controlled trial conducted in the same centers comparing azathioprine alone or in combination with curcumin. Propensity score analyses (inversed probability of treatment weighting = IPTW) were applied to compare the two groups. The primary endpoint was endoscopic POR (Rutgeerts' index ≥ i2) at 6 months. Results: Overall, 32 patients were included in the ustekinumab group and 31 in the azathioprine group. The propensity score analysis was adjusted on the main risk factors (smoking, fistulizing phenotype, prior bowel resection, resection length >30 cm and ≥2 biologics before surgery) and thiopurines or ustekinumab exposure prior to surgery making the two arms comparable (∣d∣ < 0.2). After IPTW, the rate of endoscopic POR at 6 months was lower in patients treated with ustekinumab compared to azathioprine (28.0% vs. 54.5%, p = 0.029). After IPTW, the rates of i2b‐endoscopic POR (Rutgeerts' index ≥ i2b) and severe endoscopic POR (Rutgeerts' index ≥ i3) were 20.8% versus 42.5% (p = 0.066) and 16.9% versus 27.9% (p = 0.24), in the ustekinumab and azathioprine groups, respectively. Conclusion: Ustekinumab seemed to be more effective than azathioprine in preventing POR in this cohort of CD patients.

%Z United European gastroenterology journal

Journal article

%U https://cochrane.66557.net/central/doi/10.1002/central/CN-02275682/full

%0 Journal Article

%A Buisson, A.

%A Nancey, S.

%A Manlay, L.

%A Rubin, D. T.

%A Hebuterne, X.

%A Pariente, B.

%A Fumery, M.

%A Laharie, D.

%A Roblin, X.

%A Bommelaer, G.

%A Et, Al.

%D 2020

%T Ustekinumab is more effective than azathioprine to prevent endoscopic postoperative recurrence in Crohn's disease

%V 8

%N 8 SUPPL

%P 84‐85

%8 2020-01-01

%R 10.1177/2050640620927344

%K *colon Crohn disease; Adult; Adverse drug reaction; Anastomosis; Clinical article; Clinical assessment; Clinical trial; Cohort analysis; Conference abstract; Controlled study; Crohn disease; Drug combination; Drug therapy; Female; Human; Ileocolonoscopy; Intestine resection; Male; Monotherapy; Multicenter study; Phenotype; Prevention; Probability; Randomized controlled trial; Retrospective study; Risk factor; Side effect; Smoking; Stoma; Surgery

%X Introduction: Preventing postoperative recurrence (POR) is a major concern in Crohn's disease (CD). While azathioprine is an option, no data is available on ustekinumab efficacy in this situation. Aims & Methods: We aimed to assess whether ustekinumab is more effective than azathioprine to prevent endoscopic POR in patients with CD. We retrospectively collected data from all consecutive CD patients treated with ustekinumab after intestinal resection in 9 centers. All these patients received intravenous induction with a median interval between restoration of bowel continuity and onset of ustekinumab of 30.5 days IQR [16.8‐ 49.8 days]. All the subsequent subcutaneous injections were performed using a dose of 90 mg. The control group (azathioprine 2.5 mg/kg/day monotherapy) was composed of patients who participated in a randomized controlled trial conducted in the same centers, comparing azathioprine alone or in combination with curcumin. Azathioprine was started 14 days after restoration of bowel continuity. We included patients with CD older than 18 years‐old who had undergone a surgical resection for ileal, ileocolonic, or colonic CD with ileocolonic anastomosis. All the macroscopic lesions had to be removed during the surgery. The anastomosis had to be reachable by ileocolonoscopy. The primary endpoint was endoscopic POR evaluated 6 months (M6) after intestinal resection or following stoma closure, defined as Rutgeerts' index ≥ i2. The secondary endpoints were alternative definitions of endoscopic POR using a Rutgeerts' index ≥ i2b as the cut‐off value (i2b‐endoscopic POR), and Rutgeerts' index ≥ i3 as the cut‐off value (severe endoscopic POR). Endoscopic procedures at M6 were scored by independent central readers in the control group. For the patients treated with ustekinumab, all endoscopic evaluations were performed by local readers who were experienced in assessing Rutgeerts' index in clinical trials. Propensity‐matched analyses (Inversed Probability of Treatment weighting = IPTW) were applied to make the two groups comparable. Results: Overall, 32 patients were included in the ustekinumab group and 31 in the azathioprine group. No patient received azathioprine in the ustekinumab group. The median level of 6‐TGN was 365 pmol at M3 and 260 pmol at M6. The propensity‐matched analysis was adjusted on the main risk factors (smoking, fistulizing phenotype, prior bowel resection, resection length > 30 cm and ≥ 2 biologics before surgery) but also on thiopurines or ustekinumab exposure prior to surgery making the two arms comparable (1/2d1/2< 0.2). In this cohort, 93.7 % (30/32) and 83.9% (26/31) of the patients had at least one of the main risk factors of POR in ustekinumab and azathioprine group, respectively. After IPTW, the rate of endoscopic postoperative recurrence at 6 months was lower in patients treated with ustekinumab compared to azathioprine (28.0% vs 54.5%, p =0.029). After IPTW, the rates of i2b‐endoscopic POR (Rutgeerts' index ≥ i2b) and severe endoscopic POR (Rutgeerts' index ≥ i3) were 20.8% vs 42.5% (p =0.066) and 16.9% vs 27.9% (p =0.24), in the ustekinumab and azathioprine groups, respectively. No adverse event was reported in the ustekinumab group while two serious adverse events occurred in the control group. Conclusion: In this cohort study, we found that ustekinumab was more effective than azathioprine to prevent endoscopic POR in patients with CD. Hence, ustekinumab could be an interesting option in this situation.

%Z United European gastroenterology journal

Journal article; Conference proceeding

%U https://cochrane.66557.net/central/doi/10.1002/central/CN-02242917/full

%0 Journal Article

%A Mege, D.

%A Panis, Y.

%D 2017

%T Unmet Therapeutic Needs: focus on Intestinal Fibrosis Surgical Approach: resection, Strictureplasty and Others

%V 35

%N 1‐2

%P 38‐44

%8 2017-01-01

%R 10.1159/000449081

%K *colon Crohn disease; *intestinal fibrosis; *intestine resection; *intestine stenosis; *intestine surgery; *strictureplasty; *surgical approach; Abdominal pain/co [Complication]; Adalimumab; Anastomosis; Article; Balloon dilatation; Bleeding/co [Complication]; Clinical study; Constriction, Pathologic; Controlled clinical trial; Controlled study; Digestive System Surgical Procedures [*methods]; Disease duration; Esthetic surgery; Exposure; Fibrosis; Human; Humans; Ileum; Immunosuppressive agent; Intestine resection; Intestine stenosis; Intestine, Small; Intestines [*pathology, *surgery]; Laparoscopic surgery; Metal stent; Morbidity; Mortality; Perforation/co [Complication]; Postoperative complication; Priority journal; Quality of life; Randomized controlled trial; Recurrence risk; Recurrent disease; Sample size; Short bowel syndrome; Side to side anastomosis; Stent; Surgeon; Surgery; Theoretical model; Treatment Outcome; Tumor necrosis factor inhibitor

%X BACKGROUND: In patients with ileo‐colonic Crohn's disease (CD), the main consequence of the development of intestinal fibrosis is the occurrence of a localized symptomatic stenosis for which treatment is mandatory. Besides medical treatment, which is still considered to be ineffective against non‐inflammatory fibrotic intestinal stenosis due to CD, there are 2 options for the treatment of such stenosis: endoscopic and surgical approaches. Key Messages: Endoscopic treatment includes balloon dilatation and stenting, and can be performed only on selected patients with very short stenosis. Few reports with a small sample size are available; long‐term results of endoscopic treatment remain unknown, with patients being exposed to possible early recurrence of the stenosis. For this reason, intestinal resection currently remains the first option for localized symptomatic intestinal stenosis due to CD refractory to medical therapy. Laparoscopic ileocecal resection with ileocolonic anastomosis gives good short‐term results, without mortality and with very low rate of morbidity. Furthermore, when the resection is shorter than 50 cm, very few functional consequences or no consequences are reported, and quality of life is improved. However, CD recurrence is frequent and can be required to redo surgery in up to 30% of the cases. In order to reduce the theoretical risk of short bowel syndrome, some surgeons have proposed strictureplasty as a more conservative approach. The concept is to treat stenosis without intestinal resection by opening the stenosis. There are different kinds of stricturoplasty, with similar reported morbidity and long‐term recurrence rates than those observed with resection. CONCLUSIONS: Because no randomized study exists, it is difficult to know what the best option for symptomatic ileal stenosis in CD is. However, for a majority of patients today, ileocecal resection is the first option, strictureplasty being reserved by most of the surgeons for recurrent cases and/or multiple stenoses. It requires more experience to perform endoscopic treatment with long‐term results.

%Z Digestive diseases (Basel, Switzerland)

Journal article

%U https://cochrane.66557.net/central/doi/10.1002/central/CN-01328227/full

%0 Journal Article

%A Fleshman, J.

%A Schoetz, D.

%A Phang, T.

%A MacDonald, H.

%A Koltun, W.

%A Vasilevsky, C.

%A Buie, W.

%A Hyman, N.

%A Wolff, B.

%A McLeod, R.

%A Et, Al.

%D 2009

%T Type of procedure does not affect postoperative recurrence rates following ileocolic resection

%V 52

%N 4

%P 827

%8 2009-01-01

%R 10.1007/DCR.0b013e31819e84d4

%K *recurrence risk; *society; *surgeon; *surgery; Abscess; Anastomosis; Colonoscopy; Crohn disease; Female; Fistula; Hospitalization; Male; Multivariate analysis; Patient; Randomized controlled trial; Risk

%X Purpose: to determine whether a laparoscopic approach to ileocolic resection affects post operative recurrence rates Methods: 170 patients who had an ileocolic resection for Crohn's disease as part of a multicentre randomized controlled trial assessing the effect of the anastomosis on recurrence rates were included in this study. All patients had a colonoscopy at one year. Endoscopic recurrence, defined as disease> i, 2 on a Modified Rutgeert's score, was the primary endpoint. Symptomatic recurrence was defined as symptoms plus endoscopic evidence of disease. Results: Ninety‐four (55.3%) patients (34 males:60 females; mean age 41.5 years) had an open and 76 (44.7%) patients (28 males:48 females, mean age 36.6 years) had a laparoscopic assisted procedure. The conversion rate was 30%. Those in the open group were more likely to have had previous resections (46% vs. 20%, p=0.0005), have an abscess or fistula (36% vs 28% p<0.05) and require additional procedures (20% vs. 9%, p=0.043). The mean duration of their operation was shorter (115 vs 139 mins, p=0.002) but their median hospital stay was longer (6 vs. 5 days, p=0.011). In total, 139 patients had a colonoscopy performed at mean 11.1 months post operatively. The endoscopic recurrence rate was 43.0% in the open compared to 35.5% in the laparoscopic group (p=0.65). The symptomatic recurrence rate was 20.3% in the open compared to 24.2% in the laparoscopic group (p=0.80). On multivariate analysis, procedure type was not predictive of recurrence. Conclusions: Laparoscopic ileocolic resection offers potential benefits to patients with Crohn's disease without affecting the risk of post operative recurrence.

%Z Diseases of the colon and rectum

Journal article; Conference proceeding

%U https://cochrane.66557.net/central/doi/10.1002/central/CN-01783545/full

%0 Journal Article

%A Regueiro, M.

%A Schraut, W. H.

%A Baidoo, L.

%A Kip, K.

%A Plevy, S. E.

%A El-Hachem, S.

%A Harrison, J.

%A Pesci, M.

%A Watson, A. R.

%A Binion, D. G.

%D 2009

%T Two year postoperative follow-up of patients enrolled in the randomized controlled trial (RCT) of infliximab (IFX) for prevention of recurrent Crohn's disease (CD)

%V 136

%N 5

%P A522

%8 2009-01-01

%R 10.1016/S0016-5085(09)62403-3

%K *Crohn disease; *follow up; *gastrointestinal disease; *patient; *prevention; *randomized controlled trial; Colonoscopy; Endoscopy; Infusion; Intestine resection; Recurrence risk; Remission; Surgery

%X Background: A single center RCT in CD pts with intestinal resection found that IFX was more effective than placebo in preventing 1 yr endoscopic, histologic, and clinical CD recurrence. Outcomes in pts reaching the 2 yr post‐operative time point are provided. Aims: To examine the CD endoscopic recurrence rates 2 yrs after intestinal resection. Methods: There were 24 pts in the original 1 yr trial: 13 randomized to placebo and 11 to IFX. At completion of trial, pts had a colonoscopy and were offered open‐label IFX. Another colonoscopy was performed 1 yr later. Endoscopic scoring was the same for follow‐up as in the trial; ileal (i) score of i0 or i1 = endoscopic remission, i2, i3, or i4 = endoscopic recurrence. Results: To date, 12 pts have had > 1 yr of follow‐up and colonoscopy after the original trial and > 2 yrs since surgery. (Table) Six had been on placebo and were administered IFX after completion of the trial; all had end of trial endoscopy scores between i2 and i4. All but 1 subsequently improved with IFX, however 3 (50%) had scores of i2. Six pts were originally on IFX, all but 1 ended the trial with i0. Subsequently, 4 opted for open‐label IFX and 2 for no treatment. The 2 who opted for no treatment had CD recurrence scores of i2 and i4 at post‐op yr 2. All but 1 of the 4 IFX pts who continued IFX had no change in their endoscopy scores. The primary IFX non‐responder (i3) did not improve despite continued IFX at yr 2. Conclusions: Anti‐TNF naïve pts who develop endoscopic CD recurrence 1 yr after resective surgery may be effectively treated with IFX. Endoscopic improvement appears to be attenuated in pts receiving IFX in response to recurrence rather than prophylactically. Pts treated with IFX after surgery maintain remission with ongoing infusions, but recur if IFX is stopped. Primary IFX post‐op non‐responders do not benefit from continued IFX. {Table presented}.

%Z Gastroenterology

Journal article; Conference proceeding

%U https://cochrane.66557.net/central/doi/10.1002/central/CN-01724804/full

%0 Journal Article

%A Dearnaley, D.

%A Griffin, C. L.

%A Lewis, R.

%A Mayles, P.

%A Mayles, H.

%A Naismith, O. F.

%A Harris, V.

%A Scrase, C. D.

%A Staffurth, J.

%A Syndikus, I.

%A Et, Al.

%D 2019

%T Toxicity and Patient-Reported Outcomes of a Phase 2 Randomized Trial of Prostate and Pelvic Lymph Node Versus Prostate only Radiotherapy in Advanced Localised Prostate Cancer (PIVOTAL)

%V 103

%N 3

%P 605‐617

%8 2019-01-01

%R 10.1016/j.ijrobp.2018.10.003

%K *advanced cancer /disease management /drug therapy /radiotherapy; *advanced localised prostate cancer /disease management /drug therapy /radiotherapy; *cancer radiotherapy; *intensity modulated radiation therapy; *patient‐reported outcome; *pelvis lymph node; *prostate cancer /disease management /drug therapy /radiotherapy; *radiation injury /complication; Acute toxicity; Aged; Antiandrogen therapy; Article; Biopsy; Bladder disease; Cancer hormone therapy; Cancer recurrence; Controlled study; Diarrhea; Follow up; Gastrointestinal toxicity; Glioblastoma; Heart infarction; High risk patient; Human; Humans; Image guided radiotherapy; International Prostate Symptom Score; Ischemic heart disease; Leukocyte count; Lung cancer; Lymph Nodes [*drug effects]; Lymphatic Irradiation [*methods]; Lymphatic Metastasis; Major clinical study; Male; Middle Aged; Multicenter study; Patient Reported Outcome Measures; Patient safety; Pelvis [radiation effects]; Phase 2 clinical trial; Planning target volume; Priority journal; Proctitis; Prostate [*radiation effects]; Prostatic Neoplasms [*radiotherapy]; Quality of life; Radiation dose distribution; Radiation dose fractionation; Radiation safety; Radiotherapy Dosage; Radiotherapy, Intensity‐Modulated [*methods]; Randomized controlled trial; Rectum hemorrhage; Respiratory tract disease; Scoring system; Seminal vesicle; Testosterone blood level; Treatment Outcome

%X Purpose: To establish the toxicity profile of high‐dose pelvic lymph node intensity‐modulated radiation therapy (IMRT) and to assess whether it is safely deliverable at multiple centers. Methods and Materials: In this phase 2 noncomparative multicenter trial, 124 patients with locally advanced, high‐risk prostate cancer were randomized between prostate‐only IMRT (PO) (74 Gy/37 fractions) and prostate and pelvic lymph node IMRT (P&P; 74 Gy/37 fractions to prostate, 60 Gy/37 fractions to pelvis). The primary endpoint was acute lower gastrointestinal (GI) Radiation Therapy Oncology Group (RTOG) toxicity at week 18, aiming to exclude a grade 2 or greater (G2+) toxicity‐free rate of 80% in the P&P group. Key secondary endpoints included patient‐reported outcomes and late toxicity. Results: One hundred twenty‐four participants were randomized (62 PO, 62 P&P) from May 2011 to March 2013. Median follow‐up was 37.6 months (interquartile range [IQR], 35.4‐38.9 months). Participants had a median age of 69 years (IQR, 64‐74 years) and median diagnostic prostate‐specific androgen level of 21.6 ng/mL (IQR, 11.8‐35.1 ng/mL). At week 18, G2+ lower GI toxicity‐free rates were 59 of 61 (96.7%; 90% confidence interval [CI], 90.0‐99.4) for the PO group and 59 of 62 (95.2%; 90% CI, 88.0‐98.7) for the P&P group. Patients in both groups reported similarly low Inflammatory Bowel Disease Questionnaire symptoms and Vaizey incontinence scores. The largest difference occurred at week 6 with 4 of 61 (7%) and 16 of 61 (26%) PO and P&P patients, respectively, experiencing G2+ toxicity. At 2 years, the cumulative proportion of RTOG G2+ GI toxicity was 16.9% (95% CI, 8.9%‐30.9%) for the PO group and 24.0% (95% CI, 8.4%‐57.9%) for the P&P group; in addition, RTOG G2+ bladder toxicity was 5.1% (95% CI, 1.7%‐14.9%) for the PO group and 5.6% (95% CI, 1.8%‐16.7%) for the P&P group. Conclusions: PIVOTAL demonstrated that high‐dose pelvic lymph node IMRT can be delivered at multiple centers with a modest side effect profile. Although safety data from the present study are encouraging, the impact of P&P IMRT on disease control remains to be established.

%Z International journal of radiation oncology, biology, physics

Journal article

%U https://cochrane.66557.net/central/doi/10.1002/central/CN-01789861/full

%0 Journal Article

%A NCT

%D 2018

%T Tolerability and Risk of Adverse Events With a Probiotic Supplement

%8 2018-01-01

%X The understanding of the role of the gastrointestinal microbes for human health has gained considerable interest in recent years. The butyrate‐producing bacterium Faecalibacterium prausnitzii is a naturally occurring bacterial species in the human gut that can make up to 5% of the gastrointestinal flora in healthy individuals. Several studies have shown that the presence of butyrate producing bacteria, including F. prausnitzii, is lower in patients with inflammatory bowel disease; Crohn's disease and ulcerative colitis. Furthermore, lower levels of short fatty acids have been found in people with ulcerative colitis as compared to healthy individuals. Similar results have been obtained from studies about Crohn's disease, where people with a low abundance of F. prausnitzii run a higher risk of post‐operative recurrence of their disease. It has become evident that bacteria in the human gastrointestinal tract are symbiotic and dependent on each other's metabolism. Studies conducted by the sponsor (Metabogen AB) have shown that butyrate production from F. prausnitzii increases in the presence of Desulfovibrio piger, a common sulphate‐reducing bacterium present in the human intestine. The symbiotic relationship between F. prausnitzii and D. piger can be utilised by combining these bacterial species into a probiotic dietary supplement, thus maintaining butyrate production in the intestine. In animal models, who received approximately 5,000 times higher doses per kilogram of body weight than the highest dose scheduled in the proposed study, the intake of F. prausnitzii has shown anti‐inflammatory effects as well as positive effects on the metabolism. The present study is a double‐blind, placebo‐controlled, randomized, study in 48 healthy individuals (men and women) between 20 and 40 years old recruited from the general population. These volunteers will either receive F. prausnitzii and D. piger (in two different doses) or placebo orally once a day for 8 consecutive weeks. The investigators will assess how well treatment with the study product compared to placebo is tolerated (termination due to adverse events within 8 weeks of treatment) and if it can cause gastrointestinal symptoms (measured with The Gastrointestinal Symptom Rating Scale). The investigators will also assess if the intake of the study product can potentially give positive effects in the metabolism (blood glucose, fatty acids, protein ect).

%Z https://clinicaltrials.gov/show/NCT03728868

Trial registry record

%U https://cochrane.66557.net/central/doi/10.1002/central/CN-01918304/full

%0 Journal Article

%A D'Haens, G. R.

%A Vermeire, S.

%A Van Assche, G.

%A Noman, M.

%A Aerden, I.

%A Van Olmen, G.

%A Rutgeerts, P.

%D 2008

%T Therapy of metronidazole with azathioprine to prevent postoperative recurrence of Crohn's disease: a controlled randomized trial

%V 135

%N 4

%P 1123‐1129

%8 2008-01-01

%R 10.1053/j.gastro.2008.07.010

%K Adult; Aged; Anti‐Infective Agents [*administration & dosage, adverse effects]; Azathioprine [*administration & dosage, adverse effects]; Combined Modality Therapy; Crohn Disease [*drug therapy, epidemiology, *surgery]; Drug Therapy, Combination; Female; Humans; Immunosuppressive Agents [*administration & dosage, adverse effects]; Incidence; Male; Metronidazole [*administration & dosage, adverse effects]; Middle Aged; Patient Dropouts; Postoperative Complications [epidemiology, prevention & control]; Risk Factors; Secondary Prevention; Treatment Outcome

%X BACKGROUND & AIMS: More than 80% of Crohn's disease (CD) patients undergoing resection suffer recurrence of their disease. Therapy with aminosalicylates, antimetabolites, or antibiotics leads to a modest reduction in the incidence of recurrence. Goal: We sought to examine whether metronidazole for 3 months together with azathioprine (AZA) for 12 months is superior to metronidazole alone to reduce recurrence of postoperative CD in "high‐risk" patients. METHODS: CD patients undergoing curative ileocecal resection with >or=1 risk factor for recurrence received metronidazole (3 months) and AZA/placebo (12 months). The primary end point was the proportion of patients with significant endoscopic recurrence 3 and 12 months after surgery. Secondary end points included clinical recurrence, safety, and tolerability of treatment. RESULTS: Eighty‐one patients were randomized; 19 discontinued the study early. Significant endoscopic recurrence was observed in 14 of 32 (43.7%) patients in the AZA group and in 20 of 29 (69.0%) patients in the placebo group at 12 months postsurgery (P = .048). Intention‐to‐treat analysis revealed endoscopic recurrence in 22 of 40 (55%) in the AZA group and 32 of 41 (78%) in the placebo group at month 12 (P = .035). At month 12, 7 of 32 patients had no endoscopic lesions in the AZA group, versus 1 of 29 in the placebo group (P = .037). CONCLUSIONS: Despite the enhanced risk of recurrence, the overall incidence of significant recurrence was rather low, probably owing to the metronidazole treatment that all patients received. Concomitant AZA resulted in lower endoscopic recurrence rates and less severe recurrences 12 months postsurgery, predicting a more favorable clinical outcome. This combined treatment seems to be recommendable to all operated CD patients with an enhanced risk for recurrence.

%Z Gastroenterology

Journal article

%U https://cochrane.66557.net/central/doi/10.1002/central/CN-00650750/full

%0 Journal Article

%A Dragoni, G.

%A Ding, N.

%A Gecse, K. B.

%A Mansfield, J. C.

%A Kopylov, U.

%A Beaugerie, L.

%A Bossuyt, P.

%A Sebastian, S.

%A Milla, M.

%A Galli, A.

%A Et, Al.

%D 2019

%T The Y-ECC O/Clin Com 2019 survey on postoperative Crohn's disease recurrence suggests overtreatment with prophylactic biological therapy

%V 7

%N 8

%P 324‐325

%8 2019-01-01

%R 10.1177/205064061985467

%K *Crohn disease; *recurrence risk; Aged; Clinical assessment; Clinical examination; Conference abstract; Controlled study; Feces; Female; Gastroenterologist; Gastroenterology; Human; Ileocolonoscopy; Immunoprophylaxis; Laboratory test; Major clinical study; Male; Phenotype; Randomized controlled trial; Risk assessment; Risk factor; Surgery; University hospital

%X Introduction: Prevention of post‐operative recurrence (POR) is a controversial field in Crohn's disease. Despite evidence for some clinical risk factors favoring POR (previous ileocolonic resections, extensive resection, penetrating disease, active smoking), there is no total agreement among guidelines[1‐2] on initiation of postoperative prophylactic therapy, preferred drug as well as on the modality and timing of POR assessments. Aims & Methods: Our aim was to obtain an international perspective on the current adopted therapies and strategies (immediate postoperative prophylaxis versus endoscopy‐driven) with regard to prevention of POR. A 11‐question anonymous survey was proposed to all gastroenterologists participating at various ECCO pre‐meeting workshops in March 2019. Results: Data from 168 participants were collected (participation rate 24%). Median gastroenterology experience was 8 years [IQR 4‐14 years], 65% junior (≤10 years of practice), 35% senior (>10 years). The majority were European (67%), while the remainder were representatives of 19 countries across all five continents. Most participants (60%) were working in an academic hospital. Post‐operative endoscopic evaluation to assess POR was routinely performed within 12 months by 87% of respondents. This was the same for patients who do or do not receive immediate postoperative prophylactic therapy. After a first endoscopy without POR, 89% of physicians reported following up patients with a combination of clinical examination, lab test and fecal calprotectin, and 45% considered endoscopy as a routine assessment modality. Comparison between young and senior gastroenterologists did not show any statistical difference (p>0.05). Most respondents (60%) reported starting medical prophylaxis against POR in naive patients if 1 or more risk factors are present, while 16% of respondents would prescribe immunoprophylaxis even in patients without risk factors. Only 20% of participants reported that they would wait for endoscopic proof of recurrence (Rutgeerts score ≥i2) before starting immunomodulators. Data were similar between young and senior gastroenterologists (p>0.05). In regard to the class of drug, the number of POR risk factors lead the majority (59%) to prescribe biologics over immunosuppressants. Among biologics, 98% of respondents having access to anti‐TNF reported a routine use of them in this setting, while 62% and 56% of physicians with access to vedolizumab and ustekinumab respectively prescribed these drugs for POR prophylaxis. In the specific clinical context of perianal disease, fistulizing phenotype or concomitant colonic disease, most of the participants reported adopting an immediate POR prophylaxis in 98%, 85% and 88% of cases, respectively. Conversely, a significant number of respondents were more prone to an endoscopy‐driven treatment in the elderly (42%) and in long‐standing disease after failure of thiopurines (51%). No significant difference (p>0.05) in post‐operative approach was registered in these scenarios between young and experienced gastroenterologists. Conclusion: Clinicians are well aware of the risk of POR in Crohn's disease and tight endoscopic control within twelve months is often proposed. After a first ileocolonoscopy without POR, most respondents reported relying on fecal calprotectin for routine monitoring. The surprisingly high rate of immediate postoperative prophylactic therapy with biologics, even in the absence of clear endoscopic recurrence or clinical risk factors for POR, highlights the need for a randomized trial comparing immediate prophylaxis with endoscopy‐driven therapy.

%Z United European gastroenterology journal

Journal article; Conference proceeding

%U https://cochrane.66557.net/central/doi/10.1002/central/CN-02138300/full

%0 Journal Article

%A NCT

%D 2019

%T The Vitamin D in Pediatric Crohn's Disease ( ViDiPeC-2 )

%8 2019-01-01

%K Cholecalciferol; Crohn Disease; Ergocalciferols; Immunosuppressive Agents; Vitamin D; Vitamins

%X Crohn's disease is a chronic inflammatory condition affecting all segments of the digestive tract from the mouth to the anus. This condition is associated with an increased risk of relapses throughout the course of the disease. Nearly 25% of patients with Crohn's disease are in the pediatric age range. Many epidemiological data are in favor of an increase incidence of pediatric Crohn's disease. Environmental factors could explain this increased incidence. Among them sunlight exposure and vitamin D deficiency have been suggested by many authors. Recent studies have described how varying doses of oral vitamin D supplementation can alter serum levels of 25 hydroxyvitamin D (25(OH)D), but no study has specifically addressed the question as to whether vitamin D supplementation can alter the rate of relapse/complications and/or quality of life in children diagnosed with CD. Current treatments of CD at diagnosis are effective around the time of diagnosis, but in the short and long term, some of these therapies are inefficient or lead to allergic or intolerance reactions. Altogether the rate of relapses in the year after diagnosis is significant. Thus, different therapeutic approaches must be investigated with the aim of lowering the burden of the disease. From November 2012 to July 2013, we conducted an open label pilot cohort study aiming to investigate the bioavailability and tolerance of high doses of vitamin D3 (3,000 IU or 4,000 IU per day) administered orally as an adjunct therapy in 20 children with newly diagnosed pediatric CD (http://clinicaltrials.gov/ct2/show/NCT01692808). Data from laboratory studies, observational research and pilot trials taken together suggest that vitamin D can be of great importance in the genesis and progression of CD. Vitamin D deficiency could be a true risk factor for disease occurrence and/or relapses. The results of our pilot study demonstrate that in children with active CD at diagnosis, a daily dose of 4,000 IU of vitamin D is well tolerated and quickly increases the blood levels of 25OHD3 to 100 nmol/L or above in 100% of children with CD at diagnosis. Moreover a maintenance dosage of 2,000 IU a day is required (and sufficient) for maintaining this target over several months. Currently there is no adequately powered study in the pediatric CD population exploring the relationship between vitamin D therapy at diagnosis and CD outcomes. We propose a randomized controlled trial (RCT) to study the efficacy of high‐dose oral vitamin D, as adjunct therapy, in children diagnosed CD, to reduce the relapse rate and to improve patients' quality of life. Primary Efficacy End Point: The proportion of patient with at least one relapse 52 weeks after randomization. Secondary efficacy endpoint: Quality of life scores, Cumulative steroid dose, Time to first relapse, Duration of corticotherapy, Number of relapses, Number of hospitalizations Safety Endpoint : incidence of hypercalcemia (defined as a corrected serum calcium level >2.65 mmol/L), incidence of hypercalciuria (defined as urinary calcium to creatinine molar ratio ≥1.50), incidence of supra‐optimal levels of 25OHD3 as defined by a serum level ≥ 250 nmol/L, rate of study discontinuation due to hypercalcemia or hypercalciuria. Efficacy Variable: Occurrence of relapse, Time to relapse, Change in QoL score from baseline to 26 weeks, 52 weeks. Change in physical activity score from baseline to 26 weeks, 52 weeks

%Z https://clinicaltrials.gov/show/NCT03999580

Trial registry record

%U https://cochrane.66557.net/central/doi/10.1002/central/CN-01952839/full

%0 Journal Article

%A NCT

%D 2014

%T The Vitamin D in Pediatric Crohn's Disease

%8 2014-01-01

%K Cholecalciferol; Crohn Disease; Ergocalciferols; Immunosuppressive Agents; Vitamin D; Vitamins

%X Crohn's disease is a chronic inflammatory condition affecting all segments of the digestive tract from the mouth to the anus. This condition is associated with an increased risk of relapses throughout the course of the disease. Nearly 25% of patients with Crohn's disease are in the pediatric age range. Many epidemiological data are in favor of an increase incidence of pediatric Crohn's disease. Environmental factors could explain this increased incidence. Among them sunlight exposure and vitamin D deficiency have been suggested by many authors. Recent studies have described how varying doses of oral vitamin D supplementation can alter serum levels of 25 hydroxyvitamin D (25(OH)D), but no study has specifically addressed the question as to whether vitamin D supplementation can alter the rate of relapse/complications and/or quality of life in children diagnosed with CD. Current treatments of CD at diagnosis are effective around the time of diagnosis, but in the short and long term, some of these therapies are inefficient or lead to allergic or intolerance reactions. Altogether the rate of relapses in the year after diagnosis is significant. Thus, different therapeutic approaches must be investigated with the aim of lowering the burden of the disease. From November 2012 to July 2013, we conducted an open label pilot cohort study aiming to investigate the bioavailability and tolerance of high doses of vitamin D3 (3,000 IU or 4,000 IU per day) administered orally as an adjunct therapy in 20 children with newly diagnosed pediatric CD (http://clinicaltrials.gov/ct2/show/NCT01692808). Data from laboratory studies, observational research and pilot trials taken together suggest that vitamin D can be of great importance in the genesis and progression of CD. Vitamin D deficiency could be a true risk factor for disease occurrence and/or relapses. The results of our pilot study demonstrate that in children with active CD at diagnosis, a daily dose of 4,000 IU of vitamin D is well tolerated and quickly increases the blood levels of 25OHD3 to 100 nmol/L or above in 100% of children with CD at diagnosis. Moreover a maintenance dosage of 2,000 IU a day is required (and sufficient) for maintaining this target over several months. Currently there is no adequately powered study in the pediatric CD population exploring the relationship between vitamin D therapy at diagnosis and CD outcomes. We propose a randomized controlled trial (RCT) to study the efficacy of high‐dose oral vitamin D, as adjunct therapy, in children with newly diagnosed CD, to reduce the relapse rate and to improve patients' quality of life. Primary Efficacy End Point: The proportion of patient with at least one relapse 52 weeks after randomization. Secondary efficacy endpoint: Quality of life scores, Cumulative steroid dose, Time to first relapse, Duration of corticotherapy, Number of relapses, Number of hospitalizations Safety Endpoint : incidence of hypercalcemia (defined as a corrected serum calcium level >2.65 mmol/L), incidence of hypercalciuria (defined as urinary calcium to creatinine molar ratio ≥1.50), incidence of supra‐optimal levels of 25OHD3 as defined by a serum level ≥ 250 nmol/L, rate of study discontinuation due to hypercalcemia or hypercalciuria. Efficacy Variable: Occurrence of relapse, Time to relapse, Change in QoL score from baseline to 26 weeks, 52 weeks. Change in physical activity score from baseline to 26 weeks, 52 weeks.

%Z https://clinicaltrials.gov/show/NCT02186275

Trial registry record

%U https://cochrane.66557.net/central/doi/10.1002/central/CN-01547120/full

%0 Journal Article

%A Ferrante, M.

%A Bislenghi, G.

%A Sabino, J.

%A D'Hoore, A.

%A Vermeire, S.

%D 2020

%T THE ROLE OF ANTIMICROBIAL MARKERS IN PREDICTING POSTOPERATIVE RECURRENCE IN PATIENTS WITH CROHN'S DISEASE: a POST-HOC ANALYSIS FROM PREVENT

%V 158

%N 6

%P S‐713

%8 2020-01-01

%R 10.1016/S0016-5085(20)32469-0

%K *Crohn disease; *post hoc analysis; Abscess; Adult; Cohort analysis; Conference abstract; Controlled study; Crohn Disease Activity Index; Current smoker; Drug therapy; Escherichia coli; Female; Fistula; Flax; Gastroenterology; Gene expression; Human; Human tissue; Major clinical study; Male; Nonhuman; Outer membrane; Predictive value; Preoperative evaluation; Prevention; Prospective study; Protein expression; Randomized controlled trial; Recurrent disease; Saccharomyces cerevisiae; Surgery; Univariate analysis

%X Background and Aims: Predicting Crohn’s disease (CD) recurrence after an ileocolonic resection with ileocolonic anastomosis remains challenging. Two independent cohorts previously suggested a predictive role of perioperative anti‐flagellin antibodies (anti‐Fla2 and anti‐FlaX).1‐2 Furthermore, a risk panel including anti‐Fla2, pANCA and active smoking was identified.1 We aimed to confirm these findings in prospectively collected data from PREVENT, a placebo‐controlled postoperative CD recurrence prevention trial with infliximab.3 Methods: We examined the 144 patients (54% males, 24% active smokers, 31% with previous resections, median age 34.0 years) from PREVENT who were randomised to placebo and who had a baseline serum sample available (within 1 month after surgery). The primary endpoint was clinical recurrence (CR), a composite outcome consisting of a Crohn’s Disease Activity Index score?>200 and a ≥70‐point increase from baseline, and endoscopic recurrence (ER) or development of a new fistula or abscess, before or at week 76. Endoscopic recurrence (Rutgeerts score ≥i2 at week 76, determined by a blinded central reader) was a secondary endpoint. Sera were analysed blindly at Prometheus laboratories for the expression of anti‐Saccharomyces cerevisiae IgA (ASCA IgA) and IgG antibodies (ASCA IgG), three different anti‐flagellin antibodies (CBir1, Fla2 and FlaX), antibodies to the outer‐membrane porin C of Escherichia coli (OmpC), and atypical perinuclear antineutrophilic cytoplasmic antibodies (pANCA). The Q3 value of each individual marker in this dataset was defined as the cutoff point. Predictors of both ER and CR in univariate analyses (p<0.1) were included in a binary logistic analysis. Results: By week 76, 21% of patients had developed CR and 62% ER. None of the perioperative serological markers was predictive of CR or ER (Table 1). As shown in Figure 1, the previously identified risk panel was not predictive of CR or ER (linear‐by‐linear association p=0.579 and 0.849, respectively). Previous ileocolonic resection was the only independent predictor of CR [Odds ratio 2.963 (95% confidence interval 1.272 – 6.898), p=0.012], while both previous ileocolonic resection [3.083 (1.358 – 6.997), p=0.007] and baseline CRP?> 5 mg/L [2.250 (1.037 – 4.880), p=0.040] independently predicted ER. Conclusions: Using the PREVENT data set, we were not able to confirm the predictive value of pre‐operative serological markers, including anti‐flagellin Fla2 and FlaX antibodies. Although the design of the PREVENT trial and the definitions of both CR and ER were different compared to previous trials, our data do not support the use of serological testing to predict recurrence of CD following surgery. References: FerranteM et al. Gastroenterology 2014; S‐443. Hamilton AM et al. Gastroenterology 2015: S‐116. Regueiro M et al. Gastroenterology 2016; 150: 1568‐1578.

%Z Gastroenterology

Journal article; Conference proceeding

%U https://cochrane.66557.net/central/doi/10.1002/central/CN-02131290/full

%0 Journal Article

%A Day, A. S.

%A Leach, S. T.

%A Judd, T. A.

%A Baba, K.

%A Hill, R. J.

%A Lemberg, D. A.

%D 2012

%T The probiotic VSL#3 in children with crohn disease in remission

%V 142

%N 5

%P S377

%8 2012-01-01

%K *Crohn disease; *VSL3; *child; *human; *probiotic agent; *remission; Adult; Bacterial strain; Boy; Concentration (parameters); Controlled study; Crohn disease; Diagnosis; Disease activity; Drug megadose; Inflammation; Intestine; Male; Marker; Nutritional parameters; Parameters; Patient; Placebo; Quality of life; Recurrence risk; Serum; Supplementation; Therapy; Thrombocyte count; Ulcerative colitis; Weight; adult; bacterial strain; boy; child; concentration (parameter); controlled study; diagnosis; disease activity; drug megadose; human; inflammation; intestine; male; nutritional parameters; parameters; patient; platelet count; quality of life; recurrence risk; remission; serum; supplementation; therapy; ulcerative colitis; weight

%X Background and Aims: VSL#3, a probiotic preparation containing 8 bacterial strains at a high concentration, has proven clinical efficacy in the induction and maintenance of remission of ulcerative colitis in children and adults. However, the role of this agent in the maintenance of remission in conjunction with standard therapy has not yet been evaluated in pediatric Crohn disease (CD). The primary aim of this study was to assess the role of VSL#3 in the maintenance of remission of children with CD, whilst secondary end‐points included serum and faecal inflammatory markers, growth parameters and quality of life (QOL) scores. Methods: A randomised, double‐blind, placebo‐controlled study was conducted in children aged between 4 and 18 years with existing diagnosis of CD. Additional inclusion criteria was current remission based upon Pediatric CD Disease Activity Index (PCDAI) scores less than 15. Children were randomised to receive active probiotic (standard dose of 1,800 million organisms daily with adjustment for weight) or placebo for 12 months, with evaluation of length of remission, requirement for rescue therapy, serum and faecal inflammatory markers, growth and quality of life (QOL) scores. Results: Data from 28 children was available for evaluation. Twelve children (10 boys) of average age of 13.3 (SD 3.0) years were randomised to receive VSL#3 and 16 children (11 boys) with mean age of 13.9 (SD 2.4) years were randomised to receive placebo. At baseline PCDAI scores, serum inflammatory markers and growth parameters were not different between the groups. QOL scores were not different at baseline or at any subsequent time‐points between the two groups (p>0.05). Relapse rates did not differ between the two groups (p>0.05). Platelet counts were greater at 6 months in the placebo group compared to the probiotic group (310 ± 58 vs 254 ± 58: p=0.03). Other serum inflammatory markers did not differ between the two groups at any time points. Levels of the fecal inflammatory marker, S100A12, appeared higher in the placebo group than the probiotic group at months 6, 9 and 12, and indeed approached significance at month 6 (p < 0.055). Conclusions: In this 12 month study in children with CD in remission, high‐dose probiotic therapy did not influence the relapse rate when assessed in comparison to standard therapy. However, there was a trend towards decreasing faecal markers of gut inflammation with VSL#3, and such results need to be confirmed in a larger study. In consideration of its beneficial effects on inflammatory and nutritional parameters in pediatric patients with active CD (Day et al, DDW 2012), we hypothesize that better results could be observed in the quiescent phase of the disease if VSL#3 supplementation is started during active disease.

%Z Gastroenterology

Journal article; Conference proceeding

%U https://cochrane.66557.net/central/doi/10.1002/central/CN-01026321/full

%0 Journal Article

%A Dragoni, G.

%A Ding, N.

%A Gecse, K. B.

%A Mansfield, J. C.

%A Kopylov, U.

%A Beaugerie, L.

%A Bossuyt, P.

%A Sebastian, S.

%A Milla, M.

%A Bagnoli, S.

%A Et, Al.

%D 2020

%T The prevention and management of Crohn's disease postoperative recurrence: results from the Y-ECCO/ClinCom 2019 Survey

%V 32

%N 8

%P 1062‐1066

%8 2020-01-01

%R 10.1097/MEG.0000000000001729

%K *colon Crohn disease; *immunoprophylaxis; Aged; Clinical assessment; Colonoscopy; Controlled study; Crohn Disease [diagnosis, drug therapy]; Female; Follow up; Gastroenterologist; Human; Humans; Male; Phenotype; Postoperative Period; Prescription; Randomized controlled trial; Recurrence; Review; Risk factor; Surgery; Surveys and Questionnaires; Treatment failure

%X Background: Prevention and management of postoperative recurrence (POR) is a controversial field in Crohn's disease. The aim of this survey was to report common practice in real‐life settings. Methods: An 11‐question survey was distributed among gastroenterologists attending the 14th European Crohn's and Colitis Organisation (ECCO) congress. Results: Postoperative endoscopy to assess recurrence was routinely performed within 12 months by 87% of respondents. Forty‐six percent of clinicians reported to maintain endoscopic assessment in routine follow‐up even after first negative colonoscopy. Most respondents (60%) considered starting postoperative immunoprophylaxis in naïve patients if one or more known risk factors were present. The number of risk factors was an important driver for prescribing biologics over immunosuppressants for 60% of respondents. In case of fistulizing phenotype, perianal disease, or concomitant colonic involvement, the majority of physicians reported to start an immediate prophylaxis in 85, 98 and 88% of patients, respectively. A significant percentage of clinicians were more prone to an endoscopy‐driven treatment in long‐standing disease after failure of thiopurines (51%) and elderly (43%). Conclusion: Endoscopy within the first year after surgery to assess POR has become routine in most centres. The high rate of early prophylaxis with expensive biologics despite missing solid evidence highlights the need for more randomized trials.

%Z European journal of gastroenterology & hepatology

Journal article

%U https://cochrane.66557.net/central/doi/10.1002/central/CN-02139986/full

%0 Journal Article

%A Narula, N.

%A Wong, ECL

%A Dulai, P. S.

%A Marshall, J. K.

%A Jairath, V.

%A Reinisch, W.

%D 2023

%T The Performance of the Rutgeerts Score, SES-CD, and MM-SES-CD for Prediction of Postoperative Clinical Recurrence in Crohn's Disease

%V 29

%N 5

%P 716‐725

%8 2023-01-01

%R 10.1093/ibd/izac130

%K *Crohn disease; *inflammatory bowel disease; *postoperative complication; *prediction; Article; Colon [surgery]; Colonoscopy; Complication; Controlled study; Crohn Disease [drug therapy]; Endoscopy; Human; Humans; Ileum [surgery]; Infliximab [therapeutic use]; Post hoc analysis; Prospective Studies; Recurrence; Surgery

%X BACKGROUND: We compared the Simple Endoscopic Score for Crohn's Disease (SES‐CD) and Modified Multiplied SES‐CD (MM‐SES‐CD) scores with the Rutgeerts score for predicting clinical recurrence (CR) of postoperative Crohn's disease (CD). METHODS: This post hoc analysis of the prospective, multicenter, randomized, double‐blind, placebo‐controlled trial comparing remicade and placebo in the prevention of recurrence in Crohn's disease patients undergoing surgical resection who are at an increased risk of recurrence (PREVENT) study used receiver operating characteristic curve analyses to compare the Rutgeerts, SES‐CD, and MM‐SES‐CD scores at week 76 for subsequent CR by week 104 in 208 participants. Multivariate logistic regression models evaluated cutoffs for the odds of experiencing CR by week 104, after adjustment for confounders. CR was defined as Crohn's Disease Activity Index score ≥200 and ≥70‐point increase from baseline (or development of fistulas, abscesses, or treatment failure) and endoscopic recurrence by week 104, defined as Rutgeerts score ≥i2. RESULTS: The week 76 Rutgeerts score predicted CR by week 104 with fair accuracy (area under the receiver‐operating characteristic curve [AUC], 0.74; 95% confidence interval [CI], 0.65‐0.83), which was similar to the SES‐CD ileum score (AUC, 0.72; 95% CI, 0.64‐0.80) and the MM‐SES‐CD ileum score (AUC, 0.72; 95% CI, 0.63‐0.80). Compared with cutoffs by the other scores, the MM‐SES‐CD total score ≥26 at week 76 had the highest odds ratio to predict CR by week 104. Patients with a week 76 MM‐SES‐CD total score ≥26 were 4.41 times (95% CI, 2.06‐9.43, P < .001) more likely to have CR by week 104 compared with those with an MM‐SES‐CD total score <26. CONCLUSIONS: The SES‐CD and MM‐SES‐CD perform similarly to the Rutgeerts score for predicting subsequent CR of postoperative CD. The MM‐SES‐CD threshold of ≥26 was predictive of postoperative CR. Clinicians and trialists could consider using the SES‐CD or MM‐SES‐CD to assess postoperative CD given their ability to capture colonic disease recurrence and predict CR.

%Z Inflammatory bowel diseases

Journal article

%U https://cochrane.66557.net/central/doi/10.1002/central/CN-02457863/full

%0 Journal Article

%A NCT

%D 2021

%T The OPTIMIZE Trial

%8 2021-01-01

%K Crohn Disease; Infliximab

%X Preliminary data show that proactive IFX optimization to achieve a threshold drug concentration during maintenance therapy (even if the patient is asymptomatic) compared to empiric dose escalation and/or reactive TDM is associated with better long‐term outcomes including longer drug persistence, reduced risk of relapse, and fewer hospitalizations and surgeries. IFX dosing by weight only (i.e., mg/kg) may not be adequate for many patients as interindividual variability in drug clearance and other factors affecting IFX concentrations and PK are often not accounted for. Dosing calculators take into account all of these individual factors and improve the precision of dosing towards better personalized medicine. These systems have already been validated, and personalized dosing has shown clinical benefit in patients with IBD.

%Z https://clinicaltrials.gov/show/NCT04835506

Trial registry record

%U https://cochrane.66557.net/central/doi/10.1002/central/CN-02253612/full

%0 Journal Article

%A Keefer, L.

%D 2014

%T The impact of behavioral interventions on gastrointestinal diseases

%V 76

%N 3

%P A‐59

%8 2014-01-01

%R 10.1097/PSY.0000000000000057

%K *gastrointestinal disease; *psychosomatics; *society; Analysis of variance; Anxiety; Arm; Arousal; Behavior therapy; Catastrophizing; Clinical trial; Cognitive therapy; Diseases; Human; Inflammatory bowel disease; Intestine; Irritable colon; Patient; Patient worry; Prevention study; Recurrence risk; Relapse; Remission; Skill; Ulcerative colitis

%X Background: There is strong evidence for gut‐directed hypnotherapy (HYP) and cognitive‐behavior therapy (CBT) in irritable bowel syndrome (IBS). These interventions succeed because they address centrally‐mediated symptom processes‐ catastrophizing, symptom anxiety and autonomic arousal. Research also suggests that these interventions can be translated to “organic” GI disorders, including inflammatory bowel diseases (IBD). We will demonstrate the potential for behavior therapies across the spectrum of GI diseases in the context of the Irritable Bowel Syndrome Outcome Study (IBSOS) and the Ulcerative Colitis Relapse Prevention Trial (UCRPT). Study 1: IBSOS is an ongoing, NIH‐funded trial of brief (4 session) CBT for IBS. The protocol was developed by Lackner and colleagues in response to research showing that IBS patients have better outcomes when CBT is delivered rapidly. Skills targeted in brief CBT map directly onto processes driving persistent symptoms including catastrophizing, worry and stress responsivity. The trial has 2 control arms‐education/support and standard CBT. 320 participants have been enrolled. While results will not yet be available, we will discuss the relevance of this research for the future of IBS management. Study 2: UCRPT is a recently completed NIH‐funded clinical trial of HYP for quiescent ulcerative colitis (UC). 54 patients were randomized to 7 sessions of HYP (n = 26) or attention control (CON; n = 29) and followed for 1 year to determine the impact of hypnotherapy on remission status/days to relapse. Hypnotherapy targeted factors influencing relapse rate‐immune function, heightened symptom awareness and stress. ANOVA comparing HYP and CON subjects on number of days to clinical relapse favored the HYP condition [F = 4.8 (1, 48), P = 0.03] by 78 days. Chi‐square comparing the groups on proportion maintaining remission at 1 year was also significant [X2(1) = 3.9,P = 0.04]‐68% of HYP vs 40% CON maintained remission for 1 year. Conclusion: Behavioral therapies show promise across a range of gastrointestinal diseases by targeting underlying disease processes, including cognitive‐affective dysregulation, stress and immune function.

%Z Psychosomatic medicine

Journal article; Conference proceeding

%U https://cochrane.66557.net/central/doi/10.1002/central/CN-01061478/full

%0 Journal Article

%A ChiCTR-IOR-

%D 2017

%T The effect of enteral nutrition in one year recurrence rate after bowel resection in patients with crohn’s disease: a prospective randomized controlled study

%8 2017-01-01

%X INTERVENTION: treatment group:exclusive enteral nutrition for 4 weeks after operation;control group:Conventional treatment; CONDITION: crohn's disease PRIMARY OUTCOME: clinical recurrence; INCLUSION CRITERIA: 1. Aged 16 to 65 years old; 2. The diagnosis of crohn's disease was clearly, and had the indication for surgery; 3. The patients had nutritional risk or malnutrition (NRS 2002 risk scores=3 or BMI < 18.5 kg/m2, or preoperative albumin< 30 g/L); 4. The patients could tolerate the enteral nutrition.

%Z https://trialsearch.who.int/Trial2.aspx?TrialID=ChiCTR-IOR-17011703

Trial registry record

%U https://cochrane.66557.net/central/doi/10.1002/central/CN-01885650/full

%0 Journal Article

%A ISRCTN

%D 2004

%T The effect of combined n-3 polyunsaturated fatty acid and antioxidant dietary supplements on Crohn's disease & the associated osteoporosis, malnutrition and morbidity

%8 2004-01-01

%X INTERVENTION: 1. The dietary intervention consists of: 9 capsules per day of (Maxepa) fish oil (1.62 g of eicosapentaenoic acid, 1.08 g of docosahexanoic acid) and 1 capsule per day of antioxidant vitamins containing selenium 200 ug (reference nutrient intake [RNI] 75 ug/day) manganese 3 mg (UK intake 5.5 mg/day), vitamin A 450 ug (RNI 700 ug) vitamin E 30 ug (average UK intake 5‐7 mg), vitamin C 90 mg (RNI 40 mg). 2. Placebo will consist of 9 capsules containing olive oil and 1 containing sugar. The placebos are indistinguishable from the active treatments. CONDITION: Digestive system diseases: Inflammatory bowel disease; Musculoskeletal diseases: Osteoporosis ; Digestive System ; Crohn's disease PRIMARY OUTCOME: Maintenance of disease remission, as defined by an absence of disease relapses (recognised quantitative increase in Crohn's disease activity index (CDAI) score of 100 to an absolute value of greater than 150) during the 6 month intervention period. SECONDARY OUTCOME: 1. Biochemical markers of bone turnover (osteocalcin and deoxypridinoline); 2. Markers of inflammation (1L‐1, 1L‐6 and TNF‐a); 3. Quality of life score; 4. Nutritional status INCLUSION CRITERIA: Male and female patients aged between 18 and 75, with a diagnosis of Crohn's disease based on endoscopic, histological or radiological investigation. Patients will be: 1. At high risk of active disease based biochemical markers (i.e. a C‐reactive protein [CRP] >6.9 or erythrocyte sedimentation rate [ESR] >20) and history (disease relapse within the last 2 years with Crohn's Disease Activity Index [CDAI] >150). 2. Currently in remission and not requiring use of oral or intravenous steroids or other immunosuppression (with the exception of azathioprine) within the last month.

%Z https://trialsearch.who.int/Trial2.aspx?TrialID=ISRCTN69447524

Trial registry record

%U https://cochrane.66557.net/central/doi/10.1002/central/CN-01847056/full

%0 Journal Article

%A NCT

%D 2018

%T The CURE - CD Trial

%8 2018-01-01

%K Crohn Disease

%X 1. Entry procedures: Patients will undergo clinical examination and history taking and then will undergo patency capsule. If patency is proven, patients will undergo a third generation pan‐enteric capsule endoscopy (PillCam Crohn), ileocolonoscopy with biopsies (by separate consent), MRE, Intestinal US, biomarkers, immune & microbiome analysis, and health related quality of life assessment and patient reported outcome (PRO) standard questionnaires. (see Appendix 1 for complete protocol of baseline pan‐enteric capsule endoscopy and ileocolonoscopy evaluation, Appendix 2 for complete protocol of MRE examination, Appendix 3 for blood and stool sample collection protocols, Appendix 4 for Intestinal US protocol, appendix 5 for microbiomic analysis protocol, appendix 6 for health related quality of life/cost assessments, appendix 7 for Immune analysis). 2. Follow‐up procedures: Capsule endoscopy, nutritional and intestinal US studies will be performed every 6 months for the total study duration of 2 years. For patients with only small bowel disease these will be without preparation. Additionally, periodic (every 3 months) assessment will be performed for inflammatory, microbiome, imaging attributes, quality of life and immunophenotyping, as outlined below in description of tasks for work packages. 3. Risk stratification and outcomes: Based on VCE results and according to the predictive algorithm defined by the first project's results, patients will be classified as high risk if having Lewis score (LS) ≥350 for the small bowel tertile with the highest score, or as having low risk (LS<350 highest tertile score) for future relapse of disease. Low risk patients will continue the monitoring scheme and their treatment regimen unaltered. Patients with LS ≥350 will be randomized to either continue follow‐up with unaltered therapy or to proactive therapy optimization with TDM assessment. Therapy will be optimized based on the optimization protocol described below, with the aim to prevent flares and complications. Follow up to determine the occurrence of clinical flares and complications as well as the status of inflammatory process will be performed for all patients q3months. Study will be terminated and a patient will be withdrawn upon disease flare or complication or if a change of CD medications was instituted (except for treating verified infectious complication such as C. difficile infection) and such a patient will be considered a non‐responder. A patient will also be withdrawn if in the opinion of the Principle Investigator (PI), a new adverse event or medical condition is present that endangers the patient's wellbeing if the study protocol is adhered to. To maintain temporally‐restricted blinding, VCE results will be disclosed up to three months following the performance of VCE. * In a sub‐group of consenting patients, stool samples for microbiome analysis (see below) will be collected daily for a designated period of time. In a sub‐group of consenting patients, additional on‐line data collection methods will be employed (see below). Intervention protocol in proactive arm based on current treatment and TDM finding: Risk‐based intervention: Patients meeting the VCE‐based high‐risk criteria and who were randomized to proactive treatment arm will receive therapy intensification within 30 days. Re‐assessment of response to the intensified therapy will be performed by repeated VCE and bio‐markers after 6 months. The intensification protocol will be as appears below. Briefly, patients receiving immunomodulators,5‐Aminosalicylates (5ASA) or no treatment at the time they are found to have high risk of imminent flare or complication will receive induction with a biologic of the anti‐Tumor necrosis Factor (TNF)‐ Infliximab (IFX), Adalimumab (ADA)‐ or anti‐integrin (VDZ) classes as per standard induction protocols for these agents. The choice of the biologic will be guided by the treating physician's discretion as per the individual patient‐related considerations. In patients already on a biologic, intervention will be guided by protocolized TDM results for patients on anti‐TNFs, whereas patients receiving vedolizumab will first receive an interval‐halving intervention. Protocolized anti‐TNF TDM‐based intervention will comprise of increasing the dose of anti‐TNF, or switching anti‐TNF, or switching out‐of‐class according to the drug/anti‐drug antibodies thresholds and algorithms set by previous works by our group in this field, using the same assay that will be employed in the present trial. Re‐adjustment of therapy according to these principles will be performed if 6‐month VCE re‐assessment does not show a reduction of patient risk score to the range of a low risk VCE‐based score. Patients who received an intervention and in whom follow‐up 6 months VCE does not show a reduction of 225 points on the Lewis score or highest segment Lewis score of <350, will receive the next protocolized drug optimization. ADL with drug level<8mcg/ml/AAA<4mcg/ml‐eq ADL dose‐doubling ADL with drug level <8mcg/ml/AAA>4mcg/ml‐eq Switch anti TNF or add IMM ADL with drug level >8mcg/ml Switch out of class IFX with drug level <6mcg/ml/ATI<9mcg/ml‐eq IFX dose‐doubling IFX with drug level <6mcg/ml/ATI>9mcg/ml‐eq Switch anti TNF or add IMM IFX with drug level >6mcg/ml Switch out of class VDZ e/8 week VDZ interval halving VDZ e4W/CD3CD45RO target occupied >85% Switch out of class VDZ e4W/ CD3CD45RO target occupied<85% VDZ double‐dosing 600/4w UST 90mg/SC e12w or e/8W Shorten interval e/4w No treatment, 5‐ASA or AZA/6MP >> Start biologic ADL ‐ adalimumab, IFX‐ infliximab, VDZ ‐ vedolizumab UST ‐ Ustekinumab AAA‐ antibodies to adalimumab, ATI antibodies to infliximab, IMM ‐ immunomodulator

%Z https://clinicaltrials.gov/show/NCT03555058

Trial registry record

%U https://cochrane.66557.net/central/doi/10.1002/central/CN-01660287/full

%0 Journal Article

%A Hartman, T. J.

%A Albert, P. S.

%A Snyder, K.

%A Slattery, M. L.

%A Caan, B.

%A Paskett, E.

%A Iber, F.

%A Kikendall, J. W.

%A Marshall, J.

%A Shike, M.

%A Et, Al.

%D 2005

%T The association of calcium and vitamin D with risk of colorectal adenomas

%V 135

%N 2

%P 252‐259

%8 2005-01-01

%R 10.1093/jn/135.2.252

%K *calcium; *colorectal adenoma/ep [Epidemiology]; *dietary intake; *vitamin D; Adenoma [*epidemiology]; Adult; Aged; Article; Body Mass Index; Calcium; Calcium intake; Caloric intake; Clinical trial; Colonic Neoplasms [*epidemiology]; Colonic Polyps [prevention & control]; Colonoscopy; Controlled clinical trial; Controlled study; Diet; Diet Records; Dietary Supplements; Dietary fiber; Disease association; Disease predisposition; Educational Status; Female; Fruit; Human; Humans; Low fat diet; Major clinical study; Male; Middle Aged; Nonsteroid antiinflammatory agent; Randomized controlled trial; Rectal Neoplasms [*epidemiology]; Recurrence; Recurrent disease; Risk Factors; United States [epidemiology]; Vegetable; Vitamin D

%X The Polyp Prevention Trial (PPT) was a multicenter randomized clinical trial designed to determine the effects of a high‐fiber, high‐fruit and vegetable, low‐fat diet on the recurrence of adenomatous polyps in the large bowel. Detailed dietary intake and supplement use data were collected at baseline and at each of 4 annual study visits. Adenoma recurrence was ascertained by complete colonoscopy at baseline and after 1 and 4 y. Recurrence was found in 754 of the 1905 trial participants. We evaluated the association between calcium and vitamin D intake and adenomatous polyp recurrence after adjusting for intervention group, age, gender, nonsteroidal anti‐inflammatory drug use, total energy intake, and the interaction of gender and intervention group. Vitamin D models were also adjusted for the location of the clinic site. Dietary variables were adjusted for total energy intake via the residual method. There were no overall significant associations between adenoma recurrence and dietary calcium intake [odds ratio (OR) for the 5th compared with the lowest quintile = 0.91; 95% CI = 0.67‐1.23; P‐trend = 0.68], total calcium intake (OR = 0.86; 95% CI = 0.62‐1.18; P‐trend = 0.20), or dietary vitamin D intake (OR = 0.93; 95% CI = 0.69‐1.25; P‐trend = 0.43) averaged over follow‐up. Total vitamin D intake was weakly inversely associated with adenoma recurrence (OR = 0.84; 95% CI = 0.62‐1.13; P‐trend = 0.03). Supplemental calcium and vitamin D use during follow‐up also were inversely associated with adenoma recurrence (OR for any compared with no use = 0.82; 95% CI = 0.68‐0.99; and OR = 0.82; 95% CI = 0.68‐0.99; for calcium and vitamin D, respectively). Slightly stronger associations were noted for the prevention of multiple recurrences. Our analyses did not suggest a significant effect modification between total calcium and total vitamin D intake (P = 0.14) on risk for adenoma recurrence. This trial cohort provides some evidence that calcium and vitamin D may be inversely associated with adenoma recurrence.

%Z Journal of nutrition

Journal article

%U https://cochrane.66557.net/central/doi/10.1002/central/CN-00502941/full

%0 Journal Article

%A Ferrante, M.

%A Papamichael, K.

%A Duricova, D.

%A D'Haens, G. R.

%A Vermeire, S.

%A Archavlis, E. J.

%A Rutgeerts, P. J.

%A Bortlik, M.

%A Mantzaris, G. J.

%A Van Assche, G. A.

%D 2014

%T Systematic versus endoscopy-driven treatment with azathioprine to prevent postoperative ileal crohn's disease recurrence: interim results from a randomized, multicenter trial

%V 146

%N 5

%P S‐592

%8 2014-01-01

%R 10.1016/S0016-5085(14)62145-4

%K *azathioprine; *endoscopy; *gastrointestinal disease; *human; *multicenter study; *recurrent disease; Anastomosis; Arm; Ileum resection; Intention to treat analysis; Male; Patient; Patient preference; Prophylaxis; Remission; Risk; Smoking; Surgery; Therapy; Tumor necrosis factor inhibitor; anastomosis; arm; endoscopy; gastrointestinal disease; human; ileum resection; intention to treat analysis; male; multicenter study; patient; patient preference; prophylaxis; recurrent disease; remission; risk; smoking; surgery; therapy

%X Background & Aims: Preventing postoperative Crohn's disease (CD) recurrence remains challenging. Prophylactic therapy with azathioprine (AZA) has been shown efficacious, but it is unknown whether it should be started immediately after surgery in all patients. Therefore, we compared systematic versus endoscopy‐driven therapy with AZA in preventing CD recurrence at 24 months. Methods: Patients with CD undergoing curative ileal resection with ileocolonic anastomosis and at high risk of recurrence (smoker, perforating disease, age <30 years, previous resections, or recent use of anti‐TNF agents) were included in this prospective, multicentre, IOIBD sponsored trial. Patients were randomized to systematic AZA initiated ≤2 weeks from surgery (SYS‐AZA), or endoscopy‐driven AZA (ED‐AZA). Patients in the ED‐AZA arm underwent ileocolonoscopy at 6 and 12 months, and AZA was initiated at a standard dose of 2.0‐2.5mg/kg in case of endoscopic recurrence (≥i2). The primary endpoint was the proportion of patients with endoscopic remission at 24 months, defined as a post‐operative endoscopic recurrence score of i0 or i1. Secondary endpoints included the proportion of patients with i0, and the proportion of patients in clinical remission (CDAI<150) at 24 months. We estimated that 100 patients were required in each group to show superiority of SYS‐AZA for the primary endpoint (power 80%, alpha 5%). Only patients in whom the primary endpoint was accessible or who developed clinical recurrence within 24 months, were included in the per‐protocol (PP) analysis. Results: Due to slow recruitment, only 59 patients (26 male, median age 36.5 years) were randomized between 2005‐2011 and included in the intention‐to‐treat (ITT) analysis. Baseline characteristics are shown in Table 1. Eighteen of the 59 patients withdrew prematurely from the study (7 clinical recurrence, 5 adverse events due to AZA, 6 patient's preference). Of the 30 patients included in the ED‐AZA group, 10 and 4 patients initiated AZA at months 6 and 12, respectively. Both ITT and PP analyses revealed no difference in primary and secondary endpoints between the SYS‐AZA and ED‐AZA group (Table 2). In the ITT analysis, endoscopic remission was achieved by 52% in the SYS‐AZA and 43% in the ED‐AZA group (p=0.519). Conclusions: Although this study was underpowered, we could not observe a benefit of systematic post‐operative prophylactic therapy with AZA in patients at high risk of postoperative CD recurrence. Early post‐operative endoscopic evaluation to guide further therapy seems most appropriate, but more studies are warranted. (Table Presented).

%Z Gastroenterology

Journal article; Conference proceeding

%U https://cochrane.66557.net/central/doi/10.1002/central/CN-01061180/full

%0 Journal Article

%A Ferrante, M.

%A Papamichael, K.

%A Duricova, D.

%A D'Haens, G.

%A Vermeire, S.

%A Archavlis, E.

%A Rutgeerts, P.

%A Bortlik, M.

%A Mantzaris, G.

%A Van Assche, G.

%D 2015

%T Systematic versus Endoscopy-driven Treatment with Azathioprine to Prevent Postoperative Ileal Crohn's Disease Recurrence

%V 9

%N 8

%P 617‐624

%8 2015-01-01

%R 10.1093/ecco-jcc/jjv076

%K *Crohn disease/dt [Drug Therapy]; *Crohn disease/pc [Prevention]; *Crohn disease/th [Therapy]; *azathioprine/ae [Adverse Drug Reaction]; *azathioprine/ct [Clinical Trial]; *azathioprine/dt [Drug Therapy]; *gastrointestinal endoscopy; *ileum; Adult; Anastomosis, Surgical; Article; Azathioprine [*therapeutic use]; Clinical Decision‐Making; Colon [*surgery]; Colon anastomosis; Colonoscopy; Controlled study; Crohn Disease [diagnosis, *prevention & control, surgery]; Drug Administration Schedule; Drug efficacy; Drug withdrawal; Early Termination of Clinical Trials; Female; Gastrointestinal symptom/si [Side Effect]; High risk patient; Human; Humans; Ileum [*surgery]; Immunosuppressive Agents [*therapeutic use]; Induction Chemotherapy; Infection/si [Side Effect]; Intention to Treat Analysis; Intermethod comparison; Leukopenia/si [Side Effect]; Major clinical study; Male; Middle Aged; Multicenter study; Pancreatitis/si [Side Effect]; Patient preference; Postoperative period; Priority journal; Prospective Studies; Prospective study; Randomized controlled trial; Recurrence; Recurrent disease; Remission; Rutgeerts score; Scoring system; Side effect/si [Side Effect]; Skin defect/si [Side Effect]; Systemic therapy; Thyroid cancer/si [Side Effect]; Treatment Outcome

%X BACKGROUND AND AIMS: Prophylactic azathioprine (AZA) is efficacious in preventing postoperative Crohn's disease (CD) recurrence. However, it is unknown whether AZA should be started immediately after surgery. We compared the efficacy of systematic vs endoscopy‐driven AZA in preventing CD recurrence at week 102. METHODS: This prospective, multicentre trial included CD patients undergoing curative resection with ileocolonic anastomosis and at higher risk of recurrence. Patients were randomized to systematic AZA initiated ≤2 weeks from surgery, or endoscopy‐driven AZA in which therapy was only initiated in case of endoscopic recurrence (Rutgeerts' score ≥i2) at weeks 26 or 52 following surgery. The primary endpoint was endoscopic remission (i0‐i1) at week 102. Secondary endpoints included complete endoscopic remission (i0) and clinical remission. RESULTS: The study was prematurely stopped due to slow recruitment. Between 2005 and 2011, 63 patients (28 male, median age 36 years) were randomized to systematic (n = 32) or endoscopy‐driven AZA (n = 31). Twenty‐one patients withdrew prematurely (8 clinical recurrence, 6 adverse reactions to AZA, 7 patient's preference). In the endoscopy‐driven AZA group, 14 patients had to initiate AZA (11 at week 26, 3 at week 52). Endoscopic remission was achieved by 50% in the systematic and 42% in the endoscopy‐driven AZA group (p = 0.521). No difference in secondary endpoints was found. CONCLUSIONS: Systematic AZA therapy in patients at higher risk of postoperative CD recurrence is not superior to endoscopy‐driven treatment. Early postoperative endoscopic evaluation between weeks 26 and 52 seems most appropriate to guide further therapy, but larger studies are warranted. (ClinicalTrials.gov NCT02247258.).

%Z Journal of Crohn's & colitis

Journal article

%U https://cochrane.66557.net/central/doi/10.1002/central/CN-01167156/full

%0 Journal Article

%A Ferrante, M.

%A Papamichael, K.

%A Duricova, D.

%A D'Haens, G.

%A Vermeire, S.

%A Archavlis, E.

%A Rutgeerts, P.

%A Bortlik, M.

%A Mantzaris, G.

%A Van Assche, G.

%D 2014

%T Systematic versus endoscopy-driven treatment with azathioprine to prevent postoperative ileal Crohn's disease recurrence

%V 8

%P S205‐S206

%8 2014-01-01

%K *azathioprine; *colitis; *endoscopy; *recurrent disease; Anastomosis; Arm; Human; Ileum resection; Intention to treat analysis; Male; Patient; Patient preference; Prophylaxis; Remission; Risk; Smoking; Surgery; Therapy; Tumor necrosis factor inhibitor; anastomosis; arm; colitis; endoscopy; human; ileum resection; intention to treat analysis; male; patient; patient preference; prophylaxis; recurrent disease; remission; risk; smoking; surgery; therapy

%X Background: Preventing postoperative Crohn's disease (CD) recurrence remains challenging. Prophylactic therapy with azathioprine (AZA) has been shown efficacious, but it is unknown whether it should be started immediately after surgery in all patients. Therefore, we compared systematic versus endoscopy‐driven therapy with AZA in preventing CD recurrence at 24 months. Methods: Patients with CD undergoing curative ileal resection with ileocolonic anastomosis and at high risk of recurrence (smoker, perforating disease, age <30 years, previous resections, or recent use of anti‐TNF agents) were included in this prospective, multicentre, IOIBD sponsored trial. Patients were randomized to systematic AZA initiated ≤2 weeks from surgery (SYS‐AZA), or endoscopy‐driven AZA (ED‐AZA). Patients in the ED‐AZA arm underwent ileocolonoscopy at 6 and 12 months, and AZA was initiated at a standard dose of 2.0‐2.5 mg/kg in case of endoscopic recurrence (≤i2). The primary endpoint was the proportion of patients with endoscopic remission at 24 months, defined as a post‐operative endoscopic recurrence score of i0 or i1. Secondary endpoints included the proportion of patients with i0, and the proportion of patients in clinical remission (CDAI <150) at 24 months. We estimated that 100 patients were required in each group to show superiority of SYS‐AZA for the primary endpoint (power 80%, alpha 5%). Only patients in whom the primary endpoint was accessible or who developed clinical recurrence within 24 months, were included in the per‐protocol (PP) analysis. Results: Due to slow recruitment, only 59 patients (26 male, median age 36.5 years, 22 smokers, 19 with previous resections) were randomized between 2005‐2011 and included in the intention‐to‐treat (ITT) analysis. Eighteen of the 59 patients withdrew prematurely from the study (7 clinical recurrence, 5 adverse events due to AZA, 6 patient's preference). Of the 30 patients included in the ED‐AZA group, 10 and 4 patients initiated AZA at months 6 and 12, respectively. Both ITT and PP analyses revealed no difference in primary and secondary endpoints between the SYS‐AZA and ED‐AZA group (Table). In the ITT analysis, endoscopic remission was achieved by 52% in the SYS‐AZA and 43% in the ED‐AZA group (p = 0.519). Conclusions: Although this study was underpowered, we could not observe a benefit of systematic post‐operative prophylactic therapy with AZA in patients at high risk of post‐operative CD recurrence. Early post‐operative endoscopic evaluation to guide further therapy seems most appropriate, but more studies are warranted. (Table presented) .

%Z Journal of Crohn's & colitis

Journal article; Conference proceeding

%U https://cochrane.66557.net/central/doi/10.1002/central/CN-01057524/full

%0 Journal Article

%A Luglio, G.

%A Rispo, A.

%A Imperatore, N.

%A Giglio, M. C.

%A Amendola, A.

%A Tropeano, F. P.

%A Peltrini, R.

%A Castiglione, F.

%A De Palma, G. D.

%A Bucci, L.

%D 2020

%T Surgical Prevention of Anastomotic Recurrence by Excluding Mesentery in Crohn's Disease: the SuPREMe-CD Study - A Randomized Clinical Trial

%V 272

%N 2

%P 210‐217

%8 2020-01-01

%R 10.1097/SLA.0000000000003821

%K *Crohn disease; *mesentery; Adult; Aged; Anastomosis, Surgical [*adverse effects, methods]; Article; Colectomy [*methods]; Colon [surgery]; Controlled study; Crohn Disease [diagnosis, *surgery]; Drug safety; Endoscopy [*adverse effects, methods]; Female; Follow‐Up Studies; Human; Humans; Ileum [surgery]; Logistic Models; Major clinical study; Male; Mesentery [*pathology]; Middle Aged; Postoperative Complications [epidemiology, physiopathology]; Prevention; Proportional Hazards Models; Randomized controlled trial; Recurrence; Recurrence risk; Risk Assessment; Secondary Prevention [*methods]; Severity of Illness Index; Side to side anastomosis; Tertiary Care Centers; Treatment Outcome

%X OBJECTIVE: This trial aimed to provide randomized controlled data comparing Kono‐S anastomosis and stapled ileocolic side‐to‐side anastomosis. BACKGROUND: Recently, a new antimesenteric, functional, end‐to‐end, hand‐sewn ileocolic anastomosis (Kono‐S) has shown a significant reduction in endoscopic recurrence score and surgical recurrence rate in Crohn disease (CD). METHODS: Randomized controlled trial (RCT) at a tertiary referral institution. Primary endpoint: endoscopic recurrence (ER) (Rutgeerts score ≥i2) after 6 months. Secondary endpoints: clinical recurrence (CR) after 12 and 24 months, ER after 18 months, and surgical recurrence (SR) after 24 months. RESULTS: In all, 79 ileocolic CD patients were randomized in Kono group (36) and Conventional group (43). After 6 months, 22.2% in the Kono group and 62.8% in the Conventional group presented an ER [P < 0.001, odds ratio (OR) 5.91]. A severe postoperative ER (Rutgeerts score ≥i3) was found in 13.8% of Kono versus 34.8% of Conventional group patients (P = 0.03, OR 3.32). CR rate was 8% in the Kono group versus 18% in the Conventional group after 12 months (P = 0.2), and 18% versus 30.2% after 24 months (P = 0.04, OR 3.47). SR rate after 24 months was 0% in the Kono group versus 4.6% in the Conventional group (P = 0.3). Patients with Kono‐S anastomosis presented a longer time until CR than patients with side‐to‐side anastomosis (hazard ratio 0.36, P = 0.037). On binary logistic regression analysis, the Kono‐S anastomosis was the only variable significantly associated with a reduced risk of ER (OR 0.19, P < 0.001). There were no differences in postoperative outcomes. CONCLUSIONS: This is the first RCT comparing Kono‐S anastomosis and standard anastomosis in CD. The results demonstrate a significant reduction in postoperative endoscopic and clinical recurrence rate for patients who underwent Kono‐S anastomosis, and no safety issues.ClinicalTrials.gov ID NCT02631967.

%Z Annals of surgery

Journal article

%U https://cochrane.66557.net/central/doi/10.1002/central/CN-02141841/full

%0 Journal Article

%A Luglio, G.

%A Rispo, A.

%A Imperatore, N.

%A Amendola, A.

%A Tropeano, F. P.

%A Peltrini, R.

%A Castiglione, F.

%A De Palma, G. D.

%A Corcione, F.

%A Bucci, L.

%D 2020

%T SURGICAL PREVENTION OF ANASTOMOTIC RECURRENCE BY EXCLUDING MESENTERY IN CROHN'S DISEASE: THE SUPREME-CD STUDY

%V 52

%P S37‐S38

%8 2020-01-01

%R 10.1016/S1590-8658(20)30580-6

%K *Crohn disease; *mesentery; Adult; Complication; Conference abstract; Controlled study; Feces; Female; Flatulence; Human; Major clinical study; Male; Operation duration; Patient referral; Postoperative care; Postoperative complication; Prevention; Randomized controlled trial; Recurrence risk; Side to side anastomosis; Surgery

%X Background and aim: Most patients with Crohn's disease (CD) experience disease recurrence after resection. Recently a new anti‐mesenteric, functional end‐to‐end, handsewn ileocolic anastomosis (Kono‐S) has been described, showing a significant reduction in endoscopic recurrence score and surgical recurrence rate. The aim of this trial was to provide randomized controlled data comparing Kono‐S anastomosis vs the stapled ileocolic side‐to‐side anastomosis. Materials and methods: Randomised controlled trial at a tertiary referral institution, enrolling and randomising (1:1) to undergo either the “Kono group” or the “Conventional group”, all CD subjects needing surgery. The primary endpoint was endoscopic recurrence (Rutgeerts score ≥i2) at 6 months, while secondary endpoints were clinical recurrence at 12 and 24 months (defined as a CDAI >200), endoscopic recurrence at 18 months and surgical recurrence at 24 months. Also short‐term outcomes and postoperative complications were recorded. Post‐operative treatment was established on the basis of clinical judgement and patient's profile. Results: 79 patients were enrolled and randomized in Kono group (36) or Conventional group (43) (Table 1). At 6 months, 8 patients in the Kono group (22.2%) and 27 patients in the Conventional group (62.8%) presented an endoscopic recurrence (Rutgeerts score ≥i2) (p<0.001; OR 5.91), with a mean Rutgeerts score (0‐4) of 0.92+1.05 and 2.06+1.31, respectively (p<0.001). Furthermore, a severe postoperative endoscopic recurrence (Rutgeerts score ≥i3) was found in 13.8% of Kono vs 34.8% of Conventional group (p=0.03; OR 3.32). Clinical recurrence rate was 8% in the Kono group vs 18% in the Conventional group at 12 months (p=0.2) and 18% vs 30.2% at 24 months (p=0.04, OR 3.47). Surgical recurrence rate at 24 months was 0% in the Kono group vs 4.6% in the Conventional group (p=0.3). At Kaplan‐Meyer analysis, patients with Kono‐S anastomosis presented a longer time until clinical recurrence than patients with side‐to‐side anastomosis (HR 0.36, p=0.037). At binary logistic regression, the Kono‐S anastomosis was the only variable significantly associated with the reduced risk of endoscopic recurrence (OR 0.19, p<0.001). With regard to postoperative outcomes, there were no differences in terms of surgery duration (p=0.8), time to flatus (p=0.4) or stool (p=0.8) and postoperative stay (p=0.3), as well post‐operative complications (p=0.6) and infections (p=0.7). [Table presented] Conclusions: This is the first RCT comparing Kono‐S anastomosis vs standard anastomosis in CD, which found a significant reduction of postoperative endoscopic recurrence rate by using the novel technique, without concerns about safety. The Kono‐S anastomosis could be considered the new recommended surgical technique in CD.

%Z Digestive and liver disease

Journal article; Conference proceeding

%U https://cochrane.66557.net/central/doi/10.1002/central/CN-02243916/full

%0 Journal Article

%A Tropeano, F. P.

%A Amendola, A.

%A De Palma, G. D.

%A Corcione, F.

%A Imperatore, N.

%A Castiglione, F.

%A Bucci, L.

%A Luglio, G.

%D 2020

%T Surgical prevention of anastomotic recurrence by excluding mesentery in Crohn's disease: the Supreme-CD study

%V 24

%N 4

%P 337

%8 2020-01-01

%R 10.1007/s10151-020-02154-4

%K *Crohn disease; *mesentery; Adult; Analysis of variance; Complication; Conference abstract; Controlled study; Female; Human; Human tissue; Major clinical study; Male; Operation duration; Patient referral; Postoperative care; Postoperative complication; Prevention; Randomized controlled trial; Recurrence risk; Risk assessment; Sample size; Side to side anastomosis; Surgery

%X Background Most patients with Crohn's disease (CD) experience disease recurrence after resection. Recently, a new antimesenteric, functional end‐to‐end, handsewn ileocolic anastomosis (Kono‐S) has been described, showing a significant reduction in endoscopic recurrence (ER) score and surgical recurrence (SR) rate. The aim of this trial was to provide randomized controlled data comparing Kono‐S anastomosis to stapled ileocolic side‐to‐side anastomosis. Methods A randomised controlled trial was conducted at a tertiary referral institution, enrolling and randomising (1:1) to undergo either the ''Kono group'' or the ''Conventional group'', consecutive CD patients needing surgery. The primary endpoint was ER (Rutgeerts score i2) at 6 months, while secondary endpoints were clinical recurrence (CR) at 12 and 24 months, ER at 18 months and SR at 24 months. Short‐term outcomes and postoperative complications were also recorded. Postoperative treatment was established on the basis of the patient's risk profile. A sample size of 70 patients (35 in each group) was considered necessary to demonstrate a reduction > 30% in ER at 6 months in the Kono group, when assuming a 60% ER expected rate in the control group. Chi squared, t test and ANOVA binary logistic regressions were performed. Results Seventy‐ nine CD patients were enrolled and randomized in Kono group (n = 36) or Conventional group (n = 43). At 6 months, 8 patients in the Kono group (22.2%) and 27 in the Conventional group (62.8%) had ER (p<0.01; OR 5.91) (mean Rutgeerts score at 6 months 0.92 + 1.05 and 2.06 + 1.31, respectively, p<0.01). Furthermore, severe postoperative ER (Rutgeerts score i3) was found in 13.8% of the Kono group vs 34.8% in the of Conventional group (p = 0.03; OR 3.32). The CR rate was 8% in the Kono group vs 18% in the Conventional group (p = 0.2) at 12 months and 18% vs 30.2% at 24 months (p = 0.04, OR 3.47). Only 2 patients in the Conventional group and none in the Kono group experienced a SR at 24 months (p = NS). At binary logistic regression, the Kono‐S anastomosis was the only variable significantly associated with the reduced risk of ER (OR 0.19, p<0.01). As regards postoperative outcomes, there were no differences between the two groups in terms of operating time, days to canalization, length of postoperative stay and complications (p = N.S.). Conclusions There was a significant reduction of the postoperative endoscopic recurrence rate with the novel technique, without concerns about safety. The Kono‐S anastomosis could be considered the new recommended surgical technique in CD.

%Z Techniques in coloproctology

Journal article; Conference proceeding

%U https://cochrane.66557.net/central/doi/10.1002/central/CN-02143956/full

%0 Journal Article

%A Luglio, G.

%A Rispo, A.

%A Tropeano, F. P.

%A Giglio, M.

%A Imperatore, N.

%A Cricri, M.

%A De Sire, R.

%A Castiglione, F.

%A Testa, A.

%A Guarino, A. D.

%A Et, Al.

%D 2023

%T SURGICAL PREVENTION OF ANASTOMOTIC RECURRENCE BY EXCLUDING MESENTERY IN CROHN'S DISEASE (THE SUPREMECD TRIAL): KONO-S ANASTOMOSIS REDUCES ENDOSCOPIC AND SURGICAL POST-OPERATIVE RECURRENCE IN CROHN'S DISEASE

%V 55

%P S93‐S94

%8 2023-01-01

%R 10.1016/S1590-8658(23)00261-X

%K *Crohn disease; *anastomosis; *mesentery; Adult; Conference abstract; Controlled study; Drug safety; Endoscopy; Female; Human; Major clinical study; Male; Outcome assessment; Outpatient; Prevention; Randomized controlled trial; Recurrence risk; Sample size; Surgery; Tertiary care center

%X Background and aim: Recently, a new antimesenteric, functional, end‐to‐end, hand‐sewn ileocolic anastomosis (Kono‐S) has shown a significant reduction in endoscopic recurrence (ER) and surgical recurrence (SR) rate in Crohn's disease (CD). This trial aimed to provide randomized controlled data comparing Kono‐S anastomosis and conventional stapled ileocolic side‐to‐side anastomosis. Materials and methods: Randomized controlled trial (RCT) at a tertiary referral centre. The primary endpoint: ER rate (Rutgeerts score >i2) after 6 months. Secondary endpoints: clinical recurrence (CR) rate after 24 and 36 months, and SR after 24, 36 and 60 months. Statistics was performed by using standard analyses. Furthermore, a sample size able to consent the detection of a reduction >30% in total ER (when assuming a 60% to 65% ER expected rate in the control group and a 30% in the case group) was calculated; to allow a 10% drop‐out of patients, 36 patients per group were needed (72 patients in the entire population). Results: A total of 119 CD patients were included in the study. In all, 51 (75%) ileocolic CD patients were randomized in Kono group and 68 in the conventional group. At 6 months, 17 (25%) in the Kono group and 51 patients (75%) in the control group presented an ER at endoscopy (p<0.001; OR: 6). A severe post‐operative ER (Rutgeerts score >i3) was found in 8 (15.7%) of Kono versus 23 (33.8%) CD patients in the conventional group (p<0.05; OR: 2.8). At 24 months, CR rate was 19.5% in the Kono group versus 30.9% in the conventional group (p=0.2; OR:1.6) while SR rate after 24 months was 0% in the Kono group versus 4.6% in the conventional group (p=0.2). At 36 months, CR rate was 19.6% in the Kono group and 33.8% in the conventional group (p=0.1; OR:1.7), while SR rate was 0% in Kono group versus 8.8% in the conventional group (p=0.03). Furthermore, when considering a 48‐months ad interim analysis of SR, the percentage of this outcome remained significantly lower in the Kono group (19.6% vs 30.9%; p<0.05; OR 1.8). Conclusions: This is the first RCT comparing Kono‐S anastomosis and standard anastomosis in CD. The results demonstrate a significant reduction in postoperative endoscopic, clinical and surgical recurrence rate for patients who underwent Kono‐S anastomosis, and no safety issues. ClinicalTrials.gov ID: NCT02631967.

%Z Digestive and liver disease

Journal article; Conference proceeding

%U https://cochrane.66557.net/central/doi/10.1002/central/CN-02543349/full

%0 Journal Article

%A Luglio, G.

%A Giglio, M. C.

%A Rispo, A.

%A Imperatore, N.

%A Testa, A.

%A Sollazzo, V.

%A Bucci, C.

%A Castiglione, F.

%A De Palma, G. D.

%A Bucci, L.

%D 2018

%T Supreme-CD study: surgical prevention of anastomotic recurrence by excluding mesentery in crohn'sdisease. preliminary results and trial protocol

%V 50

%N 2

%P e138

%8 2018-01-01

%K *mesentery; *preliminary data; *recurrence risk; Adult; Analysis of variance; Anastomosis leakage; Clinical assessment; Conference abstract; Controlled study; Female; Human; Human tissue; Major clinical study; Male; Morbidity; Randomized controlled trial; Sample size; Side to side anastomosis; Student; Surgery; Surgical patient

%X Background and aim: Kono‐S anastomosis after ileocolic resection for Crohn's Disease is a novel technique, supposed to reduce surgical recurrences. Aim of this ongoing trial is to provide randomized controlled data to compare Kono‐S anastomosis vs the stapled side‐to‐side anastomosis. Material and methods: All consecutive surgical patients with primary or recurrent ileocolic CD are being enrolled and randomised to undergo either the “Kono group” or the “Conventional group”. A sample size of 140 patients is considered necessary to demonstrate a 25% reduction in endoscopic recurrence at 6 months. Chi‐squared test will be used to analyse dichotomous variables, T‐Student and ANOVA for continuous variables. Trial primary endpoint: 6‐month endoscopic recurrence score and 5‐year surgical recurrence rate. Secondary end‐point: short‐term outcomes and postoperative complications. The trial has been approved by local Ethic Committee (n° 211/15) and registered on ClinicalTrial.gov (NCT02631967). Results: 46 patients have been enrolled to date; 26 received a Kono‐S anastomosis. No anastomotic leaks or major morbidity registered. Endoscopic 6‐month evaluation is available for 36 patients. Mean Rutgeerts score at 6 months was 3.2 in the “conventional group” and 0.8 in the “kono group” (p<0.001). Conclusions: Preliminary non‐randomised data on Kono‐S anastomosis are encouraging. The trial will assess if Kono‐S anastomosis is able to significantly reduce surgical recurrences after ileocolic resection for CD.

%Z Digestive and liver disease

Journal article; Conference proceeding

%U https://cochrane.66557.net/central/doi/10.1002/central/CN-01571675/full

%0 Journal Article

%A Luglio, G.

%A Giglio, M. C.

%A Rispo, A.

%A Peltrini, R.

%A Sacco, M.

%A Sollazzo, V.

%A Bucci, C.

%A Spadarella, E.

%A Terracciano, F.

%A De Palma, G. D.

%A Et, Al.

%D 2016

%T SuPREMe-CD study: surgical prevention of anastomotic recurrence by excluding mesentery in Crohn's disease-preliminary results and trial protocol

%V 10

%P S398

%8 2016-01-01

%R 10.1093/ecco-jcc/jjw019

%K *Crohn disease; *disease duration; *mesentery; *recurrence risk; Adult; Analysis of variance; Anastomosis leakage; Clinical trial; Control group; Controlled clinical trial; Controlled study; Data analysis software; Denervation; Devascularization; Feasibility study; Follow up; Human; Human tissue; Length of stay; Major clinical study; Model; Morbidity; Multivariate analysis; Patient referral; Prevention; Randomized controlled trial; Registration; Safety; Sample size; Side to side anastomosis; Student; Surgery; Young adult

%X Background: Around 70%‐80% of patients with Crohn's disease (CD) require surgeries in their lifetime. New drugs have reduced recurrences; nevertheless, the fact that disease in most of CD patients appear at the anastomotic site emphasises the importance of surgical approach. Anastomosis configuration has not been demonstrated as a risk factor for recurrence. Kono [1] described a new antimesenteric functional end‐to‐end hand‐sewn ileocolic anastomosis. They highlight how anastomotic recurrences always arise at the mesenteric side and speculate that factors such as devascularisation, denervation, and faecal stasis may have a causative role. The authors show a reduction in endoscopic surgical recurrence rate, but their data are not randomised, and the control group is represented by an historical cohort. The aim of this trial is to provide randomised controlled data to compare Kono anastomosis vs the stapled ileocolic side‐to‐side anastomosis. Methods: This is an ongoing randomised trial, performed at a tertiary referral institution. All consecutive patients (age 18‐75) with primary or recurrent ileocolic CD with an indication for surgery are being enrolled. Patients are being randomised to undergo either the 'Kono group' or the 'Conventional group', using a PC‐based randomised model. In total, 8 non‐randomised patients underwent Kono anastomosis before trial beginning to assess general safety and feasibility. A sample size of 140 patients is considered necessary to demonstrate a 25% reduction in endoscopic recurrence at 6‐month follow‐up. Chi‐squared test will be used to analyse dichotomous variables, and T‐Student and ANOVA will be used for continuous variables. Further univariate and multivariate analysis will be performed with SPSS software. The trial has been approved by the local Ethic Committee (n 211/15) and is under registration on ClinicalTrial. gov. Results: In the study, 8 non‐randomised patients underwent Kono anastomosis with no anastomotic leak or major morbidity. Median length of stay was 5 days. Endoscopic 6‐month evaluation showed no recurrence in 4 patients and a Rutgeerts 2 recurrence in 1 patient. Trial primary endpoint is the endoscopic recurrence score and surgical recurrence rate; endoscopic examination will be performed 6 months after surgery, and preliminary results will be revealed at 2‐year median follow‐up. Patients will be further follow‐up to 5 years. Secondary endpoint is the assessment of short‐term outcomes and postoperative complications. Conclusions: Preliminary non‐randomised data on Kono anastomosis are encouraging. Trial will assess if Kono anastomosis is able to significantly reduce endoscopic and surgical recurrences after ileocolic resection for CD.

%Z Journal of Crohn's & colitis

Journal article; Conference proceeding

%U https://cochrane.66557.net/central/doi/10.1002/central/CN-01732039/full

%0 Journal Article

%A Wright, E. K.

%A Kamm, M. A.

%A De Cruz, P.

%A Hamilton, A. L.

%A Ritchie, K.

%A Bell, S. J.

%A Brown, S. J.

%A Connell, W. R.

%A Desmond, P. V.

%A Liew, D.

%D 2015

%T Structured post-operative treatment and monitoring to prevent Crohn's disease recurrence is cost effective. Results from the POCER study

%V 30

%P 145

%8 2015-01-01

%R 10.1111/jgh.13094

%K *Australian; *gastroenterology; *monitoring; *recurrent disease; *surgery; Adalimumab; Algorithm; Arm; Australian; Calgranulin; Colonoscopy; Computer; Cost benefit analysis; Cost effectiveness analysis; Crohn disease; Drug monitoring; Drug therapy; Female; Health care; Health care cost; Health care utilization; Human; Intestine; Intestine resection; Medical record review; Mercaptopurine; Metronidazole; Patient; Questionnaire; Randomization; Remission; Risk; Tertiary care center; Therapy; algorithm; arm; colonoscopy; computer; cost benefit analysis; cost effectiveness analysis; drug monitoring; drug therapy; female; gastroenterology; health care; health care cost; health care utilization; human; intestine; intestine resection; medical record review; monitoring; patient; questionnaire; randomization; recurrent disease; remission; risk; surgery; tertiary care center; therapy

%X Background: Healthcare costs for Crohn's disease are high. Active disease, surgery, hospitalisations and anti‐TNF use are key cost drivers. 70% of patients with Crohn's disease require at least one surgical resection, and of these most develop disease recurrence. Post‐operative strategies to prevent disease recurrence, which include endoscopic assessment and patient‐tailored prophylactic drug therapy, are therefore desirable. However, the cost‐effectiveness of such strategies is unknown. Methods: In a randomised trial, patients undergoing intestinal resection of all macroscopically diseased bowel were treated with post‐operative drug therapy to prevent disease recurrence. All patients received 3 months of metronidazole therapy. Patients at high risk of recurrence also received a thiopurine, or adalimumab if they were intolerant to thiopurines. Patients were randomly assigned to parallel groups: colonoscopy at 6 months (active care) or no colonoscopy (standard care). Computer‐generated block randomisation was used to allocate patients to active or standard care in a 2:1 ratio. For endoscopic recurrence (Rutgeerts score ≥ i2) at 6 months patients stepped‐up to thiopurine, fortnightly adalimumab with thiopurine, or weekly adalimumab. Endoscopic recurrence was assessed at the study endpoint of 18 months. Faecal calprotectin (FC) measurement was performed post‐operatively at 6, 12 and 18 months. Healthcare utilization data were collected post‐operatively over 18 months, based on administrative data maintained by the Clinical Costing Unit at one secondary and tertiary hospital, chart review and patient questionnaires. Unit costs were based on published Australian health care sources. Results: 174 patients (median age 38, 55% female) were enrolled. Of these, 60 patients (active care n = 43, standard care n = 17) from 1 centre were included in this anaysis. Median total health care cost was $6440 per patient. Active care cost $4824 more than standard care over 18 months. Medication accounted for 78% of total cost, of which 90% was for adalimumab. Median health care cost was higher for those with endoscopic recurrence compared to those in remission ($26,347 [IQR 25,045‐27,485] vs $2,729 [IQR 1,182‐5,215], P < 0.001). FC to select patients for colonoscopy could reduce cost by $1010 per patient over 18 months. Active care was associated with 18% decreased endoscopic recurrence, costing $861 for each recurrence prevented. Conclusion: Post‐operative strategies to prevent disease recurrence after intestinal resection are associated with high healthcare costs, the majority of which is medication driven. Endoscopic recurrence when compared to remission is associated with significantly higher healthcare costs. The POCER strategy, as illustrated in the active care arm of this study, is based on risk‐related medication, monitoring for early disease recurrence, and treatment intensification when needed. It is associated with a reduction in post‐operative endoscopic recurrence without a significantly greater cost than standard care. Strategies that prevent endoscopic recurrence may therefore be associated with substantial healthcare savings downstream, making the POCER algorithm a potentially cost effective strategy. Further, using FC to select patients appropriate for colonoscopy reduces costs significantly. The long term cost‐benefit of these strategies remains to be evaluated.

%Z Journal of gastroenterology and hepatology (australia)

Journal article; Conference proceeding

%U https://cochrane.66557.net/central/doi/10.1002/central/CN-01136408/full

%0 Journal Article

%A NCT

%D 2022

%T Stress in Inflammatory Bowel Disease

%8 2022-01-01

%K Inflammatory Bowel Diseases; Intestinal Diseases

%X Similar to other chronic stressors, diagnosis with a chronic illness places youth at risk of adverse psychosocial outcomes. Inflammatory bowel diseases (IBD), Crohn's disease, ulcerative colitis, and indeterminate colitis are chronic, immune‐mediated diseases of the gastrointestinal tract characterized by unpredictable remissions of disease activity followed by relapses of symptoms. Although some research has found higher levels of disease activity to relate to greater depressive symptoms, the overall relationship between disease activity and emotional functioning has been mixed, suggesting that additional individual differences need to be considered in addition to illness‐related factors when predicting emotional outcomes. Increased risk for developing anxiety disorders and depression has been documented in youth with IBD. Individual differences in physiological reactivity may affect patients' risk for developing psychosocial difficulties within the context of chronic stress. Additional risk factors for the development of psychosocial difficulties need to be identified to identify moderators of outcomes above and beyond disease activity. Individual differences in physiological reactivity may affect patients' risk for developing psychosocial difficulties within the context of chronic stress. Physiological reactivity, which broadly refers to bodily reactions in response to a stressor, varies with regards to intensity and threshold for activation between individuals. In youth affected by non‐medical chronic stress (e.g., family conflict, trauma history), measures of autonomic dysfunction have been used to explain why some individuals have worse psychological and physical outcomes compared to others exposed to similar levels of chronic stress. Results support autonomic dysfunction as a vulnerability factor for adjustment problems within the context of chronic environmental stress. The aim of the current study is to test whether differences in psychophysiological reactivity serve as risk factors in the relationship between clinical disease activity in youth newly diagnosed with IBD and psychosocial adjustment problems. The relationship between psychophysiological reactivity and psychosocial adjustment problems in youth with IBD will be compared to healthy controls. Youth participants with IBD will be enrolled in a coping skills treatment to test the effectiveness of a cognitive‐behavioral intervention including biofeedback to reduce anxiety and depression and disease symptoms. The research team will conduct a pilot intervention targeting autonomic dysfunction through biofeedback enhanced coping skills treatment delivered virtually over 6‐sessions.

%Z https://clinicaltrials.gov/show/NCT05202418

Trial registry record

%U https://cochrane.66557.net/central/doi/10.1002/central/CN-02367074/full

%0 Journal Article

%A De Cruz, P.

%A Kamm, M.

%A Hamilton, A.

%A Ritchie, K.

%A Krejany, S.

%A Gorelik, A.

%A Liew, D.

%A Prideaux, L.

%A Lawrance, I.

%A Andrews, J.

%A Et, Al.

%D 2013

%T Strategic timing of anti-TNF therapy in postoperative Crohn's disease: comparison of routine use immediately postoperatively with selective use after demonstrated recurrence at 6 month endoscopy. Results from POCER

%V 28

%P 92

%8 2013-01-01

%R 10.1111/jgh.12365-6

%K *Crohn disease; *endoscopy; *gastroenterology; *therapy; Adalimumab; Arm; Azathioprine; Crohn disease; Healing; High risk patient; Human; Intention to treat analysis; Mercaptopurine; Metronidazole; Patient; Recurrent disease; Remission; Risk; Smoking; Surgery; arm; endoscopy; gastroenterology; healing; high risk patient; human; intention to treat analysis; patient; recurrent disease; remission; risk; smoking; surgery; therapy

%X Introduction: Recent data suggest that anti‐TNF therapy prevents postoperative recurrence of Crohn's disease. Routine postoperative use is costly and may lead to overtreatment; an alternative may be selective use for early endoscopic recurrence. These strategies have been compared with mucosal healing as the goal in this “treat to target” study. Methods: In the Post Operative Crohn's Endoscopic Recurrence, “POCER” study, after resection patients were stratified for risk of recurrence as high (smoker, perforating disease, ≥2nd operation) or low risk. All patients received 3 months metronidazole 400 mg bd. High risk patients also received daily azathioprine 2 mg/kg or 6 mercaptopurine 1.5 mg/kg, or adalimumab 40 mg fortnightly if thiopurine intolerant. Patients were randomised to endoscopy at 6 months or no endoscopy; endoscopic remission defined as Rutgeerts score i0 or i1 & recurrence as ≥i2. For endoscopic recurrence at 6 months high risk patients on thiopurine stepped up to adalimumab 40 mg fortnightly, & high risk thiopurine‐intolerant patients stepped up to weekly adalimumab. All patients were colonoscoped at 18 months: the primary endpoint endoscopic recurrence. In this intent‐to‐treat sub‐analysis we compared the 18 month outcomes of high risk patients in the endoscopy arm on immediate postoperative adalimumab to those initially on thiopurine who stepped‐up to combined adalimumab + thiopurine for recurrence at 6 months. Results: Endoscopic recurrence at 18 months occurred in 12/28 (43%) patients on adalimumab immediately postoperatively compared to 19/32 (59%) patients initially on thiopurine who stepped up to adalimumab + thiopurine at 6 months; P = 0.20. Complete mucosal normality (i0) occurred in 9/28 (32%) v 7/32 (22%); P = 0.37. Severe disease (i3 & i4) occurred in 3/28 (11%) v 3/32 (9%); P = NS. Conclusions: Recurrence did not differ significantly between immediate postoperative adalimumab & step‐up adalimumab at 6 months for endoscopic recurrence, although the former tended to be more effective. There was minimal difference in rates of severe disease recurrence between the two strategies. Step‐up with anti‐TNF therapy based on endoscopic findings is a viable postoperative strategy in patients at high risk of recurrence.

%Z Journal of gastroenterology and hepatology

Journal article; Conference proceeding

%U https://cochrane.66557.net/central/doi/10.1002/central/CN-01024469/full

%0 Journal Article

%A Brignola, C.

%A De Simone, G.

%A Belloli, C.

%A Iannone, P.

%A Belluzzi, A.

%A Gionchetti, P.

%A Campieri, M.

%A Barbara, L.

%D 1994

%T Steroid treatment in active Crohn's disease: a comparison between two regimens of different duration

%V 8

%N 4

%P 465‐468

%8 1994-01-01

%R 10.1111/j.1365-2036.1994.tb00316.x

%K Adult; Crohn Disease [*drug therapy]; Drug Administration Schedule; Female; Humans; Life Tables; Methylprednisolone [*administration & dosage]; Remission Induction; Risk Factors

%X BACKGROUND: Steroids are highly effective in active Crohn's disease; clinical relapse following steroid withdrawal, however, is frequent. We used two steroid regimens of different duration in order to compare their efficacy in inducing and maintaining clinical remission. METHODS: Seventy patients with active Crohn's disease were treated with methylprednisolone 40 mg/day i.m. for 3 weeks and then with two different regimens of tapering dosage: one for a further 4 weeks and another for a further 12 weeks. RESULTS: Steroid therapy induced remission within 3 weeks in 91% of the whole group of patients; at the end of each protocol remission rates were 85% of patients in the group treated for the shorter period and 87% of those treated for the longer period (difference 2%, CI = ‐14 to 18, P = NS); remission rates within 6 months after stopping steroids were 53% and 37% respectively (difference 16%, CI = ‐9 to 41, P = NS). CONCLUSIONS: No significant differences were found between the two regimens. Multiple courses of steroid treatment in the previous 3 years and a short time interval following previous steroid treatment seem to be risk factors for relapse.

%Z Alimentary pharmacology & therapeutics

Journal article

%U https://cochrane.66557.net/central/doi/10.1002/central/CN-00107622/full

%0 Journal Article

%A Logan, R. F.

%D 1999

%T Smoking, use of oral contraceptives, and medical induction of remission were risk factors for relapse in Crohn's disease

%V 44

%N 3

%P 311‐312

%8 1999-01-01

%R 10.1136/gut.44.3.311

%K Aged; Aged, 80 and over; Contraceptives, Oral [adverse effects]; Crohn Disease [drug therapy, *etiology, surgery]; Humans; Recurrence; Smoking [adverse effects]

%Z Gut

Journal article

%U https://cochrane.66557.net/central/doi/10.1002/central/CN-00337906/full

%0 Journal Article

%A McGovern, D. P.

%A Travis, S. P.

%D 2005

%T Smoking status in therapeutic trials in Crohn's disease

%V 54

%N 7

%P 1047‐1048

%8 2005-01-01

%R 10.1136/gut.2005.065789

%K *Crohn disease /drug therapy; *smoking habit; Clinical trial; Controlled clinical trial; Corticosteroid therapy; Crohn Disease [*therapy]; Disease severity; Human; Humans; Letter; Priority journal; Randomized Controlled Trials as Topic [methods]; Randomized controlled trial; Relapse; Research Design; Risk factor; Smoking [*adverse effects]

%Z Gut

Journal article

%U https://cochrane.66557.net/central/doi/10.1002/central/CN-01730778/full

%0 Journal Article

%A De Cruz, P.

%A Kamm, M.

%A Hamilton, A.

%A Ritchie, K.

%A Krejany, S.

%A Gorelik, A.

%A Liew, D.

%A Prideaux, L.

%A Lawrance, I.

%A Andrews, J.

%A Et, Al.

%D 2013

%T Smoking is the key risk factor that doubles the risk of postoperative recurrence of Crohn's disease despite preventive drug treatment. Results from the POCER study

%V 28

%P 92

%8 2013-01-01

%R 10.1111/jgh.12365-6

%K *Crohn disease; *drug therapy; *gastroenterology; *risk; *risk factor; *smoking; Adalimumab; Arm; Azathioprine; Cohort analysis; Colonoscopy; Crohn disease; High risk patient; Human; Mercaptopurine; Metronidazole; Patient; Prophylaxis; Recurrent disease; Remission; Surgery; arm; cohort analysis; colonoscopy; drug therapy; gastroenterology; high risk patient; human; patient; prophylaxis; recurrent disease; remission; risk; risk factor; smoking; surgery

%X Introduction: Smoking, perforating disease and previous resection have been identified individually from retrospective cohort studies as factors associated with increased risk of earlier post‐operative Crohn's disease recurrence.We assessed prospectively whether these factors are associated with increased recurrence in the setting of optimal drug therapy. Methods: In this Post Operative Crohn's Endoscopic Recurrence “POCER” study, after resection patients were stratified as high (smoker, perforating disease, ≥2nd operation) or low risk. All patients received 3 months metronidazole 400 mg bd. High risk patients also received daily azathioprine 2 mg/kg or 6 mercaptopurine 1.5 mg/kg. High risk patients intolerant of thiopurine received adalimumab 40 mg fortnightly. Patients were randomised 2:1 to colonoscopy at 6 months (“active care”) or no colonoscopy (“standard care”). Endoscopic remission was defined as Rutgeerts score i0 or i1 and recurrence as ≥i2. For endoscopic recurrence at 6 months low risk patients stepped up to thiopurine, high risk patients on thiopurine stepped up to adalimumab 40 mg fortnightly, and high risk thiopurine‐intolerant patients stepped up to weekly adalimumab. All patients were colonoscoped at 18 months, with primary end‐point endoscopic recurrence at 18 months. Results: 145 high risk (median age 38 years) & 29 low risk patients (median 36 years) were studied. Of high risk patients: 55 (38%) were current smokers, 101 (70%) had perforating disease and 50 (34%) had ≥1 prior resection. Smokers had an OR of 2.2 (95% CI 1.1‐4.2; P = 0.02) for recurrence at 18 months. Recurrence occurred in 24/38 (63%) of smokers in the active and 13/17 (76%) of smokers in the standard care arms (P = 0.33). The OR for recurrence for previous resection and penetrating disease were 1.9 (1.0‐3.8; P = 0.06) and 0.9 (0.5‐1.7; P = 0.79) respectively. The OR for recurrence if >1 one risk factors was present was 2.8 (1.2‐7.0; P = 0.023) compared to 0 risk factors. Conclusions: Despite optimised drug therapy smoking doubles the risk of Crohn's disease recurrence short term. The risk increases when more than one risk factor is present. These prospectively validated risk factors identify patients in whom more intensive preventive therapy may be warranted. Smoking should be discouraged vigorously.

%Z Journal of gastroenterology and hepatology

Journal article; Conference proceeding

%U https://cochrane.66557.net/central/doi/10.1002/central/CN-01024468/full

%0 Journal Article

%A Szigethy, E.

%A Hashash, J.

%A Vachon, A.

%A Mcauliff, K.

%A Strassburger, M.

%A Rode, N.

%A Keljo, D.

%A Fabio, A.

%A Regueiro, M.

%A Binion, D.

%A Et, Al.

%D 2016

%T Sleep disturbance and fatigue in adolescents and young adults with Crohn's disease: characterization and comparison to healthy controls

%V 22

%P S22

%8 2016-01-01

%R 10.1097/01.MIB.0000480086.95220.03

%K *Crohn disease; *adolescent; *colitis; *fatigue; *human; *inflammatory bowel disease; *non profit organization; *sleep disorder; *young adult; Actimetry; Adult; Adulthood; Anxiety; Anxiety disorder; Cannabis; Caucasian; Control group; Correlation analysis; Crohn disease; Disease activity; Drug therapy; Female; Fisher exact test; Gender; Hamilton Depression Rating Scale; Hospital; Inflammation; Interview; Male; Marker; Morbidity; Patient; Pittsburgh Sleep Quality Index; Posttraumatic stress disorder; Psychiatric diagnosis; Questionnaire; Relapse; Risk factor; Sleep; Student t test; Substance abuse; actimetry; adolescent; adult; adulthood; anxiety; anxiety disorder; colitis; control group; correlation analysis; disease activity; drug therapy; fatigue; female; gender; hospital; human; inflammation; inflammatory bowel disease; interview; male; morbidity; non profit organization; patient; posttraumatic stress disorder; psychiatric diagnosis; questionnaire; relapse; risk factor; sleep; sleep disorder; substance abuse; young adult

%X Background: Sleep disturbance and fatigue are common in patients with IBD. Sleep disturbance has recently been linked to inflammation and may be a risk factor for Crohn's disease (CD) relapse. Sleep is particularly critical during early adulthood; little is known about sleep disturbance in young IBD cohorts. This study evaluated sleep disturbance in adolescents and young adults with CD compared to healthy matched controls. Methods: Over 18 months, we screened all CD subjects ages 15 to 30 using the Pittsburgh Sleep Quality Index (PSQI) and Multidimensional Fatigue Inventory (MFI) at pediatric or adult GI clinic appointments. Participants scoring ≥7 on PSQI and ≥45 on MFI completed a further assessment, which included further probes of sleep using subjective (questionnaires) and objective (actigraphy) measures, a structured psychiatric interview for DSM‐IV (SCID) for psychiatric diagnoses, anxiety and depression severity (Hamilton Rating Scale; HRSA and HRSD), CD activity (Harvey Bradshaw [HB]), and demographic information. Further details about IBD course, and medications were obtained by blinded investigators (J.G. H. and A.V.). Labs were drawn to assess inflammatory markers. Healthy controls in the same age range were screened for mental and physical conditions. Subjects with CD were compared to healthy controls using independent t‐test for continuous variables and chi square (Fisher exact test) for categorical variables. Correlation analyses were used to further characterize clinical attributes of sleep disturbance and fatigue in both groups. Results: Over 18 months, 224 CD subjects were approached, 193 were screened, 93 screened positive on the PSQI and MFI with 51 completing the more comprehensive assessment. An additional 21 subjects met PSQI but not MFI criteria CD subjects were predominantly Caucasian, 63% female, with mean age of 23.5. Thirty healthy controls were assessed and matched on these variables. In the CD cohort, mean CD activity was mild, 24% were depressed, 57% had an anxiety disorder; 8% had post‐traumatic stress disorder, and 2% had substance abuse (marijuana). Of the total CD sample, 42% met PSQI criteria for sleep disturbance and fatigue and 59% for sleep disturbance alone. Compared to healthy controls, the CD group had significantly greater sleep disturbance (PSQI 3.2 versus 10.8), fatigue (MFI 33.2 versus 64.6) and significantly greater anxiety and depression severity. The PSQI significantly correlated with MFI (r = 0.29) and HB (r = 0.24) in the CD group and MFI (r = 0.4) in the control group. The MFI significantly correlated HB (r = 0.48), PSQI (r = 0.29), HRSD (r = 0.38; minus sleep item), HRSA (r = 0.23; minus sleep item), and gender (r = 0.22, with females . males) in the CD group and PSQI (r = 0.4) HRSA (r = 0.39; minus sleep item), HRSD (r = 0.37; minus sleep item), and age (r = 0.28) in the control group. Conclusions: Adolescents and young adults with CD have higher rates of sleep disturbance and fatigue than normal controls. In the CD cohort, sleep disturbance and fatigue are significantly associated with higher CD activity with fatigue also being associated with depression and anxiety and being female. Future studies need to address if sleep interventions can improve fatigue, disease activity and psychiatric morbidity in young patients with IBD.

%Z Inflammatory bowel diseases

Journal article; Conference proceeding

%U https://cochrane.66557.net/central/doi/10.1002/central/CN-01171823/full

%0 Journal Article

%A Boivineau, L.

%A Bourgaux, J. F.

%A Pineton De Chambrun, G.

%A Caillo, L.

%A Danan, G.

%A Boitard, J.

%A Liautard, J.

%A Guillon, F.

%A Altwegg, R.

%D 2017

%T Serum adalimumab concentration 3 months after surgery is correlated with endoscopic recurrence in Crohn's disease patients treated with adalimumab for prevention of postoperative recurrence

%V 11

%N Supplement 1

%P S47‐S48

%8 2017-01-01

%R 10.1093/ecco-jcc/jjx002.071

%K *Crohn disease; *adalimumab; *recurrence risk; Adult; Anastomosis; Area under the curve; Clinical article; Conference abstract; Controlled clinical trial; Controlled study; Crohn disease; Diagnostic test accuracy study; Drug therapy; Endogenous compound; Female; Human; Human tissue; Ileum disease; Male; Monotherapy; Multicenter study; Phenotype; Practice guideline; Prevention; Prospective study; Receiver operating characteristic; Risk factor; Surgery; Tumor necrosis factor; Young adult; adult; anastomosis; area under the curve; clinical article; conference abstract; controlled clinical trial; controlled study; diagnostic test accuracy study; drug therapy; female; human; human tissue; ileum disease; male; monotherapy; multicenter study; phenotype; practice guideline; prevention; prospective study; receiver operating characteristic; recurrence risk; risk factor; surgery; young adult

%X Background: Despite the use of anti‐TNF therapy for prevention of postoperative recurrence, more than 20% of Crohn's disease (CD) patients present an endoscopic recurrence 6 months after surgery. The aim of our study was to assess the relationship between serum adalimumab (ADA) concentration 3 months after surgery and en‐doscopic recurrence at 6 months in CD patients treated with adali‐mumab for prevention of postoperative recurrence. Methods: This was a prospective, multicenter study between January 2014 and March 2016. All CD patients who underwent an ileocecal resection with ileocolonic anastomosis and were treated with ADA monotherapy (introduced one month after surgery subcutaneously with 160 mg at week 0, 80 mg at week 2 and then 40 mg every week), for prevention of postoperative recurrence were included in the study. Serum ADA concentration was determined 2 months after ADA introduction. Endoscopic recurrence at 6 months was defined as a Rutgeerts endoscopic score ≥ i2. Results: Fifteen CD patients were included (9 male and 6 female), with a median age of 24 years‐old [15‐47]. Disease phenotype was considered as penetrating in 8 patients (53%), stricturing in 5 pa‐tients (34%) and inflammatory in 2 (13%). Eight (53%) patients had an isolated ileal disease and 7 (47%) an ileocolonic location. Eight patients (53%) had at least 2 risk factors for postoperative recurrence according to ECCO guidelines and 14 (93%) were already treated with anti‐TNF before surgery. The overall endoscopic recurrence rate was 46.7%. The median serum ADA concentration 3 months after surgery was 7.9 μg/mL (0.04‐11.8) and no patient had anti‐ADA antibodies. Median serum ADA concentration was significantly higher in CD patients without endoscopic postoperative recurrence compared to CD patients with endoscopic postoperative recurrence (8.2 μg/mL vs 2.5 μg/mL, p=0.121). According to Rutgeerts score, the median serum ADA concentration was 8.2, 5.1 and 0.2 mg/mL in patients with a score ≤ i1, i2 and ≥i3, respectively (p=0.033). Indeed, the serum ADA concentration was inversely correlated to the Rutgeerts score (Pearson coefficient =‐0.61, p=0.015). The ROC curve analysis demonstrated a specificity of 71% and a sensitivity of 87% for serum ADA concentration threshold of 4 μg/mL to predict endoscopic recurrence (area under the curve = 0.75±0.14). Thus, 83% of CD patients with a serum ADA concentration <4 μg/mL had an endoscopic recurrence, compared to 22% of CD patients with a serum ADA concentration >4μg/mL (p=0.04). Conclusions: In our study, there was a significant correlation between serum ADA concentration 3 months after surgery and endo‐scopic postoperative recurrence at 6 months in CD patients treated with ADA to prevent postoperative recurrence.

%Z Journal of Crohn's & colitis

Journal article; Conference proceeding

%U https://cochrane.66557.net/central/doi/10.1002/central/CN-01469783/full

%0 Journal Article

%A Hamilton, A. L.

%A Kamm, M. A.

%A De Cruz, P.

%A Wright, E. K.

%A Selvaraj, F.

%A Princen, F.

%A Gorelik, A.

%A Liew, D.

%A Lawrance, I. C.

%A Andrews, J. M.

%A Et, Al.

%D 2017

%T Serologic antibodies in relation to outcome in postoperative Crohn's disease

%V 32

%N 6

%P 1195‐1203

%8 2017-01-01

%R 10.1111/jgh.13677

%K *Crohn disease /drug therapy /drug therapy /surgery; *outcome assessment; Adult; Antibodies, Antineutrophil Cytoplasmic [blood]; Antibodies, Bacterial [blood]; Antibody titer; Article; Biomarkers [blood]; Colonoscopy; Controlled study; Crohn Disease [*diagnosis, *surgery]; Female; Human; Humans; Immunoglobulin A [blood]; Immunoglobulin G [blood]; Low risk patient; Major clinical study; Male; Middle Aged; Multicenter Studies as Topic; Multicenter study; Perioperative Period; Phenotype; Porins [immunology]; Prediction; Predictive value; Priority journal; Prospective Studies; Prospective study; Randomized Controlled Trials as Topic; Randomized controlled trial; Recurrence; Recurrent disease; Remission; Risk; Risk factor; Saccharomyces cerevisiae [immunology]; Sensitivity and specificity; Smoking; Smoking [adverse effects]

%X Background and Aim: Disease recurs frequently after Crohn's disease resection. The role of serological antimicrobial antibodies in predicting recurrence or as a marker of recurrence has not been well defined. Methods: A total of 169 patients (523 samples) were prospectively studied, with testing peri‐operatively, and 6, 12 and 18 months postoperatively. Colonoscopy was performed at 18 months postoperatively. Serologic antibody presence (perinuclear anti‐neutrophil cytoplasmic antibody [pANCA], anti‐Saccharomyces cerevisiae antibodies [ASCA] IgA/IgG, anti‐OmpC, anti‐CBir1, anti‐A4‐Fla2, anti‐Fla‐X) and titer were tested. Quartile sum score (range 6–24), logistic regression analysis, and correlation with phenotype, smoking status, and endoscopic outcome were assessed. Results: Patients with ≥ 2 previous resections were more likely to be anti‐OmpC positive (94% vs 55%, ≥ 2 vs < 2, P = 0.001). Recurrence at 18 months was associated with anti‐Fla‐X positivity at baseline (49% vs 29%; positive vs negative, P = 0.033) and 12 months (52% vs 31%, P = 0.04). Patients positive (n = 28) for all four antibacterial antibodies (anti‐CBir1, anti‐OmpC, anti‐A4‐Fla2, and anti‐Fla‐X) at baseline were more likely to experience recurrence at 18 months than patients negative (n = 32) for all four antibodies (82% vs 18%, P = 0.034; odds ratio 6.4, 95% confidence interval 1.16–34.9). The baseline quartile sum score for all six antimicrobial antibodies was higher in patients with severe recurrence (Rutgeert's i3‐i4) at 18 months, adjusted for clinical risk factors (odds ratio 1.16, 95% confidence interval 1.01–1.34, P = 0.039). Smoking affected antibody status. Conclusions: Anti‐Fla‐X and presence of all anti‐bacterial antibodies identifies patients at higher risk of early postoperative Crohn's disease recurrence. Serologic screening pre‐operatively may help identify patients at increased risk of recurrence.

%Z Journal of gastroenterology and hepatology

Journal article

%U https://cochrane.66557.net/central/doi/10.1002/central/CN-01729364/full

%0 Journal Article

%A Yamamoto, T.

%A Shimoyama, T.

%A Umegae, S.

%A Matsumoto, K.

%D 2016

%T Serial monitoring of faecal calprotectin for the assessment of endoscopic recurrence in asymptomatic patients after ileocolonic resection for Crohn's disease: a long-term prospective study

%V 9

%N 5

%P 664‐670

%8 2016-01-01

%R 10.1177/1756283X16646562

%K *Crohn disease; *Crohn disease/di [Diagnosis]; *Crohn disease/su [Surgery]; *asymptomatic disease; *calgranulin; *calgranulin/ec [Endogenous Compound]; *colon resection; *colonoscopy; *feces level; *ileum; *monitoring; *prospective study; *recurrent disease/di [Diagnosis]; *relapse; Adult; Article; Clinical article; Controlled clinical trial; Controlled study; Crohn disease [diagnosis, surgery]; Diagnosis; Diagnostic accuracy; Diagnostic test accuracy study; Digestive system disease assessment; Female; Follow up; Gene expression; Human; Incidence; Male; Predictive value; Priority journal; Prospective study; Remission; Rutgeerts score; Scoring system; Sensitivity and specificity; adult; article; asymptomatic disease; clinical article; colectomy; colonoscopy; controlled clinical trial; controlled study; diagnostic accuracy; diagnostic test accuracy study; digestive system disease assessment; feces level; female; follow up; human; ileum; incidence; male; predictive value; priority journal; prospective study; recurrent disease [diagnosis]; remission; scoring system; sensitivity and specificity

%X Background: It is recommended that ileocolonoscopy is performed within 1 year after resection for Crohn's disease (CD). Nevertheless, optimal monitoring strategies for recurrence after the ileocolonoscopy remain to be elucidated. This prospective study was to evaluate the value of serial monitoring of faecal calprotectin (FC) after ileocolonoscopy for the assessment of endoscopic recurrence in asymptomatic patients. Methods: Patients in clinical remission who had no endoscopic recurrence at ileocolonoscopy 6‐12 months after ileocolonic resection were studied. FC levels were measured every 2 months up to 24 months after the ileocolonoscopy. When the FC level was elevated (>3/4140 μg/g), a second ileocolonoscopy was immediately undertaken. In contrast, patients who maintained low FC levels (<140 μg/g) during the 24‐month follow up underwent a second ileocolonoscopy at the end of the study. Endoscopic recurrence was defined as a Rutgeerts score >3/4i2. Results: A total of 30 patients were studied. In eight patients, the FC level was raised during the 24‐month follow up. Six of the eight patients (75%) had endoscopic recurrence. Of 22 patients who maintained low FC levels, 20 (91%) had no endoscopic recurrence, whereas two showed endoscopic recurrence at the end of the follow up. The incidence of endoscopic recurrence was significantly higher in patients with elevation of FC levels versus those with maintained low FC levels (75% versus 9%). A cut‐off value of 140 μg/g for FC had a sensitivity of 75%, a specificity of 91%, a positive predictive value of 75%, a negative predictive value of 91% and a diagnostic accuracy of 87% to detect endoscopic recurrence. Conclusions: Consecutive monitoring of FC is useful for the assessment of endoscopic recurrence after the initial ileocolonoscopy. Increased FC levels indicate a need for repeat ileocolonoscopy, while sustained low FC levels predict a low risk of endoscopic recurrence. In patients maintaining low FC levels, unnecessary invasive endoscopic examinations can be avoided.

%Z Therapeutic advances in gastroenterology

Journal article

%U https://cochrane.66557.net/central/doi/10.1002/central/CN-01196112/full

%0 Journal Article

%A EUCTR PL

%D 2018

%T Risk-stratified randomized controlled trial in paediatric Crohn’s Disease: methotrexate versus azathioprine or adalimumab for maintaining remission in patients at low or at high risk for aggressive disease course, respectively – a treatment strategy

%8 2018-01-01

%X INTERVENTION: Trade Name: Humira Product Name: HUMIRA Pharmaceutical Form: Solution for infusion INN or Proposed INN: ADALIMUMAB CAS Number: 331731‐18‐1 Concentration unit: mg milligram(s) Concentration type: equal Concentration number: 40‐ Trade Name: Azathioprine Pharmaceutical Form: Coated tablet INN or Proposed INN: Azathioprine CAS Number: 446‐86‐6 Concentration unit: mg/g milligram(s)/gram Concentration type: equal Concentration number: 50‐ Trade Name: Methotrexate Product Name: Methotrexate Pharmaceutical Form: Solution for injection INN or Proposed INN: METHOTREXATE CAS Number: 59‐05‐2 Other descriptive name: METHOTREXATE Concentration unit: mg/ml milligram(s)/millilitre Concentration type: equal Concentration number: 50‐ CONDITION: Crohn's disease Therapeutic area: Diseases [C] ‐ Digestive System Diseases [C06] PRIMARY OUTCOME: Main Objective: to complare the effectiveness of weekly subcutaneouslly administered MTX fro mainteining relapse‐ free sustained steroid/EN ‐ free 1 year remission compared with:; ‐ daily oral AZA/EMP in low ris paediatric CD; ‐ subcutaeously administered adalimumab in high risk paediatric CD Primary end point(s): complaring the two treatment arms per group for sustained steroid/EN free remission at Month 12, where sustained remission is defined as wPCDAI =12.5 and CRP =1,5 fold the normal upper limit without a relapse since week 12. Secondary Objective: compparison between the two treatment arms per risk group (high ris or low risk for aggresive disease evolution) (and inter‐risk paediatric CD) Timepoint(s) of evaluation of this end point: At months 12 SECONDARY OUTCOME: Secondary end point(s): compparing the two traeatment arms per risk group and comparing methotrexate treatment between high and low risk group Timepoint(s) of evaluation of this end point: at month 12 INCLUSION CRITERIA: • Children 6‐17, with a new‐onset CD diagnosed < 6 months using established criteria (28, 29), requiring a steroid‐based or EN based induction therapy • At initial diagnosis, wPCDAI >40 or CRP>2 times upper limit at diagnosis • all wPCDAI scores (0‐120) are possible at inclusion (patients in remission and patients with active disease) • Luminal active CD (B1) with or without B2 and/or B3 disease behavior • Initial exposure to 5‐ASA and derivate is tolerated • Exposure to antibiotics is tolerated • If one of the following criteria is present, patients are allocated to the high risk group prior randomization: • Complex fistulizing perianal disease • Panenteric disease phenotype (defined as L3 with L4b per Paris classification or L3 with deep ulcers in duodenum, stomach or oesophagus (not HP‐ or NSAID‐related)) • Severe growth impairment (height z‐score <‐2 or crossing 2 percentiles or more) likely related to CD • Significant hypoalb

%Z https://trialsearch.who.int/Trial2.aspx?TrialID=EUCTR2016-000522-18-PL

Trial registry record

%U https://cochrane.66557.net/central/doi/10.1002/central/CN-01906420/full

%0 Journal Article

%A Mällinen, J.

%A Rautio, T.

%A Grönroos, J.

%A Rantanen, T.

%A Nordström, P.

%A Savolainen, H.

%A Ohtonen, P.

%A Hurme, S.

%A Salminen, P.

%D 2019

%T Risk of Appendiceal Neoplasm in Periappendicular Abscess in Patients Treated With Interval Appendectomy vs Follow-up With Magnetic Resonance Imaging: 1-Year Outcomes of the Peri-Appendicitis Acuta Randomized Clinical Trial

%V 154

%N 3

%P 200‐207

%8 2019-01-01

%R 10.1001/jamasurg.2018.4373

%K Abscess [*diagnostic imaging, *therapy]; Adolescent; Adult; Appendectomy; Appendiceal Neoplasms [*diagnostic imaging, *epidemiology]; Appendicitis [*diagnostic imaging, *therapy]; Colonoscopy; Conservative Treatment; Female; Finland [epidemiology]; Humans; Incidence; Magnetic Resonance Imaging [*methods]; Male; Middle Aged; Risk; Tomography, X‐Ray Computed

%X Importance: The step after conservative treatment of periappendicular abscess arouses controversy, ranging from recommendations to abandon interval appendectomy based on low recurrence rates of the precipitating diagnosis to performing routine interval appendectomy owing to novel findings of increased neoplasm risk at interval appendectomy. To our knowledge, there are no randomized clinical trials with sufficient patient numbers comparing these treatments. Objective: To compare interval appendectomy and follow‐up with magnetic resonance imaging after initial successful nonoperative treatment of periappendicular abscess. Design, Setting, and Participants: The Peri‐Appendicitis Acuta randomized clinical trial was a multicenter, noninferiority trial conducted in 5 hospitals in Finland. All patients between age 18 and 60 years with periappendicular abscess diagnosed by computed tomography and successful initial nonoperative treatment from January 2013 to April 2016 were included. Data analysis occurred from April 2016 to September 2017. Interventions: Patients were randomized either to interval appendectomy or follow‐up with magnetic resonance imaging; all patients underwent colonoscopy. Main Outcomes and Measures: The primary end point was treatment success, defined as an absence of postoperative morbidity in the appendectomy group and appendicitis recurrence in the follow‐up group. Secondary predefined end points included neoplasm incidence, inflammatory bowel disease, length of hospital stay, and days of sick leave. Results: A total of 60 patients were included (36 men [60%]; median [interquartile range] age: interval appendectomy group, 49 [18‐60] years; follow‐up group, 47 [22‐61] years). An interim analysis in April 2016 showed a high rate of neoplasm (10 of 60 [17%]), with all neoplasms in patients older than 40 years. The trial was prematurely terminated owing to ethical concerns. Two more neoplasms were diagnosed after study termination, resulting in an overall neoplasm incidence of 20% (12 of 60). On study termination, the overall morbidity rate of interval appendectomy was 10% (3 of 30), and 10 of the patients in the follow‐up group (33%) had undergone appendectomy. Conclusions and Relevance: The neoplasm rate after periappendicular abscess in this small study population was high, especially in patients older than 40 years. If this considerable rate of neoplasms after periappendicular abscess is validated by future studies, it would argue for routine interval appendectomy in this setting. Trial Registration: ClinicalTrials.gov identifier: NCT03013686.

%Z JAMA surgery

Journal article

%U https://cochrane.66557.net/central/doi/10.1002/central/CN-02004854/full

%0 Journal Article

%A McLeod, R. S.

%A Wolff, B. G.

%A Steinhart, A. H.

%A Carryer, P. W.

%A O'Rourke, K.

%A Andrews, D. F.

%A Blair, J. E.

%A Cangemi, J. R.

%A Cohen, Z.

%A Cullen, J. B.

%A Et, Al.

%D 1997

%T Risk and significance of endoscopic/radiological evidence of recurrent Crohn's disease

%V 113

%N 6

%P 1823‐1827

%8 1997-01-01

%R 10.1016/s0016-5085(97)70001-5

%K Actuarial Analysis; Adult; Crohn Disease [*diagnostic imaging, *pathology, surgery]; Endoscopy; Female; Humans; Male; Middle Aged; Radiography; Recurrence; Risk Factors; Treatment Outcome

%X BACKGROUND & AIMS: The aim of this study was to determine the risk of endoscopic/radiological recurrence of Crohn's disease postoperatively and the long‐term outcome. METHODS: A randomized placebo‐controlled trial was performed to determine the effectiveness of mesalamine in preventing recurrent Crohn's disease postoperatively. Patients in the control group were examined endoscopically/radiologically before entry into and annually during the trial. Findings were classified as minimal or severe. RESULTS: There were 76 patients (49 men and 37 women; mean age, 37.1 +/‐ 13.2 years). Fifty (61.7%) had terminal ileal resections. Overall, 55 endoscopic/radiological recurrences were observed in 51 patients (67.1%). Expressed actuarially, the recurrence rate was 27.5% at 1 year (95% confidence interval [CI], 15.8%‐37.6%), 60.8% at 2 years (95% CI, 46%‐71.3%), and 77.3% at 3 years (95% CI, 62.7%‐86.3%). Nineteen (37%) were symptomatic and 12 (24%) were initially asymptomatic but later became symptomatic (mean, 13.0 +/‐ 8.8 months), whereas 20 (39%) remained asymptomatic (mean, 16.9 +/‐ 17.4 months). Patients with severe endoscopic/radiological disease were significantly more likely to be or become symptomatic than those with minimal disease (23 of 32 vs. 8 of 19, respectively; P = 0.0437). CONCLUSIONS: This study suggests that postoperative endoscopic/radiological recurrences occur later than previously reported. Furthermore, many of these patients, especially with minimal disease, will remain asymptomatic.

%Z Gastroenterology

Journal article

%U https://cochrane.66557.net/central/doi/10.1002/central/CN-00145861/full

%0 Journal Article

%A Fazio, V. W.

%A Marchetti, F.

%D 1999

%T Recurrent Crohn's disease and resection margins: bigger is not better

%V 32

%P 135‐168

%8 1999-01-01

%K *Crohn disease /surgery; Anastomosis; Anastomosis, Surgical; Clinical trial; Controlled clinical trial; Crohn Disease [pathology, *surgery]; Frozen Sections; Frozen section; Human; Humans; Pathology; Randomized Controlled Trials as Topic; Randomized controlled trial; Recurrence; Recurrent disease; Reoperation; Review; Risk Factors; Risk factor; Treatment Outcome; Treatment outcome

%Z Advances in surgery

Journal article

%U https://cochrane.66557.net/central/doi/10.1002/central/CN-01713828/full

%0 Journal Article

%A McLeod, R. S.

%A Wolff, B. G.

%A Ross, S.

%A Parkes, R.

%A McKenzie, M.

%D 2009

%T Recurrence of Crohn's disease after ileocolic resection is not affected by anastomotic type: results of a multicenter, randomized, controlled trial

%V 52

%N 5

%P 919‐927

%8 2009-01-01

%R 10.1007/DCR.0b013e3181a4fa58

%K Adult; Anastomosis, Surgical [*methods]; Anti‐Inflammatory Agents, Non‐Steroidal [therapeutic use]; Azathioprine [therapeutic use]; Colon [*surgery]; Crohn Disease [*surgery]; Endoscopy, Gastrointestinal; Female; Humans; Ileum [*surgery]; Immunosuppressive Agents [therapeutic use]; Male; Medication Adherence; Mesalamine [therapeutic use]; Multivariate Analysis; Recurrence; Risk Factors

%X PURPOSE: This study attempts to determine whether stapled side‐to‐side anastomosis, compared with handsewn end‐to‐end anastomosis, results in decreased recurrence of Crohn's disease following ileocolic resection. METHODS: Patients with Crohn's disease who underwent an ileocolic resection were randomized to side‐to‐side anastomosis or end‐to‐end anastomosis. Colonoscopy was performed at 12 months. The primary outcome was endoscopic recurrence, while the secondary outcome was symptomatic recurrence (defined as symptoms attributable to Crohn's disease and severe enough to warrant treatment, plus endoscopic disease recurrence). RESULTS: One hundred and thirty‐nine subjects were included in the efficacy analysis. After a mean follow‐up of 11.9 months, the endoscopic recurrence rate was 42.5 percent in the end‐to‐end anastomosis group, compared with 37.9 percent in the side‐to‐side anastomosis group (‐4.6 percent difference; 95 percent confidence interval ‐21.0 to 11.9 percent; P = 0.55). The symptomatic recurrence rate was 21.9 percent in the end‐to‐end anastomosis group, compared with 22.7 percent in the side‐to‐side anastomosis group (+0.8 percent difference; 95 percent confidence interval ‐13.2 to 15.3 percent; P = 0.92). In multivariate logistic regression analysis, previous resections were predictive of a higher risk of both endoscopic (odds ratio 1.78; 95 percent confidence interval 1.06 to 2.90; P = 0.028) and symptomatic (odds ratio 2.0; 95 percent confidence interval 1.14 to 3.60; P = 0.0016) recurrence. Compliance with postoperative maintenance therapy was predictive of a lower risk of symptomatic recurrence (odds ratio 0.13, 95 percent confidence interval 0.01 to 0.78; P = 0.021). CONCLUSION: Recurrence rates are similar whether end‐to‐end anastomosis or side‐to‐side anastomosis is performed.

%Z Diseases of the colon and rectum

Journal article

%U https://cochrane.66557.net/central/doi/10.1002/central/CN-00698278/full

%0 Journal Article

%A Kulnigg, S.

%A Teischinger, L.

%A Dejaco, C.

%A Waldhör, T.

%A Gasche, C.

%D 2009

%T Rapid recurrence of IBD-associated anemia and iron deficiency after intravenous iron sucrose and erythropoietin treatment

%V 104

%N 6

%P 1460‐1467

%8 2009-01-01

%R 10.1038/ajg.2009.114

%K Adult; Anemia, Iron‐Deficiency [blood, *drug therapy, etiology]; Dose‐Response Relationship, Drug; Erythropoietin [*administration & dosage]; Female; Ferric Compounds [*administration & dosage]; Ferric Oxide, Saccharated; Ferritins [blood]; Follow‐Up Studies; Glucaric Acid; Hemoglobins [metabolism]; Humans; Inflammatory Bowel Diseases [blood, *complications]; Injections, Intravenous; Iron [blood]; Male; Prognosis; Prospective Studies; Recurrence; Treatment Outcome

%X OBJECTIVES: Anemia is a common complication of inflammatory bowel disease (IBD) and iron deficiency (ID) is its predominant cause. Therefore, oral and intravenous iron replacements are widely used. This study was performed to evaluate the frequency and timing of anemia and ID recurrence after a successful treatment cycle. METHODS: Medical records of patients who had received iron sucrose with or without erythropoietin (EPO) in one of three prospective clinical trials that had been conducted at our center (Ann Intern Med 1997, Digestion 1999, and Am J Gastroenterol 2001) were analyzed for a 5‐year follow‐up period. The risk for recurrence of anemia (hemoglobin (Hb)<12/13 g per 100 ml) and ID (ferritin <30 microg/l) was evaluated by Kaplan‐Meier analysis using the log‐rank test. RESULTS: Eighty‐eight patients were available for analysis. Patients had received a mean iron dose of 2,500 mg (range 600‐3,600 mg); 33 (37.1%) patients had also received EPO. Anemia recurred in a median of 10 months (95% confidence interval (CI) 8‐12) and ID recurred within 19 months (95% CI 11‐28). The iron dose had no influence on recurrence of ID or anemia. ID (but not anemia) recurred faster in patients with a post‐treatment ferritin level <100 microg/l (median 4 months, 95% CI 1‐7) than in patients with ferritin level between 100 and 400 microg/l (median 11 months, 95% CI 6‐16) and >400 microg/l (median 49 months, 95% CI 32‐66; P<0.001). CONCLUSIONS: IBD‐associated ID and anemia recur surprisingly fast, indicating that maintenance treatment may be needed in a portion of the patient population. Recurrence of ID (but not anemia) can be delayed by aiming for high post‐treatment ferritin levels.

%Z American journal of gastroenterology

Journal article

%U https://cochrane.66557.net/central/doi/10.1002/central/CN-00698325/full

%0 Journal Article

%A EUCTR ES

%D 2016

%T Randomized, Placebo-Controlled, Double-Blind, Multicenter Study to Determine the Effectiveness and Safety of Vedolizumab in Prevention of Recurrence of Crohn's Disease of the mucosa in Patients with Surgical Removel of the area between the small and the large bowel

%8 2016-01-01

%X INTERVENTION: Trade Name: Entyvio Product Name: Vedolizumab IV Product Code: MLN0002 Pharmaceutical Form: Powder for concentrate for solution for infusion INN or Proposed INN: vedolizumab CAS Number: 943609‐66‐3 Current Sponsor code: MLN0002 Other descriptive name: VEDOLIZUMAB Concentration unit: mg/ml milligram(s)/millilitre Concentration type: equal Concentration number: 60‐ Pharmaceutical form of the placebo: Concentrate and solvent for solution for infusion Route of administration of the placebo: Intravenous use CONDITION: Crohn's disease Therapeutic area: Diseases [C] ‐ Digestive System Diseases [C06] PRIMARY OUTCOME: Main Objective: The primary objective of this prospective placebo‐controlled study is to assess the ability of vedolizumab to prevent postoperative endoscopic recurrence of Crohn’s disease in the neoterminal ileum. Primary end point(s): Proportion of patients with severe endoscopic postoperative recurrence of CD (Rutgeerts i2b, i3 or i4) after approximately 6 months (Week 26). Secondary Objective: Prevention of clinical recurrence of Crohn’s disease Timepoint(s) of evaluation of this end point: week 26 SECONDARY OUTCOME: Secondary end point(s): 1. The proportion of patients with any endoscopic recurrence of CD (Modified Rutgeerts Grade > i0) after 6 months. ; 2. Changes in the CDAI (Crohn’s disease activity index) between week 0 and 26. This measure will give an indication for clinical recurrence. Although most patients will remain asymptomatic, we will collect global scores as well as individual components.; 3. Adverse events and serious adverse events; 4. Quality of life measure with a disease‐specific instrument (IBDQ) and a generic QoL instrument (SF‐36).; 5. Serum concentrations of vedolizumab and antibodies to vedolizumab before every infusion Timepoint(s) of evaluation of this end point: secondary endlpoint 1,2 and 4 at week 26; Secondary endpoint 3 and 5 at all time points INCLUSION CRITERIA: ‐ In the opinion of the investigator, the subject is capable of understanding and complying with protocol requirements. ‐ The subject signs and dates a written, informed consent form and any required privacy authorization prior to the initiation of any study procedures. Consent must be documented. ‐ Established Crohn’s disease as the indication for ileocolonic resection ‐ Age > 18 ‐ Ileocolonic resection with ileocolonic anastomosis and removal of all tissue macroscopically affected by CD according to the surgeon ‐ Presence of at least 1 risk factor for recurrence: o Active smoking > 10 cigarettes/day o 2nd, 3rd or later resection o Surgery for perforating complication (abscess, fistula) o Previous exposure to anti‐TNF antibodies ‐ Male or non‐pregnant, non‐lactating females. Females of child bearing potential must have a negative serum pregnancy test prior to randomization, and must use a hormonal (oral, implantable or injectable)

%Z https://trialsearch.who.int/Trial2.aspx?TrialID=EUCTR2015-000555-24-ES

Trial registry record

%U https://cochrane.66557.net/central/doi/10.1002/central/CN-01854438/full

%0 Journal Article

%A EUCTR FR

%D 2017

%T Randomized, Placebo-Controlled, Double-Blind, Multicenter Study to Determine the Effectiveness and Safety of Vedolizumab in Prevention of Recurrence of Crohn's Disease of the mucosa in Patients with Surgical Removel of the area between the small and the large bowel

%8 2017-01-01

%X INTERVENTION: Trade Name: Entyvio Product Name: Vedolizumab IV Product Code: MLN0002 Pharmaceutical Form: Powder for concentrate for solution for infusion INN or Proposed INN: vedolizumab CAS Number: 943609‐66‐3 Current Sponsor code: MLN0002 Other descriptive name: VEDOLIZUMAB Concentration unit: mg/ml milligram(s)/millilitre Concentration type: equal Concentration number: 60‐ Pharmaceutical form of the placebo: Concentrate and solvent for solution for infusion Route of administration of the placebo: Intravenous use CONDITION: Crohn's disease Therapeutic area: Diseases [C] ‐ Digestive System Diseases [C06] PRIMARY OUTCOME: Main Objective: The primary objective of this prospective placebo‐controlled study is to assess the ability of vedolizumab to prevent postoperative endoscopic recurrence of Crohn’s disease in the neoterminal ileum. Primary end point(s): Proportion of patients with severe endoscopic postoperative recurrence of CD (Rutgeerts i2b, i3 or i4) after approximately 6 months (Week 26). Secondary Objective: Prevention of clinical recurrence of Crohn’s disease Timepoint(s) of evaluation of this end point: week 26 SECONDARY OUTCOME: Secondary end point(s): 1. The proportion of patients with any endoscopic recurrence of CD (Modified Rutgeerts Grade > i0) after 6 months. ; 2. Changes in the CDAI (Crohn’s disease activity index) and HBI (Harvey Bradshaw index) between week 0 and 26. This measure will give an indication for clinical recurrence. Although most patients will remain asymptomatic, we will collect global scores as well as individual components.; 3. Adverse events and serious adverse events; 4. Quality of life measure with a disease‐specific instrument (IBDQ) and a generic QoL instrument (SF‐36).; 5. Serum concentrations of vedolizumab and antibodies to vedolizumab before every infusion; Timepoint(s) of evaluation of this end point: secondary endlpoint 1,2 and 4 at week 26; Secondary endpoint 3 and 5 at all time points INCLUSION CRITERIA: ‐ In the opinion of the investigator, the subject is capable of understanding and complying with protocol requirements. ‐ The subject signs and dates a written, informed consent form and any required privacy authorization prior to the initiation of any study procedures. ‐ Established Crohn’s disease as the indication for ileocolonic resection ‐ Age > 18 ‐ Ileocolonic resection with ileocolonic anastomosis and removal of all tissue macroscopically affected by CD according to the surgeon ‐ Presence of at least 1 risk factor for recurrence: • Active smoking > 10 cigarettes/day • 2nd, 3rd or later resection • Surgery for perforating complication (abscess, fistula) • Previous exposure to anti‐TNF antibodies ‐ Male or non‐pregnant, non‐lactating females. Females of child bearing potential must have a negative serum pregnancy test prior to randomization, and must use a hormonal (oral, implantable or injectable) or barrier method of birth

%Z https://trialsearch.who.int/Trial2.aspx?TrialID=EUCTR2015-000555-24-FR

Trial registry record

%U https://cochrane.66557.net/central/doi/10.1002/central/CN-01911108/full

%0 Journal Article

%A NCT

%D 2018

%T Randomized Trial : hemorrhoidal Pedicle Ligation vs Laser vs Open Hemorrhoidectomy

%8 2018-01-01

%K Hemorrhoids

%X Hemorrhoidal pedicle ligation vs laser hemorrhoidectomy vs open hemorrhoidectomy: randomized, double blind, multicenter trial AIMS AND OBJECTIVES The aim of this study is to compare three different modalities for treatment of symptomatic 2 to 3 ° haemorrhoids: open haemorrhoidectomy, intrahaemorrhoidal laser procedure and haemorrhoidal pedicle ligation. Objectives of the study are: To assess early outcomes (after one week and one month) of the procedures: pain, bleeding, wound healing, return to work and quality of life; To assess late outcomes (after one year) of the procedures: late functional results (continence) and recurrence of symptoms and haemorrhoids. MATERIALS AND METHODS Study design This is a randomized, parallel group (1:1:1) double‐blinded single centre prospective study. No changes in methods of the study were allowed after commen cement. This RCT compares three different modalities for treatment of symptomatic 2 to 3 ° haemorrhoids: open haemorrhoidectomy, intrahaemorrhoidal laser coagulation and haemorrhoidal artery ligation. Patients This prospective randomized study was performed at Vilnius University Hospital Santara Clinics, Vilnius, Lithuania. It is a large tertiary University hospital with dedicated outpatient clinic. A period of 3 year starting from April 2015 to November 2018. A total of 121 patients are included in the study. Patients with symptomatic 2nd or 3rd ° hemorrhoids, in 1st or 2nd risk group of ASA (American Society of Anesthesiologists), who consented to participate in this study were included into the study. Exclusion criteria were 1st or 4th °of hemorrhoids, pregnancy, patients with other anorectal diseases (fistula, abscess, rectal carcinoma, inflammatory bowel disease, etc.), patients after previous anal operations (except rubber band ligation, which should have occurred more than 3 months before the inclusion in the trial) and ≥ 3rd risk group of ASA. Preoperative evaluation Detailed physical and anorectal examination was performed with anoscopy and rigid proctoscopy in all cases, as well as colonoscopy if indicated. All patients filled a dedicated symptom questionnaire, which included questions on intensity and frequency of hemorrhoidal prolapse, bleeding, itching, pain and other symptoms. Every patient completed Wexner incontinence score and SF‐36 questionnaires. Preoperative examination, classification, operation technique, postoperative treatment and follow‐up are discussed at the introductory course. Patients preoperatively fill questionnaires on the quality of life and defecation function (Patient symptoms scale, Cleveland clinic incontinence scale, Health survey ‐ SF 36, Fecal incontinence quality of life instrument ‐ FIQol). Patient symptoms scale (appendix 1) ‐ symptoms of haemorrhoids are assessed (rectal bleeding, pain, pile prolapse, defecation disturbances, discomfort affecting normal life). Each symptom gets a number from 1 ‐ very intense to 5 ‐ no complaints. Cleveland clinic incontinence scale (appendix 2) ‐ the sum of 5 parameters is determined that are scored on a scale from 0 (=absent) to 4 (daily) frequency of incontinence to gas, liquid, solid, of need to wear pad, and of lifestyle changes. A score of 0 means perfect control, a score of 20 ‐ complete incontinence. Randomization, blinding and concealment The patients were randomized into three groups. Randomization sequence was computer‐generated before the start of the trial. Every consecutive case Health survey ‐ SF 36 (appendix 3) ‐ it measures eight domains: physical functioning, role limitations due to physical health, role limitations due to emotional problems, energy/fatigue, emotional well‐being, social functioning, pain, general health. For each of the eight domains that the SF36 measures an aggregate percentage score is produced. The percentage scores range from 0% (lowest or worst possible level of functioning) to 100% (highest or best possible level of functioning). Fecal incontinence quality of life instrument ‐ FIQol (appendix 4) ‐scale consists of 29 questions in 4 domains: lifestyle, coping/behavior, depression/self perception, embarrassment. Category ranges: 1 to 4 for lifestyle, coping/behavior, and embarrassment; 1 to 6 for depression/self perception. ‐history was assigned a randomization number (1, 2 or 3). It was written and sealed within the envelope and remained unknown neither to the patient, nor to the treating physician, to avoid selection bias. In the operating room, after induction of anesthesia, operating room junior staff was asked to unseal the envelope and the intervention was performed according to the procedure assigned. Pre‐ and postoperative patient management was identical in all three operations. The patient remained unaware of the procedure performed until the end of the study 1 year after the operation. The case‐notes and discharge summary of the patient contained only the note, saying that the patient is included in the study of hemorrhoids with D.Danys/T.Poskus as Principal Investigators, patient's number is X. This number was within the locked and coded database and the staff, evaluating the result of the patients' treatment remained unaware of the procedure allocated. The patients were followed‐up by different surgeons (E.P. and V.J.) than the ones, performing the operation. They had the access to the patient notes but not to the coded database, and were not able to know, which procedure was performed. In emergency situations, un‐blinding of the patient and treating physicians was possible but was not required in any of the patients. Statistical analysis Sample size calculation was performed using R statistical software package(©The R Foundation).Presuming the effect size of 0.3, power of 0.8 and alpha=0.05 the sample size was calculated to be 108 patients. To compensate for possible follow‐up losses sample size was increased to 120 patients. Chi‐square test and Anova tests were used to compare differences between the groups. Operative procedure Patients were started on lactulose the day before the operation, which was continued after the operation to have regular bowel movements. Preoperative intravenous antibiotic prophylaxis was given according to the hospital protocol, which was 1g of Cephazolin (2g if patient was over 80 kg of weight), 240 mg of gentamycin and 500 mg of metronidazole (Ciprofloxacin can be used if patient is allergic to cephalosporins). Each surgeon performing operative procedures (T.P., D.D. and S.M.) had personal experience of at least 50 operations of each modality. 1 hour seminar was conducted between all surgeons before the start of the trial to unify the technique of operative procedures.All patients were photographed after induction of anesthesia before the start of the procedure and immediately after the procedure.LHP was performed using Ceralas diode laser of 1420 nm wavelength (Biolitec). Disposable LHP kit (Biolitec) was used, which contains sharp‐tipped laser fiber and anoscope. Perianal skin immediately aboral to hemorrhoid was penetrated using needle���tip cautery (Figure 2). Laser fiber was introduced into the opening until the level of hemorrhoidal pedicle and coagulation was activated. 8 Watt 3 second pulses with 1 second pulse‐pauses were used to coagulate the area of hemorrhoids. 5 mm of hemorrhoidal tissue is coagulated with one such pulse (experimental data). 250 Joules was the upper limit of energy delivered per 1 hemorrhoidal quadrant. Smaller hemorrhoids were treated with less energy, larger ones ‐ with more energy. The procedure was repeated in three other quadrants, thus treating all anal circumference. RAR was performed as described by Schurmann JP et al. For these patients, the ligations were placed in the area of visible pathologic hemorrhoidal tissue, and in patients with large prolapse mucopexy ‐ lifting of prolapsing hemorrhoidal tissue with sutures was performed. Standard EH was performed up to the level of hemorrhoidal pedicle, with ligation or suture‐ligation of the pedicle and meticulous hemostasis. Follow‐up was performed by different surgeons to those, performing the operations (E.P., V.J.). Each of them has more than 25 years of experience of colorectal and hemorrhoidal surgery. 1 hour seminar was conducted with them to unify the evaluation of the patients within the study. Each patient was followed up at 1 and 6 weeks and after 1 year after the operation (Figure 1). Perianal examination with photographic documentation was performed during all the visits. Anoscopy was performed during visits at 6 weeks and 1 year. Each patient was asked to fill in the diary during each day of the first post‐operative week and present it at the first visit after 1 week. Symptom questionnaires were filled in during visits at 1 and 6 weeks and 1 year. Wexner incontinence score was filled during the visit at 6 weeks and 1 year. SF‐36 QOL questionnaire was filled during the final visit at 1 year.

%Z https://clinicaltrials.gov/show/NCT03757728

Trial registry record

%U https://cochrane.66557.net/central/doi/10.1002/central/CN-01795167/full

%0 Journal Article

%A Sungurtekin, U.

%A Ozgen, U.

%A Sungurtekin, H.

%D 2022

%T Prospective, Randomized, Controlled Trial of Ultra-modified Internal Sphincterotomy vs Closed Lateral Internal Sphincterotomy for Chronic Fissure-in-Ano

%V 88

%N 9

%P 2388‐2396

%8 2022-01-01

%R 10.1177/00031348211011104

%K *anus fissure; *feces incontinence; *prospective study; *sphincterotomy; Adult; Anus sphincter; Article; Cleveland Clinic Florida Fecal Incontinence score; Complication; Continence; Controlled study; Deterioration; Female; Florida; Follow up; Healing rate; Human; Major clinical study; Male; Outcome assessment; Pain; Patient satisfaction; Postoperative complication; Randomization; Randomized controlled trial; Side effect; Surgeon; adult; anal stenosis; anorectal pressure; anus continence; anus disease; anus fissure [surgery]; anus pruritus [complication]; article; assessment of humans; bleeding [complication]; clinical assessment; closed lateral internal sphincterotomy; comparative effectiveness; constipation [complication]; controlled study; diarrhea; disease severity; female; follow up; hospital admission; human; induration [complication]; inflammatory bowel disease; major clinical study; male; manometry; outcome assessment; patient satisfaction; postoperative complication [complication]; prospective study; randomized controlled trial; scoring system; skin tag [complication]; sphincterotomy; ultra modified internal sphincterotomy; visual analog scale

%X Background: Currently, the lateral internal sphincterotomy is the treatment of choice for a chronic anal fissure (CAF). However, the length of the internal sphincter incision varies, due to lack of standardization. Insufficient length increases the risk of recurrence. To compare a new ultra‐modified internal sphincterotomy (UMIS) to the closed lateral internal sphincterotomy (CLIS) for treating CAF, based on internal anal sphincter function and postoperative complications. The primary endpoint was continence after UMIS. The secondary outcomes were CAF healing complications, visual analog scale pain scores, and sphincter pressures. Methods: This was a prospective, randomized, controlled trial (block randomization method). 200 patients with CAFs were randomly assigned to receive either UMIS (n = 100) or the closed lateral internal sphincterotomy (CLIS) (n = 100). Follow‐up was 2 years. RESULTS: All (100%) patients in both groups showed clinical improvement at 1 month post‐surgery. Recurrences were accompanied by deteriorations in Cleveland Clinic Florida Fecal Incontinence scores at 12 months and 2 years (P <.05). The groups showed significant differences in fissure healing rates and pain scores. After 1 and 2 years, incontinence rates were significantly higher, and patient satisfaction scores were significantly lower in the CLIS group than the UMIS group (P <.05). Conclusion: UMIS provided a faster healing rate and fewer side effects than the CLIS for treating CAFs. These results might lead to a standardized treatment among surgeons.

%Z American surgeon

Journal article

%U https://cochrane.66557.net/central/doi/10.1002/central/CN-02343357/full

%0 Journal Article

%A Lochs, H.

%A Mayer, M.

%A Fleig, W. E.

%A Mortensen, P. B.

%A Bauer, P.

%A Genser, D.

%A Petritsch, W.

%A Raithel, M.

%A Hoffmann, R.

%A Gross, V.

%A Et, Al.

%D 2000

%T Prophylaxis of postoperative relapse in Crohn's disease with mesalamine: european Cooperative Crohn's Disease Study VI

%V 118

%N 2

%P 264‐273

%8 2000-01-01

%R 10.1016/s0016-5085(00)70208-3

%K Adult; Aged; Anti‐Inflammatory Agents, Non‐Steroidal [*therapeutic use]; Crohn Disease [*prevention & control, *surgery]; Double‐Blind Method; Europe; Female; Follow‐Up Studies; Humans; Male; Mesalamine [*therapeutic use]; Middle Aged; Placebos; Proportional Hazards Models; Prospective Studies; Recurrence; Risk Factors; Time Factors; Treatment Outcome

%X BACKGROUND & AIMS: This study investigated if long‐term treatment with high‐dose mesalamine reduces the risk of clinical relapse of Crohn's disease after surgical resection. METHODS: In a prospective, randomized, double‐blind, multicenter study, 4 g of mesalamine (Pentasa; Ferring A/S, Vanlose, Denmark) daily was compared with placebo in 318 patients. Treatment was started within 10 days after resective surgery and continued for 18 months. Primary outcome parameter was clinical relapse as defined by an increase in Crohn's Disease Activity Index, reoperation, septic complication, or newly developed fistula. Risk factors for recurrence were prospectively defined to be analyzed in a stepwise proportional hazards model. RESULTS: Cumulative relapse rates (+/‐SE) after 18 months were 24.5% +/‐ 3.6% and 31.4% +/‐ 3.7% in the mesalamine (n = 152) and placebo (n = 166) groups, respectively (P = 0.10, log‐rank test, 1‐sided). Retrospective analysis showed a significantly reduced relapse rate with mesalamine only in a subgroup of patients with isolated small bowel disease (n = 124; 21.8% +/‐ 5.6% vs. 39.7% +/‐ 6.1%; P = 0.02, log‐rank test). Probability of relapse was predominantly influenced by the duration of disease (P = 0.0006) and steroid intake before surgery (additional risk, P = 0.0003). CONCLUSIONS: Eighteen months of mesalamine, 4 g daily, did not significantly affect the postoperative course of Crohn's disease. Some relapse‐preventing effect was found in patients with isolated small bowel disease.

%Z Gastroenterology

Journal article

%U https://cochrane.66557.net/central/doi/10.1002/central/CN-00266147/full

%0 Journal Article

%A McLeod, R. S.

%A Wolff, B. G.

%A Steinhart, A. H.

%A Carryer, P. W.

%A O'Rourke, K.

%A Andrews, D. F.

%A Blair, J. E.

%A Cangemi, J. R.

%A Cohen, Z.

%A Cullen, J. B.

%D 1995

%T Prophylactic mesalamine treatment decreases postoperative recurrence of Crohn's disease

%V 109

%N 2

%P 404‐413

%8 1995-01-01

%R 10.1016/0016-5085(95)90327-5

%K Adult; Aminosalicylic Acids [adverse effects, *therapeutic use]; Anti‐Inflammatory Agents, Non‐Steroidal [adverse effects, *therapeutic use]; Combined Modality Therapy; Confidence Intervals; Crohn Disease [prevention & control, *surgery]; Double‐Blind Method; Female; Follow‐Up Studies; Humans; Male; Mesalamine; Pancreatitis [chemically induced]; Patient Compliance; Recurrence; Risk Factors

%X BACKGROUND & AIMS: Recurrence of Crohn's disease frequently occurs after surgery. A randomized controlled trial was performed to determine if mesalamine is effective in decreasing the risk of recurrent Crohn's disease after surgical resection is performed. METHODS: One hundred sixty‐three patients who underwent a surgical resection and had no evidence of residual disease were randomized to a treatment group (1.5 g mesalamine twice a day) or a placebo control group within 8 weeks of surgery. The follow‐up period was a maximum of 72 months. RESULTS: The symptomatic recurrence rate (symptoms plus endoscopic and/or radiological confirmation of disease) in the treatment group was 31% (27 of 87) compared with 41% (31 of 76) in the control group (P = 0.031). The relative risk of developing recurrent disease was 0.628 (90% confidence interval, 0.40‐0.97) for those in the treatment group (P = 0.039; one‐tail test) using an intention‐to‐treat analysis and 0.532 (90% confidence interval, 0.32‐0.87) using an efficacy analysis. The endoscopic and radiological rate of recurrence was also significantly decreased with relative risks of 0.654 (90% confidence interval, 0.47‐0.91) in the effectiveness analysis and 0.635 (90% confidence interval, 0.44‐0.91) in the efficacy analysis. There was only one serious side effect (pancreatitis) in subjects in the treatment group. CONCLUSIONS: Mesalamine (3.0 g/day) is effective in decreasing the risk of recurrence of Crohn's disease after surgical resection is performed.

%Z Gastroenterology

Journal article

%U https://cochrane.66557.net/central/doi/10.1002/central/CN-00116234/full

%0 Journal Article

%A Caprilli, R.

%A Corrao, G.

%A Taddei, G.

%A Tonelli, F.

%A Torchio, P.

%A Viscido, A.

%D 1996

%T Prognostic factors for postoperative recurrence of Crohn's disease. Gruppo Italiano per lo Studio del Colon e del Retto (GISC)

%V 39

%N 3

%P 335‐341

%8 1996-01-01

%R 10.1007/BF02049478

%K Adult; Aminosalicylic Acids [therapeutic use]; Anti‐Inflammatory Agents, Non‐Steroidal [therapeutic use]; Combined Modality Therapy; Crohn Disease [drug therapy, *surgery]; Female; Follow‐Up Studies; Humans; Life Tables; Male; Mesalamine; Predictive Value of Tests; Prognosis; Proportional Hazards Models; Recurrence; Risk Factors

%X Prognostic factors for postoperative recurrence of Crohn's disease (CD) have been widely investigated but not yet clearly identified. PURPOSE: Aim of this study was, therefore, to analyze the association between demographic, clinical, laboratory, and surgical characteristics of patients and the cumulative probability of endoscopic postoperative recurrence. METHODS: The study was performed in 110 patients who were enrolled in the Italian multicenter, randomized, controlled trial on the effectiveness of 5‐aminosalicylic acid (5‐ASA) in the prevention of postoperative recurrence in CD. Patients had undergone their first intestinal resection for CD of the terminal ileum with or without involvement of cecum ascending colon. Recurrence was defined on the basis of endoscopy. The following variables were evaluated as potential prognostic factors: gender, age, years since diagnosis, clinical course (perforative and non‐perforative), Crohn's Disease Activity Index score, white blood count, erythrocyte sedimentation rate, C‐reactive protein, and orosomucoids assessed before the operation. Timing of operation (elective or urgent), type of anastomosis (end‐to‐end, end‐to‐side, side‐to‐side), and prophylactic treatment were also evaluated. Colon ileoscopy was performed at 6, 12, 24, and 36 months after operation. The association between variables and the cumulative proportion of recurrence was analyzed both by univariate analysis (life table method, log‐rank test) and multivariate regression analysis (Cox's model, stepwise procedure). RESULTS: Results of this study indicate that, of the features considered before surgery, only leukocytosis (white blood count, >9,000 ml) was significantly associated with an increased risk of recurrence (P < 0.05) at univariate analysis. This finding was not confirmed by multivariate analysis. A trend toward a higher risk of recurrence for patients who have had a resection with end‐to‐end anastomosis compared with those who have had a resection and other types of anastomosis was also observed. This trend reached significantly in the group of patients submitted to treatment with 5‐ASA. The multivariate analysis showed that 5‐ASA‐treated patients with end‐to‐end had a risk of recurrence more than threefold higher than those with other types of anastomosis (relative risk, 3.40; 95 percent confidence interval, 1.00‐11.96; P < 0.03). CONCLUSIONS: From a practical point of view, it has been estimated that the combination of intestinal resection plus side‐to‐side or end‐to‐side anastomosis with oral 5‐ASA treatment reduces by 64 percent the postoperative recurrence rate in CD at three years follow‐up.

%Z Diseases of the colon and rectum

Journal article

%U https://cochrane.66557.net/central/doi/10.1002/central/CN-00123697/full

%0 Journal Article

%A Gelbmann, C. M.

%A Rogler, G.

%A Gross, V.

%A Gierend, M.

%A Bregenzer, N.

%A Andus, T.

%A Schölmerich, J.

%D 2002

%T Prior bowel resections, perianal disease, and a high initial Crohn's disease activity index are associated with corticosteroid resistance in active Crohn's disease

%V 97

%N 6

%P 1438‐1445

%8 2002-01-01

%R 10.1111/j.1572-0241.2002.05685.x

%K Adrenal Cortex Hormones [*therapeutic use]; Adult; Aged; Anus Diseases [*complications]; Constriction, Pathologic; Crohn Disease [complications, *drug therapy, physiopathology, *surgery]; Double‐Blind Method; Drug Resistance; Female; Forecasting; Glucocorticoids [therapeutic use]; Humans; Intestinal Diseases [complications]; Intestines [*surgery]; Male; Middle Aged; Prednisolone [therapeutic use]; Prospective Studies; Rectal Fistula [complications]; Severity of Illness Index; Treatment Outcome

%X OBJECTIVES: Some patients with Crohn's disease (CD) do not respond to corticosteroid therapy. Furthermore, corticosteroids frequently cause side effects. Thus, predictive parameters for treatment refractoriness would be helpful for treatment decisions. METHODS: A total of 300 patients with active CD (i.e., with a Crohn's Disease Activity Index [CDAI] >200) entered the study. Treatment started with 60‐100 mg/day prednisolone equivalent, which was then tapered to 10‐15 mg/day within 6 wk and maintained at that dose for another 4 wk. After 10 wk of treatment, response to steroids was defined by a CDAI <150, steroid resistance by a CDAI always > or =150 and steroid dependency by a relapse after dose reduction. Of 239 eligible patients, 196 were responders, 26 were steroid resistant, and 17 were steroid dependent. RESULTS: Prior bowel resections, a high initial CDAI, and perianal disease were associated with steroid resistance. Of the steroid resistant patients 53.9% were bowel‐resected compared to 20.4% of the responders (relative risk = 3.63; 95% CI = 1.79‐7.36). Perianal disease was observed in 42.3% of steroid resistant patients versus 21.9% of responders (relative risk = 2.28; 95% CI = 1.12‐4.66) and initial CDAI was 347+/‐91 in resistant patients versus 301+/‐81 in responders (p < 0.05). Parameters for steroid dependent patients were not significantly different from those of responders. CONCLUSIONS: In this study (thus far the largest study for the evaluation of predictive factors for treatment refractoriness to corticosteroids in CD), only prior bowel resection, perianal disease, and a high initial CDAI were found to be predictive of resistance to steroid treatment.

%Z American journal of gastroenterology

Journal article

%U https://cochrane.66557.net/central/doi/10.1002/central/CN-00389978/full

%0 Journal Article

%A Swoger, J. M.

%A Regueiro, M.

%D 2010

%T Preventive therapy in postoperative Crohn's disease

%V 26

%N 4

%P 337‐343

%8 2010-01-01

%R 10.1097/MOG.0b013e328338f724

%K *Crohn disease /drug therapy /complication /diagnosis /drug therapy; *postoperative complication; *recurrent disease /drug therapy /complication /diagnosis /drug therapy /prevention; Add on therapy; Anti‐Inflammatory Agents [therapeutic use]; Cigarette smoking; Clinical trial; Colonoscopy; Controlled clinical trial; Crohn Disease [genetics, *prevention & control, *surgery]; Disease duration; Drug efficacy; Drug safety; Drug withdrawal; Echography; Endosonography; Family history; Feces [chemistry]; Feces analysis; Gastrointestinal Agents [therapeutic use]; Human; Humans; Lactoferrin [analysis]; Leukocyte L1 Antigen Complex [analysis]; Meta analysis; Nuclear magnetic resonance imaging; Postoperative Complications [*prevention & control]; Postoperative infection /side effect; Predictor variable; Randomized controlled trial; Recurrence; Review; Risk Factors; Risk factor; Smoking [adverse effects]; Surgical technique; Systematic review; Time Factors; Treatment outcome; Unspecified side effect /side effect; Wound healing impairment /side effect

%X PURPOSE OF REVIEW: Recurrence of Crohn's disease following surgical resection is common, but the optimal strategy to assess, prevent, and treat postoperative recurrence remains unclear. Recent developments in the prevention and management of postoperative recurrence have provided additional information. RECENT FINDINGS: Predictors of Crohn's disease recurrence after surgery include cigarette smoking, disease behavior, number of prior resections, family history, anastomotic type, and time to first surgery. Only penetrating disease behavior and continued cigarette smoking after surgery remain clear predictors of postoperative Crohn's disease recurrence. Ileocolonoscopy is the only modality to detect mucosal recurrence after surgery; however, surrogate markers of inflammation, specifically stool lactoferrin and calprotectin as well as small intestine contrast ultrasound, are promising. Due to the high rate of surgery for the treatment of complications of Crohn's disease, prevention of postoperative disease has received considerable attention. Recent studies of azathioprine/6‐mercaptopurine, nitroimidazole antibiotics, and infliximab have broadened the spectrum of medication options postoperatively. SUMMARY: Smoking cessation and ileocolonoscopy for early detection of Crohn's disease recurrence should be part of any postoperative management strategy. The selection of medication and optimal time to initiate treatment after surgery is less certain. Postoperative immunomodulators and antitumor necrosis factor agents may prevent Crohn's disease in those at high risk for recurrence. Treatment of patients by predictors of recurrence and personalization of management based on genotypes/phenotypes will be the focus of future study.

%Z Current opinion in gastroenterology

Journal article

%U https://cochrane.66557.net/central/doi/10.1002/central/CN-01770285/full

%0 Journal Article

%A Cezard, J. P.

%A Munck, A.

%A Mouterde, O.

%A Morali, A.

%A Lenaerts, C.

%A Lachaux, A.

%A Turck, D.

%A Schmitz, J.

%A Maurage, C.

%A Girardet, J. P.

%A Et, Al.

%D 2009

%T Prevention of relapse by mesalazine (Pentasa) in pediatric Crohn's disease: a multicenter, double-blind, randomized, placebo-controlled trial

%V 33

%N 1 Pt 1

%P 31‐40

%8 2009-01-01

%R 10.1016/j.gcb.2008.07.007

%K Anti‐Inflammatory Agents, Non‐Steroidal [*therapeutic use]; Child; Crohn Disease [*drug therapy]; Double‐Blind Method; Female; Humans; Male; Mesalamine [*therapeutic use]; Secondary Prevention; Treatment Outcome

%X AIM: This study aimed to test the efficacy of mesalazine in maintaining remission in pediatric Crohn's disease (CD) following successful flare‐up treatment. METHODS: In this double‐blind, randomized, placebo‐controlled trial, 122 patients received either mesalazine 50mg/kg per day (n=60) or placebo (n=62) for one year. Treatment allocation was stratified according to flare‐up treatment (nutrition or medication alone). Recruitment was carried out over two periods, as the first period's results showed a trend favoring mesalazine. Relapse was defined as a Harvey‐Bradshaw score more than or equal to 5. Time to relapse was analyzed using the Cox model. RESULTS: The one‐year relapse rate was 57% (n=29) and 63% (n=35) in the mesalazine and placebo groups, respectively. We demonstrated a twofold lower relapse risk (P<0.02) in patients taking mesalazine in the medication stratum (first recruitment period), and a twofold higher risk in patients taking mesalazine in the nutrition stratum (second recruitment period), compared with the other groups. None of the children's characteristics, which differed across the two recruitment periods, accounted for the between‐period variation in mesalazine efficacy. One serious adverse event was reported in each treatment group. CONCLUSION: Overall, mesalazine does not appear to be an effective maintenance treatment in pediatric CD.

%Z Gastroenterologie clinique et biologique

Journal article

%U https://cochrane.66557.net/central/doi/10.1002/central/CN-00680959/full

%0 Journal Article

%A Armuzzi, A.

%A Felice, C.

%A Marzo, M.

%A Pugliese, D.

%A Andrisani, G.

%A Papa, A.

%A De Vitis, I.

%A Rapaccini, G. L.

%A Guidi, L.

%D 2012

%T Prevention of postoperative recurrence with azathioprine or anti-TNF alpha in patients with crohn's disease: an open-label pilot study

%V 142

%N 5

%P S780

%8 2012-01-01

%K *Crohn disease; *azathioprine; *human; *patient; *pilot study; *prevention; *tumor necrosis factor alpha; C reactive protein; Colonoscopy; Crohn disease; Disease course; Infliximab; Male; Metronidazole; Postoperative care; Prophylaxis; Prospective study; Recurrence risk; Risk; Smoking; Surgery; Therapy; colonoscopy; disease course; human; male; patient; pilot study; postoperative care; prevention; prophylaxis; prospective study; recurrence risk; risk; smoking; surgery; therapy

%X Background and aim: Patients with Crohn's disease (CD) often require surgery over their clinical course. However, endoscopic and clinical recurrence of disease appear respectively in up to 80% and 30% of patients after 1 year. Thus, a prophylactic treatment is needed to reduce the possibility of recurrence. At this regard, both azathioprine and infliximab have been demonstrated to be effective, but there are no studies that directly compared them. Aim of this open‐label prospective study was to analyse endoscopic and clinical recurrence after 1 year of treatment with azathioprine or anti‐TNFα as postoperative therapies in CD patients with “high risk” of recurrence. Methods: Consecutive CD patients who underwent curative ileocolonic resection were randomized (1:1) to receive anti‐TNFα (standard induction and maintenance schedule) or azathioprine (2.5 mg/kg/day) for 1 year. Therapy was started within 2‐4 weeks after surgery. All patients also received metronidazole for 2 weeks after surgery. No other CD‐related drugs were admitted during the study. Primary endpoint was to compare percentages of endoscopic and clinical recurrence after 12 months of therapy between the two groups. Colonoscopy, C‐Reactive Protein (CRP) and evaluation of clinical activity (Harvey‐Bradshaw index) were performed after 12 months of therapy. Results: Twenty‐two consecutive CD patients (15 male; median age 32 years, range 18‐70) were enrolled after curative ileocolonic resection. Eleven patients were treated with anti‐TNFα and 11 received azathioprine. There were no differences between groups (age, duration of disease, smoking, previous surgery, disease behaviour and location, perianal disease, extraintestinal manifestations). No re‐operation occurred during the study. One patient did not tolerate azathioprine and withdrew from the study. No other significant adverse events were reported. Among patients treated with azathioprine, 4/10 (40%) had endoscopic recurrence (Rutgeerts' score ≥2) compared to 1/11 (9%) in the anti‐TNFα group (P=ns). No significant difference was found in clinical relapse rates between the two groups: 3/11 (27%) of patients treated with anti‐TNFα and 2/10 (20%) of those who received azathioprine. At the end of the study, also median CRP resulted not different between groups. Conclusions: In this small open‐label prospective study, anti‐TNFα was more effective than azathioprine in reducing endoscopic recurrence after curative ileocolonic resection in “high risk” CD patients. Both treatments were well tolerated.

%Z Gastroenterology

Journal article; Conference proceeding

%U https://cochrane.66557.net/central/doi/10.1002/central/CN-01026274/full

%0 Journal Article

%A NCT

%D 2021

%T Prevention of Postoperative Endoscopic Recurrence With Endoscopy-driven Versus Systematic Biological Therapy

%8 2021-01-01

%K Adalimumab; Crohn Disease; Infliximab; Recurrence; Ustekinumab; Vedolizumab

%X This will be a prospective, randomized, parallel group, pragmatic trial. Prior to study group assignment, the type of biological therapy to be (eventually) used in the postoperative phase will be selected by the treating physician after thorough discussion with the patient. The use of cheaper anti‐TNF biosimilars will be encouraged, but patients who received adalimumab and/or infliximab preoperatively cannot receive the same treatment again in SOPRANO CD if the participants previously encountered immunogenicity issues to this treatment. Systematic postoperative prophylaxis with a biological: Biological therapy (adalimumab, infliximab, ustekinumab, vedolizumab or risankizumab) will be initiated within 14 to 40 days after ileocolonic resection or restoration of the faecal stream (day 0). In patients with both Harvey‐Bradshaw Index (HBI) based clinical recurrence (HBI >4) and endoscopic recurrence (Rutgeerts score ≥i2b) at week 30, biological therapy will be optimized (reimbursed or through the available free goods / samples programs). Beyond week 32 optimization of this biological therapy will be allowed following daily clinical practice including proactive therapeutic drug monitoring. However, the timing, type and reason for dose optimization should be recorded. Endoscopy‐driven postoperative biological therapy: No CD related therapy will be administered between Baseline (14 to 40 days after ileocolonic resection or restoration of the faecal stream) and the endoscopic evaluation at week 30 Patients with endoscopic recurrence (Rutgeerts score ≥i2b) at week 30 will initiate biological therapy (adalimumab, infliximab, ustekinumab, vedolizumab or risankizumab) following a classical induction and maintenance schedule. The type of biological therapy has to be decided already in the perioperative phase to allow a proper stratification. In patients initiating biological therapy at week 30, this therapy maybe optimized from week 32 onwards following daily clinical practice including proactive therapeutic drug monitoring. However, the timing, type and reason for dose optimization should be recorded. In patients not on biological therapy yet but developing clinical recurrence (HBI >4) with objective signs of disease recurrence (faecal calprotectin >250 µg/g, C‐reactive protein >5 mg/L or endoscopic recurrence ≥i2b or clear radiological disease activity at the neo‐terminal ileum) beyond week 32, biological therapy can be initiated, but this will be regarded as a study failure. Randomization: Eligible patients will be allocated to one of the two treatment arms (1:1) according to a computer generated randomisation list in REDCap. Stratified randomisation will be performed to achieve approximate balance for: ‐ Type of selected postoperative prophylactic therapy: adalimumab, infliximab, ustekinumab, vedolizumab or risankizumab. ‐ Number of risk factors for postoperative recurrence: 1, 2 or >2 (out of 5 predefined factors: active smoking, penetrating disease, previous ileocolonic resection ≤10 years of index surgery, ≥2 previous ileocolonic resections, biological therapy ≤3 months of index ileocolonic resection)

%Z https://clinicaltrials.gov/show/NCT05169593

Trial registry record

%U https://cochrane.66557.net/central/doi/10.1002/central/CN-02355455/full

%0 Journal Article

%A Clarke, K.

%A Regueiro, M.

%D 2009

%T Prevention and treatment options for postoperative Crohn's disease: a clinical dilemma

%V 5

%N 8

%P 581‐588

%8 2009-01-01

%K *Crohn disease /drug therapy /drug therapy /prevention /surgery; Accuracy; Clinical evaluation; Clinical trial; Controlled clinical trial; Disease activity; Drug dose comparison; Drug efficacy; Drug tolerability; Echography; Endoscopy; Follow up; Human; Intestine resection; Patient monitoring; Placebo effect; Practice guideline; Prophylaxis; Randomized controlled trial; Recurrence risk; Recurrent disease; Review; Risk factor; Sensitivity and specificity; Treatment outcome

%X The majority of patients with Crohn's disease require surgery for disease‐related complications. Postoperative Crohn's disease recurrence is common after intestinal resection. The optimal management strategy for postoperative recurrence of Crohn's disease is controversial. In the absence of universally adopted guidelines, clinicians and patients must discuss and weigh the risks and benefits of postoperative pharmacotherapy. Those patients at low risk of disease recurrence may not require treatment. On the other hand, patients with more aggressive disease and high risk of recurrence may be best treated early in the postoperative period with an immunomodulator or antitumor necrosis factor agents. Ideally, postoperative treatment decisions would be made using predictable, reliable, and reproducible clinical prediction criteria that would guide treatment. This article reviews the data on postoperative Crohn's disease, including predictors of early recurrence, available options for postoperative monitoring, timing of initiation, and choice of postoperative therapy for prevention and management.

%Z Gastroenterology and hepatology

Journal article

%U https://cochrane.66557.net/central/doi/10.1002/central/CN-01727672/full

%0 Journal Article

%A Regueiro, M.

%A El-Hachem, S.

%A Kip, K. E.

%A Schraut, W.

%A Baidoo, L.

%A Watson, A.

%A Swoger, J.

%A Schwartz, M.

%A Barrie, A.

%A Pesci, M.

%A Et, Al.

%D 2011

%T Postoperative infliximab is not associated with an increase in adverse events in Crohn's disease

%V 56

%N 12

%P 3610‐3615

%8 2011-01-01

%R 10.1007/s10620-011-1785-9

%K Adult; Antibodies, Monoclonal [*administration & dosage]; Colectomy [*adverse effects]; Colonoscopy; Crohn Disease [diagnosis, drug therapy, *surgery]; Dose‐Response Relationship, Drug; Double‐Blind Method; Drug Administration Schedule; Female; Follow‐Up Studies; Gastrointestinal Agents [administration & dosage]; Humans; Incidence; Infliximab; Infusions, Intravenous; Male; Postoperative Care [*methods]; Postoperative Complications [epidemiology, etiology, *prevention & control]; Prospective Studies; Treatment Outcome; Tumor Necrosis Factor‐alpha [antagonists & inhibitors]

%X BACKGROUND: Infliximab is effective treatment for Crohn's disease and has been associated with rare, but serious infectious complications. Emerging data suggest a benefit of infliximab in preventing postoperative Crohn's disease recurrence. It is not known whether administration of infliximab shortly after resective surgery for Crohn's disease increases postoperative complications. AIMS: To evaluate the risk of developing postoperative complications among Crohn's disease patients receiving infliximab within 4 weeks of intestinal resection. METHODS: As part of a randomized placebo‐controlled infliximab postoperative prevention study, adverse events were prospectively monitored. Crohn's disease patients undergoing intestinal resection were randomized to placebo or infliximab 2‐4 weeks after surgery. Study infusions were administered at 0, 2, and 6 weeks then every 8 weeks for 1 year. To evaluate whether infliximab increased postoperative complications, we analyzed all adverse events for 1 year after surgery. RESULTS: Twenty‐four patients were randomized to infliximab or placebo after intestinal resection for Crohn's disease. Mean time to first postoperative infusion was 20 days (range 14‐25 days). Over the course of 1 year, there were 22 total adverse events, but no difference between infliximab and placebo patients (12 versus 10, respectively, P = 1.0). In the immediate postoperative period, within 8 weeks of surgery, the number of adverse events was also similar between the two groups (3 infliximab and 5 placebo patients, P = 0.68). There were no serious adverse events and no complications related to wound healing or infection. CONCLUSIONS: Initiation of infliximab within 4 weeks of intestinal resection was not associated with postoperative complications.

%Z Digestive diseases and sciences

Journal article

%U https://cochrane.66557.net/central/doi/10.1002/central/CN-00811247/full

%0 Journal Article

%A de Bruyn, J.

%A Bossuyt, P.

%A Ferrante, M.

%A West, R.

%A Dijkstra, G.

%A Witteman, B.

%A Franchimont, D.

%A van der Bilt, J. D.

%A Tollens, T.

%A Bemelman, W. A.

%A Et, Al.

%D 2019

%T POSTOPERATIVE ENDOSCOPIC AND CLINICAL RECURRENCE AFTER ILEOCOLONIC RESECTION IN PATIENTS WITH CROHN'S DISEASE CANNOT BE PREVENTED WITH HIGH DOSE VITAMIN D

%V 156

%N 6

%P S‐33

%8 2019-01-01

%R 10.1016/S0016-5085(19)36857-X

%K *Crohn disease; *relapse; Abscess; Adult; Adverse event; Anastomosis; Antiinflammatory activity; Aphthous stomatitis; Belgium; Conference abstract; Controlled study; Crohn Disease Activity Index; Drug megadose; Drug safety; Drug therapy; Endoscopist; Endoscopy; Ethnicity; European Quality of Life 5 Dimensions questionnaire; Female; Human; Human tissue; Ileum; Incidence; International standard unit; Major clinical study; Male; Multicenter study; Netherlands; Pharmacokinetics; Prospective study; Quality of life; Randomized controlled trial; Risk factor; Season; Short Form 36; Vitamin D deficiency; Wound infection

%X Introduction: Vitamin D deficiency is common amongst patients with Crohn's disease (CD). Previous preclinical experiments and one clinical trial suggested anti‐inflammatory effects of vitamin D in IBD. We performed a prospective placebo controlled clinical trial in patients with CD undergoing an ileocolonic resection with ileocolonic anastomosis in which we studied the potential anti‐inflammatory effects of vitamin D. Aims and Methods: This trial was performed in 17 centres in Belgium and the Netherlands. CD patients with at least 1 risk factor for recurrence were randomized to receive weekly 25.000 International Units (IU) of vitamin D3 (Cholecalciferol in 1 milliliter vials) or placebo for 6 months following their first or second ileocolonic resection. All other CD medication was stopped. The primary endpoint at week 26 was endoscopic recurrence defined as a modified Rutgeerts score ≥i2b (>5 aphthous ulcerations in the neoterminal ileum, with or without anastomotic lesions); secondary endpoints included clinical recurrence (Crohn's disease activity index (CDAI) ≥ 220), quality of life (SF‐36, IBD‐Q and EQ‐5D), safety and differential outcomes by baseline vitamin D serum concentrations. All endoscopies were centrally read and adjudicated by two expert blinded endoscopists. Results: 143 patients were randomized (72 to vitamin D and 71 to placebo); baseline patient characteristics were comparable between the two groups (mean age (±SD) 34 (±12) vs 37 (±15) years, and 38% vs 40% male, respectively). Serum 25‐OH vitamin D levels increased from median (IQR) 42 (27–56) nmol/L to 87 (73–105) nmol/L at week 26 in the intervention group (p=<0.00001), and remained unchanged at 43 (29–64) nmol/L in patients on placebo throughout the whole study. No difference was seen in the incidence and severity of endoscopic recurrence at 26 weeks between the two groups (Table 1). In addition, the cumulative clinical recurrence rates at week 26 were also comparable (Table 1). Quality of life as measured by SF‐36, IBD‐Q and EQ‐5D improved slightly over time in both groups but was not significantly different between the two groups. Adverse events were uncommon in either group; adverse events with an incidence >5% included abscess formation in both groups and wound infection in the placebo group, and were related to surgery. Outcome was not affected by baseline serum vitamin D level, season of inclusion, or ethnicity. Conclusion: High‐dose vitamin D treatment did not reduce the incidence of postoperative endoscopic and clinical recurrence in CD patients, despite normalization of serum 25‐OH vitamin D concentrations. Hence, vitamin D deficiency might merely be a consequence of disease activity rather than a causal explanation in the pathophysiology of CD. Endoscopic and clinical recurrence rates at week 26 [Table presented]

%Z Gastroenterology

Journal article; Conference proceeding

%U https://cochrane.66557.net/central/doi/10.1002/central/CN-01962812/full

%0 Journal Article

%A Meng, A.

%A Sharma, S.

%A Xin, Y.

%A Namour, F.

%A Mathias, A.

%D 2017

%T Population PK/PD modeling for evaluation of filgotinib efficacy in subjects with moderate to severe crohn's disease

%V 44

%N 1

%P S17

%8 2017-01-01

%R 10.1007/s10928-017-9536-y

%K Crohn Disease Activity Index; Crohn disease; adult; clinical trial; comparative effectiveness; controlled clinical trial; controlled study; disease simulation; drug effect; drug therapy; female; human; major clinical study; male; outcome assessment; phase 2 clinical trial; placebo effect; rate constant; recurrence risk

%X Objectives: Filgotinib (FIL) is a potent and selective inhibitor of JAK1, which is a therapeutic target for a range of inflammatory conditions including Crohn's Disease (CD). Severity of CD is assessed by the Crohn's Disease Activity Index (CDAI) score. A PK/ PD model describing the progression of CDAI was developed to evaluate the exposure‐efficacy relationship for FIL in CD patients. Methods: In a Phase II study, subjects received 200 mg FIL (N = 128) or placebo (N = 44) during first 10 weeks. Subsequently, based on Week 10 efficacy outcome, subjects were re‐assigned to receive 200 mg, 100 mg FIL or placebo through Week 20. Population PK of FIL has been characterized previously. Total systemic exposure (AUCtau) was incorporated to account for drug effect. PK/PD (CDAI score progression) analysis was conducted with NONMEM® v7.3 and R v3.3.2 used for processing/visualizing data. Results: CDAI score progression was characterized by a bi‐exponential function: CDAI = BL ∗ (1+ PLC ∗ (1 ‐ e‐k1∗TIME1 ‐ e‐k2∗TIME2)), where BL was the baseline, PLC was the magnitude of placebo effect, k1 was the onset rate constant described as kpl ∗ (1 + slope ∗ AUC), k2 was the relapse rate constant, TIME1 was time since first dose and TIME2 was time to relapse. The final model estimates (rse%) were ‐0.793 (3%) for PLC, 0.0169/day (12%) for kpl, 0.0142/day (11%) for k2, and 0.0262 mL/(ng∗h) (54%) for slope of drug effect. Inter‐individual variability (%CV) was 106% on kpl, 184% on slope, 45.2% for time when relapse occcurs and 13.5% for BL. Diagnostics showed that the model adequately described the observed CDAI scores. Conclusions: The PK/PD model characterized the progression of CD, and captured drug effect across different FIL treatment arms of the phase II study. This model can be applied to simulate various treatment paradigms for FIL in subjects with CD.

%Z Journal of pharmacokinetics and pharmacodynamics

Journal article; Conference proceeding

%U https://cochrane.66557.net/central/doi/10.1002/central/CN-01622517/full

%0 Journal Article

%A NCT

%D 2019

%T Polidocanol Foam Versus Rubber Band Ligation in the Treatment of Hemorrhoidal Disease

%8 2019-01-01

%K Hemorrhoids; Polidocanol

%X INTRODUCTION Hemorrhoids are normal vascular structures in the anal canal, arising from a cushion of dilated arteriovenous channels and connective tissues, that drains into the superior and inferior hemorrhoidal veins. Their main function is to maintain anal continence, serve as a protection for the anal sphincters during the act of defecation and have a sensory function, allowing to differentiate liquids, solids or gases and to signal defecation. The hemorrhoidal disease develops when the supporting tissues of hemorrhoidal cushions deteriorate due to various processes: abnormal venous dilation, vascular thrombosis, degenerative processes of collagen and fibroelastic tissue, distortion and rupture of anal subepithelial muscle, hyperperfusion of the hemorrhoidal plexus, inflammatory phenomena and hormonal changes (typical of pregnancy). Hemorrhoidal disease occurs frequently in the adult population and a considerable number of patients are asymptomatic. Both sexes are similarly affected. The peak incidence occurs between 45‐65 years, being rare before the age of 20. Classification of hemorrhoidal disease corresponds to its position relative to the dentate line. External hemorrhoids are located below the dentate line and are covered by modified squamous epithelium, being richly innervated and therefore painful when there is associated thrombosis. On the contrary, internal hemorrhoids lie above the dentate line. Internal hemorrhoids are further classified based on their appearance and degree of prolapse according to the Goligher classification: grade I, without prolapse (they have the potential to bleed but are not visualized without the aid of an anoscope); grade II, prolapsed with defecation, but reduced spontaneously; grade III, prolapse with defecation requiring manual reduction and grade IV, prolapsed and non‐reducible. Since hemorrhoidal disease is a benign pathology, its treatment should be guided by the symptoms and the impact of the disease on quality of life. A prospective study by Pucher et al. developed and validated the Sodergren scale, which is based on a set of symptoms to assess the severity of hemorrhoidal disease. This scale can be used to assess the efficacy of treatment of haemorrhoids and the comparison of trials, consequently it is helpful in the choice of the best therapeutic option. Treatment of hemorrhoidal disease can be divided into conservative measures, office‐based procedures and surgical treatments. First line therapy should be conservative and includes a set of lifestyle changes, dietary changes, laxative medication and phlebotonic and/or topical anti‐inflammatory drugs. These measures produce beneficial effects and should be implemented in every grades of hemorrhoidal disease or in patients undergoing instrumental or surgical treatment. The instrumental office‐based treatment is usually indicated for hemorrhoidal disease grade I and II, thought it can also be used in grade III hemorrhoidal disease. It is aimed at decreasing hemorrhoidal vascularization, reducing redundant tissue and increasing hemorrhoidal rectal wall fixation to minimize prolapse. It includes rubber band ligation, sclerotherapy (liquid and foam agents), infrared photocoagulation, cryotherapy and radiofrequency ablation. There have been no comparative studies between polidocanol foam sclerotherapy and rubber band ligation. The study was approved by the ethics committee of CHUP. All the participants enrolled sign an informed consent. For purposes of evaluating efficacy and safety, two distinct periods were considered: intervention and follow‐up. At all visits, a proctologic examination with anoscopy is performed and clinical evaluation is carried out by filling the Sodergren's scale of symptoms. The severity of bleeding is also evaluated. Rubber band ligation is the most commonly performed procedure in the office and is indicated for grade II and III internal hemorrhoids and works by causing hemorrhoid tissue necrosis and its fixation to the rectal mucosa. Complications associated with rubber band ligation include bleeding (ranging from mild to severe), pain, urinary symptoms, priapism, vagal symptoms, hemorrhoidal thrombosis, sepsis, fistulation or even death. Hemorrhage and pain are among the most frequent. Cumulatively, a success rate of 80% is observed with rubber band ligation. Hemorrhoidal sclerosis is a procedure commonly used to treat grade I and II hemorrhoidal disease. It has also been used in internal grade III hemorrhoids, although in these cases there is little scientific evidence supporting its efficacy. In this technique a needle is introduced through an endoscope or anoscope and the sclerosing agent is injected into the hemorrhoid above the dentated line ‐ Blanchard technique. There are a variety of sclerosing agents such as 5% phenol in vegetable oil, quinine, tetradecyl sodium sulfate, sodium morphate or potassium aluminum sulfate and tannic acid (ALTA). More recently, a new sclerosing substance, polidocanol, a non‐ionic detergent consisting of two components, a polar hydrophilic chain and a non‐polar hydrophobic, started to be employed in the treatment of hemorrhoidal disease. The experience of its use in sclerotherapy comes mainly from the treatment of varicose veins and it can be used in its liquid or foam form. Several studies reported the efficacy of the use of sclerotherapy with liquid polidocanol in hemorrhoidal disease; it is considered a sclerosing agent with anesthetic properties, well tolerated, with low necrotic potential and a very promising agent for the treatment of grade I hemorrhoidal disease. The foam formation is based on the Tessari technique which uses a device that combines two syringes and a three‐way tap in which the polidocanol is mixed with air under mechanical force ("Tourbillon technique"). This formulation allows for greater efficacy and use of lower doses of sclerosing agent since the volume will be greatly increased and hence also the area of contact with the vascular endothelium where the drug will exert its sclerosing action. The use of polidocanol foam in the treatment of varicose veins is safe and effective and has been shown to be superior to the use of liquid polidocanol. Its use is not indicated in cases of acute thromboembolism and allergy to polidocanol. There is only one study showing the superiority of polidocanol foam compared to its liquid formulation in the treatment of grade I hemorrhoidal disease. In a recently published non‐controlled study, 2000 patients with hemorrhoidal disease grades I to IV were treated with polidocanol foam and the authors concluded that this therapy was very successful, with 98% of the patients reporting satisfaction regarding bleeding control and prolapse reduction. Complications were rare and usually minor. There's a lack of studies comparing it with other ablative techniques. The most common complications of sclerotherapy include mild anal discomfort and bleeding. However, the bleeding risk is lower compared to that observed with rubber band ligation. Rare complications include erectile dysfunction, mucosal ulceration, necrosis, prostatic abscess, retroperitoneal sepsis and transient bacteremia. Sclerotherapy is a valid alternative for the treatment of patients whose hemorrhage is the main symptom and where conservative therapy has not been effective, as well as for patients on anticoagulant medication, and for cirrhotic or immunocompromised patients. With the present study, the investigators aim to fill a gap in the literature by evaluating the safety and efficacy in the treatment of hemorrhoidal disease with the seemingly most effective non‐surgical office‐based methods (rubber band ligation and sclerotherapy with polidocanol foam). METHODS PARTICIPANTS AND ETHICAL ASPECTS Are included patients referred to proctologic consultation of Centro Hospitalar Universitário do Porto (CHUP) older than 18 years with symptomatic hemorrhoidal disease grade I, II and III (Goligher's classification) refractory to conservative management (dietary modification, intestinal transit modifiers, topical and phlebotonic medications) for a period of no less than 4 weeks. All participants must have prior endoscopic study, at least recto‐sigmoidoscopy, or complete colonoscopy if they are older than 50 years or younger with family history of colorectal cancer, colon adenomas, or suspected inflammatory bowel disease. STATISTICAL ANALYSIS Sample size was determined considering a power of 80% (type II error ß of 20%) and significance level α of 5% (type I error). The required number in each treatment was 44 patients. In order to safeguard against potential drop‐outs, a sample size of 120 (60+60) patients was considered. It was generated a 1:1 randomization sequence, stratified by for the degree of hemorrhoidal disease (Goligher's classification), to assign participants to each one of the therapeutic arms either sclerotherapy with polidocanol foam or rubber band ligation. Since the two office‐based therapies under study have completely different techniques and procedures, it is not possible to blind either the patient or the clinician who apply the treatment. Therefore, an open label study is being conducted. VISITS AND DATA COLLECTION Demographic data such as age, sex, body mass index (BMI) are collected. In the first visit informed consent and an information brochure explaining the study and adequate dietary and behavioral care is provided. During the intervention period, when office‐based treatments are performed, patients are observed at 3‐week intervals (minimum of 3 weeks and maximum of 9 weeks depending on the number of instrumental treatments performed) and, during follow‐up period, every 3 months, 3 weeks after the last session of treatment to evaluate recurrence of hemorrhoidal disease (maximum 1 year; 4 visits). INTERVENTION PERIOD AND TECHNICAL ASPECTS The required number of sessions of any of the office‐based treatments (maximum of 3 sessions) is determined by clinical and anoscopy response i.e., if 3 weeks after the previous treatment the participant scores zero points in the Sodergren scale and has bleeding grade ≤1 or the anoscopy doesn't reveal significant hemorrhoidal disease, there is no place for additional instrumental therapy and the patient starts the follow‐up period. If there is therapeutic failure (participants that, at the end of three sessions of instrumental treatment, aggravate or maintain the initial Sodergren score and bleeding grade) or if there is a significant complication (moderate or severe) the patient's participation in the study ends and he is referred for treatment with other type of office‐based procedure or surgery.

%Z https://clinicaltrials.gov/show/NCT04091763

Trial registry record

%U https://cochrane.66557.net/central/doi/10.1002/central/CN-01984300/full

%0 Journal Article

%A Boland, K.

%A Haritunians, T.

%A Schumm, L. P.

%A McGovern, D.

%A Brant, S. R.

%A Rioux, J. L.

%A Sharma, Y.

%A Duerr, R.

%A Cho, J.

%A Silverberg, M.

%D 2017

%T Phenotypic predictors of endoscopic recurrence after ileal resection for Crohn's disease: an NIDDK IBD Genetics Consortium prospective study

%V 11

%N Supplement 1

%P S213‐S214

%8 2017-01-01

%R 10.1093/ecco-jcc/jjx002.394

%K *Crohn disease; *genetics; *ileum resection; *prospective study; *recurrence risk; Adult; Anastomosis; Biological product; Bivariate analysis; Chi square test; Classification; Cohort analysis; Colonoscopy; Conference abstract; Controlled clinical trial; Controlled study; Crohn disease; Drug combination; Drug therapy; Endogenous compound; Female; Follow up; Gender; Human; Longitudinal study; Major clinical study; Male; Microbiome; Monotherapy; Multicenter study; Nonhuman; Patient history of surgery; Phenotype; Smoking; Steroid; Terminal ileum; Tumor necrosis factor; Tumor necrosis factor inhibitor; adult; anastomosis; bivariate analysis; chi square test; classification; cohort analysis; colonoscopy; conference abstract; controlled clinical trial; controlled study; drug combination; drug therapy; female; follow up; gender; genetics; human; ileum resection; longitudinal study; major clinical study; male; microbiome; monotherapy; multicenter study; nonhuman; patient history of surgery; phenotype; prospective study; recurrence risk; smoking; terminal ileum

%X Background: Disease recurrence in patients after ileal resection of Crohn's Disease (CD) is predictable and represents an excellent model to study the mechanisms of intestinal inflammation in an at‐risk population. Our aim is to investigate genomic and microbial factors associated with post‐operative endoscopic recurrence (ER). Here we present preliminary phenotypic analysis of recruited subjects to a prospective NIDDK Inflammatory Bowel Disease Genetics Consortium longitudinal study. Methods: Patients with CD scheduled to undergo ileocolic resection with primary anastomosis were recruited at 6 North American research centres using a standardised protocol. Clinical data and bio specimen collection for microbiome and histological assessment was performed pre‐operatively and at follow up. A Rutgeert's score of at least i2 determined endoscopic recurrence. Bivariate analysis (χ2 test) was performed using Graphpad. Results: 294 patients were enrolled up to August 2016 and 122 had at least 1 post‐operative endoscopy. The overall recurrence rate in the neo‐terminal ileum up to 18 months was 33.6% (n=41/122). Early ER was present in 23.7% (n=29/122) at a median 6 months. CD recurrence was not significantly associated with Montreal classification, age, gender, smoking, or previous hospitalisations. Patients with a prior history of ileal resection had a higher risk of post‐operative ER (p=0.004, RR 2.6 95% CI [1.5‐3.8], n=9/41 vs n=3/81). Peri operative steroids (p=0.002, RR 3.4 95% CI [1.46‐8.9]), combined immune suppressants and anti‐TNF agents (p=0.028) and anti‐TNF monotherapy use (p=0.056, RR 1.03 95% CI [1.002‐4.04]) were associated reduced likelihood of ER. Use of anti‐TNF therapy post‐operatively was also associated with reduced recurrence (p=0.03, RR 2.81 95% CI [1.18‐7.3], 15.7%, n=6/38 vs 41.6%, n=35/84). Patients recruited in the USA were more likely to receive anti‐TNF therapy prior to first post‐operative endoscopy (p=0.02, RR 2 95% CI l1.4‐3.6]). Early recurrence rates were higher in Canadian centres al‐though this was not statistically significant (p=0.45 [20% vs 40%]). Conclusions: Preliminary phenotypic results showed that previous surgery predicted endoscopic post‐operative recurrence, potentially indicating a more aggressive phenotype. Steroid exposure periop‐eratively and use of anti‐TNF biologic therapy peri‐and post‐operatively before colonoscopy were associated with lower risk of endoscopic recurrence, validating studies which show benefit of anti‐TNF in prevention of post‐operative recurrence. Future studies in this population will investigate microbial and transcriptomic profiles related to disease recurrence and ongoing recruitment will further expand our cohort.

%Z Journal of Crohn's & colitis

Journal article; Conference proceeding

%U https://cochrane.66557.net/central/doi/10.1002/central/CN-01469853/full

%0 Journal Article

%A EUCTR DE

%D 2017

%T Phase II clinical trial, conducted in different sites, with random assignation of treatment, where neither the patient or the medical doctor know the assigned treatment, drug or placebo, to evaluate the efficacy and safety of Rifaximin delayed release 400 mg tablet in the prevention of post-operative endoscopic Crohn’s disease recurrence

%8 2017-01-01

%X INTERVENTION: Product Name: Rifaximin delayed release 400 mg film coated tablet Product Code: Rifaximin‐EIR Pharmaceutical Form: Coated tablet INN or Proposed INN: RIFAXIMIN CAS Number: 80621‐81‐4 Current Sponsor code: Rifaximin‐EIR Other descriptive name: RIFAXIMINA Concentration unit: mg milligram(s) Concentration type: equal Concentration number: 400‐ Pharmaceutical form of the placebo: Coated tablet Route of administration of the placebo: Oral use CONDITION: Post‐operative endoscopic Crohn’s disease recurrence ; MedDRA version: 20.0 Level: LLT Classification code 10013099 Term: Disease Crohns System Organ Class: 100000004856 Therapeutic area: Diseases [C] ‐ Digestive System Diseases [C06] PRIMARY OUTCOME: Main Objective: To demonstrate the efficacy of Rifaximin –EIR 400 mg Tablet (800mg /BID, total daily dose 1600 mg) versus placebo in the prevention of endoscopic Crohn’s disease recurrence following ileocolonic resection. Primary end point(s): The primary end point of this trial will be the proportion of patients with endoscopic recurrence at 26 weeks after randomization defined as Rutgeerts score = i2 (with central blinded reading of video ileocolonoscopies). Secondary Objective: To demonstrate the efficacy of Rifaximin‐ EIR 400 mg Tablet (800mg /BID, total daily dose 1600 mg) versus placebo in the maintenance of clinical remission.; To evaluate the safety profile of Rifaximin‐EIR 400 mg Tablet (800mg /BID, total daily dose 1600 mg); Assess the effects of Rifaximin‐EIR treatment on biological (inflammatory) markers of disease.; Evaluate the effects of Rifaximin ‐EIR treatment on quality of life Timepoint(s) of evaluation of this end point: 26 weeks SECONDARY OUTCOME: Secondary end point(s): ? Patients with endoscopic modified Rutgeerts score = 2a (lesions limited to the anastomosis or maximum of 5 aphthous lesions in the neo‐terminal ileum with normal mucosa between the lesions) at 26 weeks after randomization. ; ; ? Clinical recurrence according to CDAI score (defined as an increase higher than 70 points in CDAI score from baseline and CDAI>220 points; CDAI will be assessed at each visit). ; ; ? Proportion of patients with clinical symptom remission according to PRO defined by (1) the total number of liquid/very soft stools for the 7 days prior to each visit being = 10 (from CDAI Item 1); AND (2) an abdominal pain rating of = 1(from CDAI Item 2) on each day for the 7 days prior to each visit. ; ; ? Development of a new/re‐draining fistula or abscess by week 26 ; ; ? Time to clinical recurrence ; ; ? Change from baseline in CRP and faecal calprotectin at each visit during the Treatment Period. ; ; ? Change from baseline in indices of health outcomes (SF‐36®) at Week 26. ; ; ? Assessments of safety (vital signs, routine laboratory parameters, adverse events, withdraw due to adverse events) ; Timepoint(s) of evaluation of this end point: At week26 ; 4‐8‐16‐26‐28 weeks ; 4‐8‐16‐26‐28 weeks ; At week26 ; At week26 ; 4‐8‐16‐26‐28 weeks ; 4‐8‐16‐26‐28 weeks ; At randomization visit, week 4‐8‐16‐26‐28 INCLUSION CRITERIA: ? Patients of both sexes aged between 18 and 75 years old, inclusively. ? Patients with diagnosis of CD, who had undergone curative ileocolonic resection, with ileocolonic anastomosis and for whom a randomization within 45 days from surgical intervention is feasible. ? If the patients had an end or loop ileostomy within 1 year prior to randomization, they can be included, but stoma closure should occur within 45 days prior to randomization. ? Patients have had faecal stream restoration at least 14 days prior to randomization. ? Patients must have at least one of the following risk factors for the development of early/severe post‐operative endoscopic recurrence of their CD: 1) qualifying surgery that was their second intestinal resection within 10 years 2) third or more intestinal resections, 3) surgery for a penetrating CD complication (e.g., abscess or fistula), or 4) smoking 10 or more cigarettes per day for the past year. <

%Z https://trialsearch.who.int/Trial2.aspx?TrialID=EUCTR2017-002258-36-DE

Trial registry record

%U https://cochrane.66557.net/central/doi/10.1002/central/CN-01886439/full

%0 Journal Article

%A EUCTR BE

%D 2017

%T Phase II clinical trial, conducted in different sites, with random assignation of treatment, where neither the patient or the medical doctor know the assigned treatment, drug or placebo, to evaluate the efficacy and safety of Rifaximin delayed release 400 mg tablet in the prevention of post-operative endoscopic Crohn’s disease recurrence

%8 2017-01-01

%X INTERVENTION: Product Name: Rifaximin delayed release 400 mg film coated tablet Product Code: Rifaximin‐EIR Pharmaceutical Form: Coated tablet INN or Proposed INN: RIFAXIMIN CAS Number: 80621‐81‐4 Current Sponsor code: Rifaximin‐EIR Other descriptive name: RIFAXIMINA Concentration unit: mg milligram(s) Concentration type: equal Concentration number: 1600‐ Pharmaceutical form of the placebo: Coated tablet Route of administration of the placebo: Oral use CONDITION: Post‐operative endoscopic Crohn’s disease recurrence ; MedDRA version: 20.0 Level: LLT Classification code 10013099 Term: Disease Crohns System Organ Class: 100000016693 Therapeutic area: Diseases [C] ‐ Digestive System Diseases [C06] PRIMARY OUTCOME: Main Objective: To demonstrate the efficacy of Rifaximin –EIR 400 mg Tablet (800mg /BID, total daily dose 1600 mg) versus placebo in the prevention of endoscopic Crohn’s disease recurrence following ileocolonic resection. Primary end point(s): The primary end point of this trial will be the proportion of patients with endoscopic recurrence at 26 weeks after randomization defined as Rutgeerts score =i2. Secondary Objective: To demonstrate the efficacy of Rifaximin‐ EIR 400 mg Tablet (800mg /BID, total daily dose 1600 mg) versus placebo in the maintenance of clinical remission.; To evaluate the safety profile of Rifaximin‐EIR 400 mg Tablet (800mg /BID, total daily dose 1600 mg); Assess the effects of Rifaximin‐EIR treatment on biological (inflammatory) markers of disease.; Evaluate the effects of Rifaximin ‐EIR treatment on quality of life Timepoint(s) of evaluation of this end point: 26 weeks SECONDARY OUTCOME: Secondary end point(s): ? Patients with endoscopic Rutgeerts score <i2a (lesions limited to the anastomosis or maximum of 5 aphthous lesions in the neo‐terminal ileum with normal mucosa between the lesions) at 26 weeks after randomization.; ; ? Clinical recurrence according to CDAI score (defined as an increase higher than 70 points in CDAI score from baseline and CDAI>220 points; CDAI will be assessed at each visit).; ; ? Proportion of patients with clinical symptom remission according to PRO defined by (1) the total number of liquid/very soft stools for the 7 days prior to each visit being = 10 (from CDAI Item 1); AND (2) an abdominal pain rating of = 1(from CDAI Item 2) on each day for the 7 days prior to each visit.; ; ? Development of a new/re‐draining fistula or abscess by week 26; ; ? Time to clinical recurrence; ; ? Change from baseline in CRP and faecal calprotectin at each visit during the Treatment Period.; ; ? Change from baseline in indices of health outcomes (SF‐36®) at Week 26.; ; ? Assessments of safety will be based on the following:; ‐ vital signs (including blood pressure, pulse, temperature and body weight);; ‐ routine laboratory parameters (haematology, chemistry, urinalysis);; ‐ adverse events;; ‐ withdraw due to adverse events (AEs).; ; Timepoint(s) of evaluation of this end point: At week26; 4‐8‐16‐26‐28 weeks; 4‐8‐16‐26‐28 weeks; At week26; At week26; 4‐8‐16‐26‐28 weeks; 4‐8‐16‐26‐28 weeks; At randomization visit, week 4‐8‐16‐26‐28 INCLUSION CRITERIA: ? Patients of both sexes aged between 18 and 75 years old, inclusively. ? Patients with diagnosis of CD, who had undergone curative ileocolonic resection, with ileocolonic anastomosis and for whom a randomization within 45 days from surgical intervention is feasible. ? If the patients had an end or loop ileostomy within 1 year prior to randomization, they can be included, but stoma closure should occur within 45 days prior to randomization. ? Patients have had faecal stream restoration at least 14 days prior to randomization. ? Patients must have at least one of the following risk factors for the development of early/severe post‐operative endoscopic recurrence of their CD: 1) qualifying surgery that was their second intestinal resection within 10 years 2) third or more intestinal resections, 3) surgery for a penetrating CD complication (e.g., abscess or fistula), or 4) smoking 10 or more cigarettes per day for the past year. ?

%Z https://trialsearch.who.int/Trial2.aspx?TrialID=EUCTR2017-002258-36-BE

Trial registry record

%U https://cochrane.66557.net/central/doi/10.1002/central/CN-01905727/full

%0 Journal Article

%A NCT

%D 2013

%T Perianal Abscess Packing Randomized ControlledTrial Pilot Study

%8 2013-01-01

%K Abscess

%X 1. INTRODUCTION 1.1 BACKGROUND Perianal abscesses are common with an incidence of 0.5‐1%. Some present as emergencies and all require surgery, placing a significant burden on health resources. The mainstay of management is incision and drainage. Traditionally the residual cavity is then packed. On discharge, the cavity packing requires frequent changing. This uses considerable community nursing resource. Perianal abscesses can alternatively be treated by primary closure or without packing the cavity. Benefits of treating without packing include greater patient comfort and acceptance and reduced nursing requirement. However treating without packing is not yet widely accepted, in the absence of sufficient evidence that it is as safe and effective. This study aims to address this issue. 1.2 RATIONALE FOR CURRENT STUDY ‐ Question: In patients with perianal abscesses, does incision and drainage without packing the subsequent cavity reduce patient discomfort without increasing healing time or recurrence compared with management involving cavity packing? ‐ Hypothesis: Perianal abscess can be managed without cavity packing, with no increase in healing time or recurrence. ‐ Note this was partly addressed by Tonkin et al (2004) but their study was underpowered. We intend to have a sufficiently powered study to definitively answer the question. 2. STUDY OBJECTIVES ‐ Assess whether there is any statistically significant difference in patients with perianal abscesses managed with and without cavity packing in terms of: ‐ Length of hospital stay ‐ Time to cavity healing ‐ Recurrent abscess or fistula formation ‐ Pain score ‐ Analgesia usage 3. STUDY DESIGN ‐ This is a randomised controlled trial. ‐ Due to the nature of the intervention (packing), it is impossible for either the subjects or the research team to be blinded. ‐ Duration: recruitment into the study will remain open until the target number of subjects has been reached. ‐ Number and type of subjects: power calculations from a published pilot study show that to demonstrate a difference of 10% vs 20% in e.g. fistula and recurrent abscess formation, 316 patients need to be recruited. We will perform a pilot study of 20 patients in each group and at this time calculate the number of subjects to be recruited 3.1 STUDY OUTCOME MEASURES ‐ Length of hospital stay ‐ Time to cavity healing ‐ Recurrent abscess or fistula formation ‐ Pain score ‐ Analgesia usage 8.2 CONFIDENTIALITY The Chief Investigator will preserve the confidentiality of participants taking part in the study and is registered under the Data Protection Act. 8.3 AUDITS The study may be subject to inspection and audit by Imperial College London under their remit as sponsor and other regulatory bodies to ensure adherence to GCP and the NHS Research Governance Framework for Health and Social Care (2nd edition). 9. STUDY MANAGEMENT The day‐to‐day management of the study will be coordinated through Mr Mikael Sodergren. 10. PUBLICATION POLICY All publications and presentations relating to the study will be authorized by the Trial Management Group (TMG). The first publication of the trial results will be in the name of the Trial Management Group, or appropriately names authors. If there are named authors, these will include at least the trial's Chief Investigator, and Trial Coordinator. Members of the TMG will be listed and contributors will be cited by name if published in a Joint Research Office journal where this does not conflict with the journal's policy. Authorship of parallel studies initiated outside of the Trial Management Group will be according to the individuals involved in the project but must acknowledge the contribution of the Trial Management Group and the Study Coordination Centre. 4. PARTICIPANT ENTRY 4.1 PRE‐REGISTRATION EVALUATIONS ‐ Documentation of the duration of symptoms before presentation. ‐ Examination and documentation of abscess characteristics (can be done at the time of the operation): size, site and type. ‐ Comorbidities 4.2 INCLUSION CRITERIA ‐ Perianal abscess. ‐ Over 18 years of age. 4.3 EXCLUSION CRITERIA ‐ Under 18 years of age. ‐ Those unable to give informed consent. ‐ Abscesses associated with Crohn's disease or other underlying causes. ‐ Abscesses in which initial drainage is considered inadequate (if the skin is not open sufficiently to allow drainage of the abscess cavity). 4.4 WITHDRAWAL CRITERIA ‐ If the patient wants to withdraw from the study at any point they can do so and resume standardized treatment pathways 5. ADVERSE EVENTS 5.1 DEFINITIONS Adverse Event (AE): any untoward medical occurrence in a patient or clinical study subject. Serious Adverse Event (SAE): any untoward and unexpected medical occurrence or effect that: • Results in death ‐ Is life‐threatening ‐ refers to an event in which the subject was at risk of death at the time of the event; it does not refer to an event which hypothetically might have caused death if it were more severe ‐ Requires hospitalization, or prolongation of existing inpatients' hospitalization ‐ Results in persistent or significant disability or incapacity ‐ Is a congenital anomaly or birth defect Medical judgement should be exercised in deciding whether an AE is serious in other situations. Important AEs that are not immediately life‐threatening or do not result in death or hospitalization but may jeopardize the subject or may require intervention to prevent one of the other outcomes listed in the definition above, should also be considered serious. 5.3 REPORTING PROCEDURES All adverse events should be reported. Depending on the nature of the event the reporting procedures below should be followed. Any questions concerning adverse event reporting should be directed to the Chief Investigator in the first instance. 6. ASSESSMENT AND FOLLOW‐UP ‐ Patients in the packing group will have their dressing changed day 1 post‐operatively. On discharge they will have their care transferred to the District Nurses for daily dressing care in their own home. ‐ Patients in the non‐packing group will have the initial haemostatic dressing removed day 1 post‐operatively and be discharged with a superficial protective dressing to absorb any discharge and protect the wound. ‐ All patients will be reviewed at 2 weekly intervals in the outpatient clinic until the cavity is closed and the skin completely re‐epithelialized. At outpatient appointments, patients will be asked to score their pain over the previous two weeks on a standard 10cm Visual Analogue Scale for pain. ‐ Patients who do not attend clinic will be interviewed by telephone to gather the information listed above. ‐ The end point is patient discharge from the outpatient clinic with complete healing (as described above) or the development of a fistula or recurrent abscess. 7. STATISTICS AND DATA ANALYSIS ‐ Data and all appropriate documentation will be stored for a minimum of 5 years after the completion of the study, including the follow‐up period. ‐ The data will be tested for normal distribution and analyzed accordingly: o If it is normally distributed, an unpaired T‐test will be performed on the continuous data (time to healing, length of stay, pain score, morphine dose) and a Fischer test will be performed on the categorical data (fistula rates, recurrence, delayed healing). ‐ If it is not normally distributed, a Mann‐Whitney U test will be performed in place of the T‐test. ‐ The tests will be two‐tailed with a significance level of 0.05. ‐ Following a pilot study of 40 patients we will perform a power calculation however a similar pilot study already published indicates the need for 316 subjects to be recruited 8.1 CONSENT Consent to enter the study must be sought from each participant only after a full explanation has been given, an information leaflet offered and time allowed for consideration. Signed participant consent should be obtained. The right of the participant to refuse to participate without giving reasons must be respected. After the participant has entered the study the clinician remains free to give alternative treatment to that specified in the protocol at any stage if he/she feels it is in the participant's best interest, but the reasons for doing so should be recorded. In these cases the participants remain within the study for the purposes of follow‐up and data analysis. All participants are free to withdraw at any time from the protocol treatment without giving reasons and without prejudicing further treatment.

%Z https://clinicaltrials.gov/show/NCT01853267

Trial registry record

%U https://cochrane.66557.net/central/doi/10.1002/central/CN-02025162/full

%0 Journal Article

%A Timmer, A.

%A Sutherland, L. R.

%A Martin, F.

%D 1998

%T Oral contraceptive use and smoking are risk factors for relapse in Crohn's disease. The Canadian Mesalamine for Remission of Crohn's Disease Study Group

%V 114

%N 6

%P 1143‐1150

%8 1998-01-01

%R 10.1016/s0016-5085(98)70419-6

%K Adult; Aged; Aged, 80 and over; Cohort Studies; Contraceptives, Oral [*adverse effects]; Crohn Disease [*etiology]; Double‐Blind Method; Female; Humans; Life Tables; Male; Middle Aged; Prospective Studies; Recurrence; Risk Factors; Smoking [*adverse effects]

%X BACKGROUND & AIMS: Lifestyle factors have been shown to influence prognosis in Crohn's disease. The purpose of this study was to prospectively assess the effects of smoking and oral contraceptive use on clinical relapse rates. METHODS: Placebo‐treated patients formed a prospective cohort, followed up for 48 weeks or until relapse. The influence of smoking and the use of oral contraceptives on relapse risk was examined by life‐table analysis (log rank tests) and Cox proportional hazards modeling, taking into account demographic and disease characteristics. RESULTS: Of 152 patients, 61 (40%) had a relapse. Univariate analysis showed unfavorable outcomes for women (P = 0.05), current smokers (P = 0.005), and use of oral contraceptives (P = 0.001). Recent surgery was associated with a decreased risk of relapse (P = 0.02). The Cox model retained current smoking vs. never smoking (hazard ratio, 2.1; 95% confidence interval, 1.1‐4.2), oral contraceptive use (hazard ratio, 3.0; 95% confidence interval, 1.5‐5.9), and medical compared with surgical induction of remission (hazard ratio, 2.1; 95% confidence interval, 1.0‐4.2) as predictors of relapse. Ex‐smokers did not have an increased risk. Finally, sex, age, time in remission, disease location, and disease duration were not significant predictors. CONCLUSIONS: Oral contraceptive use and smoking are associated with an increased risk of relapse in patients with Crohn's disease.

%Z Gastroenterology

Journal article

%U https://cochrane.66557.net/central/doi/10.1002/central/CN-00684291/full

%0 Journal Article

%A Kamm, M. A.

%A De Cruz, P. P.

%A Wright, E. K.

%A Hamilton, A. L.

%A Ritchie, K. J.

%A Krejany, E. O.

%A Gorelik, A.

%A Liew, D.

%A Prideaux, L.

%A Lawrance, I. C.

%A Et, Al.

%D 2014

%T Optimising post-operative Crohn's disease management: best drug therapy alone versus endoscopic monitoring, disease evolution, and faecal calprotectin monitoring. The POCER study

%V 8

%P S13

%8 2014-01-01

%K *calgranulin; *colitis; *disease course; *disease management; *drug therapy; *monitoring; Adalimumab; Colonoscopy; Crohn disease; Disease control; Healing; High risk patient; Hospital; Human; Immunosuppressive treatment; Intestine resection; Mercaptopurine; Metronidazole; Patient; Recurrent disease; Remission; Risk; Smoking; colitis; colonoscopy; disease control; disease course; disease management; drug therapy; healing; high risk patient; hospital; human; immunosuppressive treatment; intestine resection; monitoring; patient; recurrent disease; remission; risk; smoking

%X Background: Disease recurs in most Crohn's disease patients after intestinal resection, with endoscopic recurrence preceding clinical recurrence. We investigated (i) whether early endoscopic monitoring with treatment step‐up for endoscopic recurrence is superior to standard drug therapy alone; (ii) disease evolution under optimal drug therapy is it possible to regain remission after endoscopic recurrence and is ongoing monitoring needed after early remission? (iii) whether faecal calprotectin (FC) can substitute for endoscopic monitoring. Methods: This IIS Post‐Operative Crohn's Endoscopic Recurrence (POCER) treat‐to‐target study aimed for mucosal healing. All patients received 3 months metronidazole. High risk patients (smoker, perforating disease, ≥2nd operation) also received daily thiopurine, or adalimumab if thiopurine intolerant. Patients were randomised 2:1 to colonoscopy at 6 months (“active care”) or no colonoscopy (“standard care”). Endoscopic remission was defined as Rutgeerts score i0 or i1 and recurrence as ≥i2. For endoscopic recurrence at 6 months low risk patients stepped up to thiopurine, high risk patients stepped up to adalimumab fortnightly, and high risk thiopurine‐intolerant patients stepped up to weekly adalimumab. All patients were colonoscoped at 18 months, scored centrally blind to treatment, with primary end‐point endoscopic recurrence at 18 months. FC (319 samples) CRP and CDAI were measured pre‐operatively, and at 6, 12, & 18 months. Results: 174 patients (83% high risk) in 21 hospitals enrolled. Of 122 active care patients 39% underwent 6 month treatment step‐up. 18 months endoscopic recurrence occurred in 49% active care v 67% standard care patients (P = 0.028). Step up at 6 months brought 38% of patients with endoscopic recurrence into remission 1 year later; conversely endoscopic disease recurred 1 year later in 41% of patients who were in remission at 6 months. FC correlated with endoscopic recurrence (r =‐0.42, p < 0.001) and score (r =‐0.44, p < 0.001); CRP and CDAI did not. FC >100mcg/g indicated endoscopic recurrence with a sensitivity 0.89 and NPV 91%, potentially allowing avoidance of colonoscopy in 41% of patients. Conclusions: Treating according to risk of recurrence, with early colonoscopy and treatment step‐up for recurrence, is superior to optimal drug therapy alone in preventing postop disease recurrence. Selective immunosuppression, with colonoscopy‐based adjustment, rather than its use in all high risk patients, leads to effective disease control in a majority. Early endoscopic remission requires ongoing monitoring. FC can be used to monitor for recurrence and is superior to CRP and CDAI.

%Z Journal of Crohn's & colitis

Journal article; Conference proceeding

%U https://cochrane.66557.net/central/doi/10.1002/central/CN-01057533/full

%0 Journal Article

%A Reibetanz, J.

%A Germer, C. T.

%D 2015

%T Optimal management of Crohn's disease after intestinal resection

%V 86

%N 11

%P 1070

%8 2015-01-01

%R 10.1007/s00104-015-0094-9

%K Adalimumab [administration & dosage]; Colonoscopy [methods]; Combined Modality Therapy; Crohn Disease [*surgery]; Humans; Metronidazole [administration & dosage]; Postoperative Care; Postoperative Complications [*surgery]; Recurrence; Risk Factors

%Z Der Chirurg; Zeitschrift fur alle Gebiete der operativen Medizen

Journal article

%U https://cochrane.66557.net/central/doi/10.1002/central/CN-01200760/full

%0 Journal Article

%A Feagan, B. G.

%A Sandborn, W. J.

%A Mittmann, U.

%A Bar-Meir, S.

%A D'Haens, G.

%A Bradette, M.

%A Cohen, A.

%A Dallaire, C.

%A Ponich, T. P.

%A McDonald, J. W.

%A Et, Al.

%D 2008

%T Omega-3 free fatty acids for the maintenance of remission in Crohn disease: the EPIC Randomized Controlled Trials

%V 299

%N 14

%P 1690‐1697

%8 2008-01-01

%R 10.1001/jama.299.14.1690

%K Adult; Crohn Disease [physiopathology, *prevention & control]; Double‐Blind Method; Fatty Acids, Omega‐3 [*therapeutic use]; Female; Glucocorticoids [therapeutic use]; Humans; Male; Middle Aged; Remission Induction; Secondary Prevention

%X CONTEXT: Maintenance therapy for Crohn disease features the use of immunosuppressive drugs, which are associated with an increased risk of infection. Identification of safe and effective maintenance strategies is a priority. OBJECTIVE: To determine whether the oral administration of omega‐3 free fatty acids is more effective than placebo for prevention of relapse of Crohn disease. DESIGN, SETTING, AND PATIENTS: Two randomized, double‐blind, placebo‐controlled studies (Epanova Program in Crohn's Study 1 [EPIC‐1] and EPIC‐2) conducted between January 2003 and February 2007 at 98 centers in Canada, Europe, Israel, and the United States. Data from 363 and 375 patients with quiescent Crohn disease were evaluated in EPIC‐1 and EPIC‐2, respectively. INTERVENTIONS: Patients with a Crohn's Disease Activity Index (CDAI) score of less than 150 were randomly assigned to receive either 4 g/d of omega‐3 free fatty acids or placebo for up to 58 weeks. No other treatments for Crohn disease were permitted. MAIN OUTCOME MEASURE: Clinical relapse, as defined by a CDAI score of 150 points or greater and an increase of more than 70 points from the baseline value, or initiation of treatment for active Crohn disease. RESULTS: For EPIC‐1, 188 patients were assigned to receive omega‐3 free fatty acids and 186 patients to receive placebo. Corresponding numbers for EPIC‐2 were 189 and 190 patients, respectively. The rate of relapse at 1 year in EPIC‐1 was 31.6% in patients who received omega‐3 free fatty acids and 35.7% in those who received placebo (hazard ratio, 0.82; 95% confidence interval, 0.51‐1.19; P = .30). Corresponding values for EPIC‐2 were 47.8% and 48.8% (hazard ratio, 0.90; 95% confidence interval, 0.67‐1.21; P = .48). Serious adverse events were uncommon and mostly related to Crohn disease. CONCLUSION: In these trials, treatment with omega‐3 free fatty acids was not effective for the prevention of relapse in Crohn disease. TRIAL REGISTRATION: clinicaltrials.gov Identifiers: EPIC‐1: NCT00613197, EPIC‐2: NCT00074542.

%Z JAMA

Journal article

%U https://cochrane.66557.net/central/doi/10.1002/central/CN-00631206/full

%0 Journal Article

%A NCT

%D 2023

%T Natural Orifice Specimen Extraction Surgery for Colorectal Cancer

%8 2023-01-01

%K Colorectal Neoplasms

%X Endpoints (Outcome measure)： Primary endpoint: The primary outcome measure was the postoperative inflammatory response, which was evaluated by monitoring the C‐reactive protein (CRP) level during hospitalization on the 3rd day following surgery. Early morbidity and mortality rate (postoperative 30 days): The early morbidity and mortality rate is defined as the event observed during the operation and within 30 days after surgery. Postoperative 30‐day hospital readmission data will be also collected. 1. Secondary endpoints: A. Duration of Operation time: The length of the surgery will be recorded. B.Peritoneal Cytologyduring surgery: The investigators assess the tumor cells identified by peritoneal cytologic specimens. Given the relationship of positive cytology with metachronous peritoneal seeding, it is essential to evaluate datasets from patients who undergo the NOSE group. C. Peritoneal Contamination during surgery: Peritoneal fluid samples were collected under sterile circumstances at the end of the surgery and sent for aerobic and anaerobic cultures. The investigators evaluate the contamination rate of peritoneal fluid in the two groups. D.Postoperative Pain Score: Pain intensity is assessed using a Numeric Rating Scale (NRS) with scores from 0 to 10 (10 = the worst pain). The highest pain scores of patients on each day for three consecutive days postoperatively will be recorded for further evaluation. E.Postoperative Recovery course: Time to first flatus passage Time to the first liquid diet Time to the first soft diet The length of hospital stays F.Number of retrieved lymph nodes G.Recurrence incidence and pattern 2. Exploratory endpoints (if any): Long‐term outcome: Overall survival Disease‐free survival Cancer‐specific survival Inclusion/Exclusion Criteria： Patient Enrollment Histological or cytological confirmation of colorectal adenocarcinoma. Inclusion criteria 1. Age ≥ 18 2. Performance status of 0 ‐ 2 on the ECOG (Eastern Cooperative Oncology Group) scale 3. American Society of Anesthesiology (ASA) score is Ⅰ‐Ⅲ 4. Tumor location: CRC with the lower margin of the tumor greater than 10 cm from the anal verge 5. Pre‐operative T staging: T0‐T4a at preoperative evaluation according to the American Joint Committee on Cancer (AJCC) Cancer Staging Manual 8th Edition 6. Preoperative M staging: M0 according to AJCC 8th 7. Tumor size: 4 cm or less 8. Written informed consent for participation Exclusion criteria (1) Not suitable for minimally invasive surgery (2) Body mass index (BMI) >30 kg/m2 (3) Malnutrition: albumin level less than 3.5 (4) Previous pelvic surgery (5) Emergency surgery Study Procedures： Randomization Randomization will be performed in the operating room at the Colorectal division, Linkou Chang Gung Memorial Hospital. Following the induction of minimally invasive surgery, an independent research assistant randomly assigned patients to undergo either NOSE surgery or conventional laparoscopic mini‐laparotomy resection by sealed‐envelope randomization. To ensure every group is similar in terms of covariates, especially the operation method (including right hemicolectomy, left hemicolectomy, and anterior resection). Randomization assignment is performed by the statisticians of the clinical trial center to generate random codes. Minimally invasive surgery Minimally invasive surgery will be performed in all operations, including multi‐port laparoscopic surgery and robotic surgery. After the segment of bowel resection, the strategy for surgical specimen removal is according to the result of randomization. # Conventional laparoscopy group: The investigators can select either the intracorporeal or extracorporeal method to create bowel anastomoses. For the extracorporeal way, a mini‐laparotomy wound is created and exteriorizes the bowel to do the anastomosis. The specimen is removed via the mini‐laparotomy wound after the anastomosis is accomplished for the intracorporeal approach. # NOSE group: After bowel resection, all bowel anastomoses are created via side‐to‐side intracorporeal anastomosis, either isoperistaltic or antiperistaltic. The surgical steps of NOSE with the transrectal method are illustrated in Figure 1. First, the rectosigmoid colonic lumen is blocked with a bowel clamp. After rectal irrigation with povidone‐iodine water, a transanal endoscopic microsurgery (TEM) scope or Alexis wound protector is inserted through the anus, reaching the upper rectum. Enterotomy is performed at the upper rectum, and a suction device is used to clean any fecal spillage. The TEM scope is pushed forward beyond the rectal opening, and the specimen is extracted with the TEM scope. The rectal opening is closed with a barbed suture, and an air leak test is performed to identify anastomotic leakage. Intra‐operative evaluation Peritoneal lavage with 50 ml of normal saline on the Douglas pouch and the sub‐phrenic area will be performed after the bowel anastomosis and before the abdominal wound closure. The estimated time would be 5 to 10 minutes. No additional risk will occur during the procedure. The investigators will do the peritoneal lavage fluid analysis in the following two phases: ‐ Peritoneal washing cytology (PWC) Peritoneal washing cytology (PWC) is a helpful indicator of peritoneal surface involvement and peritoneal dissemination of colorectal cancer. It may identify subclinical peritoneal spread and thus provide prognostic information. PWC is a useful prognostic tool in patients undergoing curative surgery for colorectal cancer since positive PWC was shown to be a potential risk factor for recurrence. The investigators will compare the positive rate of peritoneal cytology in the two groups. ‐ Peritoneal fluid bacterial culture: Although minimally invasive surgery is performed with standard procedure, contamination is inconceivable to avoid. In the NOSE group, the rectum is opened and exposed to the peritoneal cavity, and bacterial contamination is inevitable. After finishing the anastomosis in each group, a microbiological sample is obtained from the peritoneal fluid specimens. The investigators will collect the data and analyze the correlation between NOSE and wound infection and intra‐abdominal infection rates. Post‐operative assessment The postoperative outcome will be analyzed as below: 1. Postoperative complications The complication within 30 days will be recorded and categorized according to the Clavien‐Dindo classification. 2. Bowel function recovery The investigators will record and analyze the time to the first flatus passage, the first liquid diet, and the time to a soft diet. 3. The length of hospital stays The length of hospital stays is calculated from the date of operation to the discharge date. 4. Second operation and Readmission Reoperation and unplanned readmission will be recorded.

%Z https://clinicaltrials.gov/show/NCT05740267

Trial registry record

%U https://cochrane.66557.net/central/doi/10.1002/central/CN-02526906/full

%0 Journal Article

%A NCT

%D 2020

%T Mindfulness-based Cognitive Therapy for Patients With Inflammatory Bowel Disease

%8 2020-01-01

%K Inflammatory Bowel Diseases; Intestinal Diseases

%X Introduction: Inflammatory bowel diseases (IBD) are chronic inflammatory diseases of the intestinal tract, consisting of crohn's disease (CD) and ulcerative colitis (UC). In The Netherlands, there are about 90,000 IBD patients, mainly young adults who are in a turbulent part of their life with starting a family and career. The prevalence of IBD seems to be rising. The peak incidence is between 15 and 30 years. The disease is characterized by periods of disease activity (flares) alternating with periods of (clinical) remission. Physical symptoms include abdominal pain, diarrhea, rectal bleeding and fatigue. Although the arsenal of drugs has increased in recent decades, there is no cure for the disease and patients are usually long‐term treated with different (immunosuppressive) medications. Nevertheless, at this moment, we are only partially able to keep our IBD patients in remission. Even in those in remission, IBD is associated with increased levels of psychological stress, with estimated prevalence rates of 21% for depressive symptoms and 35% for anxiety symptoms. In periods of disease activity, prevalence of elevated anxiety levels was even found to be as high as 75.6%. Besides psychological stress, IBD patients in remission report lower quality of life compared to the general population. This is due to the physical symptoms but also to fatigue, worries, anxiety, depression, impaired satisfaction with social role and sleep disturbances. More than 40 % of the IBD patients experience fatigue. This may contribute to the higher sick leave days and higher work impairment rates in quiescent IBD, causing high societal costs. In addition, poor sleep seems common in individuals with IBD and has been linked to disease activity and shown as a predictor for subclinical inflammation and a risk factor for relapse and poorer outcomes. Mindfulness training, typically in the form of mindfulness‐based stress reduction (MBSR) or mindfulness‐based cognitive therapy (MBCT), is a psychosocial group‐based intervention that has been shown to reduce psychological distress and improving quality of life, both in patients with mental health problems and those with chronic diseases such as diabetes, cancer, cardiovascular disease, rheumatoid arthritis and fibromyalgia. Mindfulness training focuses on the progressive acquisition of mindful awareness to better cope with negative repetitive thoughts and feelings, and for that reason may be a good option to reduce psychological stress and fatigue, and improve quality of life in patients with chronic conditions including IBD. Although the available literature on the effectiveness of mindfulness‐based interventions for IBD is still relatively scarce, a recent meta‐analysis showed significant benefits in terms of stress, depression, and quality of life. However, the variety in types of interventions was rather high, and individual studies typically suffered from methodological limitations, including small sample size, poor blinding of the outcome assessors, incomplete data reporting and a relative lack of fidelity measures. Therefore, it seems warranted to conduct a high‐quality, multicenter randomized controlled trial investigating the effectiveness of MBCT to improve stress, sleep quality, and quality of life. Aims: Considering the limited availability of psychosocial interventions for IBD, this study aims to investigate MBCT as an adjunctive treatment to TAU to reduce psychological stress in patients with IBD who report elevated stress levels. In addition, we aim to improve sleep quality/regularity and fatigue, IBD‐related quality of life, perceived control over IBD, clinical indicators (fecal calprotectin, c‐reactive protein levels, Harvey Brashaw Index for CD, Simple Clinical Colitis Activity Index for UC), IBD‐related flare. We also aim to improve repetitive negative thinking, mindfulness skills, self‐compassion skills, and positive mental health. We will investigate whether improving sleep quality may be one of the pathways through which mindfulness may help reduce psychological distress and improve quality of life in IBD (working mechanism). Method: A two‐armed randomized, multicenter, parallel group pragmatic trial comparing Mindfulness‐Based Cognitive Therapy (MBCT) vs. Treatment As Usual (TAU) for reducing psychological distress in distressed patients with Inflammatory Bowel Disease (IBD). Assessments will be conducted at baseline, post treatment (3 months) and follow‐ups at 6, 9, and 12 months. Objective sleep measures will be taken at baseline, post treatment and 12 months. The control group will receive treatment as usual (TAU) according to Dutch and European IBD treatment guidelines. The intervention group will receive Mindfulness‐Based Cognitive Therapy (MBCT) in addition to TAU. The MBCT protocol that will be used is based on the protocol published by Segal, Williams and Teasdale. MBCT consists of eight weekly 2.5h group sessions, a six‐hour silent day and daily home practice assignments guided by audio files. Mindfulness is characterized by deliberate, non‐judging and sustained moment‐to‐moment awareness. Health related benefits include enhanced emotional processing and coping regarding the effects of chronic illness and stress, improved self‐efficacy and control, and a more accepting attitude towards difficult emotions, thought and bodily sensations. Psycho‐education and interactive dialogue typically focus on stress management, balancing activities, lifestyle factors, and strategies to stay well in the future (relapse prevention). Each group will be comprising 8‐12 participants. MBCT courses will be taught on site by qualified mindfulness teachers. Teacher competency will be assessed with the Mindfulness‐Based Interventions ‐ Teaching Assessment Criteria, using a random selection of videotaped sessions, assessed by expert teachers who have been trained to use these assessment criteria.

%Z https://clinicaltrials.gov/show/NCT04646785

Trial registry record

%U https://cochrane.66557.net/central/doi/10.1002/central/CN-02205970/full

%0 Journal Article

%A Herfarth, H.

%A Barnes, E. L.

%A Jackson, S.

%A Valentine, J. F.

%A Hanson, J.

%A Higgins, PDR

%A Isaacs, K. L.

%A Osterman, M. T.

%A Sands, B. E.

%A Lewis, J. D.

%D 2018

%T Methotrexate is not superior to placebo in maintaining remission in patients with ulcerative colitis: results from the MERIT-UC study

%V 12

%P S300‐S301

%8 2018-01-01

%K *recurrence risk; *remission; *ulcerative colitis; Adult; Conference abstract; Controlled study; Double blind procedure; Drug combination; Drug therapy; Endoscopy; Female; Gene expression; Human; Major clinical study; Male; Multicenter study; Nausea; Pharmacokinetics; Randomization; Randomized controlled trial; Treatment failure

%X Background: Parenteral methotrexate (MTX) is effective in inducing and maintaining remission in patients with Crohn's disease. In the METEOR trial, MTX induced steroid‐free clinical remission but not endoscopic healing in a significantly higher percentage of patients compared with placebo (P). We conducted a randomised, multicentre placebo‐controlled trial to determine the efficacy and safety of MTX in maintaining steroid free remission in patients with moderately‐severely active UC, who had responded and were able to discontinue steroids after open label induction with steroids and MTX. Methods: The 48‐week trial comprised a 16 week open label induction period followed by a 32 week double‐blind placebocontrolled maintenance period (MP). Patients with active UC (Mayo score 6‐12 with endoscopy subscore ≥2) despite previous conventional or anti‐TNF therapy were included and treated with open label MTX 25 mg/week sc and a 12 week steroid taper. At week 16, responders, defined as a decrease of the clinical Mayo score to ≤5 and steroid free since week 12, were randomly assigned to either continue MTX 25 mg/week or P until week 48. All patients received 2.4 g mesalamine daily. We compared the efficacy of treatment by analysing the proportion of patients who remained relapse‐free during MP defined by a clinical Mayo score ≤2 at week 32 without increase ≥3 points during MP and no use of steroids or other medications to control disease activity during MP. We evaluated faecal calprotectin levels at screening, week 16 and week 48. Results: Fifty‐one percent (91/179) of patients responded and 30% (53/179) achieved remission at week 16. Of 91 patients with steroid free response, 7 patients declined randomisation and 84 patients were randomised in the P controlled MP. In the P and MTX arm, 63% (25/40) and 66% (29/44) of patients experienced a relapse (p = 0.75) (Figure 1). Of the patients without relapse at week 48, 75% (12/16) and 85% (12/15) of patients on P and MTX, respectively, were in steroid‐free clinical remission. There were no significant differences comparing relapse rates based on different calprotectin levels at week 16 or failed previous therapies before entry in the trial. Nausea was numerically more common with MTX. No new safety signals were detected. (Figure presented) Conclusions: Although when combined with a standardised steroid taper, parenteral MTX 25 mg/week induced a steroid free response and remission rate in a substantial proportion of pat. with active UC, it was not superior to P in preventing relapse of disease. Thus, MTX does not represent a therapeutic option for long‐term maintenance of remission in pat. with UC.

%Z Journal of Crohn's & colitis

Journal article; Conference proceeding

%U https://cochrane.66557.net/central/doi/10.1002/central/CN-01466842/full

%0 Journal Article

%A Herfarth, H. H.

%A Barnes, E. L.

%A Jackson, S.

%A Valentine, J. F.

%A Hanson, J.

%A Higgins, P. D.

%A Isaacs, K. L.

%A Osterman, M. T.

%A Sands, B. E.

%A Lewis, J. D.

%D 2018

%T METHOTREXATE IS NOT SUPERIOR TO PLACEBO IN MAINTAINING REMISSION IN PATIENTS WITH ULCERATIVE COLITIS -RESULTS FROM THE MERIT-UC STUDY

%V 154

%N 6

%P S‐389

%8 2018-01-01

%R 10.1016/S0016-5085(18)31597-X

%K *recurrence risk; *remission; *ulcerative colitis; Adult; Conference abstract; Controlled study; Crohn disease; Double blind procedure; Drug safety; Drug therapy; Endoscopy; Feces; Female; Gene expression; Human; Major clinical study; Male; Multicenter study; Nausea; Pharmacokinetics; Protein expression; Randomization; Randomized controlled trial; Treatment failure

%X Background: Parenteral methotrexate (MTX) is effective in inducing and maintaining remission in patients (pat.) with Crohn's disease. In the METEOR trial, MTX induced steroid free clinical remission but not endoscopic healing in a significantly higher percentage of pat. compared to placebo. We conducted a randomized, multicenter placebo‐controlled trial to determine the efficacy and safety of MTX in maintaining steroid free remission in pat. with moderately‐severely active UC, who had responded and were able to discontinue steroids after open label induction with steroids and MTX. Methods: The 48‐week trial comprised a 16‐week open label induction period followed by a 32‐week double‐blind placebo‐controlled maintenance period (MP). Pat. with active UC (Mayo score 6‐12 with endoscopy subscore ≥2) despite previous conventional therapy or tumor necrosis factor antagonist were included and treated with open label MTX 25 mg/week subcutaneously and a 12‐week steroid taper. At week 16, responders, defined as a decrease of the clinical Mayo score to ≤5 and steroid free since week 12, were randomly assigned to either continue MTX 25 mg/week or placebo until week 48. All pat. received 2.4 g mesalamine daily. We compared the efficacy of treatment by analyzing the proportion of pat. who remained relapse free during the 32‐week MP defined by a clinical Mayo score ≤ 2 at week 32 without increase ≥ 3 points during MP and no use of steroids or other medications to control disease activity during MP. We evaluated fecal calprotectin levels at screening, week 16 and week 48. Results: Fifty‐one percent (91/179) of patients responded and 30% (53/179) achieved remission at week 16. Of 91 pat. with steroid free response, 7 pat. declined randomization and 84 pat. were randomized in the placebo controlled MP. In the placebo and MTX arm, 63% (25/40) and 66% (29/44) of pat. experienced a relapse (p=0.75) (fig.1). Of the pat. without relapse at week 48, 75% (12/16) and 85% (12/15) of pat. on placebo and MTX, respectively, were in steroid free clinical remission. There were no significant differences comparing relapse rates based on different calprotectin levels at week 16 or failed previous therapies before entry in the trial. Nausea was numerically more common with MTX. No new safety signals were detected. Conclusion: Although when combined with a standardized steroid taper, parenteral MTX 25 mg/week induced a steroid free response and remission rate in a substantial proportion of pat. with active UC, it was not superior to placebo in preventing relapse of disease. Thus, MTX does not represent a therapeutic option for long term maintenance of remission in pat. with UC. [Figure Presented]

%Z Gastroenterology

Journal article; Conference proceeding

%U https://cochrane.66557.net/central/doi/10.1002/central/CN-01964883/full

%0 Journal Article

%A Houben, M. H.

%A van Wijk, H. J.

%A Driessen, W. M.

%A van Spreeuwel, J. P.

%D 1994

%T Methotrexate as possible treatment in refractory chronic inflammatory intestinal disease

%V 138

%N 51

%P 2552‐2556

%8 1994-01-01

%K Adolescent; Adult; Colitis [drug therapy]; Colitis, Ulcerative [*drug therapy]; Crohn Disease [*drug therapy]; Drug Administration Schedule; Female; Humans; Ileitis [drug therapy]; Male; Methotrexate [administration & dosage, *therapeutic use]; Middle Aged; Treatment Outcome

%X OBJECTIVE: To determine the value of low dose methotrexate therapy in chronic inflammatory bowel disease. SETTING: Catharina Hospital and Diaconessenhuis, Eindhoven, and St. Joseph Hospital, Veldhoven. DESIGN: Descriptive. METHOD: From 1988 until 1993 we treated 15 patients 16 times (one patient was treated twice) with methotrexate 25 mg i.m. once a week during 12 weeks, followed by a tapering oral dose. Our population consisted of 4 men and 11 women with a mean age of 31 year. The diagnosis was Crohn ileitis (1 patient), Crohn colitis (8), ileocolitis (4) and ulcerative colitis (2). The indication for methotrexate was resistance to therapy (9) and steroid dependency (7). Retrospectively the disease activity was determined after 1, 2 and 3 months of therapy. RESULTS: The mean defaecation frequency went down from 7 to 2 times daily after 12 weeks, the ESR from 47 to 17 mm/1st hour, the thrombocytes from 436 x 10(9)/l to 325 x 10(9)/l and the prednisone dose could be lowered from 22 mg to 15 mg after 3 months. In 13/16 treatment cases there was a subjective positive response to methotrexate. Initial response was seen within 4 weeks. No serious side effects were seen. After 10 of the 13 response episodes the disease recurred. CONCLUSION: Methotrexate 25 mg once a week i.m. was associated with a subjective and objective improvement in 12/15 patients, but the risk of recurrence after tapering appears to be large.

%Z Nederlands tijdschrift voor geneeskunde

Journal article

%U https://cochrane.66557.net/central/doi/10.1002/central/CN-00109476/full

%0 Journal Article

%A Pascua, M.

%A Su, C.

%A Lewis, J. D.

%A Brensinger, C.

%A Lichtenstein, G. R.

%D 2008

%T Meta-analysis: factors predicting post-operative recurrence with placebo therapy in patients with Crohn's disease

%V 28

%N 5

%P 545‐556

%8 2008-01-01

%R 10.1111/j.1365-2036.2008.03774.x

%K *Crohn disease /drug therapy /surgery; Adult; Aged; Aged, 80 and over; Article; Clinical trial; Colon disease; Crohn Disease [*surgery]; Data Interpretation, Statistical; Endoscopic surgery; Endoscopy [methods]; Female; Follow up; Human; Humans; Intestine fistula; Maintenance therapy; Male; Meta analysis; Middle Aged; Phenotype; Placebos; Postoperative period; Priority journal; Randomized Controlled Trials as Topic; Randomized controlled trial; Recurrence; Recurrence risk; Remission; Remission Induction [methods]; Severity of Illness Index; Small intestine disease; Steroid therapy; Systematic review; Treatment Outcome; Treatment outcome

%X BACKGROUND: The use of placebo in randomized clinical trials (PC‐RCTs) is often required to evaluate drug efficacy in maintenance of Crohn's disease (CD). AIM: To determine pooled estimates of placebo rates of maintaining clinical remission and endoscopic recurrence following surgery for CD and identify factors that influenced placebo outcomes. METHODS: We performed a systematic review and meta‐analysis of PC‐RCTs evaluating post‐operative maintenance therapies for CD identified from MEDLINE from 1966 to 2005. RESULTS: Twelve studies met our inclusion criteria. The pooled placebo rate of maintaining clinical remission was 56% (95% CI 47‐64%; range 34‐89%) during a median follow‐up of 52 weeks (range 12‐156 weeks), but significant heterogeneity existed among the studies (P < 0.001). Prior steroid therapy was the only factor found to be associated with maintaining remission (P = 0.04). The pooled placebo endoscopic recurrence rate was 58% (95% CI 51‐65%; range 36‐80%) during a median follow‐up of 52 weeks (range 12‐156 weeks), with significant heterogeneity noted (P = 0.0003). Prior surgery, concomitant small bowel and colonic disease, fistulizing phenotype, or prior immunomodulator therapy influenced endoscopic recurrence (P < 0.05). CONCLUSION: Placebo rates in PC‐RCTs evaluating post‐operative clinical and endoscopic recurrence demonstrate significant variability, which is influenced by specific study characteristics.

%Z Alimentary pharmacology & therapeutics

Journal article

%U https://cochrane.66557.net/central/doi/10.1002/central/CN-01715991/full

%0 Journal Article

%A Doherty, G. A.

%A Bennett, G.

%A Patil, S.

%A Cheifetz, A.

%A Moss, A. C.

%D 2009

%T Meta-analysis of probiotics in the prevention of post-operative recurrence of Crohn's disease

%V 136

%N 5

%P A772‐A773

%8 2009-01-01

%R 10.1016/S0016-5085(09)63569-1

%K *Crohn disease; *gastrointestinal disease; *meta analysis; *prevention; Bacterium; Controlled study; Data base; Inflammation; Lactobacillus; Lactobacillus johnsonii; Patient; Randomized controlled trial; Risk; Risk factor; Software; Surgery; Systematic review; Therapy

%X Background. Post‐operative recurrence of Crohn's Disease (CD) is common following surgical resection of a diseased segment. Recolonisation with luminal bacteria appears important in the recurrence of inflammation. There has been sustained interest in the idea that probiotic therapy might prevent pathogenic recolonisation and reduce the risk of recurrence. Objectives. To undertake a systematic review of the published literature relating to the use of probiotic agents in the prevention of post‐operative recurrence of CD. Strategy. A literature search was performed (using electronic databases and hand‐searching of conference abstracts) to identify published and unpublished randomized controlled trials comparing any probiotic agent to placebo (or other agents) in prevention of endoscopic or clinical recurrence of CD after resection. Abstracts which met search criteria were reviewed by two reviewers and those meeting inclusion criteria were selected for full data abstraction. Included studies were graded as having low, moderate or high risk of bias using standard criteria (Jadad quality score). A fixed‐effect meta‐analysis was performed using RevMan Software (version 4.2). Results. Five randomised controlled studies were identified which met inclusion criteria. Two studies examined the effect of Lactobacillus johnsonii (LA1), one study the effect of Lactobacillus GG (LGG) and the remainder evaluated the effect of probiotic cocktails (VSL#3, one study; Synbiotic 2000 prebiotic/probiotic cocktail, one study). In aggregate, a total of 245 patients were randomized to receive probiotic therapy or placebo. The use of probiotics was not associated with a significant reduction in the risk of clinical recurrence (relative risk 1.37, 95%CI 0.65‐2.89, p=0.41), see Fig 1 . Relative risk of any endoscopic recurrence (RR 0.98, 0.74‐1.29, p=0.87) or of severe endoscopic recurrence (Rutgeert's score>2, RR 0.98, 0.6‐1.62, p=0.94) were not significantly different with probiotic therapy. Conclusion. Evidence to date from randomized trails does not demonstrate any benefit to probiotic therapy for prevention of post‐operative recurrence of CD. {Figure presented}.

%Z Gastroenterology

Journal article; Conference proceeding

%U https://cochrane.66557.net/central/doi/10.1002/central/CN-01709713/full

%0 Journal Article

%A Doherty, G. A.

%A Bennett, G.

%A Patil, S.

%A Cheifetz, A.

%A Moss, A. C.

%D 2009

%T Meta-Analysis of mesalamine in the prevention of post-operative recurrence of Crohn's disease

%V 136

%N 5

%P A522

%8 2009-01-01

%R 10.1016/S0016-5085(09)62405-7

%K *Crohn disease; *gastrointestinal disease; *meta analysis; *prevention; Arm; Data base; Patient; Randomized controlled trial; Recurrent disease; Risk; Risk factor; Software; Surgery; Systematic review; Toxicity

%X Background. Crohn's Disease (CD) recurs frequently following surgical resection. The ideal strategy to prevent disease recurrence is unknown. Mesalamine has demonstrated modest efficacy in a number of RCTs. Objectives. To undertake a systematic review of the published literature relating to the use of mesalamine in the prevention of post‐operative recurrence of CD. Strategy. A literature search was performed (using electronic databases and hand‐searching of conference abstracts) to identify published and unpublished randomized controlled trials comparing mesalamine to placebo or azathioprine/6MP in prevention of endoscopic or clinical recurrence of CD after resection. Included studies were graded as having low, moderate or high risk of bias using standard criteria. Raw data was extracted and analyzed in an intention‐to‐treat manner. A fixed‐effect meta‐analysis was performed using RevMan Software. Results. Nine RCTs were identified which met inclusion criteria; six studied the effect of mesalamine versus placebo and three studies evaluated the effect of mesalamine versus azathioprine/6‐MP (one also had a placebo arm). A total of 542 patients were randomized to mesalamine, 135 to azathioprine/6MP, and 451 to placebo. There was no significant statistical heterogeneity in the studies included in meta‐analysis. The analysis demonstrated a reduced relative risk of clinical recurrence with mesalamine compared to placebo (RR 0.72, 95%CI 0.58‐0.89, p=0.002)(Fig. 1). Relative risk of severe endoscopic recurrence (Rutgeert's score>2) also favoured mesalamine relative to placebo (RR 0.64; 0.43‐0.94, p=0.02), though the risk of any endoscopic recurrence was not significantly lowered (RR 0.89; 0.75‐1.05, p=0.17). However, mesalamine was inferior to azathioprine/6MP in preventing clinical recurrence (RR 1.4; 1.01‐1.95, p=0.05) or any endoscopic recurrence (RR 1.69: 1.13‐2.52, p=0.01). Conclusion. Meta‐analysis of existing trials suggests that mesalamine is more effective than placebo, but less effective than azathioprine/6MP, in the prevention of post‐operative recurrence of CD. The relative toxicity of both strategies requires careful evaluation in formulating the optimal prevention strategy. {Table presented}.

%Z Gastroenterology

Journal article; Conference proceeding

%U https://cochrane.66557.net/central/doi/10.1002/central/CN-01724805/full

%0 Journal Article

%A van der Does De Willebois, EML

%D 2022

%T Mesenteric SParIng versus extensive mesentereCtomY in primary ileocolic resection for ileocaecal Crohn's disease (SPICY): study protocol for randomized controlled trial

%V 6

%N 1

%8 2022-01-01

%R 10.1093/bjsopen/zrab136

%K *Crohn disease; *mesentery; Adult; Aged; Article; Clinical evaluation; Controlled study; Crohn Disease [surgery]; Female; Human; Humans; Ileum [surgery]; Intestine resection; Major clinical study; Male; Mesentery [pathology, surgery]; Morbidity; Multicenter Studies as Topic; Multicenter study; Neoplasm Recurrence, Local [pathology]; Outcome assessment; Practice guideline; Quality of Life; Quality of life; Randomized Controlled Trials as Topic; Randomized controlled trial; Recurrence risk; Recurrent disease; Reoperation; Trunk

%X BACKGROUND: There is emerging evidence to suggest that Crohn's disease (CD) may be a disease of the mesentery, rather than of the bowel alone. A more extensive mesenteric resection, removing an increased volume of mesentery and lymph nodes to prevent recurrence of CD, may improve clinical outcomes. This study aims to analyse whether more extensive 'oncological' mesenteric resection reduces the recurrence rate of CD. METHODS: This is an international multicentre randomized controlled study, allocating patients to either group 1‐mesenteric sparing ileocolic resection (ICR), the current standard procedure for CD, or group 2‐extensive mesenteric ICR, up to the level of the ileocolic trunk. To detect a clinically relevant difference of 25 per cent in endoscopic recurrence at 6 months, a total of 138 patients is required (including 10 per cent dropout). Patients aged over 16 with CD undergoing primary ICR are eligible. Primary outcome is 6‐month postoperative endoscopic recurrence rate (modified Rutgeerts score of greater than or equal to i2b). Secondary outcomes are postoperative morbidity, clinical recurrence, quality of life, and the need for (re)starting immunosuppressive medication. For long‐term results, patients will be followed up for up to 5 years to determine the reoperation rate for recurrence of disease at the anastomotic site. CONCLUSION: Analysing these two treatment strategies in a head‐to‐head comparison will allow an objective evaluation of the clinical relevance of extensive mesenteric resection in CD. If a clinical benefit can be demonstrated, this could result in changes to guidelines which currently recommend close bowel resection. REGISTRATION NUMBER: NCT00287612 (http://www.clinicaltrials.gov).

%Z BJS open

Journal article

%U https://cochrane.66557.net/central/doi/10.1002/central/CN-02374417/full

%0 Journal Article

%A Li, Y.

%A Mohan, H.

%A Lan, N.

%A Wu, X.

%A Zhou, W.

%A Gong, J.

%A Shen, B.

%A Stocchi, L.

%A Coffey, J. C.

%A Zhu, W.

%D 2020

%T Mesenteric excision surgery or conservative limited resection in Crohn's disease: study protocol for an international, multicenter, randomized controlled trial

%V 21

%N 1

%P 210

%8 2020-01-01

%R 10.1186/s13063-020-4105-x

%K *Crohn disease /surgery; *extensive mesenteric excision; *gastrointestinal surgery; *limited mesenteric excision; Adult; Aged; Article; Clinical trial protocol; Cohort analysis; Colectomy [*adverse effects, *methods]; Colon [pathology, surgery]; Controlled study; Crohn Disease [*surgery]; Demography; Disease Progression; Dissection [methods]; Endoscopy; Human; Humans; Ileum [pathology, surgery]; Internationality; Intraoperative period; Lymph Node Excision; Maintenance therapy; Major clinical study; Medical practice; Mesentery [*pathology, *surgery]; Morbidity; Multicenter Studies as Topic; Multicenter study; Outcome and Process Assessment, Health Care; Outcome assessment; Postoperative period; Randomized Controlled Trials as Topic; Randomized controlled trial; Recurrence; Recurrent disease; Risk Factors; Risk factor; Risk reduction; Sample size; Study design

%X Background: The structures of the mesentery including adipose tissue, nerves, and lymphatics play an important role in the pathogenesis and disease progression of Crohn's disease (CD). Conventional surgical resection for CD usually does not involve resecting the mesentery en bloc with the specimen. This contrasts with complete mesocolic excision (CME) in colorectal cancer, which involves radical resection of the mesentery. Preliminary evidence from smaller studies suggests that applying the principle of mesocolic excision to CD surgery may reduce the risk of postoperative recurrence. This randomized controlled trial is designed to test whether applying the principles of mesocolic excision to CD results in reduced postoperative recurrence. It also aims to evaluate intra‐ and postoperative morbidity between the two approaches. Methods: This international, multicenter, randomized controlled trial will randomize patients (n = 116) scheduled to undergo primary ileocolic resection to either receive extensive mesenteric excision (EME) or conventional ileocolic resection with limited mesenteric excision (LME). Five sites will recruit patients in three countries. In the EME group, the mesentery is resected following CME, while avoiding the root region, i.e., 1 cm from the root of the ileocolic artery and vein. In the LME group, the mesentery is retained, i.e., "close shave" or < 3 cm from the border of bowel. The primary end point will be surgical recurrence after surgery. The secondary end points will be the postoperative endoscopic and clinical recurrence, and intra‐ and postoperative morbidity. Demographics, risk factors, laboratory investigations, endoscopy, postoperative prophylaxis and imaging examination will be assessed. Analysis of the primary outcome will be on an intention‐to‐treat basis. Discussion: If mesocolic excision in CD reduces postoperative disease recurrence and does not increase morbidity, this trial has the potential to change practice and reduce recurrence of CD after surgical resection. Trial registration: Clinical Trials.gov, ID: NCT03769922. Registered on February 27, 2019.

%Z Trials

Journal article

%U https://cochrane.66557.net/central/doi/10.1002/central/CN-02099394/full

%0 Journal Article

%A Modigliani, R.

%A Colombel, J. F.

%A Dupas, J. L.

%A Dapoigny, M.

%A Costil, V.

%A Veyrac, M.

%A Duclos, B.

%A Soulé, J. C.

%A Gendre, J. P.

%A Galmiche, J. P.

%A Et, Al.

%D 1996

%T Mesalamine in Crohn's disease with steroid-induced remission: effect on steroid withdrawal and remission maintenance, Groupe d'Etudes Thérapeutiques des Affections Inflammatoires Digestives

%V 110

%N 3

%P 688‐693

%8 1996-01-01

%R 10.1053/gast.1996.v110.pm8608877

%K Adolescent; Adult; Aminosalicylic Acids [*therapeutic use]; Anti‐Inflammatory Agents [administration & dosage, *therapeutic use]; Anti‐Inflammatory Agents, Non‐Steroidal [therapeutic use]; Belgium; Chi‐Square Distribution; Crohn Disease [*drug therapy]; Double‐Blind Method; Drug Therapy, Combination; Female; Follow‐Up Studies; France; Humans; Logistic Models; Male; Mesalamine; Prednisolone [administration & dosage, *therapeutic use]; Proportional Hazards Models; Recurrence; Remission Induction

%X BACKGROUND & AIMS: Steroid dependence and early relapse are frequent after a prednisolone‐induces remission in Crohn's disease. The aim of this trial was to test whether mesalamine started at the onset of steroid tapering increases the rate of weaning from prednisolone and reduces the relapse rate after prednisolone cessation. METHODS: One hundred fifty patients with active Crohn's disease were administered oral prednisolone (1 mg.kg(‐1). day(‐1)) x 3‐7 weeks; 129 patients went into clinical remission and were randomized to Pentasa (4 g . day(‐1)) or placebo, administered until weaning and for 1 year thereafter. RESULTS: Groups were similar for clinical and biological items collected initially. Weaning failure rate was 30% and 12% in the placebo and mesalamine arms, respectively. At the end of the trial, 9 of 36 patients administered placebo and 14 of 48 administered mesalamine were in remission. Both groups had similar time to relapse curves in the postweaning year; after adjusting for risk factors (high Crohn's Disease Activity Index, white blood cell count of >9 x 10(9) /l‐1 at weaning, and use of a medical treatment in the month before inclusion), Pentasa was found to be superior to placebo. CONCLUSIONS: After a prednisolone‐induces remission in Crohn's disease, mesalamine facilitates steroid withdrawal and, during the postweaning year, may reduce the relapse rate in certain patient subgroups.

%Z Gastroenterology

Journal article

%U https://cochrane.66557.net/central/doi/10.1002/central/CN-00123525/full

%0 Journal Article

%A Mowat, C.

%A Arnott, I.

%A Cahill, A.

%A Smith, M.

%A Ahmad, T.

%A Subramanian, S.

%A Travis, S.

%A Morris, J.

%A Hamlin, J.

%A Dhar, A.

%A Et, Al.

%D 2016

%T Mercaptopurine versus placebo to prevent recurrence of Crohn's disease after surgical resection (TOPPIC): a multicentre, double-blind, randomised controlled trial

%V 1

%N 4

%P 273‐282

%8 2016-01-01

%R 10.1016/S2468-1253(16)30078-4

%K Administration, Oral; Adolescent; Adult; Aged; Crohn Disease [diagnosis, *prevention & control, *surgery]; Double‐Blind Method; Drug Administration Schedule; Female; Follow‐Up Studies; Humans; Immunosuppressive Agents [*therapeutic use]; Male; Mercaptopurine [*therapeutic use]; Middle Aged; Recurrence; Secondary Prevention [*methods]; Smoking [adverse effects]; Treatment Outcome; Young Adult

%X BACKGROUND: Up to 60% of patients with Crohn's disease need intestinal resection within the first 10 years of diagnosis, and postoperative recurrence is common. We investigated whether mercaptopurine can prevent or delay postoperative clinical recurrence of Crohn's disease. METHODS: We did a randomised, placebo‐controlled, double‐blind trial at 29 UK secondary and tertiary hospitals of patients (aged >16 years in Scotland or >18 years in England and Wales) who had a confirmed diagnosis of Crohn's disease and had undergone intestinal resection. Patients were randomly assigned (1:1) by a computer‐generated web‐based randomisation system to oral daily mercaptopurine at a dose of 1 mg/kg bodyweight rounded to the nearest 25 mg or placebo; patients with low thiopurine methyltransferase activity received half the normal dose. Patients and their carers and physicians were masked to the treatment allocation. Patients were followed up for 3 years. The primary endpoint was clinical recurrence of Crohn's disease (Crohn's Disease Activity Index >150 plus 100‐point increase in score) and the need for anti‐inflammatory rescue treatment or primary surgical intervention. Primary and safety analyses were by intention to treat. Subgroup analyses by smoking status, previous thiopurines, previous infliximab or methotrexate, previous surgery, duration of disease, or age at diagnosis were also done. This trial is registered with the International Standard Randomised Controlled Trial Register (ISRCTN89489788) and the European Clinical Trials Database (EudraCT number 2006‐005800‐15). FINDINGS: Between June 6, 2008, and April 23, 2012, 240 patients with Crohn's disease were randomly assigned: 128 to mercaptopurine and 112 to placebo. All patients received at least one dose of study drug, and no randomly assigned patients were excluded from the analysis. 16 (13%) of patients in the mercaptopurine group versus 26 (23%) patients in the placebo group had a clinical recurrence of Crohn's disease and needed anti‐inflammatory rescue treatment or primary surgical intervention (adjusted hazard ratio [HR] 0·54, 95% CI 0·27‐1·06; p=0·07; unadjusted HR 0·53, 95% CI 0·28‐0·99; p=0·046). In a subgroup analysis, three (10%) of 29 smokers in the mercaptopurine group and 12 (46%) of 26 in the placebo group had a clinical recurrence that needed treatment (HR 0·13, 95% CI 0·04‐0·46), compared with 13 (13%) of 99 non‐smokers in the mercaptopurine group and 14 (16%) of 86 in the placebo group (0·90, 0·42‐1·94; pinteraction=0·018). The effect of mercaptopurine did not significantly differ from placebo for any of the other planned subgroup analyses (previous thiopurines, previous infliximab or methotrexate, previous surgery, duration of disease, or age at diagnosis). The incidence and types of adverse events were similar in the mercaptopurine and placebo groups. One patient on placebo died of ischaemic heart disease. Adverse events caused discontinuation of treatment in 39 (30%) of 128 patients in the mercaptopurine group versus 41 (37%) of 112 in the placebo group. INTERPRETATION: Mercaptopurine is effective in preventing postoperative clinical recurrence of Crohn's disease, but only in patients who are smokers. Thus, in smokers, thiopurine treatment seems to be justified in the postoperative period, although smoking cessation should be strongly encouraged given that smoking increases the risk of recurrence. FUNDING: Medical Research Council.

%Z The lancet. Gastroenterology & hepatology

Journal article

%U https://cochrane.66557.net/central/doi/10.1002/central/CN-01600522/full

%0 Journal Article

%A Mowat, C.

%A Arnott, I.

%A Cahill, A.

%A Smith, M.

%A Ahmad, T.

%A Subramanian, S.

%A Travis, S.

%A Morris, J.

%A Hamlin, J.

%A Dhar, A.

%A Et, Al.

%D 2016

%T Mercaptopurine versus placebo to prevent recurrence of Crohn's disease after surgical resection (TOPPIC): a multicentre, double-blind, randomised controlled trial

%V 1

%N 4

%P 273‐282

%8 2016-01-01

%R 10.1016/S2468-1253%2816%2930078-4

%K *Crohn disease; *Crohn disease/dt [Drug Therapy]; *Crohn disease/pc [Prevention]; *disease duration; *mercaptopurine; *mercaptopurine/cm [Drug Comparison]; *mercaptopurine/ct [Clinical Trial]; *mercaptopurine/dt [Drug Therapy]; *mercaptopurine/po [Oral Drug Administration]; *placebo; *postoperative complication/dt [Drug Therapy]; *postoperative complication/pc [Prevention]; *relapse; Adolescent; Adult; Adverse drug reaction; Aged; Article; Body weight; Calgranulin; Clinical trial; Colonoscopy; Controlled clinical trial; Controlled study; Crohn Disease Activity Index; Crohn disease/dt [Drug Therapy]; Data base; Diagnosis; Double blind procedure; Drug therapy; Endogenous compound; England; Female; Follow up; Funding; Genetic analysis; Hazard ratio; Human; Ileocecal valve; Incidence; Infliximab; Intestine resection; Ischemic heart disease; Major clinical study; Male; Medical research; Methotrexate; Multicenter study; Patient history of surgery; Physician; Placebo; Postoperative complication/dt [Drug Therapy]; Postoperative period; Prevention; Priority journal; Quality of life; Randomization; Randomized controlled trial; Safety; Scotland; Side effect; Smoking; Smoking cessation; Surgery; Tertiary care center; Thiopurine methyltransferase; Treatment outcome; Visually impaired person; Wales

%X Background Up to 60% of patients with Crohn's disease need intestinal resection within the first 10 years of diagnosis, and postoperative recurrence is common. We investigated whether mercaptopurine can prevent or delay postoperative clinical recurrence of Crohn's disease. Methods We did a randomised, placebo‐controlled, double‐blind trial at 29 UK secondary and tertiary hospitals of patients (aged >16 years in Scotland or >18 years in England and Wales) who had a confirmed diagnosis of Crohn's disease and had undergone intestinal resection. Patients were randomly assigned (1:1) by a computer‐generated web‐based randomisation system to oral daily mercaptopurine at a dose of 1 mg/kg bodyweight rounded to the nearest 25 mg or placebo; patients with low thiopurine methyltransferase activity received half the normal dose. Patients and their carers and physicians were masked to the treatment allocation. Patients were followed up for 3 years. The primary endpoint was clinical recurrence of Crohn's disease (Crohn's Disease Activity Index >150 plus 100‐point increase in score) and the need for anti‐inflammatory rescue treatment or primary surgical intervention. Primary and safety analyses were by intention to treat. Subgroup analyses by smoking status, previous thiopurines, previous infliximab or methotrexate, previous surgery, duration of disease, or age at diagnosis were also done. This trial is registered with the International Standard Randomised Controlled Trial Register (ISRCTN89489788) and the European Clinical Trials Database (EudraCT number 2006‐005800‐15). Findings Between June 6, 2008, and April 23, 2012, 240 patients with Crohn's disease were randomly assigned: 128 to mercaptopurine and 112 to placebo. All patients received at least one dose of study drug, and no randomly assigned patients were excluded from the analysis. 16 (13%) of patients in the mercaptopurine group versus 26 (23%) patients in the placebo group had a clinical recurrence of Crohn's disease and needed anti‐inflammatory rescue treatment or primary surgical intervention (adjusted hazard ratio [HR] 0.54, 95% CI 0.27‐1.06; p=0.07; unadjusted HR 0.53, 95% CI 0.28‐0.99; p=0.046). In a subgroup analysis, three (10%) of 29 smokers in the mercaptopurine group and 12 (46%) of 26 in the placebo group had a clinical recurrence that needed treatment (HR 0.13, 95% CI 0.04‐0.46), compared with 13 (13%) of 99 non‐smokers in the mercaptopurine group and 14 (16%) of 86 in the placebo group (0.90, 0.42‐1.94; pinteraction=0.018). The effect of mercaptopurine did not significantly differ from placebo for any of the other planned subgroup analyses (previous thiopurines, previous infliximab or methotrexate, previous surgery, duration of disease, or age at diagnosis). The incidence and types of adverse events were similar in the mercaptopurine and placebo groups. One patient on placebo died of ischaemic heart disease. Adverse events caused discontinuation of treatment in 39 (30%) of 128 patients in the mercaptopurine group versus 41 (37%) of 112 in the placebo group. Interpretation Mercaptopurine is effective in preventing postoperative clinical recurrence of Crohn's disease, but only in patients who are smokers. Thus, in smokers, thiopurine treatment seems to be justified in the postoperative period, although smoking cessation should be strongly encouraged given that smoking increases the risk of recurrence. Funding Medical Research Council. Copyright © 2016 The Author(s). Published by Elsevier Ltd. This is an Open Access article under the CC BY license

%Z The lancet gastroenterology and hepatology

Journal article

%U https://cochrane.66557.net/central/doi/10.1002/central/CN-01288144/full

%0 Journal Article

%A Brown, S.

%D 2021

%T MEErKAT MESENTERIC EXCISION AND KONO-S ANASTOMOSIS TRIAL

%V 23

%P 199

%8 2021-01-01

%R 10.1111/codi.15867

%K *anastomosis; *excision; *mesentery; Adult; Aged; B lymphocyte; Calculation; Cell population; Clinical trial; Complication; Conference abstract; Controlled study; Crohn disease; Dendritic cell; Female; Follow up; Human; Human cell; Ileocecal resection; Immunocompetent cell; Macrophage; Major clinical study; Male; Monocyte; Multicenter study; Natural killer cell; Open study; Outcome assessment; Radical resection; Randomized controlled trial; Recurrence risk; Recurrent disease; Sample size; Surgeon; Surgery; Surgical approach; T lymphocyte; Traction therapy; Young adult

%X Background: Despite advances in medical therapy 70%‐90% of patients with Crohn's Disease eventually need surgery. Unfortunately even with the use of adjuvant medical therapy, over half of patients develop recurrent disease after surgery and one third will need further surgery within 10 years. Many surgeons have deliberated on whether the methods of resection and rejoining the bowel influence outcome. Various anastomotic configurations have been trialled, with conflicting results. Others have considered the mesentery as the focus of disease proposing a more radical mesenteric resection reduces recurrence. The Kono‐S anastomosis is a novel anastomotic technique that has been gaining traction based on relatively poor quality evidence but an apparent spectacular reduction in recurrence. An interesting component of the technique is the proposal that the mesentery is essentially preserved. This contrasts with the claims of those who see the mesentery as the driver of recurrence and radical resection as essential. Objectives and research questions: One commonality of both approaches is the fact that both aim to isolate the anastomosis from diseased mesentery. Indeed, the techniques may be complementary and could be carried out together. The lack of good quality data for these surgical approaches combined with our lack of understanding of the role of the mesentery in promoting recurrence allows for an efficient trial design comparing various surgical approaches for Crohn's resection and a mechanistic analysis of locality of recurrence and key immune drivers of recurrence. Methods: Multicentre UK, superiority, 2 × 2 factorial, randomized, open ‐label trial with a minimum one‐year follow‐up. Participants aged 18‐75 years undergoing ileocaecal resection for primary/recurrent Crohn's disease where an anastomosis is carried out will be randomised (1:1:1:1) to one of four groups: 1. Kono‐S + radical mesenteric resection 2. Kono‐S + closed mesenteric resection 3. Standard anastomosis + radical mesenteric resection 4. Standard anastomosis + closed mesenteric resection An additional mechanistic element will determine in those that develop endoscopic recurrence, the locality of that recurrence and the key immune drivers of recurrence. The primary outcome will be time to endoscopic recurrence (after a minimum of 12 months and maximum of 3 years follow‐up) using Rutgeert's score [≥i2]. Secondary outcomes include symptomatic recurrence, severe recurrence, complications, locality of recurrence in relation to the mesentery. Mechanistic outcomes include analysis of key innate and adaptive immune cell populations (monocytes/macrophages, dendritic cells, B cells,T cells, NK cells and inflammatory cytokines). The sample size calculation assumes: 90% power; 5% (two‐sided) significance level; and estimated 1‐year endoscopic recurrence rates of 65% in the standard anastomosis/close mesenteric resection group; 35% in the standard anastomosis and radical mesenteric resection group (combined 50%); 35% in the Kono‐S and close mesenteric resection group and a 25% recurrence rate in the Kono‐S and radical mesenteric resection group (estimated assuming no interaction) (combined 30%). Based on a reduction in the 1‐year endoscopic recurrence rate from 50% to 30%, a total of 104 recurrences are required (using the Freedman method). To account for surgeon effects we assume an ICC of 0.01. Assuming 5% attrition we require 308 patients (77 per group).

%Z Colorectal disease

Journal article; Conference proceeding

%U https://cochrane.66557.net/central/doi/10.1002/central/CN-02533359/full

%0 Journal Article

%A Belaiche, J.

%A Louis, E.

%D 1999

%T Management of postoperative Crohn's disease recurrence

%V 29

%N 3

%P 253‐262

%8 1999-01-01

%R 10.1007/BF03019415

%K *Crohn disease /drug therapy /etiology /surgery; Article; Clinical trial; Colitis /drug therapy /etiology /surgery; Controlled study; Disease course; Double blind procedure; Gastrointestinal endoscopy; Human; Major clinical study; Meta analysis; Multicenter study; Pathophysiology; Postoperative period; Randomized controlled trial; Recurrent disease

%X The risk for recurrence in Crohn's disease after surgical resection is high. The etiology of recurrence, as well as the one of the disease itself, remains unknown. Endoscopic evidence of recurrence at the level of the anastomosis is a frequent and early sign and precedes clinical recurrence in all cases. Among the numerous factors suggested, only the initial site of the disease, the type of surgery and smoking habits seem to really define risk factors. Currently, an important role in recurrence is suggested for fecal stream. Drugs derived from 5‐ASA are moderately active in the prevention of recurrence. Natural history of postoperative recurrence defines a privileged model for the study of physiopathology in Crohn's disease.

%Z Acta endoscopica

Journal article

%U https://cochrane.66557.net/central/doi/10.1002/central/CN-01755892/full

%0 Journal Article

%A Regueiro, M.

%D 2009

%T Management and prevention of postoperative Crohn's disease

%V 15

%N 10

%P 1583‐1590

%8 2009-01-01

%R 10.1002/ibd.20909

%K *Crohn disease /drug therapy /complication /drug therapy /prevention /therapy; *postoperative complication /drug therapy /complication /drug therapy /prevention /therapy; Cigarette smoking; Clinical trial; Combination chemotherapy; Controlled clinical trial; Crohn Disease [*prevention & control, *surgery]; Early intervention; Gastroscopy; Human; Humans; Intestine surgery; Low drug dose; Maintenance therapy; Monotherapy; Patient monitoring; Postoperative Complications [*prevention & control]; Priority journal; Randomized controlled trial; Recurrence; Recurrence risk; Relapse; Remission; Reoperation; Review; Risk assessment; Risk reduction; Single drug dose; Smoking cessation; Surgical risk; Treatment duration; Treatment response

%X Postoperative Crohn's disease (CD) recurrence is a common occurrence after intestinal resection. Currently, the optimal management of patients who have undergone surgical resection is unknown and treatment remains subjective. Clinicians in conjunction with patients must balance the risks of recurrence against the potential risks associated with treatment. For those at very low risk of recurrence, no therapy may be needed; however, for patients at moderate risk immunomodulators should be considered. For those at highest risk of recurrence, biologic therapy, specifically antitumor necrosis factor agents, have emerged as appropriate treatment. Any postoperative management strategy should include a colonoscopy 6‐12 months after surgery to identify recurrence. This review discusses current evidence for various pharmacologic approaches in the prevention of postoperative recurrence and provides guidance for clarifying patient risk.

%Z Inflammatory bowel diseases

Journal article

%U https://cochrane.66557.net/central/doi/10.1002/central/CN-01762402/full

%0 Journal Article

%A Gendre, J. P.

%A Mary, J. Y.

%A Florent, C.

%A Modigliani, R.

%A Colombel, J. F.

%A Soulé, J. C.

%A Galmiche, J. P.

%A Lerebours, E.

%A Descos, L.

%A Viteau, J. M.

%D 1993

%T Maintenance treatment of Crohn's disease using orally administered mesalazine (Pentasa). A controlled multicenter study. The Study Groups on the Treatment of Inflammatory Digestive Disorders

%V 29

%N 5

%P 251‐256

%8 1993-01-01

%K Administration, Oral; Adult; Aminosalicylic Acids [administration & dosage, adverse effects, *therapeutic use]; Crohn Disease [*drug therapy, pathology, *prevention & control]; Double‐Blind Method; Drug Tolerance; Female; Humans; Male; Mesalamine; Patient Compliance; Placebos; Risk Factors

%X BACKGROUND: Mesalamine provides a new therapeutic approach in treating Crohn's disease. METHODS: To assess the efficacy and safety of slow‐release mesalamine (Pentasa) in maintaining remission in Crohn's disease, 161 patients with inactive disease were randomized to receive either Pentasa (2 g/day) or placebo in a 2‐year double‐blind, multicenter trial. Two strata were defined according to the duration of their remission: < 3 months (n = 64) or 3‐24 months (n = 97), presumed to be high and low relapse risk strata, respectively. RESULTS: The probability of relapse was higher in the short‐remission placebo group than in the three other groups (p < 0.003), showing there was a significant benefit from Pentasa in the high relapse risk stratum. In this stratum, the 2‐year on‐going remission rate was of 29% +/‐ 9% and 45% +/‐ 11% (mean +/‐ SD) in the placebo and Pentasa groups, respectively. The incidences of side effects were similar in both groups. CONCLUSION: Pentasa (2 g/day for 2 years) is a safe and effective maintenance treatment for Crohn's disease when given within 3 months of achieving remission.

%Z Annales de gastroenterologie et d'hepatologie

Journal article

%U https://cochrane.66557.net/central/doi/10.1002/central/CN-00097442/full

%0 Journal Article

%A Hamilton, A. L.

%A Kamm, M. A.

%A De Cruz, P.

%A Wright, E. K.

%A Feng, H.

%A Wagner, J.

%A Sung, JJY

%A Kirkwood, C. D.

%A Inouye, M.

%A Teo, S. M.

%D 2020

%T Luminal microbiota related to Crohn's disease recurrence after surgery

%V 11

%N 6

%P 1713‐1728

%8 2020-01-01

%R 10.1080/19490976.2020.1778262

%K *Crohn disease /drug therapy /surgery; *microflora; *recurrent disease; Adult; Article; Bacteria [classification, genetics, *isolation & purification]; Bioinformatics; Colon resection; Colonoscopy; Controlled study; Crohn Disease Activity Index; Crohn Disease [*microbiology, *surgery]; DNA extraction; Endoscopy; Enterobacteriaceae; Feces [microbiology]; Feces analysis; Female; Gastrointestinal Microbiome; Gene amplification; Gene sequence; Haemophilus; Human; Humans; Ileum [microbiology]; Lachnospiraceae; Major clinical study; Male; Metagenomics; Microbial community; Microbial diversity; Middle Aged; Middle aged; Multicenter study; Operation duration; Paired end sequencing; Polymerase chain reaction; Prospective Studies; Prospective study; Randomized controlled trial; Recurrence; Recurrence risk; Rutgeerts score; Scoring system; Small intestine resection

%X Background: Microbial factors are likely to be involved in the recurrence of Crohn’s disease (CD) after bowel resection. We investigated the luminal microbiota before and longitudinally after surgery, in relation to disease recurrence, using 16S metagenomic techniques. Methods: In the prospective Post‐Operative Crohn’s Endoscopic Recurrence (POCER) study, fecal samples were obtained before surgery and 6, 12, and 18 months after surgery from 130 CD patients. Endoscopy was undertaken to detect disease recurrence, defined as Rutgeerts score ≥i2, at 6 months in two‐thirds of patients and all patients at 18 months after surgery. The V2 region of the 16S rRNA gene was sequenced using Illumina MiSeq. Cluster analysis was performed at family level, assessing microbiome community differences between patients with and without recurrence. Results: Six microbial cluster groups were identified. The cluster associated with maintenance of remission was enriched for the Lachnospiraceae family [adjusted OR 0.47 (0.27–0.82), P = .007]. The OTU diversity of Lachnospiraceae within this cluster was significantly greater than in all other clusters. The cluster enriched for Enterobacteriaceae was associated with an increased risk of disease recurrence [adjusted OR 6.35 (1.24–32.44), P = .026]. OTU diversity of Enterobacteriaceae within this cluster was significantly greater than in other clusters. Conclusions: Luminal bacterial communities are associated with protection from, and the occurrence of, Crohn’s disease recurrence after surgery. Recurrence may relate to a higher abundance of facultatively anaerobic pathobionts from the Enterobacteriaceae family. The ecologic change of depleted Lachnospiraceae, a genus of butyrate‐producing bacteria, may permit expansion of Enterobacteriaceae through luminal environmental perturbation.

%Z Gut microbes

Journal article

%U https://cochrane.66557.net/central/doi/10.1002/central/CN-02140666/full

%0 Journal Article

%A Glick, L.

%A Sossenheimer, P. H.

%A Hirsch, A.

%A Hurst, R. D.

%A Cohen, R. D.

%A Hyman, N.

%A Rubin, D. T.

%D 2017

%T Low-dose metronidazole is associated with a decreased rate of endoscopic recurrence of Crohn's disease after ileal resection

%V 11

%N Supplement 1

%P S275

%8 2017-01-01

%R 10.1093/ecco-jcc/jjx002.514

%K *Crohn disease; *ileum resection; *metronidazole; *recurrence risk; Adult; Adverse drug reaction; Anastomosis; Cohort analysis; Conference abstract; Controlled clinical trial; Controlled study; Crohn disease; Drug therapy; Female; Follow up; Human; Low drug dose; Major clinical study; Male; Proportional hazards model; Retrospective study; Risk factor; Side effect; Surgery; Univariate analysis; adult; adverse drug reaction; anastomosis; cohort analysis; conference abstract; controlled clinical trial; controlled study; drug therapy; female; follow up; human; ileum resection; low drug dose; major clinical study; male; proportional hazards model; recurrence risk; retrospective study; risk factor; side effect; surgery; univariate analysis

%X Background: Recurrence of Crohn's disease (CD) after surgical resection and primary anastomosis is an important clinical challenge. Pre‐vious studies have demonstrated the benefit of imidazole antibiotics, but have been limited by adverse events and medication intolerance. We evaluated whether administration of low‐dose (LD) metronida‐zole (250 mg three times a day) for three months reduces endoscopic postoperative recurrence rates. Methods: We performed a retrospective cohort study of patients with Crohn's disease who underwent ileal resection with a primary anastomosis and subsequently received care at our center. We compared the cases who received LD metronidazole (primarily from one clini‐cian, DTR) to control patients (DTR and others) who did not receive this therapy. Data collected included demographics, risk factors for recurrence, and medications before and after surgery. The primary endpoint was the number of patients with >i2 (Rutgeerts) endoscopic recurrence by 12 months. Variables found to be predictive in univariate analysis at p<0.10 were introduced in the Cox model for multivariate analysis. Results: 70 Crohn's patients (35 cases) met inclusion criteria. Risk factors for Crohn's recurrence were similar between groups (Table 1). [Table Presented] Median time to endoscopic follow‐up for both groups was similar (cases: 184 days, IQR 178‐246; controls: 192 days, IQR 166‐250). The number of patients with >i2 endoscopic recurrence following ileal resection was significantly lower in the LD metronidazole group (7 of 35 patients; 20%) compared to the control group (19 of 35 patients; 54.3%) (p=0.0058) (). [Table Presented] Eight participants (22.86%) in the LD metronidazole group experi‐enced adverse events, and 3 of these patients (8.57%) discontinued the therapy. Conclusions: Low‐dose metronidazole for 3 months postoperatively significantly reduces endoscopic recurrence of CD and is safe and well‐tolerated. This intervention should be considered as a bridge to other therapies after ileocecetomy.

%Z Journal of Crohn's & colitis

Journal article; Conference proceeding

%U https://cochrane.66557.net/central/doi/10.1002/central/CN-01469791/full

%0 Journal Article

%A Feagan, B. G.

%A McDonald, J. W.

%A Rochon, J.

%A Laupacis, A.

%A Fedorak, R. N.

%A Kinnear, D.

%A Saibil, F.

%A Groll, A.

%A Archambault, A.

%A Gillies, R.

%D 1994

%T Low-dose cyclosporine for the treatment of Crohn's disease. The Canadian Crohn's Relapse Prevention Trial Investigators

%V 330

%N 26

%P 1846‐1851

%8 1994-01-01

%R 10.1056/NEJM199406303302602

%K Adult; Aminosalicylic Acids [therapeutic use]; Crohn Disease [*drug therapy]; Cyclosporine [*administration & dosage, adverse effects, therapeutic use]; Double‐Blind Method; Female; Follow‐Up Studies; Humans; Male; Mesalamine; Prednisone [therapeutic use]; Treatment Outcome

%X BACKGROUND: Long‐term corticosteroid therapy for Crohn's disease is associated with important types of morbidity, such as osteoporosis. Safe and effective alternative treatments are required. Although a short‐term benefit of cyclosporine in active Crohn's disease has been suggested, the long‐term safety and efficacy of this treatment have not been established. METHODS: We conducted a randomized, double‐blind, placebo‐controlled evaluation of the effect of 18 months of low‐dose cyclosporine treatment on the course of Crohn's disease. Adult patients whose disease had been active within the previous two years were randomly assigned to receive cyclosporine (151 patients) or placebo (154 patients) in addition to their usual therapy. Randomization was stratified according to center and score on the Crohn's Disease Activity Index (193 patients had scores of 150 or less, and 112 had scores greater than 150). The primary outcome measure was clinically important worsening of Crohn's disease, defined as a 100‐point increase in the Crohn's Disease Activity Index from the patient's base‐line value. Secondary outcomes were the use of prednisone and 5‐amino‐salicylates, mean score on the Crohn's Disease Activity Index and mean quality‐of‐life score, and the need for surgery. RESULTS: The condition of more patients worsened with cyclosporine than with placebo (91 of 151, or 60.3 percent, vs. 80 of 154, or 51.9 percent; P = 0.10). The median time to worsening of disease in patients receiving cyclosporine was 338 days, as compared with 492 days in patients receiving placebo (P = 0.25; relative risk, 1.22; 95 percent confidence interval, 0.86 to 1.72). Analyses of the mean Crohn's Disease Activity Index and quality‐of‐life scores and of the use of prednisone and 5‐aminosalicylates also failed to demonstrate benefit. CONCLUSIONS: In our patient population, the addition of low‐dose cyclosporine to conventional treatment for Crohn's disease did not improve symptoms or reduce requirements for other forms of therapy.

%Z New England journal of medicine

Journal article

%U https://cochrane.66557.net/central/doi/10.1002/central/CN-00101648/full

%0 Journal Article

%A Gross, V.

%A Andus, T.

%A Ecker, K. W.

%A Raedler, A.

%A Loeschke, K.

%A Plauth, M.

%A Rasenack, J.

%A Weber, A.

%A Gierend, M.

%A Ewe, K.

%A Et, Al.

%D 1998

%T Low dose oral pH modified release budesonide for maintenance of steroid induced remission in Crohn's disease. The Budesonide Study Group

%V 42

%N 4

%P 493‐496

%8 1998-01-01

%R 10.1136/gut.42.4.493

%K Administration, Oral; Adolescent; Adult; Aged; Anti‐Inflammatory Agents [*administration & dosage, therapeutic use]; Budesonide [*administration & dosage, therapeutic use]; Crohn Disease [*drug therapy]; Double‐Blind Method; Drug Administration Schedule; Female; Follow‐Up Studies; Humans; Male; Middle Aged; Recurrence; Remission Induction

%X BACKGROUND: The relapse rate after steroid induced remission in Crohn's disease is high. AIMS: To test whether oral pH modified release budesonide (3 x 1 mg/day) reduces the relapse rate and to identify patient subgroups with an increased risk of relapse. METHODS: In a multicentre, randomised, double blind study, 179 patients with steroid induced remission of Crohn's disease received either 3 x 1 mg budesonide (n = 84) or placebo (n = 95) for one year. The primary study aim was the maintenance of remission of Crohn's disease for one year. RESULTS: Patient characteristics at study entry were similar for both groups. The relapse rate was 67% (56/84) in the budesonide group and 65% (62/95) in the placebo group. The relapse curves in both groups were similar. The mean time to relapse was 93.5 days in the budesonide group and 67.0 days in the placebo group. No prognostic factors allowing prediction of an increased risk for relapse or definition of patient subgroups who derived benefit from low dose budesonide were found. Drug related side effects were mild and no different between the budesonide and the placebo group. CONCLUSION: Oral pH modified release budesonide at a dose of 3 x 1 mg/day is not effective for maintaining steroid induced remission in Crohn's disease.

%Z Gut

Journal article

%U https://cochrane.66557.net/central/doi/10.1002/central/CN-00684343/full

%0 Journal Article

%A Gross, V.

%A Andus, T.

%A Ecker, K. W.

%A Raedler, A.

%A Loeschke, K.

%A Plauth, M.

%A Rasenack, J.

%A Weber, A.

%A Gierend, M.

%A Ewe, K.

%A Et, Al.

%D 1998

%T Low dose oral pH modified release budesonide for maintenance of steroid induced remission in Crohn's disease

%V 42

%N 4

%P 493‐496

%8 1998-01-01

%K Budesonide ‐‐Clinical Trial ‐‐Ct/ Budesonide ‐‐Drug Dose ‐‐Do/ Budesonide ‐‐Drug Therapy ‐‐Dt/ Budesonide ‐‐Pharmaceutics ‐‐Pr/ Eudragit ‐‐Pharmaceutics ‐‐Pr/ Crohn Disease ‐‐Drug Therapy ‐‐Dt/ Controlled Drug Release/ Corticosteroid Therapy/ Remission/ Drug Efficacy/ Human/ Male/ Female/ Major Clinical Study/ Randomized Controlled Trial/ Double Blind Procedure/ Multicenter Study/ Controlled Study/ Adult/ Oral Drug Administration/ Article/ Priority Journal

%X Background‐ The relapse rate after steroid induced remission in Crohn's disease is high. Aims‐ To test whether oral pH modified release budesonide (3 x 1 mg/day) reduces the relapse rate and to identify patient subgroups with an increased risk of relapse. Methods‐ In a multicentre, randomised, double blind study, 179 patients with steroid induced remission of Crohn's disease received either 3 x 1 mg budesonide (n=84) or placebo (n=95) for one year. The primary study aim was the maintenance of remission of Crohn's disease for one year. Results‐Patient characteristics at study entry were similar for both groups. The relapse rate was 67% (56/84) in the budesonide group and 65% (62/95) in the placebo group. The relapse curves in both groups were similar. The mean time to relapse was 93.5 days in the budesonide group and 67.0 days in the placebo group. No prognostic factors allowing prediction of an increased risk for relapse or definition of patient subgroups who derived benefit from low dose budesonide were found. Drug related side effects were mild and no different between the budesonide and the placebo group. Conclusion‐ Oral pH modified release budesonide at a dose of 3 x 1 mg/day is not effective for maintaining steroid induced remission in Crohn's disease. Copyright © 2011 Elsevier B. V., Amsterdam. All Rights Reserved.

%Z Gut

Journal article

%U https://cochrane.66557.net/central/doi/10.1002/central/CN-00151424/full

%0 Journal Article

%A Prins, M. H.

%A Lensing, AWA

%A Prandoni, P.

%A Beyer-Westendorf, J.

%A Van Bellen, B.

%A Pap, A. F.

%A Gebel, M.

%A Verhamme, P.

%A Wells, P. S.

%A Weitz, J. I.

%D 2017

%T Long-term risk of recurrent venous thromboembolism with and without effective antithrombotic therapy

%V 1

%P 187

%8 2017-01-01

%R 10.1002/rth2.12012

%K *cancer recurrence; *venous thromboembolism; Adult; Anticoagulation; Body mass; Cancer patient; Cancer surgery; Conference abstract; Congestive heart failure; Controlled study; Creatinine clearance; Drug therapy; Family history; Female; Heredity; Hormonal therapy; Human; Immobilization; Incidence; Inflammatory bowel disease; Leg injury; Major clinical study; Major surgery; Malignant neoplasm; Paresis; Pregnancy; Puerperium; Randomized controlled trial; Relapse; Risk assessment; Risk factor; Thrombophilia; Travel

%X Background: There is uncertainty about the appropriate duration of anticoagulation in patients with venous thromboembolism (VTE) and practice varies between countries. Aims: To provide estimates of the risk of recurrent VTE, with and without effective antithrombotic therapy, according to the risk factor profile. Methods: We used data from two large randomized trials (EINSTEINExtension and EINSTEIN‐Choice) that compared rivaroxaban (20 mg or 10 mg) with placebo or aspirin (100 mg) for extended VTE treatment in patients who had already received 6 to 12 months of anticoagulation. The index VTE events were centrally classified using the following hierarchy: 1) unprovoked, or provoked with a 2) major permanent risk factor (i.e. cancer), 3) minor permanent risk factor (i.e. inflammatory bowel disease, lower extremity paralysis/paresis, congestive heart failure, BMI > 30, creatinine clearance < 50 ml/min, family history of VTE, hereditary/acquired thrombophilia), 4) minor transient risk factor (i.e. immobilization, travel > 8 h, hormonal therapy, pregnancy or puerperium, leg injury with impaired mobility, or 5) major transient risk factor (i.e. major surgery or trauma). Results: A total of 2832 patients received rivaroxaban (10 or 20 mg) and 1721 received placebo or aspirin. Conclusions: Although patients with unprovoked VTE are at highest risk of recurrence, those with a minor persisting or transient risk factor also are at a high risk. In patients with these risk profiles, rivaroxaban substantially reduces the incidence of recurrence. (Table Presented) .

%Z Research and practice in thrombosis and haemostasis

Journal article; Conference proceeding

%U https://cochrane.66557.net/central/doi/10.1002/central/CN-01653721/full

%0 Journal Article

%A Stocchi, L.

%A Milsom, J. W.

%A Fazio, V. W.

%D 2008

%T Long-term outcomes of laparoscopic versus open ileocolic resection for Crohn's disease: follow-up of a prospective randomized trial

%V 144

%N 4

%P 622‐7; discussion 627‐8

%8 2008-01-01

%R 10.1016/j.surg.2008.06.016

%K Adult; Anastomosis, Surgical [adverse effects, methods]; Colon [pathology, *surgery]; Crohn Disease [diagnosis, mortality, *surgery]; Female; Follow‐Up Studies; Humans; Ileum [pathology, *surgery]; Laparoscopy [adverse effects, *methods]; Laparotomy [adverse effects, *methods]; Male; Middle Aged; Pain, Postoperative [diagnosis]; Postoperative Complications [epidemiology]; Probability; Prospective Studies; Recurrence; Reoperation [statistics & numerical data]; Risk Assessment; Sensitivity and Specificity; Severity of Illness Index; Statistics, Nonparametric; Survival Rate; Time Factors; Treatment Outcome

%X BACKGROUND: The purpose of this study was to analyze long‐term recurrence rates and complications in patients previously enrolled in a prospective randomized trial comparing laparoscopic (LC) and open ileocolectomy (OC) for ileocolic Crohn's disease (CD). METHODS: Follow‐up data were available on 56 of 60 patients. Demographic data, recurrence rates, need for additional surgery related to primary procedure, and medication use were recorded. RESULTS: Mean follow‐up for 56 patients (27 LC vs 29 OC) was 10.5 years and comparable between LC and OC (10.0 vs 11.0, respectively; P = .64). One patient died 8 years after OC of causes unrelated to CD. Eight patients for each group underwent initial reoperative (26% LC vs 28% OC; P = .89). One patient underwent incisional hernia repair after LC (4%) versus 4 patients (14%) after OC (P = .61). Two patients in the LC group underwent adhesiolysis versus none after OC (P = .23). Incidences of anorectal disease, anorectal surgery, endoscopic or radiologic recurrence, and medication use were also similar between LC and OC. OC patients requiring operation during follow‐up were significantly more likely than LC to require multiple operations (P = .006). CONCLUSIONS: Long‐term data from this prospective randomized trial confirm that LC is at least comparable to OC in the treatment of ileocolic CD.

%Z Surgery

Journal article

%U https://cochrane.66557.net/central/doi/10.1002/central/CN-00651382/full

%0 Journal Article

%A Eshuis, E. J.

%A Slors, J. F.

%A Stokkers, P. C.

%A Sprangers, M. A.

%A Ubbink, D. T.

%A Cuesta, M. A.

%A Pierik, E. G.

%A Bemelman, W. A.

%D 2010

%T Long-term outcomes following laparoscopically assisted versus open ileocolic resection for Crohn's disease

%V 97

%N 4

%P 563‐568

%8 2010-01-01

%R 10.1002/bjs.6918

%K Adult; Body Image; Colectomy [*methods, mortality]; Crohn Disease [mortality, *surgery]; Female; Humans; Kaplan‐Meier Estimate; Laparoscopy [*methods, mortality]; Male; Middle Aged; Prospective Studies; Quality of Life; Recurrence; Reoperation; Treatment Outcome

%X BACKGROUND: Long‐term results of laparoscopically assisted versus open ileocolic resection for Crohn's disease were evaluated in a randomized trial. METHODS: Sixty patients who underwent ileocolic resection between 1999 and 2003 were followed prospectively. Primary outcomes were reoperation, readmission and repeat resection rates for recurrent Crohn's disease. Secondary outcomes were quality of life (QOL), body image and cosmesis. RESULTS: Five patients were lost to follow‐up. Median follow‐up was 6.7 (interquartile range 5.7‐7.9) years. Sixteen of 29 and 16 of 26 patients remained relapse free after ileocolic resection in the laparoscopic and open groups respectively (risk difference 6 (95 per cent confidence interval ‐ 20 to 32) per cent). Resection of recurrent Crohn's disease was necessary in two of 29 versus three of 26 patients (risk difference 5 (‐11 to 20) per cent). Overall reoperation rates for recurrent Crohn's disease, incisional hernia and adhesion‐related problems were two of 29 versus six of 26 (risk difference 16 (‐3 to 35) per cent). QOL was similar, whereas body image and cosmesis scores were significantly higher after laparoscopy (P = 0.029 and P < 0.001 respectively). CONCLUSION: Laparoscopically assisted ileocolic resection results in better body image and cosmesis, whereas open surgery is more likely to produce incisional hernia and obstruction.

%Z British journal of surgery

Journal article

%U https://cochrane.66557.net/central/doi/10.1002/central/CN-00734702/full

%0 Journal Article

%A Papamichael, K.

%A Rivals-Lerebours, O.

%A Billiet, T.

%A Casteele, N. V.

%A Gils, A.

%A Ferrante, M.

%A Assche, G. V.

%A Rutgeerts, P. J.

%A Mantzaris, G. J.

%A Peyrin-Biroulet, L.

%A Et, Al.

%D 2016

%T Long-term outcome of patients with ulcerative colitis and primary non-response to infliximab

%V 10

%N 9

%P 1015‐1023

%8 2016-01-01

%R 10.1093/ecco-jcc/jjw067

%K *C reactive protein; *albumin; *clinical study; *colon resection; *confidence interval; *hazard ratio; *infliximab; *infliximab/cm [Drug Comparison]; *infliximab/dt [Drug Therapy]; *infliximab/pd [Pharmacology]; *long term care; *probability; *proportional hazards model; *relapse; *treatment response; *ulcerative colitis; *ulcerative colitis/dt [Drug Therapy]; *ulcerative colitis/su [Surgery]; *vedolizumab; Adult; Adverse drug reaction; Albumin/ec [Endogenous Compound]; Article; C reactive protein/ec [Endogenous Compound]; Clinical practice; Clinical trial; Colon resection; Comparative study; Controlled clinical trial; Controlled study; Corticosteroid therapy; Disease duration; Drug blood level; Drug response; Drug withdrawal; Electronic medical record; Endogenous compound; Enzyme linked immunosorbent assay; Female; Follow up; Health care quality; Human; Human tissue; Immunoadsorption; Major clinical study; Male; Middle aged; Multicenter study; Observational study; Outcome assessment; Patient referral; Priority journal; Retrospective study; Side effect; Sigmoidoscopy; Tumor necrosis factor; Ulcerative colitis/dt [Drug Therapy]; Vedolizumab/cm [Drug Comparison]

%X Background and Aims: We studied the long‐term outcome of patients with ulcerative colitis [UC] and primary non response [PNR] to infliximab and searched for predictors of colectomy in these patients. Methods: This retrospective, multi‐centre study included UC patients from three European referral centres, with PNR to infliximab defined as a lack of clinical improvement after the induction therapy, leading to drug discontinuation. Relapse, for patients who continued on biologicals after PNR to infliximab, was defined as drug discontinuation for PNR, loss of response, or serious adverse event. Serum infliximab concentrations at Weeks 2 and 6 were evaluated using an enzyme‐linked immunosorbent assay [ELISA] developed in house. Results: The study population consisted of 99 anti‐tumour necrosis factor [TNF]‐naive patients with UC and PNR to infliximab. At the end of follow‐up (median: 3.2 [interquartile range 1‐6.3] years), 55 [55.6%] of these patients underwent colectomy. Multiple Cox regression analysis identified acute severe UC (hazard ratio [HR]: 24; 95% confidence interval [CI]: 2.5‐231; p = 0.006], baseline C‐reactive protein [CRP] > 5 mg/l [HR: 11; 95% CI: 2.1‐58.8; p = 0.005], baseline albumin < 40 g/l [HR: 9.5; 95% CI: 1.3‐71.4; p = 0.026], and infliximab concentration at Week 2 < 16.5 mug/ml [HR: 5.6; 95% CI: 1.1‐27.8; p = 0.034] as independent predictors of colectomy. Regarding patients who continued on biologicals after PNR to infliximab, there was a marginally higher cumulative probability for relapse in patients switching to another anti‐TNF agent compared with those swapping to vedolizumab [p logrank = 0.08]. Conclusions: About half of UC patients with PNR to infliximab will undergo colectomy. Patients with severe inflammation and low serum infliximab concetrations during the induction phase are at greatest risk. Copyright © 2016 European Crohn's and Colitis Organisation (ECCO). Published by Oxford University Press. All rights reserved.

%Z Journal of Crohn's & colitis

Journal article

%U https://cochrane.66557.net/central/doi/10.1002/central/CN-01329435/full

%0 Journal Article

%A El-Hachem, S.

%A Regueiro, M.

%A Kevin, K.

%A Schraut, W.

%A Baidoo, L.

%A Harrison, J.

%A Pesci, M.

%A Watson, A.

%A Binion, D.

%D 2009

%T Long-term follow-up of patients enrolled in the randomized controlled trial (RCT) of infliximab for prevention of recurrent Crohn's disease (CD)

%V 15

%P S11

%8 2009-01-01

%R 10.1002/ibd.21172

%K *Crohn disease; *colitis; *enteritis; *follow up; *non profit organization; *patient; *prevention; *randomized controlled trial; Colonoscopy; Endoscopy; Gastroenterology; Infusion; Intestine resection; Prevention study; Recurrence risk; Remission; Surgery

%X BACKGROUND: A single center RCT in CD pts with intestinal resection found that infliximab was more effective than placebo in preventing 1 yr endoscopic, histologic, and clinical CD recurrence (Gastroenterology 2009;136:441‐450). Upon completion of the 1 yr trial, pts could receive open label infliximab based on endoscopic activity. Long‐term follow up data (max 4 yrs) are provided and are important in determining whether: 1) infliximab is effective beyond the 1st postop year, 2) infliximab may be stopped at one year and 3) infliximab is effective in treating endoscopic recurrence in pts who had been on placebo, ie whether infliximab is as effective in response to recurrence rather than as a preventive measure after surgery. AIMS: To examine long‐term endoscopic CD remission and recurrence rates after surgery in the postop prevention trial. METHODS: There were 24 pts in the original 1 yr trial: 13 randomized to placebo and 11 to infliximab. At the completion of the trial, pts had a colonoscopy and were offered open‐label infliximab. Another colonoscopy was performed 6‐12 months after completion of the post‐op trial and planned for yearly thereafter. Endoscopic scoring was the same for follow‐up as in the trial; ileal (i) score of i0 or i1 consistent with endoscopic remission and i2, i3, or i4 with endoscopic recurrence. All pts included in the long‐term follow up evaluation had at least one colonoscopy at post op year 2 and/or 3. RESULTS: To date, 2/3/4‐year follow‐up has been performed in 16/6/2 pts, respectively. At post op yr 1 (end of RCT), 7 of the placebo pts opted for open label infliximab with 5 (71%) being in remission at 2‐year follow‐up. In contrast, at post op yr 1, 3 infliximab pts stopped treatment, and all showed evidence of recurrence at 2‐year endoscopy. At year 3, one patient was switched from infliximab to adalimumab due to infusion reaction, and maintained remission. Among the 24 pts, a total of 48 post‐surgical endoscopic evaluations has been performed to date with most recent treatment regimen classified as: 25 anti‐tumor necrosis factor (antiTNF: Infliximab or Adalimumab) (52%), 18 no‐antiTNF (38%), and 5 partial antiTNF (10%), defined as prior antiTNF but not within 8 weeks of endoscopy. Throughout long‐term follow‐up, there was a strong gradient relationship between antiTNF treatment (antiTNF/partial antiTNF/no‐antiTNF) and rate of endoscopic remission (table). These finding were unaffected by concomitant use of Imuran/6MP or 5 ASA agents. CONCLUSIONS: Based on long‐term follow‐up from the post‐op study, we conclude that: 1) pts treated with infliximab after surgery maintain remission with ongoing infusions, but recur if infliximab is stopped, 2) anti‐TNF naïve pts who develop endoscopic CD recurrence 1 yr after resective surgery may be effectively treated with infliximab.

%Z Inflammatory bowel diseases

Journal article; Conference proceeding

%U https://cochrane.66557.net/central/doi/10.1002/central/CN-01742751/full

%0 Journal Article

%A Ortiz, O.

%A Daca Alvarez, MDLA

%A Rivero Sanchez, L.

%A Balaguer, F.

%A Cuatrecasas, M.

%A Moreta Saa, M. J.

%A Saez De Gordoa, K.

%A Pellise Urquiza, M.

%D 2022

%T LINKED-COLOR IMAGING VERSUS HIGH-DEFINITION WHITE LIGHT ENDOSCOPY FOR EVALUATION OF POST-POLYPECTOMY SCARS OF NON-PEDUNCULATED LESIONS. LCI-SCAR STUDY

%V 10

%P 220

%8 2022-01-01

%R 10.1002/ueg2.12294

%K *histology; *polypectomy; *severe cutaneous adverse reaction; *white light endoscopy; Adult; Blue light; Colonoscopy; Conference abstract; Confirmation bias; Controlled study; Diagnosis; Diagnostic test accuracy study; Endoscopist; False positive result; Female; Gold standard; Histopathology; Human; Human tissue; Inflammatory bowel disease; Informed consent; Major clinical study; Male; Predictive value; Prospective study; Randomized controlled trial; Scar; Serrated adenoma; Splenic flexure; Surgery

%X Introduction: Early detection and treatment of recurrence after piecemeal endoscopic mucosal resection of non‐pedunculated colorectal polyps is crucial to avoid unnecessary surgeries and post‐colonoscopy cancer. ESGE recommends the use of conventional or virtual chromoendoscopy for assessing piecemeal polypectomy scar site. Linked Color Imaging is a new light modality (LCI) that has demonstrated to improve polyp and adenoma detection in average risk population but has never been assessed for evaluation of post‐polypectomy scars. Aims & Methods: We aimed at comparing the sensitivity and negative predictive value of LCI in comparison with white light endoscopy (WLE) for detection of post‐polypectomy recurrence. In this single center, prospective, cross‐over trial (NCT04899700) eligible patients ≥ 18 years undergoing surveillance colonoscopy (up to third) after polypectomy of ≥15mm polyps that did not meet exclusion criteria (inflammatory bowel disease and inability/refuse to sign informed consent) were randomized 1:1 to either LCI followed by WLE (LCI‐WLE group) or WLE followed by LCI (WLE‐LCI group). A maximum of three scars per patient were included. Two blinded independent endoscopists assessed each scar with one of randomized light modalities followed by blue light imaging (BLI). This method allowed that each scar was its own control reducing variability and confirmation bias. The endoscopists made a diagnosis of recurrence (yes or no) with a level of confidence (high or low). Suspected recurrences were resected, and suspected normal scars were biopsied. Histology was used as gold standard.Based on previous literature, assuming a sensitivity of 90% for WLE and 95% for LCI for recurrence detection, 165 scars were needed to achieve a power of 80% and alpha 5%. Results: One hundred twenty nine patients with 173 scars LCI‐WLE (N=93) and WLE‐LCI (N=80) were included. Baseline patient, lesion and procedural characteristics were similar in both arms. Median size of scars was 12 mm (interquartile range +/‐ 5 mm). 76.9% (133) were located proximal to splenic flexure, 48.3% (84) raised from adenoma, 47.1% (81) from serrated lesions and 4.6% (8) from pT1. In 27% (47) of the scars the indication was second or third surveillance after polypectomy. In 2.3% (4) of the scar's clips were placed at baseline procedure. Based on histology, 32.4% (56) scars presented recurrence: 50.9% (27) of them were ≤5mm in size, 62.5% (35) had sessile morphology and 37.5 % (21) were flat‐elevated. Regarding histology of recurrences 48.2% (27) were adenomas and 51.8% (29) serrated. Diagnosis values for optical diagnosis of LCI (+/‐BLI) and WLE (+/‐BLI) for recurrence based on histology are shown in Table 1.Paired concordance between light modalities was 96% (166/173). In the discordant cases, LCI identified 4 true positive cases not detected by WLE and 2 false positives correctly classified with WLE whereas WLE detected one false positive correctly classified by LCI. Conclusion: LCI was superior to WLE and to WLE+BLI for detection of recurrence in post‐polypectomy sites.

%Z United European gastroenterology journal

Journal article; Conference proceeding

%U https://cochrane.66557.net/central/doi/10.1002/central/CN-02496475/full

%0 Journal Article

%A NCT

%D 2022

%T Ligation of Intersphincteric Fistula Tract Versus Rectal Advancement Flap in the Treatment of Complex Anal Fistula

%8 2022-01-01

%K Fistula; Rectal Fistula

%X Material and method Design: Phase III, single centre, randomised, open‐label, parallel group trial controlled with active comparator, with blind evaluation of results by a non‐commercial third party. This study was conducted in 72 patients who had undergone complex anal fistula surgery and met the inclusion criteria. The study was conducted by the Colo proctology Unit of the General and Digestive Surgery Department of Hospital General Universitario Reina Sofia, Murcia, Spain, from February 2013 to June 2016, and approved by the hospital's Independent Ethics Committee (IEC). The study was conducted pursuant to the principles of the Declaration of Helsinki (Seoul, October 2008) and the Good Clinical Practice (GP) standards of the European Economic Community's task force on the efficacy of medicinal substances (1990) and all legislation currently applicable in Spain (Royal Decree 223/2004 of 6 February). Patients with a diagnosis or suspected diagnosis of anal fistula were referred to the Coloproctology Office. At the first visit, demographic data and a clinical history was obtained, followed by a physical examination of the anal region, with rigid anoscopy, ordering an endoanal ultrasound. At a second visit, if the diagnosis of complex anal fistula was confirmed, after providing information about the study, the patient was invited to participate, signed the consent form, and underwent the pre‐operative Wexner scale. Patients were allocated a randomisation code for LIFT or RAF on the day of surgery. After surgery, controls were performed on days 7 and 14, by means of a symptoms diary to be completed by each patient, and 1, 3, 6 and 12 months after the surgery, with the Wexner scale calculated in the last control of each patient. Complex anal fistula was defined as a fistula at risk of affecting continence involving more than 30% of the sphincteric apparatus. Infection of surgical site at or near the incision in the 30 days following surgery (28). Haematoma was defined as accumulation of blood in the surgical area, with bluish or violet colouring of the affected area; surgical wound dehiscence was defined as the spontaneous separation of the edges of the wound, in the absence of surgical site infection. The inclusion criteria were patient diagnosed with transsphincteric (medium or high) or suprasphincteric cryptoglandular anal fistula, over 18 years of age, with no abscess at the time of surgery, who agreed to participate and signed the informed consent form, and was capable transsphincteric understanding and following study instructions. The exclusion criteria were minor patient, diagnosed with, or with a suspected diagnosis of, Crohn's disease, malignant anal tumour, tuberculosis or suppurative hidradenitis, a history of radiotherapy of the ano‐perineal region, language difficulties that prevent comprehension of the study, or refusal to participate. Each patient's data were collected in the case report form and transferred to a database for their statistical analysis by an analyst unrelated to the study, preserving their anonymity and the treatment group to which they belonged. Study objectives: The primary objective was to show the non‐inferiority in therapeutic efficacy of LIFT versus RAF in the surgical treatment of CAF. The primary efficacy endpoint was the proportion of subjects in each treatment group without fistula recurrence 12 months after surgery. The secondary objectives were to show the non inferiority of LIFT versus RAF in therapeutic safety: ‐ Evaluating functionality, both before and after surgery, in both treatment groups, through anal continence, using the Wexner scale. ‐ Comparing post‐operative complications in both treatment groups: infection of surgical site, haematoma and surgical wound dehiscence. ‐ Comparing time to fistula relapse in both treatment groups, by a survival analysis. ‐ Analysing hospital management factors (duration of surgery and hospitalisation) in both treatment groups. Randomisation and surgeries: The subjects were randomised 1:1 to receive LIFT or RAF surgery. Patient randomisation was by randomisation in balanced six‐cell blocks; the sequence was custodied by the Teaching Secretary, unrelated to the study. The surgeon requested each patient's randomisation code on the day of surgery. LIFT or RAF. The patients were operated by three surgeons from the Coloproctology Unit, whose learning curve was based on unifying experimental technique (LIFT) criteria in six patients before the start of the study. All the surgeries used epidural anaesthesia. ‐ Ligation of the intersphincteric fistula tract (LIFT): jackknife position. Location of IFO by injecting a diluted (1/3) solution of hydrogen peroxide through the external fistula orifice (EFO). Opening of the fistula trajectory with a stylus. Curved 2.5 cm incision in the intersphincteric space until trajectory is skeletalised, ligation of internal and external sphincter with 3/0 single thread polyglecaprone 25 absorbable suture (MonocrylTM Plus®), exeresis of the trajectory between the ligations with cold scalpel. Cleansing of distal part with curette and enlargement of the external fistula orifice (EFO). Closure of the muscular intersphincteric plane with loose stitches of 3/0 polyglecaprone 25 single‐thread absorbable suture (MonocrylTM Plus®). Closure of intersphincteric skin with simple plaited polyglycolic acid absorbable sutures (SSA90®). ‐ Rectal advancement flap (CAR): jackknife in case of anterior fistula and lithotomy in case of posterior fistula. Location of IFO by injecting a diluted (1/3) solution of hydrogen peroxide through EFO. Opening of the fistula trajectory with a stylus. Excision of the extrasphincteric component of the fistula and cleansing of sphincteric tract. Design of a flap with electric scalpel, of partial thickness including mucosa‐submucosa and part of the fibres of the internal sphincter, with a broad base (twice the apex), sliding in a craniocaudal direction, without tension, to completely cover the internal part of the fistula, closure of the internal orifice with cross stitch and suture of flap with simple 3/0 polyglecaprone 25 absorbable suture (MonocrylTM Plus®). Peri‐operative care and follow‐up: All patients were advised to follow a waste‐free diet 48 hours before admission, and were given an enema on the night before surgery. All patients received antibiotic prophylaxis with IV amoxicillin/clavulanic acid 2 g half an hour before the start of surgery. Six hours after surgery, liquid diet commenced, progressing to semi‐bland for dinner that night, and bland on the first day after surgery. No postoperative antibiotic treatment was indicated. The ward surgeon, unrelated to the study, discharged the patient based on these criteria: afebrile, controlled pain, appropriate oral tolerance and surgical wound correct. All patients received 12 months of follow‐up, except for one who left the study after 8 months. After discharge, the patient attended for cleaning of the surgical wound on the following two Fridays, with review of the symptom diary (SD) and evaluation of possible complications by the Coloproctology Unit surgeon. The patient attended a scheduled appointment one month after surgery (1st post‐op visit), where he was evaluated and data was collected in the case report form (CRF), the SD was collected and possible complications were evaluated (infection of surgical site, haematoma, wound dehiscence). New appointments were scheduled at 3 (2nd post‐op visit) and 6 months (3rd post‐op visit), where the patient was evaluated and the respective data were collected. The final visit (4th post‐op visit) took place at 12 months, where the post‐op Wexner scale was applied. Sample size and statistical analysis: When the study began, publications reported a relapse rate of 0‐33% with rectal advancement flap, with an average of 19% (19) and we can assume that the response rate to surgical treatment was approximately 85% for RAF, while the expected response rate for LIFT was 70%, the average of the rates observed in clinical studies published to date, which ranged from 57% to 94% (19). With these data, considering response as the absence of fistula recurrence at 12 months, and in order to obtain a power of 80% to reject the null hypothesis (Ho), that the difference between proportions p1 and p2 was lower than the non‐inferiority limit, by a normal asymptotic test for proportions, unilateral for two independent samples, considering that the level of significance was 0.5, assuming that the proportion in the reference group was 85%, the proportion in the experimental group was 70%, the proportion of patients in the reference group relative to the total was 50% and the non‐inferiority limit was 10% and the expected withdrawal rate was 5%, 36 patients were required for the reference group and 36 for the experimental group, making a total of 72 patients in the study. The categorical variables were described with mean values and percentages. The continuous variables were described with mean values and standard deviation, or median values and range. The Chi‐square or Fisher test was used to compare two qualitative variables, to see whether there was dependence between them. The Bonferroni correction was used for column comparisons. The Student's t‐test was used for comparison of the two groups, after checking the normality and homogeneity of variances assumptions with the Kolmogorov‐Smirnov and Levene tests, respectively. For the analysis of non‐inferiority of the primary and categorical variables, the calculation was through the Farrington‐Manning score and Miettinen‐Nurminen Score tests, with a level of significance of 0.05 and a non‐inferiority limit of 0.10. For the continuous variables, non‐inferiority was evaluated by difference of means, with the Mann‐Whitney U‐test and the Wilcoxon test. All statistical comparisons used a bilateral test with a level of significance of p < 0.05. The primary study analysis was based on the intention‐to‐treat population.

%Z https://clinicaltrials.gov/show/NCT05263661

Trial registry record

%U https://cochrane.66557.net/central/doi/10.1002/central/CN-02382125/full

%0 Journal Article

%A NCT

%D 2017

%T Lengthening Adalimumab Dosing Interval in Quiescent Crohn's Disease Patients

%8 2017-01-01

%K Adalimumab; Crohn Disease

%X Rationale Adalimumab is both an effective induction and maintenance therapy for Crohn's disease (CD). Due to the risk of side effects (infections, injection reaction) and high costs, an extension of the injection interval is an attractive option. However, this strategy has not been evaluated yet in a randomized controlled trial in CD patients. Objective To assess non‐inferiority of extending the adalimumab dosing interval, under strict disease monitoring in CD patients in sustained (>12 months) clinical remission, compared to standard care. Study design Multicenter, randomized controlled, open label non‐inferiority trial, with two treatment arms. Study population Crohn's disease patients, in sustained clinical remission on adalimumab maintenance therapy. Intervention Intervention arm: The adalimumab injection interval during maintenance therapy (40 mg per 2 weeks) will be extended through a stepwise disease activity guided manner to 3 weeks and subsequently ‐ after 24 weeks ‐ to 4 weeks. If a step‐down leads to recurrence of disease activity patients will return to the preceding effective dosing interval. Control arm: patients will continue adalimumab treatment following their usual dose and schedule. Treatment decisions are made at the discretion of the treating physician who will be blinded for the results of additional assessments beyond standard of care. Main study parameters/endpoints Primary outcome: The difference in cumulative incidence of pre‐defined persistent flares between the dose reduction and usual care groups at 48‐week follow‐up. A persistent flare is defined as increased inflammatory markers (FC >250 μg/g and/or CRP ≥10 mg/L) combined with patient‐reported symptoms (HBI ≥ 5), persisting for >8 weeks despite dose escalation of adalimumab.

%Z https://clinicaltrials.gov/show/NCT03172377

Trial registry record

%U https://cochrane.66557.net/central/doi/10.1002/central/CN-01494610/full

%0 Journal Article

%A Luglio, G.

%A Rispo, A.

%A Tropeano, F. P.

%A Giglio, M. C.

%A Cricri, M.

%A Imperatore, N.

%A De Sire, R.

%A Guarino, A. D.

%A Bucci, L.

%A Castiglione, F.

%A Et, Al.

%D 2023

%T Kono-S anastomosis reduces endoscopic and surgical post-operative recurrence in Crohn's disease. The SuPREMe-CD Trial Update

%V 17

%P i650

%8 2023-01-01

%R 10.1093/ecco-jcc/jjac190.0652

%K *Crohn disease; *anastomosis; Adult; Colonoscopy; Conference abstract; Controlled study; Drug safety; Female; Follow up; Human; Major clinical study; Male; Outpatient; Randomized controlled trial; Recurrence risk; Sample size; Side to side anastomosis; Surgery; Tertiary care center

%X Background: Recently, a new antimesenteric, functional, end‐to‐end, hand‐sewn ileocolic anastomosis (Kono‐S) has shown a significant reduction in endoscopic recurrence (ER) and surgical recurrence (SR) rate in Crohn's disease (CD). This trial aimed to provide randomized controlled data comparing Kono‐S anastomosis and conventional stapled ileocolic side‐to‐side anastomosis. Methods: Randomized controlled trial (RCT) at a tertiary referral centre. The primary endpoint is ER rate (Rutgeerts score ≥ i2) after 6 months and surgical recurrence at 60 months. Secondary endpoints are clinical recurrence (CR) rate after 24 and 36 months, and SR after 24 and 36 months. Statistics was performed by using standard analyses. Furthermore, a sample size able to consent the detection of a reduction >30% in total endoscopic recurrence (when assuming a 60% to 65% ER expected rate in the control group and a 30% in the case group) was calculated; to allow a 10% drop‐out of patients, 36 patients per group were needed (72 patients in the entire population). Results: A total of 119 CD patients were included in the study. In all, 51 ileocolic CD patients were randomized in the Kono group and 68 in the conventional side‐to‐side group. At 6 months, 17 (33.3%) patients in the Kono group and 51 (75%) patients in the conventional group presented an ER (Rutgeerts score≥i2) at colonoscopy (p<0.001). A severe post‐operative ER (Rutgeerts score≥i3) was found in 8 (15.7%) patients in the Kono group versus 23 (33.8%) patients in the conventional group (p=0.03). At 24 months, CR rate was 19.6% in the Kono group versus 30.9% in the conventional group (p=0.2), while SR rate was 0% in the Kono group versus 4.4% (3 patients) in the standard group (p=0.2). At 36 months, CR rate was 19.6% in the Kono‐s group versus 33.8% in the conventional group (p=0.1). At 36 months, no patients (0%) in the Kono group experienced SR, while 6 patients (8.8%) in the conventional group did (p=0.03). Furthermore, when considering a median follow‐up of 54 (42‐60) months at interim analysis of SR, the difference between the two groups was confirmed to be significant (0% vs 8.8%; p=0.02). Conclusion: This is the first RCT comparing Kono‐S anastomosis and conventional side‐to‐side anastomosis in CD. The results demonstrate a significant reduction in postoperative endoscopic and surgical recurrence rates, no safety issues, and a favourable trend for lower clinical recurrence rate in patients who underwent Kono‐S anastomosis compared to the conventional side‐to‐side anastomosis.

%Z Journal of Crohn's & colitis

Journal article; Conference proceeding

%U https://cochrane.66557.net/central/doi/10.1002/central/CN-02526687/full

%0 Journal Article

%A Chaparro, M.

%A Garcia Donday, M.

%A Riestra, S.

%A Lucendo, A. J.

%A Benitez, J. M.

%A Navarro-Llavat, M.

%A Barrio, J.

%A Morales-Alvarado, V. J.

%A Rivero, M.

%A Busquets, D.

%A Et, Al.

%D 2023

%T Is the withdrawal of anti-tumour necrosis factor in inflammatory bowel disease patients in remission feasible without increasing the risk of relapse? Results from the randomised clinical trial of GETECCU (EXIT)

%V 17

%P i50‐i54

%8 2023-01-01

%R 10.1093/ecco-jcc/jjac190.0037

%K *inflammatory bowel disease; *relapse; *remission; Adult; Clinical trial; Cohort analysis; Conference abstract; Controlled study; Crohn disease; Data quality; Drug therapy; Drug withdrawal; Feces; Female; Follow up; Human; Intravenous drug administration; Major clinical study; Male; Multicenter study; Prospective study; Randomized controlled trial; Ulcerative colitis

%X Background: The feasibility of anti‐TNF discontinuation in inflammatory bowel disease (IBD) must be proven in clinical trials including patients in clinical, endoscopic, and radiologic remission at the time of anti‐TNF withdrawal to make recommendations for clinical practice Aims: Primary: To compare the rates of clinical remission at 1 year in patients who discontinue anti‐TNF treatment vs. those who continue treatment. Secondary objectives: To know the effect of anti‐TNF withdrawal on relapse‐free time, mucosal healing and safety; and to identify predictive factors for relapse Methods: Prospective, quadruple‐blind, multicentre, randomised controlled trial. Patients with ulcerative colitis (UC) or Crohn's disease (CD) in clinical remission for > 6 months were randomised to maintain anti‐TNF treatment [maintenance arm (MA)] or to withdraw it [withdrawal arm (WA)]. Patients who were on infliximab (IFX) received IFX 5 mg/kg or an intravenous placebo every 8 weeks, while patients on adalimumab (ADA) received subcutaneous ADA 40 mg or placebo every other week. Patients were followed‐up until month 12 or up to the time of clinical relapse, whichever came first. Inclusion and exclusion criteria, trial scheme and definitions are summarized in figures 1a, 1b and 1c. Results were analysed by intention‐to‐treat (ITT) and by per‐protocol (PP). Local investigators were blinded to faecal calprotectin (FC) and IFX and ADA trough levels. On‐site monitoring was performed to assess data quality Results: 159 patients were screened, from whom 140 were randomised and comprised the ITT cohort: 70 allocated to the MA and 70 to the WA. Fifteen patients dropped out before the end of follow‐up (12 months or relapse), leaving 63 patients in the MA and 62 patients in the WA for the PP analysis. The characteristics of patients in the MA and WA were similar (figure 2). The proportions of patients who maintained clinical remission ‐59/70 (84%) 95% confidence interval (CI)=74‐92% in the MA vs. 53/70 (76%) 95%CI=64‐85% in the WA‐ and who remained without significant endoscopic lesions at the end of follow‐up were similar between groups (figures 3a, 3b, 3c). Only the proportion of patients with FC >250 mg/g was higher in the WA at the end of follow‐up (figure 3d) Maintenance of clinical remission was no different between groups (figure 4). The same percentage of patients in both groups had at least one adverse event (69%). The proportion of patients with serious adverse events was also similar between groups (4% in MA vs 7% in WA) Conclusion: Anti‐TNF withdrawal in selected IBD patients in clinical endoscopic, and radiologic remission could be feasible without an increase in the risk of clinical relapse. Long‐term follow‐up of these patients is warranted.

%Z Journal of Crohn's & colitis

Journal article; Conference proceeding

%U https://cochrane.66557.net/central/doi/10.1002/central/CN-02526785/full

%0 Journal Article

%A Wright, E. K.

%A De Cruz, P. P.

%A Kamm, M. A.

%A Hamilton, A. L.

%A Ritchie, K. J.

%A Krejany, E. O.

%A Gorelik, A.

%A Liew, D.

%A Prideaux, L.

%A Lawrance, I. C.

%A Et, Al.

%D 2014

%T Intestinal resection in Crohn's disease is associated with significant and durable improvement in health related quality of life although to a lesser extent in women and smokers. Results from the POCER study

%V 8

%P S56‐S57

%8 2014-01-01

%K *Crohn disease; *colitis; *female; *human; *intestine resection; *quality of life; *smoking; Adalimumab; Calgranulin; Clinical Disease Activity Index; Colonoscopy; Crohn disease; Diseases; Drug effect; Drug therapy; Emotional stress; Functional status; Healing; Health; High risk patient; Male; Mercaptopurine; Metronidazole; Mucosal disease; Patient; Population; Questionnaire; Remission; Surgery; Therapy; colitis; colonoscopy; diseases; drug effect; drug therapy; emotional stress; female; functional status; healing; health; high risk patient; human; intestine resection; male; mucosal disease; patient; population; quality of life; questionnaire; remission; smoking; surgery; therapy

%X Background: Health‐related QoL (HRQoL) measures perceptions, illness experience, and functional status. Crohn's disease patients have lower HRQoL, poorer function and more emotional distress than healthy individuals, even when in remission. HRQoL improves with medically or surgically induced remission. We evaluated the effects of surgery on HRQoL. Methods: Mucosal healing was the target in the Post‐Operative Crohn's Endoscopic Recurrence (POCER) treat‐to‐target study. Drug treatments included metronidazole, and thiopurine or adalimumab for high risk patients (smoker, perforating disease, ≥2nd operation). Patients were randomised to colonoscopy at 6 months with intensified treatment for endoscopic recurrence (“active care”) or no colonoscopy (“standard care”). All patients were colonoscoped at 18 months. HRQoL was assessed with a general (SF36) and disease‐specific (IBDQ) questionnaire pre‐operatively and at 6, 12 and 18 months. CRP, CDAI and faecal calprotectin (FC) were measured longitudinally. Results: 174 patients (median age 38, 55% female) were included. HRQoL was poor pre‐operatively: median SF36 = 40 (where maximum = 100, Australian normal = 70‐90) and IBDQ = 120 (maximum = 224, average score in Australian Crohn's disease patients=156). For all patients both SF36 and IBDQ improved significantly at 6 months to 78 and 178 respectively, and this was sustained at 12 months (81 and 183) and 18 months (80 and 182 respectively). Females had lower HRQoL than males post‐op at 6 (SF36 p = 0.012; IBDQ p = 0.007) and 12 months (SF36 p = 0.001, IBDQ p = 0.006). Smokers had poorer HRQoL compared to non‐smokers at both 12 and 18 months: SF36 at 12 month p = 0.002, and IBDQ at 12 and 18 months (p = 0.046, p = 0.047 respectively). Persistent endoscopic remission, thiopurine or adalimumab therapy and treatment step up were not associated with changes in HRQoL. There was a significant inverse correlation between CDAI and both SF‐36 and IBDQ at 6, 12 and 18 months. HRQoL did not correlate with endoscopic remission, CRP or FC. Conclusions: Intestinal resection of all macroscopic Crohn's disease, with a focus on maintaining remission, is associated with significant and sustained improvement in general and disease‐specific HRQoL. The lower HRQoL in female patients and smokers may reflect partly their lower QoL in the healthy and IBD populations, but this requires further investigation. A higher clinical disease activity index, but not direct measures of active disease or type of drug therapy, is associated with a lower HRQoL, suggesting that symptoms reflect subjective personal factors and not active mucosal disease or drug effects.

%Z Journal of Crohn's & colitis

Journal article; Conference proceeding

%U https://cochrane.66557.net/central/doi/10.1002/central/CN-01057526/full

%0 Journal Article

%A Wright, E. K.

%A De Cruz, P.

%A Kamm, M. A.

%A Hamilton, A. L.

%A Ritchie, K. J.

%A Krejany, S.

%A Gorelik, A.

%A Liew, D.

%A Prideaux, L.

%A Lawrance, I. C.

%A Et, Al.

%D 2014

%T Intestinal resection in crohn's disease is associated with significant and durable improvement in health related quality of life although to a lesser extent in women and smokers. Results from the POCER study

%V 146

%N 5

%P S‐435

%8 2014-01-01

%R 10.1016/S0016-5085(14)61564-X

%K *Crohn disease; *female; *gastrointestinal disease; *human; *intestine resection; *quality of life; *smoking; Adalimumab; Calgranulin; Clinical Disease Activity Index; Colonoscopy; Crohn disease; Diseases; Drug effect; Drug therapy; Emotional stress; Functional status; Healing; Health; High risk patient; Male; Mercaptopurine; Metronidazole; Mucosal disease; Patient; Population; Questionnaire; Remission; Surgery; Therapy; colonoscopy; diseases; drug effect; drug therapy; emotional stress; female; functional status; gastrointestinal disease; healing; health; high risk patient; human; intestine resection; male; mucosal disease; patient; population; quality of life; questionnaire; remission; smoking; surgery; therapy

%X Introduction: Health‐related QoL (HRQoL) measures perceptions, illness experience, and functional status. Crohn's disease patients have lower HRQoL, poorer function and more emotional distress than healthy individuals, even when in remission. HRQoL improves with medically or surgically induced remission. We evaluated the effects of surgery on HRQoL. Materials and Methods: Mucosal healing was the target in the Post‐Operative Crohn's Endoscopic Recurrence (POCER) treat‐to‐target study. Drug treatments included metronidazole, and thiopurine or adalimumab for high risk patients (smoker, perforating disease, ≥2nd operation). Patients were randomised to colonoscopy at 6 months with intensified treatment for endoscopic recurrence (“ active care” ) or no colonoscopy (“ standard care” ). All patients were colonoscoped at 18 months. HRQoL was assessed with a general (SF36) and diseasespecific (IBDQ) questionnaire pre‐operatively and at 6, 12 and 18 months. CRP, CDAI and faecal calprotectin (FC) were measured longitudinally. Results: 174 patients (median age 38, 55% female) were included. HRQoL was poor pre‐operatively: median SF36=40 (where maximum=100, Australian normal=70‐90) and IBDQ=120 (maximum=224, average score in Australian Crohn's disease patients=156). For all patients both SF36 and IBDQ improved significantly at 6 months to 78 and 178 respectively, and this was sustained at 12 months (81 and 183) and 18 months (80 and 182 respectively). Females had lower HRQoL than males post‐op at 6 (SF36 p=0.012; IBDQ p=0.007) and 12 months (SF36 p=0.001, IBDQ p=0.006). Smokers had poorer HRQoL compared to non‐smokers at both 12 and 18 months: SF36 at 12 month p=0.002, and IBDQ at 12 and 18 months (p=0.046, p=0.047 respectively). Persistent endoscopic remission, thiopurine or adalimumab therapy and treatment step up were not associated with changes in HRQoL. There was a significant inverse correlation between CDAI and both SF‐36 and IBDQ at 6, 12 and 18 months. HRQoL did not correlate with endoscopic remission, CRP or FC. Conclusion: Intestinal resection of all macroscopic Crohn's disease, with a focus on maintaining remission, is associated with significant and sustained improvement in general and disease‐specific HRQoL. The lower HRQoL in female patients and smokers may reflect partly their lower QoL in the healthy and IBD populations, but this requires further investigation. A higher clinical disease activity index, but not direct measures of active disease or type of drug therapy, is associated with a lower HRQoL, suggesting that symptoms reflect subjective personal factors and not active mucosal disease or drug effects.

%Z Gastroenterology

Journal article; Conference proceeding

%U https://cochrane.66557.net/central/doi/10.1002/central/CN-01061199/full

%0 Journal Article

%A Kennedy, N. A.

%A Ennis, H.

%A Gaya, D. R.

%A Mowat, C.

%A Arnott, IDR

%A Satsangi, J.

%D 2015

%T Interobserver agreement in assessment of Rutgeerts' score of endoscopic recurrence of ileal Crohn's disease: a substudy of the TOPPIC trial

%V 9

%P S231‐S232

%8 2015-01-01

%R 10.1093/ecco-jcc/jju027

%K *Crohn disease; *European; *colitis; *organization; Clinical trial (topic); Colonoscopy; Controlled study; Crohn disease; Endoscopist; Endoscopy; European; Human; Mercaptopurine; Patient; Placebo; Prevention; Procedures; Reliability; Risk; Statistical analysis; Surgery; clinical trial (topic); colitis; colonoscopy; controlled study; endoscopist; endoscopy; human; organization; patient; prevention; procedures; reliability; risk; statistical analysis; surgery

%X Background: Rutgeerts' score is widely used for the assessment of endoscopic recurrence following ileocaecal (IC) resection for Crohn's disease (CD). Higher scores have been shown to be associated with an increased risk of clinical recurrence. TOPPIC is a double‐blind randomised, placebo‐controlled trial of mercaptopurine for the prevention of post‐operative recurrence after IC resection for CD and includes a secondary endpoint of endoscopic recurrence. Few published data are available on interobserver agreement of Rutgeerts' score. This study aimed to assess the interobserver agreement of Rutgeerts' score on images from endoscopies carried out as part of the TOPPIC trial. (figure present) Methods: Five TOPPIC trial investigators were shown endoscopic images taken from 43 colonoscopies performed in Edinburgh as part of the TOPPIC trial. The investigators were blinded to the original report and were shown only the images with a description of the anatomical location from which each image was taken. Each investigator was independently asked to score each colonoscopy using the Rutgeerts' score and a custom‐designed application. Statistical analysis was performed using R and the psych package. The five scores for each colonoscopy were compared with each other and the score made by the original endoscopist. Shrout and Fleiss' intraclass correlation and pairwise weighted Cohen's kappa were calculated. Results: The original scores for the colonoscopies were spread across the possible scores, with 11 i0, 10 i1, 7 i2, 10 i3 and 5 i4. Intraclass correlation for single ratings (ICC3) was 0.82 (95% confdence interval 0.74‐0.88). The weighted Cohen's kappa when assessed for each possible pair of scorers ranged from 0.72 to 0.88. A graphical representation of the agreement between the original score and the rescores is shown in figure 1. When scores were stratified into endoscopic recurrence or not, as defined by a score of i2 or greater, all five scorers agreed with the original score in 34/43 (79%). There was no significant difference in this agreement between those procedures with an original score ≥i2 or <i2. Conclusions: Interobserver agreement for Rutgeerts' score of endo‐scopic recurrence was generally good in this cohort of patients. Despite the limitation of using still images, agreement with the score of the original endoscopistwas also good. However, there was some variation in assessment, even when assessing the presence/absence of endoscopic recurrence. These findings are important when considering the reliability of outcome data in multicentre clinical trials.

%Z Journal of Crohn's & colitis

Journal article; Conference proceeding

%U https://cochrane.66557.net/central/doi/10.1002/central/CN-01130641/full

%0 Journal Article

%A Kennedy, N. A.

%A Ennis, H.

%A Gaya, D. R.

%A Mowat, C.

%A Arnott, I. D.

%A Satsangi, J.

%D 2015

%T Interobserver agreement in assessment of rutgeerts' score of endoscopic recurrence of ileal crohn's disease

%V 148

%N 4

%P S431

%8 2015-01-01

%K *Crohn disease; *gastrointestinal disease; Clinical trial (topic); Colonoscopy; Confidence interval; Controlled study; Crohn disease; Endoscopist; Endoscopy; Gastroenterology; Human; Mercaptopurine; Patient; Placebo; Prevention; Procedures; Reliability; Risk; Statistical analysis; Surgery; clinical trial (topic); colonoscopy; confidence interval; controlled study; endoscopist; endoscopy; gastroenterology; gastrointestinal disease; human; patient; prevention; procedures; reliability; risk; statistical analysis; surgery

%X Introduction Rutgeerts' score is widely used for the assessment of endoscopic recurrence following ileocaecal (IC) resection for Crohn's disease (CD). Higher scores have been shown to be associated with an increased risk of clinical recurrence (Rutgeerts et al., Gastroenterology 1990 Oct;99(4):956‐63). TOPPIC is a double‐blind randomised, placebo‐controlled trial of mercaptopurine for the prevention of post‐operative recurrence after IC resection for CD and includes a secondary endpoint of endoscopic recurrence (MRCG060329; EudraCT Number: 2006‐005800‐15). Few published data are available on interobserver agreement of Rutgeerts' score. This study aimed to assess the interobserver agreement of Rutgeerts' score on images from endoscopies carried out as part of the TOPPIC trial. Methods Five TOPPIC trial investigators were shown endoscopic images taken from 43 colonoscopies performed in Edinburgh as part of the TOPPIC trial. The investigators were blinded to the original report and were shown only the images with a description of the anatomical location from which each image was taken. Each investigator was independently asked to score each colonoscopy using the Rutgeerts' score and a custom‐designed application. Statistical analysis was performed using R and the psych package. The five scores for each colonoscopy were compared with each other and the score made by the original endoscopist. Shrout and Fleiss' intraclass correlation and pairwise weighted Cohen's kappa were calculated. Results The original scores for the colonoscopies were spread across the possible scores, with 11 i0, 10 i1, 7 i2, 10 i3 and 5 i4. Intraclass correlation for single ratings (ICC3) was 0.82 (95% confidence interval 0.74‐0.88). The weighted Cohen's kappa when assessed for each possible pair of scorers ranged from 0.72 to 0.88. A graphical representation of the agreement between the original score and the rescores is shown in figure 1. When scores were stratified into endoscopic recurrence or not, as defined by a score of i2 or greater, all five scorers agreed with the original score in 34/43 (79%). There was no significant difference in this agreement between those procedures with an original score ≥i2 or Conclusions Interobserver agreement for Rutgeerts' score of endoscopic recurrence was generally good in this cohort of patients. Despite the limitation of using still images, agreement with the score of the original endoscopist was also good. However, there was some variation in assessment, even when assessing the presence/absence of endoscopic recurrence. These findings are important when considering the reliability of outcome data in multicentre clinical trials such as TOPPIC, PREVENT and POCER. (Figure Presented).

%Z Gastroenterology

Journal article; Conference proceeding

%U https://cochrane.66557.net/central/doi/10.1002/central/CN-01089120/full

%0 Journal Article

%A NCT

%D 2023

%T Injection of Freshly Collected Autologous Adipose Tissue Additional to Bascom's Cleft Lift Surgery

%8 2023-01-01

%X The overall aim of the project is to investigate whether the addition of autologous fatty cell transplantation in the surgical treatment of pilonidal disease (PD) reduces the incidence of patients with prolonged postoperative healing. In a double‐blinded randomized controlled clinical trial, the investigators will investigate the effect of injections of freshly harvested autologous fatty cells in addition to Bascom's cleft lift operation versus Bascom's cleft lift operation alone. The investigators hypothesize that addition of autologous fatty cell injection to Bascom's cleft lift operation for pilonidal disease will reduce the proportion of patients with poor postoperative healing compared to patients in the control group who are treated with Bascom's cleft lift operation alone. The primary endpoint of this study is healing of the pilonidal lesion (max 1 defect ≤5mm, no undermining) after 4 weeks and 12 weeks. Further, symptom development, postoperative pain, complication rate, time to healing and recurrence rates are also explored. BRIEF LITERATURE REVIEW: Pilonidal disease (PD) is a widespread disorder that occurs in the crena ani. A paradigm shift in our perception of the pathology causing this disorder has been coming for several decades and the treatment is slowly conforming to this. The estimated incidence is 26‐48 per 100,000, although globally there is great geographical variation (1). Men present with treatment‐requiring pilonidal disease more than twice as often as women, and population studies of young students have found up to a 10:1 male/female ratio, including asymptomatic pilonidal cysts(2). In the investigators own material, the proportion of women are 17% of patients with complicated pilonidal disease (62/363). The disorder most often affects younger patients with an average age at first manifestation of 21 years for men and 19 for women (1). Acute manifestations are infected cysts and results in the formation of pilonidal abscesses. The treatment of these is uncontroversial and is based on lateral incision and drainage with secondary open healing. Chronic pilonidal disease manifests itself in the formation of more or less widespread subcutaneous branching systems containing hair which is "sucked" into the system by negative pressure that occurs when changing position, especially from sitting to standing. The condition is characterized by pain and foul‐smelling secretions and bleeding. The treatment of the chronic manifestations, as opposed to the acute abscesses, has been controversial and over the last 1‐2 decades has gradually moved more towards lateralization techniques, the gap between the buttocks is (partially) levelled surgically and the suture line is placed outside the midline of the crena ani (3,4). Bascom's cleft lift operation is one of several lateralization techniques that have shown promising results over the past decades (5‐10). Nevertheless, in the hands of the investogators poor or delayed healing is found in up to a third of these otherwise young and healthy patients, which seems to be in line with the international literature (11). Many patients thus experience long outpatient courses postoperatively with several additional treatments such as regular surgical wound revision, treatment with various forms of negative pressure bandaging, steroid cream regimens or silvernitrate dressings. Platelet‐rich plasma therapy (BPT) is used with good effect as an addition to the surgical treatment of pilonidal disease, as BPT promotes angiogenesis, cell regeneration and thus promotes healing. A review finds a reduction in healing time of 36% in BPT‐treated patients compared to the control group(12,13). Moreover, treatments with allogeneic adipose‐derived stem cells are used in other healing‐challenged areas, such as mb. Crohn's fistulas and burns (14‐16). However, both treatments are both time‐consuming and expensive, and the investigators believe that autologous fat cell transplantation is a faster and more affordable alternative, which a study in Mb. Crohn's patients with complicated peri‐anal fistulas also support: Here, in a series of 21 patients healing was found in 57% (clinical and MR‐verified) ‐ a very satisfactory result in this context (17). The cell composition in the stromal vascular fraction (SVF) of the harvested adipose tissue has been found to contain 10%‐50% live stromal (mesenchymal) stem cells and 7%‐30% endothelial cells (mature and stem cells) depending on where the fat is harvested and which method is used (18‐20). This cell composition supports the healing potential in the treatment with autologous fatty cell transplantation. In the department of the investigators, a series of operations with autologous fatty cell injections for non‐healing pilonidal wounds has been carried out with good results. Thus, in these highly selected patients, the investigators have a healing rate of 83% (69‐96%) over a median time of 159 days. Two patients had recurrence (6.7%) (21). The method is minimally invasive and is combined in this study with Bascom's cleft lift (BCL) operation. The results seem promising with minimal risks and it could be an important supplement to the basic treatment of these patients. 3) METHOD STUDYDESIGN: The study will be conducted as a double‐blinded randomized controlled clinical trial that will compare healing after BLC surgery with injections of freshly harvested autologous adipose tissue versus BLC surgery alone. The trial is being conducted as a multicenter study, where patients are included at two centers in Denmark. The two centers are located at Randers Regional Hospital and Slagelse Hospital. SURGICAL PROCEDURE: Included patients will undergo BLC surgery according to standardized principles. Prior to surgery, the randomization is carried out using allocation tables in REDCap, where an equal distribution of patients in the intervention group and the control group is also ensured in each center. Intervention group: Patients in the intervention group will undergo liposuction to harvest autologous fatty cells. The patient is placed in prone position. For the liposuction, a small incision is made bilaterally in the flanks. 2‐300 ml of Ringer's acetate with 1 mg of added adrenaline is introduced with an infusion cannula fan‐shaped into the subcutaneous adipose tissue on each side of the lumbar region. Adipose tissue is harvested with an equal distribution on each side of the region for a total of approx. 80 ml. 20 ml Bupivacaine 5 mg/ml is distributed on each side of the lower back after harvesting the fatty cells. The incisions are then closed with Steri‐Strips and a compressive dressing is applied to reduce the risk of hematoma. The adipose tissue is then prepared: The harvested adipose tissue is distributed equally in 10 ml syringes and centrifuged in bulks of four at 1000 rpm. minute for 3 minutes. The liquid fraction is discarded. Using a three‐way tap system, the adipose tissue is homogenized/microfragmented between two 10 ml syringes by moving the contents back and forth at least 20 times. The adipose tissue is then distributed in 2 ml syringes for injection. A total of between 25 and 55 ml of usable adipose tissue will be harvested. Bascom cleft lift operation is performed as described in appendix 7. Before the cleft lift incision is sutured, the fatty tissue will be systematically injected with a sharp 1.2 mm needle and cover all the wound surfaces with 0.2‐0.5 ml at a time. The procedure was published as a video vignette in 2019(22). Results from the first seven patients (2020) and the first 30 patients (2022) have been published (21,23). Patients in the control group: Control patients are given a sham, where a small incision is also made bilaterally in the lumbar region and 20 ml Bupivacaine 5 mg/ml is distributed on each side of the lower back. The incisions are then closed with Steri‐Strips and compressive dressings are applied in the same way as the intervention group. COOPERATION PARTNERS: Surgeries will be performed by specialists in abdominal surgery with a sub‐specialty within this type of surgery in two centres: Surgical Department at Randers Regionshospital and Slagelse Hospital. I. Faurschou at Randers Regionshospital, Region Midt, has the overall responsibility for the project with Supervisor Susanne Haas as the legally responsible person. The local manager at Slagelse Hospital is P. Maine. Those responsible at each site are responsible for inclusion and informing the patients, guided and supported by I. Faurschou. POST‐OPERATIV FOLLOW‐UP: The patients will have follow ups at 4 and 12 weeks after the operation, where the healing will be clinically assessed based on endpoints by project manager I. Faurschou, blinded to the randomisation. The control takes place by telephone consultation together with answering the symptom questionnaire and pain diary sent out automatically from REDCap® as well as weekly photo documentation of healing through Pleje.net® (Dansk Telemedicin A/S), with the option of immediate physical consultation within a week in case of lack of healing. Photos will be taken using the Pleje.net app weekly in relation to hair removal by a nurse/person who handles hair removal. Patients are given a guide to Pleje.net. On Pleje.net, a user group has been set up for each site, so only project managers can see all the patients. If there is no healing after 12 weeks, the patient will be offered further treatment in line with our normal treatment practice. 4) STATISTICAL CONSIDERATIONS: Sample size: In a recent study, the investigators found somewhat surprisingly that only 12% of PS patients operated with BCL surgery were fully healed after 14 days (≤ 1 defect, ≤ 5 mm, no undermining). After 12 weeks, this proportion was 72%. The investigaros expect 50% of patients to have healed after 4 weeks. Using fatty cells in non‐healing PS wounds, the investigators have a healing rate of 83% (69‐96%) over a median time of 159 days. In combination with fatty cells, it is expected that 75% of patients have healed by 4 weeks. With a significance level of 5% (α) and a power of 80% (β), 66 patients will be included in each group (Fleiss with continuity correction). Assuming a dropout of 4 patients in each group, a total of 140 patients will be included. Interim analysis: Interim analysis: Halfway through the inclusion, an interim analysis will be carried out. A Data Monitoring Committee (DMC) is set up, which will make a recommendation to the project managers on whether the study should continue as planned, be interrupted due to unintended side effects as a result of AFT, interrupted because the effect of AFT has been unequivocally established; or is interrupted because it will not be possible to see a difference if the study is continued. 5) PATIENTS: The patients are referred to the investigators outpatient clinic and informed about the project if BCL surgery is needed (recurrent or complex disease). After reflection time, patients are included on the day of surgery, where they are randomised. After the operation, they will be followed in the outpatient 4 weeks and 12 weeks postoperatively. If there is no healing after 12 weeks, the patient will be offered further treatment in line with our normal treatment practice.

%Z https://clinicaltrials.gov/show/NCT05830734

Trial registry record

%U https://cochrane.66557.net/central/doi/10.1002/central/CN-02559698/full

%0 Journal Article

%A Regueiro, M.

%A Feagan, B. G.

%A Zou, B.

%A Johanns, J.

%A Blank, M. A.

%A Chevrier, M.

%A Plevy, S.

%A Popp, J.

%A Cornillie, F. J.

%A Lukas, M.

%A Et, Al.

%D 2016

%T Infliximab Reduces Endoscopic, but Not Clinical, Recurrence of Crohn's Disease After Ileocolonic Resection

%V 150

%N 7

%P 1568‐1578

%8 2016-01-01

%R 10.1053/j.gastro.2016.02.072

%K *Crohn disease/dt [Drug Therapy]; *Crohn disease/pc [Prevention]; *Crohn disease/su [Surgery]; *colon resection; *ileocolonic resection; *infliximab/ae [Adverse Drug Reaction]; *infliximab/cr [Drug Concentration]; *infliximab/ct [Clinical Trial]; *infliximab/dt [Drug Therapy]; *recurrent disease/pc [Prevention]; Adult; Aphthous stomatitis; Article; Colectomy [*adverse effects]; Colon [pathology, surgery]; Colonoscopy; Controlled study; Crohn Disease Activity Index; Crohn Disease [*drug therapy, pathology, surgery]; Digestive system disease assessment; Double blind procedure; Double‐Blind Method; Drug blood level; Drug efficacy; Drug safety; Female; Gastrointestinal Agents [*administration & dosage]; Gastrointestinal endoscopy; Gastrointestinal symptom/si [Side Effect]; Hospitalization; Human; Humans; Ileum [pathology, surgery]; Immunogenicity; Immunosuppressive treatment; Infection rate/si [Side Effect]; Infestation/si [Side Effect]; Infliximab [*administration & dosage]; Infusion related reaction/si [Side Effect]; Jejunum ulcer; Major clinical study; Male; Middle Aged; Multicenter study; Phase 3 clinical trial; Placebo; Postoperative Period; Postoperative complication/co [Complication]; Priority journal; Randomized controlled trial; Recurrence; Recurrence risk; Rutgeerts score; Secondary Prevention [*methods]; Treatment Outcome; Treatment failure; Tumor necrosis factor inhibitor

%X Background & Aims Most patients with Crohn's disease (CD) eventually require an intestinal resection. However, CD frequently recurs after resection. We performed a randomized trial to compare the ability of infliximab vs placebo to prevent CD recurrence. Methods We evaluated the efficacy of infliximab in preventing postoperative recurrence of CD in 297 patients at 104 sites worldwide from November 2010 through May 2012. All study patients had undergone ileocolonic resection within 45 days before randomization. Patients were randomly assigned (1:1) to groups given infliximab (5 mg/kg) or placebo every 8 weeks for 200 weeks. The primary end point was clinical recurrence, defined as a composite outcome consisting of a CD Activity Index score >200 and a >70‐point increase from baseline, and endoscopic recurrence (Rutgeerts score >i2, determined by a central reader) or development of a new or re‐draining fistula or abscess, before or at week 76. Endoscopic recurrence was a major secondary end point. Results A smaller proportion of patients in the infliximab group had a clinical recurrence before or at week 76 compared with the placebo group, but this difference was not statistically significant (12.9% vs 20.0%; absolute risk reduction [ARR] with infliximab, 7.1%; 95% confidence interval: ‐1.3% to 15.5%; P =.097). A significantly smaller proportion of patients in the infliximab group had endoscopic recurrence compared with the placebo group (30.6% vs 60.0%; ARR with infliximab, 29.4%; 95% confidence interval: 18.6% to 40.2%; P <.001). Additionally, a significantly smaller proportion of patients in the infliximab group had endoscopic recurrence based only on Rutgeerts scores >i2 (22.4% vs 51.3%; ARR with infliximab, 28.9%; 95% confidence interval: 18.4% to 39.4%; P <.001). Patients previously treated with anti‐tumor necrosis factor agents or those with more than 1 resection were at greater risk for clinical recurrence. The safety profile of infliximab was similar to that from previous reports. Conclusions Infliximab is not superior to placebo in preventing clinical recurrence after CD‐related resection. However, infliximab does reduce endoscopic recurrence. ClinicalTrials.gov ID NCT01190839.

%Z Gastroenterology

Journal article

%U https://cochrane.66557.net/central/doi/10.1002/central/CN-01165611/full

%0 Journal Article

%A Cottone, M.

%A Mocciaro, F.

%A Orlando, A.

%D 2009

%T Infliximab prevents Crohn's disease recurrence after ileal resection

%V 3

%N 3

%P 231‐234

%8 2009-01-01

%R 10.1586/egh.09.13

%K *Crohn disease /drug therapy /drug therapy /prevention /surgery; *ileum resection; *recurrent disease /prevention; Article; Clinical article; Clinical protocol; Clinical trial; Colonoscopy; Controlled clinical trial; Controlled study; Disease severity; Drug withdrawal; Endoscopy; Human; Neutrophil chemotaxis; Randomized controlled trial; Relapse; Remission; Unspecified side effect /side effect

%X In the evaluation of drugs for the prevention of postoperative recurrence in Crohn's disease (CD), severe endoscopic recurrence (Rutgeerts score: i‐2) is the primary outcome because of its high predictive value of clinical relapse. At 1 year, severe endoscopic recurrence rates range from 30 to 79% in untreated patients. Many drugs have been evaluated in controlled clinical trials for the prevention of postoperative recurrence of CD, such as mesalamine, azathioprine/ 6‐mercaptopurine, metronidazole/ornidazole, probiotics, budesonide and IL‐10. The results of these studies are conflicting. Amongst these drugs, mesalamine and metronidazole, alone or in combination with azathioprine, have been shown to be effective in the risk reduction of postoperative recurrence of CD, with nonhomogeneous results. A recent small trial reports the role of infliximab in the prevention of severe endoscopic recurrence; the drug was effective in obtaining an absolute risk reduction of 75.5%. Considering the cost and safety problems of biological therapy, this result should be confirmed in a large trial before infliximab can be recommended for the prevention of endoscopic recurrence in CD. © 2009 Expert Reviews Ltd.

%Z Expert review of gastroenterology & hepatology

Journal article

%U https://cochrane.66557.net/central/doi/10.1002/central/CN-01731125/full

%0 Journal Article

%A Panaccione, R.

%D 2001

%T Infliximab for the treatment of Crohn's disease: review and indications for clinical use in Canada

%V 15

%N 6

%P 371‐375

%8 2001-01-01

%R 10.1155/2001/490921

%K *Crohn disease /drug therapy; Abdominal cramp; Abdominal pain; Antibodies, Monoclonal [*therapeutic use]; Arthralgia /side effect; Arthritis /drug therapy; Article; Behcet disease /drug therapy; Body weight loss; Canada; Clinical trial; Complication /complication /surgery; Controlled clinical trial; Controlled study; Crohn Disease [*drug therapy]; Daily life activity; Delayed hypersensitivity /side effect; Diarrhea; Digestive system inflammation /drug therapy; Dose response; Drug indication; Drug mechanism; Drug safety; Drug tolerability; Drug use; Dyspnea /side effect; Enterocutaneous fistula /drug therapy; Fever /side effect; Flushing; Gastrointestinal Agents [*therapeutic use]; Headache /side effect; Human; Humans; Infection /side effect; Infliximab; Lymphoma /side effect; Multicenter study; Nausea /side effect; Phase 2 clinical trial; Priority journal; Pyoderma gangrenosum /drug therapy; Quality of life; Randomized controlled trial; Recurrent disease /complication; Relapse; Remission; Scoring system; Side effect /side effect; Skin manifestation /side effect; Spondyloarthropathy /drug therapy; Symptomatology; Thorax pain /side effect; Treatment Outcome; Ulcerative colitis /drug therapy; Urticaria /side effect; Vomiting; Work environment

%X Crohn's disease is a chronic inflammatory disorder of the gastrointestinal tract. It may affect any portion of the gastrointestinal tract from the mouth to the anus. Symptoms typically include cramping abdominal pain, diarrhea (which may be bloody) and nausea. As the severity of the illness worsens, patients may experience constant abdominal pain, vomiting, weight loss and fever. From the perspective of the patient, disease symptoms significantly impair quality of life, and interfere with their work environment and activities of daily living. Unfortunately, there is no cure for Crohn's disease. Patients experience a chronic, relapsing course characterized by recurrent flares of their disease. Conventional medical treatment of Crohn's disease includes the use of non‐specific anti‐inflammatory drugs (5‐aminosalicylic acid agents, prednisone, budesonide), immunosuppressives (6‐mercaptopurine, azathioprine, methotrexate) and antibiotics. A variable onset of action, incomplete response rates and a significant risk of adverse effects characterize current therapies. Although surgery is frequently used to treat complications or medically refractory disease, postoperative recurrence is a common problem. Infliximab, a murine chimeric monoclonal antibody directed toward tumour necrosis factor‐alpha, is a highly effective treatment of active Crohn's disease. In randomized, placebo‐controlled clinical trials, 33% of patients treated with infliximab 5 mg/kg achieved remission (Crohn's Disease Activity Index score less than 150), compared with only 4% of those receiving placebo (P<0.001). Additionally, infliximab is the only drug therapy shown to be effective for the treatment of fistulizing Crohn's disease. In studies done to date, infliximab appears to be well tolerated and has a favourable side effect profile.

%Z Journal canadien de gastroenterologie [Canadian journal of gastroenterology]

Journal article

%U https://cochrane.66557.net/central/doi/10.1002/central/CN-01715584/full

%0 Journal Article

%A Feagan, B. G.

%A Enns, R.

%A Fedorak, R. N.

%A Panaccione, R.

%A Pare, P.

%A Steinhart, A. H.

%A Wild, G.

%D 2001

%T Infliximab for the treatment of Crohn's disease: efficacy, safety and pharmacoeconomics

%V 8

%N 4

%P 188‐198

%8 2001-01-01

%K *Crohn disease /drug therapy /surgery; Adult; Antibodies, Monoclonal [adverse effects, economics, *therapeutic use]; Anus fistula /complication /drug therapy /prevention; Article; Clinical trial; Controlled clinical trial; Controlled study; Cost effectiveness analysis; Cost of Illness; Cost utility analysis; Cost‐Benefit Analysis; Crohn Disease [*drug therapy]; Daily life activity; Drug cost; Drug efficacy; Drug safety; Drug tolerability; Gastrointestinal Agents [adverse effects, economics, *therapeutic use]; Health care system; Health economics; Human; Humans; Infliximab; Major clinical study; Quality of Life; Quality of life; Randomized controlled trial; Recurrent disease

%X Crohn's disease is a chronic inflammatory disorder of the gastrointestinal tract. From the perspective of the patient, symptoms of the disease significantly impair quality of life and interfere with activities of daily living. Conventional medical treatment of Crohn's disease includes the use of nonspecific anti‐inflammatory drugs, immunosuppressives and antibiotics. These therapies are characterized by a delayed onset of action, incomplete response rates and a substantial risk of adverse effects. Although surgery is frequently used to treat complications, postoperative recurrence is an important problem. Infliximab, a chimeric monoclonal antibody directed toward tumour necrosis factor alpha, is highly effective for the treatment of active Crohn's disease. In randomized, placebo‐controlled clinical trials, 82% of patients who received 5 mg/kg of infliximab had a clinically significant response, compared with 17% of those given placebo (P<0.001). Moreover, infliximab is the only medical therapy that has been shown to be effective for the treatment of fistulizing Crohn's disease. Infusion reactions are the most common adverse effect. Whether treatment with infliximab is associated with an increased risk of neoplasia, infection or autoimmune disease is unknown. Therefore, further long term safety studies are required. Despite the relatively high cost of drug acquisition, preliminary pharmacoeconomic analysis indicates that infliximab is cost effective compared with existing treatments. Infliximab is recommended for the treatment of active Crohn's disease refractory to conventional drugs, and is the treatment of choice for fistulizing Crohn's disease.

%Z Journal canadien de pharmacologie clinique [Canadian journal of clinical pharmacology]

Journal article

%U https://cochrane.66557.net/central/doi/10.1002/central/CN-01720395/full

%0 Journal Article

%A Regueiro, M.

%A Feagan, B. G.

%A Zou, B.

%A Johanns, J.

%A Blank, M.

%A Chevrier, M.

%A Plevy, S. E.

%A Popp, J.

%A Cornillie, F.

%A Lukas, M.

%A Et, Al.

%D 2015

%T Infliximab for prevention of recurrence of post-surgical crohn's disease following ileocolonic resection: a randomized, placebo-controlled study

%V 148

%N 4

%P S141

%8 2015-01-01

%K *Crohn disease; *controlled study; *gastrointestinal disease; *human; *infliximab; *placebo; *prevention; *surgery; Abscess; Anastomosis; Crohn Disease Activity Index; Crohn disease; Death; Diarrhea; Fistula; Infection; Infection rate; Infusion related reaction; Intestine resection; Miliary tuberculosis; Non melanoma skin cancer; Patient; Reading; Risk; Safety; abscess; anastomosis; controlled study; death; diarrhea; fistula; gastrointestinal disease; human; infection; infection rate; infusion related reaction; intestine resection; miliary tuberculosis; non melanoma skin cancer; patient; prevention; reading; risk; safety; surgery

%X Objectives: To compare the efficacy of infliximab (IFX) with placebo (PBO) for the prevention of clinical recurrence of Crohn's disease (CD) in patients (pts) following intestinal resection with ileocolonic anastomosis. Method: PREVENT, a global, randomized, double‐blind, PBOcontrolled, parallel‐group study evaluated the therapeutic benefit and safety of IFX in pts with an increased risk of recurrence of active CD after ileocolonic resection. Eligible pts with Crohn's Disease Activity Index (CDAI) < 200 who had undergone resection < 45 days prior were randomized equally to receive IFX 5 mg/kg or PBO at wk 0 then every 8wks thereafter through wk200. The primary endpoint of clinical recurrence prior to or at wk76 was a composite endpoint, defined by a > 70 point increase from baseline in CDAI and a CDAI > 200, endoscopic recurrence (Rutgeerts score of >i2) and negative stool C. difficile (if symptoms were predominantly diarrhea). Development of new/re‐draining fistula or abscess also constituted clinical recurrence. The major secondary endpoint was endoscopic recurrence as scored by an expert central reader. Efficacy analyses included all randomized pts. Endpoints were sequentially tested. Results: 297 pts (IFX, N=147; PBO, N=150) were randomized. Demographics were balanced between groups. The proportion of pts with clinical recurrence prior to or at wk76 was lower for IFX than PBO (12.9% vs 20.0%), although the difference was not statistically significant (p=0.097). A smaller proportion of IFX pts than PBO pts had endoscopic recurrence prior to or at wk76 (30.6% vs 60%; nominal p<0.001). Consistent with wk76, a lower proportion of IFX than PBO pts had clinical recurrence prior to or at wk104 (17.7% vs 25.3%; nominal p=0.098). A decision was made to stop the study. Through wk104, the safety profile of IFX‐treated pts was consistent with prescribing information. There were more infusion‐related reactions leading to discontinuation of IFX vs PBO. Infection rates, including serious infections, were similar between groups; miliary tuberculosis occurred in one IFX‐treated pt. Two malignancies (excluding non‐melanoma skin cancer) and one death occurred in PBO‐treated pts. Conclusions: Trends favoring IFX were observed for fewer clinical (wk76, wk104) and endoscopic (wk76) recurrences in PREVENT (similar to that reported previously at 1 year1), but the primary endpoint of clinical recurrence was not met. No new safety issues were identified.

%Z Gastroenterology

Journal article; Conference proceeding

%U https://cochrane.66557.net/central/doi/10.1002/central/CN-01089160/full

%0 Journal Article

%A Ankersen, D. V.

%A Weimers, P.

%A Marker, D.

%A Bennedsen, M.

%A Saboori, S.

%A Paridaens, K.

%A Burisch, J.

%A Munkholm, P.

%D 2019

%T Individualized home-monitoring of disease activity in adult patients with inflammatory bowel disease can be recommended in clinical practice: a randomized-clinical trial

%V 25

%N 40

%P 6158‐6171

%8 2019-01-01

%R 10.3748/wjg.v25.i40.6158

%K *clinical practice; *home monitoring; *inflammatory bowel disease; Adult; Article; Biological Products [therapeutic use]; Colitis, Ulcerative [*diagnosis, drug therapy]; Controlled study; Crohn Disease [*diagnosis, drug therapy]; Disease Progression; Disease activity score; Fatigue; Feces; Feces [chemistry]; Female; Glucocorticoids [therapeutic use]; Human; Humans; Immunologic Factors [therapeutic use]; Internet‐Based Intervention; Leukocyte L1 Antigen Complex [analysis]; Major clinical study; Male; Mass Screening [instrumentation, *methods]; Mass screening; Medication Adherence; Middle Aged; Program Evaluation; Quality of Life; Quality of life; Randomized controlled trial; Recurrence; Recurrence risk; Remission; Severity of Illness Index; Telemedicine [instrumentation, *methods]

%X BACKGROUND: The optimal way to home‐monitor patients with inflammatory bowel disease (IBD) for disease progression or relapse remains to be found. AIM: To determine whether an electronic health (eHealth) screening procedure for disease activity in IBD should be implemented in clinical practice, scheduled every third month (3M) or according to patient own decision, on demand (OD). METHODS: Adult IBD patients were consecutively randomized to 1‐year open‐label eHealth interventions (3M vs OD). Both intervention arms were screening for disease activity, quality of life and fatigue and were measuring medical compliance with the constant care web‐application according to the screening interventions OD or 3M. Disease activity was assessed using home measured fecal calprotectin (FC) and a disease activity score. RESULTS: In total, 102 patients were randomized (n = 52/50 3M/OD) at baseline, and 88 patients completed the 1‐year study (n = 43 3M; n = 45 OD). No difference in the two screening procedures could be found regarding medical compliance (P = 0.58), fatigue (P = 0.86), quality of life (P = 0.17), mean time spent in remission (P > 0.32), overall FC relapse rates (P = 0.49), FC disease courses (P = 0.61), FC time to a severe relapse (P = 0.69) and remission (P = 0.88) during 1 year. Median (interquartile range) numbers of FC home‐monitoring test‐kits used per patient were significantly different, 3M: 6.0 (5.0‐8.0) and OD: 4.0 (2.0‐9.0), P = 0.04. CONCLUSION: The two eHealth screening procedures are equally good in capturing a relapse and bringing about remission. However, the OD group used fewer FC home testkits per patient. Individualized screening procedures can be recommended for adult IBD patients in clinical web‐practice.

%Z World journal of gastroenterology

Journal article

%U https://cochrane.66557.net/central/doi/10.1002/central/CN-02005381/full

%0 Journal Article

%A van Linschoten, RCA

%A Jansen, F. M.

%A Pauwels, RWM

%A Smits, LJT

%A Atsma, F.

%A Kievit, W.

%A de Jong, D. J.

%A de Vries, A. C.

%A Boekema, P. J.

%A West, R. L.

%A Et, Al.

%D 2023

%T Increased versus conventional adalimumab dose interval for patients with Crohn's disease in stable remission (LADI): a pragmatic, open-label, non-inferiority, randomised controlled trial

%V 8

%N 4

%P 343‐355

%8 2023-01-01

%R 10.1016/S2468-1253(22)00434-4

%K *Harvey Bradshaw Index; *remission; Adalimumab [therapeutic use]; Adolescent; Adult; Article; Attributable risk; Clinical assessment; Clinical outcome; Clinical trial; Controlled study; Crohn Disease [drug therapy]; Cumulative incidence; C‐Reactive Protein; Drug therapy; Feces; Female; General hospital; Health care personnel; Human; Humans; Intestine obstruction; Major clinical study; Male; Medical research; Methotrexate [therapeutic use]; Multicenter study; Netherlands; Outcome assessment; Patient preference; Randomization; Randomized controlled trial; Recurrent disease; University hospital

%X BACKGROUND: Despite its effectiveness in treating Crohn's disease, adalimumab is associated with an increased risk of infections and high health‐care costs. We aimed to assess clinical outcomes of increased adalimumab dose intervals versus conventional dosing in patients with Crohn's disease in stable remission. METHODS: The LADI study was a pragmatic, open‐label, multicentre, non‐inferiority, parallel, randomised controlled trial, done in six academic hospitals and 14 general hospitals in the Netherlands. Adults (aged ≥18 years) diagnosed with luminal Crohn's disease (with or without concomitant perianal disease) were eligible when in steroid‐free clinical and biochemical remission (defined as Harvey‐Bradshaw Index [HBI] score <5, faecal calprotectin <150 μg/g, and C‐reactive protein <10 mg/L) for at least 9 months on a stable dose of 40 mg subcutaneous adalimumab every 2 weeks. Patients were randomly assigned (2:1) to the intervention group or control group by the coordinating investigator using a secure web‐based system with variable block randomisation (block sizes of 6, 9, and 12). Randomisation was stratified on concomitant use of thiopurines and methotrexate. Patients and health‐care providers were not masked to group assignment. Patients allocated to the intervention group increased adalimumab dose intervals to 40 mg every 3 weeks at baseline and further to every 4 weeks if they remained in clinical and biochemical remission at week 24. Patients in the control group continued their 2‐weekly dose interval. The primary outcome was the cumulative incidence of persistent flares at week 48 defined as the presence of at least two of the following criteria: HBI score of 5 or more, C‐reactive protein 10 mg/L or more, and faecal calprotectin more than 250 μg/g for more than 8 weeks and a concurrent decrease in the adalimumab dose interval or start of escape medication. The non‐inferiority margin was 15% on a risk difference scale. All analyses were done in the intention‐to‐treat and per‐protocol populations. This trial was registered at ClinicalTrials.gov, NCT03172377, and is not recruiting. FINDINGS: Between May 3, 2017, and July 6, 2020, 174 patients were randomly assigned to the intervention group (n=113) or the control group (n=61). Four patients from the intervention group and one patient from the control group were excluded from the analysis for not meeting inclusion criteria. 85 (50%) of 169 participants were female and 84 (50%) were male. At week 48, the cumulative incidence of persistent flares in the intervention group (three [3%] of 109) was non‐inferior compared with the control group (zero; pooled adjusted risk difference 1·86% [90% CI ‐0·35 to 4·07). Seven serious adverse events occurred, all in the intervention group, of which two (both patients with intestinal obstruction) were possibly related to the intervention. Per 100 person‐years, 168·35 total adverse events, 59·99 infection‐related adverse events, and 42·57 gastrointestinal adverse events occurred in the intervention group versus 134·67, 75·03, and 5·77 in the control group, respectively. INTERPRETATION: The individual benefit of increasing adalimumab dose intervals versus the risk of disease recurrence is a trade‐off that should take patient preferences regarding medication and the risk of a flare into account. FUNDING: Netherlands Organisation for Health Research and Development.

%Z The lancet. Gastroenterology & hepatology

Journal article

%U https://cochrane.66557.net/central/doi/10.1002/central/CN-02522540/full

%0 Journal Article

%A Yoshimura, N.

%A Kawaguchi, T.

%A Sako, M.

%A Saniabadi, A.

%A Takazoe, M.

%D 2014

%T In patients with crohn's disease, concomitant enteral nutrition reduces the loss of response to adalimumab maintenance therapy

%V 146

%N 5

%P S‐382

%8 2014-01-01

%R 10.1016/S0016-5085(14)61374-3

%K *Crohn disease; *adalimumab; *enteric feeding; *gastrointestinal disease; *human; *maintenance therapy; *patient; Control group; Crohn disease; Demography; Japan; Monoclonal antibody; Multivariate analysis; Remission; Risk factor; Survival; Therapy; Tumor necrosis factor; control group; demography; enteric feeding; gastrointestinal disease; human; maintenance therapy; multivariate analysis; patient; remission; risk factor; survival; therapy

%X BACKGROUND: In recent years, Adalimumab (ADA), an anti‐tumor necrosis factor (TNF) monoclonal antibody has been applied to induce and maintain clinical remission in patients with Crohn's disease (CD). However, loss of response to ADA during maintenance therapy in the clinical management of CD is a major therapeutic issue. Further, in Japan, enteral nutrition (EN) has been associated with efficacy for inducing and maintaining remission in patients with CD, but hitherto no study has investigated the efficacy of concomitant EN for sustaining response to ADA in patients with quiescent CD receiving ADA maintenance therapy. This study was to assess the efficacy of concomitant EN in patients with CD during ADA induction and maintenance therapy. METHODS: We retrospectively reviewed a cohort of 76 consecutive CD patients with moderate to severe disease, CD activity index (CDAI) ≥150 who had received subcutaneous ADA, 160mg, and 80mg at weeks 0, 2, respectively as induction therapy. These 76 patients were randomly divided into an EN group (≥300kcal/ day) who received concomitant EN and a non‐EN group (control). At entry, there was no significant difference between the 2 groups with respect to demography. At week 4, clinical remission rates, defined as CDAI<150 were compared between the 2 groups. Patients who had successful ADA induction therapy received ADA maintenance therapy at 40mg, every other week. Patients in the 2 groups were followed for 52 weeks and loss of response to ADA was defined as CDAI≥150; an increase of ≥70 points in the CDAI. The cumulative remission rates were compared by the Kaplan‐Meier survival analysis, while the risk factors for recurrence were evaluated by multivariate analysis. RESULTS: Forty‐two of the 76 patients were in the EN group (522±260kcal/day) and 34 were in the control group. In the EN group, 26 of 42 patients (61.9%) achieved remission vs 25 of 34 patients (73.5%) in the control group. There was no significant difference in clinical remission rates between the 2 groups. Forty nine patients (24 in the EN and 25 in the control groups) among 51 who achieved remission during ADA induction therapy received maintenance ADA. Four of 24 patients (16.7%) in the EN group showed loss of response to ADA vs 10 of 25 (40.0%) in the control group (P<0.01). Likewise, the Kaplan‐Meier survival analysis showed significantly lower loss of response in favor of the EN group (P<0.05). There were no significant differences in other clinical risk factors for recurrence between the 2 groups. CONCLUSIONS: This is the first study that has investigated the clinical efficacy of concomitant EN in CD patients with the loss of response to ADA maintenance therapy. Based on the outcomes of the present investigation, concomitant EN should be considered in the management of CD during ADA maintenance therapy.

%Z Gastroenterology

Journal article; Conference proceeding

%U https://cochrane.66557.net/central/doi/10.1002/central/CN-01061213/full

%0 Journal Article

%A Coleman, C. I.

%A Turpie, A. G.

%A Bunz, T. J.

%A Beyer-Westendorf, J.

%A Baker, W. L.

%D 2018

%T Impact of prolonged anticoagulation with rivaroxaban on provoked venous thromboembolism recurrence: the improve-VTE study

%V 132

%8 2018-01-01

%R 10.1182/blood-2018-99-114133

%K *anticoagulation; *cancer recurrence; *deep vein thrombosis; *relapse; Adult; Bleeding; Cancer patient; Cancer staging; Chronic kidney failure; Cohort analysis; Comparative effectiveness; Conference abstract; Controlled study; Drug safety; Drug therapy; Drug withdrawal; Emergency ward; Female; Heart failure; Heredity; Hormonal therapy; Hospitalization; Human; Human cell; Incidence; Inflammatory bowel disease; Insurance; Leg injury; Lung embolism; Major clinical study; Major surgery; Paralysis; Pharmacokinetics; Pregnancy; Prescription; Probability; Propensity score; Puerperium; Risk factor; Thrombophilia

%X Background: The optimal duration of anticoagulation following an incident provoked venous thromboembolism (VTE) remains unclear. Two randomized trials have shown extended duration rivaroxaban use in patients experiencing an unprovoked or provoked VTE can reduce patients' risk of recurrent thrombosis without a significant increased risk of major bleeding compared to placebo or aspirin. Objectives: We sought to evaluate the real‐world effectiveness and safety of prolonged anticoagulation with rivaroxaban following an incident provoked VTE. Methods: Using claims data from the US Truven MarketScan databases from November 1, 2012 through March 31, 2017, we identified adult patients with a primary discharge diagnosis of VTE (deep vein thrombosis and/or pulmonary embolism) during a hospitalization or emergency department visit, who had a provoking (major or minor, persistent or transient) risk factor, at least 3‐months of continuous rivaroxaban treatment and ≥12‐months of continuous medical and prescription insurance benefits prior to their incident VTE. Patients were categorized as either continuing rivaroxaban or discontinuing anticoagulation (no anticoagulation or antiplatelet agents, with or without aspirin) after the initial 3‐months of rivaroxaban treatment (index date). Differences in baseline covariates between cohorts were adjusted for using inverse probability‐oftreatment weights (IPTW) based on propensity‐scores (residual standardized differences <0.1 achieved for all covariates after adjustment). Study endpoints included recurrent VTE and major bleeding (per the Cunningham algorithm). Patients were followed until occurrence of an endpoint, insurance disenrollment or up to 12‐months post‐index date. The incidences of recurrent VTE or major bleeding were compared between cohorts using Cox regression and reported as hazard ratios (HRs) with 95% confidence intervals (CIs). Subgroup analyses were performed for each endpoint according to whether patients had a major persisting (i.e. cancer), minor persisting (i.e. inflammatory bowel disease, lower extremity paralysis, heart failure, stage III or worse chronic kidney disease, hereditary or acquired thrombophilia), minor transient (i.e. admitted to hospital for ≥3‐consecutive days in past 3‐months, hormonal therapy, pregnancy or puerperium, leg injury with impaired mobility) or major transient risk factor (i.e. major surgery or trauma). Results: Among patients experiencing an incident provoked VTE and treated with rivaroxaban for the first 3‐months (N=4,990), continued rivaroxaban use beyond 3‐months [median (25%, 75% range) duration of additional rivaroxaban use = 3 (2, 5) months] was associated with a 44% lower hazard of recurrent VTE without significantly altering major bleeding risk when compared to anticoagulation discontinuation with or without aspirin use (Table). The highest risk of recurrent thrombosis following provoked VTE (3.1% in the discontinued anticoagulation cohort) appeared to occur in patients with a minor persisting risk factor; with continued rivaroxaban use associated with a significant 73% reduction in recurrent VTE. Conclusions: Although the absolute incidence of recurrence was low, our study suggests continuing rivaroxaban after the initial 3‐month period was associated with a decreased risk of recurrent VTE, particularly in those with a minor persisting risk factor. The observed reduction in recurrent VTE with prolonged rivaroxaban use was not associated with a significantly increased risk of major bleeding. (Table presented).

%Z Blood

Journal article; Conference proceeding

%U https://cochrane.66557.net/central/doi/10.1002/central/CN-01966601/full

%0 Journal Article

%A Narula, N.

%A Cooray, M.

%A Anglin, R.

%A Muqtadir, Z.

%A Narula, A.

%A Marshall, J. K.

%D 2017

%T Impact of High-Dose Vitamin D3 Supplementation in Patients with Crohn's Disease in Remission: a Pilot Randomized Double-Blind Controlled Study

%V 62

%N 2

%P 448‐455

%8 2017-01-01

%R 10.1007/s10620-016-4396-7

%K *25 hydroxyvitamin D; *colecalciferol; *remission; *ulcerative colitis; *visually impaired person; Adult; Affect; Anxiety; Cholecalciferol [*administration & dosage]; Clinical article; Clinical trial; Controlled clinical trial; Controlled study; Crohn Disease [blood, *drug therapy, physiopathology, psychology]; Dose‐Response Relationship, Drug; Double blind procedure; Double‐Blind Method; Drug megadose; Female; Human; Human tissue; Humans; Low drug dose; Male; Mood; Pharmacokinetics; Pilot Projects; Placebo; Randomized controlled trial; Recurrence; Recurrence risk; Registration; Safety; Vitamin D [analogs & derivatives, blood]; Vitamins [*administration & dosage]

%X AIM: To assess the tolerability and efficacy of high‐dose vitamin D3 in patients with Crohn's disease (CD). METHODS: This was a randomized, double‐blind placebo‐controlled trial of high‐dose vitamin D3 at 10,000 IU daily (n = 18) compared to 1000 IU daily (n = 16) for 12 months in patients with CD in remission. The primary outcome was change in serum 25‐hydroxy‐vitamin D levels. Secondary outcomes included clinical relapse rates and changes in mood scores. RESULTS: High‐dose vitamin D3 at 10,000 IU daily significantly improved 25‐hydroxy‐vitamin D levels from a mean of 73.5 nmol/L [standard deviation (SD) 11.7 nmol/L] to 160.8 nmol/L (SD 43.2 nmol/L) (p = 0.02). On an intention‐to‐treat basis, the rate of relapse was not significantly different between patients receiving low‐ and high‐dose vitamin D3 (68.8 vs 33.3%, p = 0.0844). In per‐protocol analysis, clinical relapse of Crohn's disease was less frequently observed in patients receiving a high dose (0/12 or 0%) compared to those receiving a low dose of 1000 IU daily (3/8 or 37.5%) (p = 0.049). Improvement in anxiety and depression scores and a good safety profile were observed in both groups treated with vitamin D3. CONCLUSIONS: Oral supplementation with high‐dose vitamin D3 at 10,000 IU daily significantly improved serum 25‐hydroxy‐vitamin D levels. Rates of clinical relapse were similar between both groups. Larger studies using high‐dose vitamin D3 for treatment of inflammatory bowel diseases are warranted. CLINICALTRIALS. GOV REGISTRATION NO: NCT02615288.

%Z Digestive diseases and sciences

Journal article

%U https://cochrane.66557.net/central/doi/10.1002/central/CN-01333086/full

%0 Journal Article

%A NCT

%D 2012

%T Impact of Disease Activity and Nutrition Treat on Postoperative Recurrence and Complications After Bowel Resection for Crohn's Disease

%8 2012-01-01

%K Crohn Disease; Recurrence

%X The investigators want to assess the impact of possible risk factors on intestinal resection and postoperative recurrence in Crohn's disease (CD) and to evaluate the disease course. In our clinical treatments, the investigators found that perioperative CDAI and nutrition treatment may have same impact on postoperative recurrence and complication in Crohn's disease. The investigators design this study to test it. The study prepare to collect the clinical data of patients who need bowel resection for CD to analyzed. The investigators divide those patients into two groups (N,I group). They accept intestinal nutrition with the respective aim that get the inflammatory index (erythrocyte sedimentation rate (ESR), C‐reactive protein (CRP), Crohns Disease Activity Index (CDAI)) norm or nutritional index norm before they accept surgery. Postoperative recurrence and complications in patients will be recorded and compared.

%Z https://clinicaltrials.gov/show/NCT01540942

Trial registry record

%U https://cochrane.66557.net/central/doi/10.1002/central/CN-02024594/full

%0 Journal Article

%A Ankersen, D.

%A Marker, D.

%A Burisch, J.

%A Munkholm, P.

%D 2016

%T Home monitoring of disease activity and fecal calproctin in adult patients with inflammatory bowel disease

%V 4

%N 5

%P A626

%8 2016-01-01

%R 10.1177/2050640616663689

%K *Crohn disease; *home monitoring; Adult; Clinical practice; Controlled study; Fatigue; Feasibility study; Female; Follow up; Human; Major clinical study; Male; Medication compliance; Quality of life; Questionnaire; Randomized controlled trial; Remission; Telehealth; Ulcerative colitis

%X Introduction: Due to the chronic relapsing nature of Inflammatory bowel disease (IBD) it is of great importance to detect and treat a relapse as soon as possible in order to decrease the total inflammation burden, decrease or postpone the risk of surgery and possibly change of disease course. A validated Fecal Calprotectin (FC) home testing kit and smart phone application (app) has been added to an existing eHealth web app, thereby making it possible for the patients to monitor their disease activity using clinical scores and FC from home. FC levels can be obtained within 18 minutes using the home testing kit and is shown on the smart phone. Results from clinical scores and FC are categorized in a traffic light manner on the eHealth web app for instant recommendation of individualized treatment strategy ‐ treat to target1. Aims & Methods: Our aim, with this one‐year randomized control trail of 120 adult IBD patients, is to determine if an eHealth screening procedure for disease activity should be implemented in clinical practice 'on demand' or 'every 3 months'. Both groups use the web program noh.constant‐care.dk to tightly monitor their disease activity either 'on demand' or 'every 3 months'. The web algorithm consists of a short disease questionnaire either Harvey‐Bradshaw Index (HBI) for Crohn's disease (CD) or Simple Clinical Colitis Activity Index (SCCAI) for Ulcerative colitis (UC) plus home monitoring of FC, which together gives a total inflammation burden scoring (TIBS). Moreover, but not a part of the TIBS disease activity web algorithm, patients also enter in the web program their fatigue score (FACIT‐F), Medication Adherence Report scale (MARS), Quality of Life (s‐IBDQ) and Copenhagen IBD Disease Course (CIDC) type. The latter will only be measured at inclusion and at follow up. Results: So far, baseline results of ninety‐eight IBD patients are available, 78 with UC and 20 with CD. All of them have fulfilled HBI or SCCAI on the web program at inclusion. Ninety (91.8%) FC results were obtained at baseline and eight (8.2%) were missing. According to the web algorithm, patients were in median in remission in both groups, 'every 3 months' vs. 'on demand', Median (interquartile range): FC in mg/kg 70 (20‐560) vs. 88 (20‐808), SCCAI: 1 (0‐3) vs. 1 (0.5‐2.5), HBI 1 (0‐2) vs. 3 (1‐6) and TIBS 5.5 (0‐33) vs. 9 (2‐34). Baseline descriptive data for the ninety‐eight patients randomized to 'on demand' or 'every 3 months' is listed in table 1 and no significant differences between the two groups were found. Conclusion: Adult IBD patients in remission regarding disease activity were included. Feasibility and usability of the web apps were acceptable and only 8.2% failed home monitoring FC test at inclusion, mostly due to technical problems prior to the app being CE‐marked and distributed from App Store and Play Store. Results are awaiting for long‐term course.

%Z United European gastroenterology journal

Journal article; Conference proceeding

%U https://cochrane.66557.net/central/doi/10.1002/central/CN-01438955/full

%0 Journal Article

%A NCT

%D 2018

%T Health Coaching as a Tool for Improving Medication Adherence in Adult Patients With Crohn's Disease

%8 2018-01-01

%K Crohn Disease

%X Crohn's disease (CD) is a chronic idiopathic intestinal disorder involving the interplay of environmental, immunomodulatory and genetic causative factors. Treatment for Crohn's disease is multimodal and includes lifestyle modification (dietary modification and smoking cessation), chronic pharmacotherapy and surgery. Given the need for chronic pharmacotherapy, medication adherence is a crucial therapeutic goal in the management of Crohn's disease. In fact, medication non‐adherence has been associated with greater risk of relapse and increased healthcare costs. In a previous study, the investigators found clinically identifiable risk factors for non‐adherence for self‐injectable biologic medications in a population with moderate to severe CD. These risk factors included smoking, prior biologic use, psychiatric history, and current narcotic use. The primary objective of this study is to use a multidisciplinary team approach that implements a targeted coaching intervention to promote behavioral change and improve medication adherence in adult patients with Crohn's disease who are at high risk of non‐adherence.

%Z https://clinicaltrials.gov/show/NCT03757533

Trial registry record

%U https://cochrane.66557.net/central/doi/10.1002/central/CN-01795162/full

%0 Journal Article

%A Gutierrez, A.

%A Zapater, P.

%A Juanola, O.

%A Sempere, L.

%A Garcia, M.

%A Laveda, R.

%A Martinez, A.

%A Scharl, M.

%A Gonzalez-Navajas, J. M.

%A Such, J.

%A Et, Al.

%D 2016

%T Gut bacterial DNA translocation is an independent risk factor of flare at short term in patients with crohn's disease

%V 111

%N 4

%P 529‐540

%8 2016-01-01

%R 10.1038/ajg.2016.8

%K *Crohn disease/dt [Drug Therapy]; *bacterial DNA/ec [Endogenous Compound]; *gene translocation; *genomic DNA/ec [Endogenous Compound]; *intestine flora; *recurrent disease; Adalimumab/cb [Drug Combination]; Adalimumab/dt [Drug Therapy]; Adult; Article; Azathioprine/cb [Drug Combination]; Azathioprine/dt [Drug Therapy]; Bacterial gene; Calgranulin/ec [Endogenous Compound]; Caspase recruitment domain protein 15/ec [Endogenous Compound]; Colonoscopy; Controlled clinical trial; Controlled study; Crohn Disease Activity Index; Female; Follow up; Genetic Variation; Genotype; Hospital admission; Hospitalization; Human; Immunosuppressive treatment; Infliximab/cb [Drug Combination]; Infliximab/dt [Drug Therapy]; Major clinical study; Male; Mesalazine/dt [Drug Therapy]; Methotrexate/cb [Drug Combination]; Methotrexate/dt [Drug Therapy]; Multicenter study; Nonhuman; Priority journal; Prospective study; RNA 16S/ec [Endogenous Compound]; Relapse; Remission; Risk factor; Steroid therapy; Steroid/cb [Drug Combination]; Steroid/dt [Drug Therapy]

%X OBJECTIVES:We aimed at evaluating bacterial DNA (bactDNA) presence in blood of Crohn's disease (CD) patients in remission as an independent risk factor of flare at 6 months.METHODS:This is a prospective, multicenter study on CD patients with Crohn's disease activity index (CDAI)<150. The primary end point was time‐to‐relapse as evaluated by CDAI>150 in the following 6 months. BactDNA in blood, the nucleotide‐binding oligomerization domain containing 2 (NOD2) genotype, and serum cytokine levels were determined at baseline.RESULTS:A total of 288 patients were included. BactDNA was detected in 98 patients (34.0%). A variant‐NOD2 genotype was identified in 114 patients (39.6%). Forty patients (14%) relapsed during follow‐up. Multivariate survival analysis identified bactDNA as an independent risk factor of flare (hazard ratio (HR) 8.75 (4.02‐19.06) 95% confidence interval (CI)). Hospitalization, surgery, switch of treatment, initiation and escalation of anti‐tumor necrosis factor (TNF) therapy, steroids initiation, and increased fecal calprotectin levels at 6 months were associated with bactDNA at baseline. A logistic regression analysis showed bactDNA as an independent and significant predictive factor of hospitalization (odds ratio (OR) 11.9 (3.4‐42.3); P<0.001), steroids startup (OR 8.5 (2.7‐27.1); P<0.001), and switch of treatment (OR 3.5 (1.6‐7.7); P=0.002) at 6 months. No relationship was observed between bactDNA and mucosal lesions in patients with colonoscopy at admission. Serum pro‐inflammatory cytokines were significantly increased in patients with bactDNA or a variant‐NOD2 genotype. The combination of both factors induced decreased anti‐TNF‐alpha levels and a higher percentage of patients on intensified anti‐TNF therapy.CONCLUSIONS:BactDNA is an independent risk factor of relapse at 6 months in CD patients. BactDNA is also independently associated with an increased risk of hospitalization, switch of treatment, and steroids initiation.

%Z American journal of gastroenterology

Journal article

%U https://cochrane.66557.net/central/doi/10.1002/central/CN-01159561/full

%0 Journal Article

%A NCT

%D 2016

%T GIS-SUSANTI-TNF-2015 (Anti-TNF Discontinuation )

%8 2016-01-01

%K Adalimumab; Colitis; Colitis, Ulcerative; Crohn Disease; Inflammatory Bowel Diseases; Infliximab; Intestinal Diseases; Pharmaceutical Solutions; Ulcer

%X A multicentre prospective randomized trial. Hypothesis: The discontinuation of anti‐TNF treatment in inflammatory bowel disease (IBD) patients in clinical remission is associated with an increased risk of recurrence compared with maintaining such treatment. Main objective: To assess the percentage of patients with IBD who, after stopping anti‐TNF treatment, have sustained clinical remission at one year compared to those in which the treatment is continued at stable doses Secondary objectives: To compare treatment discontinuation vs. treatment continuation of anti‐TNF agents in patients with Crohn´s disease or ulcerative colitis in terms of: 1. remission (relapse‐free) time, 2. phenotype changes with both strategies 3. mucosal healing, 4. radiologic healing 5. impact on quality of life and productivity 6. safety 7. to identify relapse predictive factors. 8. To identify relapse predictive factors after anti‐TNF drug discontinuation 9. Determining the profile of serum cytokines in patients with both strategies, depending on drug exposure and if maintained clinical remission or relapse. Planned number of subject to be included: 194 The participation of at 50 hospitals in Spain with an inclusion of about 5 patients per hospital is required.. Case report Form was designed on REDCap (a free, secure, web‐based application designed to support data capture for research studies).

%Z https://clinicaltrials.gov/show/NCT02994836

Trial registry record

%U https://cochrane.66557.net/central/doi/10.1002/central/CN-02048198/full

%0 Journal Article

%A Allegretti, J. R.

%A Kelly, C.

%A Grinspan, A. M.

%A Mullish, B. H.

%A Hurtado, J.

%A Nemes, S.

%A Sagi, S.

%A Bohm, M.

%A Kassam, Z.

%A Fischer, M.

%D 2020

%T Fecal Microbiota Transplantation Decolonizes C. difficile in Patients with Inflammatory Bowel Disease and Concomitant C. difficile Infection

%V 115

%N SUPPL

%P S326

%8 2020-01-01

%R 10.14309/01.ajg.0000704648.18611.4c

%K *Clostridium difficile infection; *Crohn disease; *fecal microbiota transplantation; Adult; Clinical article; Cohort analysis; Conference abstract; Controlled study; Drug safety; Female; Follow up; Human; Male; Multicenter study; Outcome assessment; Polymerase chain reaction; Prevention; Prospective study; Public health; Randomized controlled trial; Tertiary care center; Treatment failure; Ulcerative colitis

%X INTRODUCTION: Patients with inflammatory bowel disease (IBD) are at increased risk for C. difficile infection (CDI) and associated morbidity and mortality. Persistent C. difficile colonization occurs even after standard‐of‐care CDI antibiotics, and may contribute to the increased risk of recurrent CDI. Additionally, C. difficile colonization has public health consequences as patients may spread C. difficile and may lead to contact isolation and high‐cost cleaning procedures in a healthcare setting. Fecal microbiota transplantation (FMT) is a promising therapy for recurrent CDI, however its efficacy among IBD patients is limited and the rate of decolonization is unknown. Accordingly, we assessed the safety, efficacy and C. difficile decolonization rate in in IBD patients with recurrent CDI following FMT. METHODS: This open‐label, prospective single‐arm multicenter cohort study enrolled patients from 4 tertiary care centers. Patients with IBD and 2 or more episodes of CDI received a single colonoscopic FMT from a universal stool bank. CDI testing (GDH/Toxin EIA and PCR) was performed pre‐FMT, and at 1, 8, and 12 weeks post‐FMT. The primary outcome was FMT failure through week 8 (defined as diarrhea and Toxin EIA positive for C. difficile). The secondary outcome assessed was C. difficile decolonization post‐FMT (defined as negative PCR testing) at week 12. RESULTS: 50 participants were enrolled with a mean age of 43.3 years (range 21‐91) and primarily female (n = 28, 56%), 14 of which had Crohn's disease (CD) (mean HBI = 6.4) and 36 had ulcerative colitis (UC) (mean Partial Mayo Score = 4.5). One patient did not receive FMT after enrollment. There were no treatment‐related serious adverse events. Among the treated patients, 8% (4/49) participants experienced a confirmed FMT failure and received a second FMT. Two additional patients received a repeat FMT based on investigator discretion. In terms of C. difficile decolonization, 88.9% (40/45 eligible participants) experienced decolonization at week 12 follow‐up period. CONCLUSION: To our knowledge, this is the largest prospective trial to assess the safety, efficacy and C. difficile decolonization rate of FMT in patients with IBD‐CDI. This data suggests FMT is a safe and promising therapy for preventing CDI recurrence and achieving C. difficile decolonization which may have public health and economic implications. Future randomized controlled trials are needed.

%Z American journal of gastroenterology

Journal article; Conference proceeding

%U https://cochrane.66557.net/central/doi/10.1002/central/CN-02212600/full

%0 Journal Article

%A Hellstrom, P. M.

%A Lonnkvist, M.

%A Diaz Tartera, H. O.

%A Befrits, R.

%A Holst, M.

%D 2018

%T Faecal calprotectin predicts sustained treatment response of infliximab induction therapy superior to C-reactive protein and clinical activity scores in inflammatory bowel disease

%V 12

%N Supplement 1

%P S310‐S311

%8 2018-01-01

%K *C reactive protein; *calgranulin; *inflammatory bowel disease; *infliximab; *treatment response; Adult; Azathioprine; CD47 antigen; Clinical assessment; Conference abstract; Controlled clinical trial; Controlled study; Disease exacerbation; Drug combination; Drug therapy; Endogenous compound; Female; Gene expression; Human; Incidence; Kaplan Meier method; Male; Monitoring; Receiver operating characteristic; Relapse; Risk assessment; Sensitivity and specificity; Surgery; adult; clinical assessment; conference abstract; controlled clinical trial; controlled study; disease exacerbation; drug combination; drug therapy; female; gene expression; human; incidence; inflammatory bowel disease; male; monitoring; receiver operating characteristic; relapse; risk assessment; sensitivity and specificity; surgery; treatment response

%X Background: Faecal calprotectin (FC) and C‐reactive protein (CRP) are non‐invasive markers of inflammation that are well associated with clinical activity in patients with Crohn's disease and ulcerative colitis. However, there is still not fully known if they can be used to monitor anti‐TNF therapy with infliximab and if values of FC and CRP can be used to predict relapse after infliximab induction therapy, or if clinical disease activity scores can be used as well. The aim of our study was to assess if FC and CRP can be implemented into clinical routine when monitoring therapy response and risk of relapse in UC and CD patients after infliximab induction therapy (0, 2, and 6 weeks). Methods: FC and CRP were analysed in 123 (76 CD, 47 UC) patients before infliximab induction therapy and after 12 weeks. Clinical activity was assessed by the Harvey Bradshaw index (HBi) for CD and the partial Mayo score for UC. Clinical activity was evaluated before and after infliximab induction and then related to values of FC and CRP. Responders were then followed for further 48 weeks to seek for eventual incidence (shortened dosage interval, increased dosage, surgery). Data were presented as mean and standard deviation, or medians and range as appropriate. The likelihood ratio, sensitivity, and specificity of FC and CRP were analysed using receiver‐operating characteristic (ROC) curves. Kaplan‐Meier survival curves were used to assess disease progression. Results: FC, CRP, and clinical indices decreased from baseline and after infliximab induction in both CD and UC patients. Patients treated with infliximab in combination with azathioprine showed no significant difference in FC and CRP levels compared with patients treated with infliximab alone. Clinical disease activity correlated with FC and was similar to CRP. FC was also correlated to CRP in a linear manner. The FC ROC best cut‐off point was 221 μg/g, with a likelihood ratio of 2 (sensitivity 66% specificity 67%). Levels of FC exceeding 221 μg/g at induction therapy were associated with an increased risk of meeting an incident within the forthcoming 48 weeks. Similarly, the corresponding cut‐off value of CRP 2.1 mg/l, with a likelihood ratio of 1.4 (sensitivity 54% and specificity 60%) was associated with an incident within 48 weeks. Conclusions: We show that FC and CRP are associated to the inflammatory activity in patients with CD and UC before and after infliximab induction evaluated by activity scores. We found that FC is better used than CRP and clinical activity scores to predict sustained treatment response. Levels of FC >221 μg/g after infliximab induction therapy were associated with an increased risk of a disease incident (dosage increase, shortening of dose interval, surgery) within the next 48 weeks.

%Z Journal of Crohn's & colitis

Journal article; Conference proceeding

%U https://cochrane.66557.net/central/doi/10.1002/central/CN-01466696/full

%0 Journal Article

%A NCT

%D 2019

%T Extended-Duration Low-Dose Apixaban to Prevent Recurrence in High-Risk Patients With Provoked Venous Thromboembolism

%8 2019-01-01

%X Inclusion Criteria: ‐ Man or woman ‐ Age = 18 years ‐ Objectively‐confirmed DVT and/or PE ‐ Treated for at least 3 months with standard therapeutic anticoagulant therapy ‐ Has not suffered symptomatic recurrence during prior anticoagulant therapy ‐ Outpatient follow‐up at BWH ‐ AND have at least one of the following persistent provoking VTE risk factors: ‐ Persistent immobility (defined as paralysis, other inability to ambulate freely, bed‐bound, wheelchair‐bound) ‐ Obesity (defined as BMI = 30 kg/m2) ‐ Heart failure (systolic, diastolic, or combined) ‐ Chronic lung disease (COPD, asthma, interstitial lung disease) ‐ Chronic kidney disease (nephrotic/nephritic syndrome, renal dysfunction with creatinine = 2.5 mg/dL) ‐ Chronic inflammatory/autoimmune disorder (inflammatory arthritis, vasculitis, inflammatory bowel disease, chronic infection) ‐ Atherosclerotic cardiovascular disease (coronary, cerebrovascular, or peripheral artery disease) (up to 35% in each study group may have atherosclerotic cardiovascular disease as a qualifying persistent risk factor) ‐ NOTE: Eligible patients will be allowed to have multiple risk factors, and there will not be a limit as to how many of the above risk factors a subject may have. In addition, we will place no limit on the number of patients included with multiple risk factors. A study population with multiple risk factors is highly representative of the provoked VTE population and will provide the greatest generalizability of the study results to real‐world clinical practice. Including patients with single and multiple persistent provoking risk factors will also facilitate enrollment. As noted, there is clinical and research equipoise regarding whether patients with a single or multiple persistent provoking VTE risk factors should receive extended duration thromboprophylaxis for secondary prevention. ‐ Willing to provide written informed consent Exclusion Criteria: ‐ Women who are pregnant or breastfeeding ‐ Women of child‐bearing potential who are unwilling or unable to use an acceptable method of birth control (such as oral contraceptives, other hormonal contraceptives [vaginal products, skin patches, or implanted or injectable products], or mechanical products such as an intrauterine device or barrier methods [diaphragm, condoms, spermicides]) to avoid pregnancy for the entire study ‐ Active cancer within the past 5 years ‐ Contraindication to antithrombotic or antiplatelet therapy ‐ Requirement for ongoing anticoagulant therapy, dual antiplatelet therapy, P2Y12 inhibition, or aspirin at a dose of > 81 mg daily ‐ Hemoglobin level < 9 mg/dL, a platelet count < 100,000/mm3, a serum creatinine level > 2.5 mg/dL, an ALT or AST level > 2 times the upper limit of the normal range, or a total bilirubin level > 1.5 times the upper limit of the normal range ‐ History of bleeding diathesis or have had recent active bleeding ‐ Active severe hepatobiliary disease ‐ More than 6 months that have elapsed without taking an anticoagulant or low‐dose aspirin o NOTE: The risk of recurrent VTE following cessation of anticoagulation rises slowly over the first 3‐6 months (26). After this initial period, the cumulative risk of recurrent VTE steepens. Using a limit of no greater than 6 months of interruption in anticoagulation before potential reinitiation of anticoagulation as part of this trial will safely facilitate enrollment as opposed to restricting the population to no greater than 3 months of interruption. ‐ Known severe thrombophilia (any increased titer antiphospholipid antibody or positive lupus anticoagulant/DRVVT or deficiency of antithrombin, protein C, or protein S) which would indicate long‐term full therapeutic anticoagulation with a vitamin K antagonist ‐ Life expectancy < 12 months or hospice care ‐ Prisoners or subjects who are involuntarily incarcerated ‐ Subjects who are compulsorily detained for treatment of either a psychiatric or physical (e.g., infectious disease) illness ‐ Receiving concurrent non‐FDA‐approved or investigational agents or has received an investigational agent within the past 30 days prior to the first dose of study treatment (with the exception of approved medications being used for an approved indication, e.g., investigating a new dosing regimen for an approved indication) ‐ Any condition, which in the opinion of the investigator, would put the subject at an unacceptable risk from participating in the study ‐ Any other medical, social, logistical, or psychological reason, which in the opinion of the investigator, would preclude compliance with, or successful completion of, the study protocol Deep Vein Thrombosis;Pulmonary Embolism;Venous Thromboembolism

%Z Trial registry record

%U https://cochrane.66557.net/central/doi/10.1002/central/CN-02004437/full

%0 Journal Article

%A Garcia-Olmo, D.

%A Panes, J.

%A Brayshaw, N.

%A Richard, M. P.

%A Agboton, C.

%D 2018

%T EXPANDED ALLOGENEIC ADIPOSE DERIVED MESENCHYMAL STEM CELL THERAPY (CX601) FOR COMPLEX PERIANAL FISTULAS IN CROHN'S DISEASE HAS RAPID ONSET OF CLINICAL REMISSION AND LOW RELAPSE RATE BY WEEK 52

%V 154

%N 6

%P S‐370

%8 2018-01-01

%R 10.1016/S0016-5085(18)31548-8

%K *Crohn disease; *anus fistula; *mesenchymal stem cell transplantation; *recurrence risk; *remission; Adult; Clinical assessment; Conference abstract; Controlled study; Drug therapy; Female; Finger; Human; Kaplan Meier method; Major clinical study; Male; Nuclear magnetic resonance imaging; Phase 3 clinical trial; Randomized controlled trial

%X Background: Cx601 is an expanded allogeneic adipose‐derived mesenchymal stem cell therapy developed as a local therapy for complex perianal fistulas in patients with Crohn's disease (CD). In the Phase 3, pivotal, ADMIRE‐CD study of patients with CD and treatment‐refractory, draining complex perianal fistulas, more patients receiving Cx601 achieved clinical remission at week 24 compared with standard of care (SoC). Here we describe time to clinical remission and relapse rates by week 52 in ADMIRE‐CD. Methods: Clinical remission was defined as closure of all treated external openings that were draining at baseline despite gentle finger pressure. Relapse by week 52 was defined as reopening of any treated external opening with active drainage as clinically assessed, and/or development of a perianal collection >2 cm of treated perianal fistulas confirmed by centrally blinded MRI assessment in patients with combined remission at week 24 (i.e. clinical remission and no collections >2 cm). Confidence intervals for difference in rates were calculated by the asymptotic Wald method. Times to clinical remission and relapse were analysed by Cox regression with accompanying Kaplan‐Meier estimates. Analyses of subgroups with and without concomitant medication were also performed. Results: The intent‐to‐treat population comprised 212 randomized patients. At the first time point after baseline (week 6), 51.4% of patients receiving Cx601 were in clinical remission vs 34.3% in the SoC group (absolute difference 17.1 percentage points; 95% confidence interval [CI]: 4.0‐30.2; relative difference 49.9%). The difference persisted across visits and at week 52 was 17.0 percentage points (57.0% vs 40.0% for Cx601 and SoC, respectively; 95% CI: 3.8‐30.3; relative difference 42.5%). The median time to clinical remission in patients receiving Cx601 was 6.7 weeks (95% CI: 6.4‐11.9) compared with 14.6 weeks (95% CI: 11.9‐22.9) in the SoC group (hazard ratio [HR]=1.72 [95% CI: 1.25‐2.38]). By week 52, 25.0% (13/52) of patients receiving Cx601 relapsed vs 44.1% (15/34) in the SoC group (difference of minus 19.1 percentage points; 95% CI: ‐39.5 to 1.3). A longer time to relapse by week 52 was observed for patients receiving Cx601 compared with the SoC group (HR=0.47 [95% CI: 0.22‐1.00]). Conclusion: Patients receiving Cx601 experienced a shorter time to clinical remission and a longer duration of remission compared with SoC. This was observed in subgroups of patients with and without concomitant medication. These results support the efficacy of Cx601 in patients with CD and treatment‐refractory, draining complex perianal fistulas.

%Z Gastroenterology

Journal article; Conference proceeding

%U https://cochrane.66557.net/central/doi/10.1002/central/CN-01964869/full

%0 Journal Article

%A Garcia-Olmo, D.

%A Herreros, D.

%A Pascual, I.

%A Pascual, J. A.

%A Del-Valle, E.

%A Zorrilla, J.

%A De-La-Quintana, P.

%A Garcia-Arranz, M.

%A Pascual, M.

%D 2009

%T Expanded adipose-derived stem cells for the treatment of complex perianal fistula: a phase II clinical trial

%V 52

%N 1

%P 79‐86

%8 2009-01-01

%R 10.1007/DCR.0b013e3181973487

%K Adipocytes [*cytology]; Adult; Crohn Disease [complications]; Female; Fibrin Tissue Adhesive [adverse effects, *therapeutic use]; Humans; Male; Mesenchymal Stem Cell Transplantation [adverse effects]; Middle Aged; Rectal Fistula [etiology, pathology, *therapy]; Tissue Adhesives [*administration & dosage, adverse effects]; Wound Healing

%X PURPOSE: The feasibility and safety of stem cell‐based therapy with expanded adipose‐derived stem cells (ASCs) has been investigated in a phase I clinical trial. The present study was designed as a phase II multicenter, randomized controlled trial to further investigate the effectiveness and safety of ASCs in the treatment of complex perianal fistulas. METHODS: Patients with complex perianal fistulas (cryptoglandular origin, n = 35; associated with Crohn's disease, n = 14) were randomly assigned to intralesional treatment with fibrin glue or fibrin glue plus 20 million ASCs. Fistula healing and quality of life (SF‐12 questionnaire) were evaluated at eight weeks and one year. If healing was not seen at eight weeks, a second dose of fibrin glue or fibrin glue plus 40 million ASCs was administered. RESULTS: Fistula healing was observed in 17 (71 percent) of 24 patients who received ASCs in addition to fibrin glue compared with 4 (16 percent) of 25 patients who received fibrin glue alone (relative risk for healing, 4.43; confidence interval, 1.74‐11.27); P < 0.001). The proportion of patients with healing was similar in Crohn's and non‐Crohn's subgroups. ASCs were also more effective than fibrin glue alone in patients with a suprasphincteric fistulous tract (P = 0.001). Quality of life scores were higher in patients who received ASCs than in those who received fibrin glue alone. At one year follow‐up, the recurrence rate in patients treated with ASCs was 17.6 percent. Both treatments were well tolerated. CONCLUSION: Administration of expanded ASCs (20 to 60 million cells) in combination with fibrin glue is an effective and safe treatment for complex perianal fistula and appears to achieve higher rates of healing than fibrin glue alone.

%Z Diseases of the colon and rectum

Journal article

%U https://cochrane.66557.net/central/doi/10.1002/central/CN-00686412/full

%0 Journal Article

%A Feleshtynskiy, Y.

%A Mylianovska, A.

%A Pirogovsky, V.

%A Dyadyk, O.

%D 2021

%T Evaluation of the endoscopic treatment with topical prednisolone administration for intestinal strictures in Crohn's disease

%V 94

%N 1

%P 28‐33

%8 2021-01-01

%R 10.5604/01.3001.0015.2471

%K Colonoscopy [adverse effects]; Constriction, Pathologic [drug therapy, etiology, surgery]; Crohn Disease [drug therapy]; Dilatation [adverse effects, methods]; Humans; Intestinal Obstruction [surgery]; Prednisolone [therapeutic use]; Retrospective Studies; Treatment Outcome

%X Introduction: The prevalence of Crohn's disease amounts to 5.9 cases per 100,000 population. Complications such as intestinal strictures usually occur within a long course of Crohn's disease. Intestinal resection for stricture does not prevent a stricture recurrence and the need for repeated resections, which in turn leads to the formation of short intestine syndrome. The advantage of endoscopic balloon dilatation is organ preservation and a quick clinical therapeutic effiect. However, the frequency of recurrences after conventional endoscopic balloon dilatation of the intestinal stricture in Crohn's disease is still at a relatively high level and amounts to 59%, which justifies the need to improve the endoscopic dilatation technique. Aim: The aim of this study to improve the treatment effectiveness for intestinal strictures in Crohn's disease using endoscopic balloon dilatation combined with prednisolone injection in the stricture area. Materials and methods: Endoscopic treatment for intestinal strictures in Crohn's disease was performed in 64 patients. Depending on the endoscopic technique, patients were randomized into 2 groups. The first group consisted of 32 (50%) patients who underwent conventional endoscopic balloon dilatation of strictured areas. The second group consisted of 32 (50%) patients in whom an endoscopic balloon dilatation in combination with submucosal injection of prednisolone to the area of stricture after dilatation was performed. Patient groups were comparable in age, sex and length of stricture. Results and conclusions: The results showed that endoscopic balloon dilatation with administration of 40 mg of prednisolone in group II patients was more effective compared to conventional balloon dilatation. The recurrence rate was reduced from 34.4% to 9.3%. The risk of recurrence of intestinal stricture in group I during the first year of observation was found to be 4.5 times higher ‐ HR = 4.5 (1.6‐12.9); P = 0.010. The effectiveness of advanced endoscopic balloon dilatation for intestinal strictures was confirmed by colonoscopy with patomorphological examination of the intestinal mucosa 6 months after dilation in patients of both groups.</br>.

%Z Polski przeglad chirurgiczny

Journal article

%U https://cochrane.66557.net/central/doi/10.1002/central/CN-02380181/full

%0 Journal Article

%A Feleshtynskiy, Y.

%A Mylianovska, A.

%A Pirogovsky, V.

%A Dyadyk, O.

%D 2021

%T Evaluation of the endoscopic treatment with topical prednisolone administration for intestinal strictures in Crohn's disease

%V 94

%N 1

%P 28‐33

%8 2021-01-01

%R 10.5604/01.3001.0015.2471

%K *Crohn disease; *balloon dilatation; *stenosis; Adult; Article; Clinical trial; Colonoscopy; Controlled study; Drug therapy; Endoscopic surgery; Female; Human; Intestine mucosa; Major clinical study; Male; Organ preservation; Randomized controlled trial; Recurrence risk; Surgery; Topical drug administration

%X Introduction: The prevalence of Crohn's disease amounts to 5.9 cases per 100,000 population. Complications such as intestinal strictures usually occur within a long course of Crohn's disease. Intestinal resection for stricture does not prevent a stricture recurrence and the need for repeated resections, which in turn leads to the formation of short intestine syndrome. The advantage of endoscopic balloon dilatation is organ preservation and a quick clinical therapeutic effiect. However, the frequency of recurrences after conventional endoscopic balloon dilatation of the intestinal stricture in Crohn's disease is still at a relatively high level and amounts to 59%, which justifies the need to improve the endoscopic dilatation technique. Aim: The aim of this study to improve the treatment effectiveness for intestinal strictures in Crohn's disease using endoscopic balloon dilatation combined with prednisolone injection in the stricture area. Materials and methods: Endoscopic treatment for intestinal strictures in Crohn's disease was performed in 64 patients. Depending on the endoscopic technique, patients were randomized into 2 groups. The first group consisted of 32 (50%) patients who underwent conventional endoscopic balloon dilatation of strictured areas. The second group consisted of 32 (50%) patients in whom an endoscopic balloon dilatation in combination with submucosal injection of prednisolone to the area of stricture after dilatation was performed. Patient groups were comparable in age, sex and length of stricture. Results and conclusions: The results showed that endoscopic balloon dilatation with administration of 40 mg of prednisolone in group II patients was more effective compared to conventional balloon dilatation. The recurrence rate was reduced from 34.4% to 9.3%. The risk of recurrence of intestinal stricture in group I during the first year of observation was found to be 4.5 times higher ‐ HR = 4.5 (1.6‐12.9); P = 0.010. The effectiveness of advanced endoscopic balloon dilatation for intestinal strictures was confirmed by colonoscopy with patomorphological examination of the intestinal mucosa 6 months after dilation in patients of both groups.</br>.

%Z Polski przeglad chirurgiczny

Journal article

%U https://cochrane.66557.net/central/doi/10.1002/central/CN-02384776/full

%0 Journal Article

%A Hengel, R. L.

%A Ritter, T. E.

%A Nathan, R. V.

%A Van Anglen, L. J.

%A Schroeder, C. P.

%A Marcella, S.

%A Garey, K. W.

%D 2018

%T Evaluation of Bezlotoxumab in Prevention of Recurrent C. Difficile Infection: a Multicenter Single-Arm Study in Outpatient Infusion Centers

%V 5

%P S186

%8 2018-01-01

%R 10.1093/ofid/ofy210.510

%K *outpatient; Aged; Antibiotic therapy; Clostridium difficile infection; Comorbidity; Conference abstract; Controlled study; Drug therapy; Fecal microbiota transplantation; Feces analysis; Female; Follow up; Human; Inflammatory bowel disease; Major clinical study; Male; Medical record; Multicenter study; Randomized controlled trial; Relapse; Retrospective study; Risk assessment; Risk factor; Stomach acid

%X Background. Bezlotoxumab (BEZ) was approved in October 2016 for the prevention of recurrent C. difficile (rCDI) infection in patients receiving standard‐ofcare (SoC) antibiotic therapy for active CDI who are at high risk for CDI recurrence. Presently, there are little real‐world data on recurrence rates and factors associated with recurrence in patients receiving BEZ. This study describes characteristics of patients receiving BEZ in US Outpatient Infusion Centers (OICs) and analyzes subsequent CDI recurrences. Methods. Medical records from 24 OICs were retrospectively reviewed of all patients treated with BEZ through December 2017. Data collected included demographics, comorbidities, and all therapy parameters, including SoC antibiotic therapy. Risk factors for rCDI were assessed and included age, immunocompromised status, prior number of CDI episodes, use of gastric acid suppressants, inflammatory bowel disease (IBD), and history of fecal microbiota transplant (FMT). rCDI, defined as diarrhea lasting ≥2 days with treatment for CDI with or without a positive stool test for toxigenic C. difficile, was assessed through a follow‐up visit or phone call 90 days post BEZ administration. Risk factors for rCDI were evaluated using Student's t‐test and Pearson χ2 test. Results. Eighty patients received BEZ (10 mg/kg) with 78 available for follow‐up evaluation for rCDI ≥90 days post treatment. Mean age was 65 ± 16 years with 51% female. Mean number of CDI episodes were 3 ± 1 with a mean Charlson score of 4 ± 3. SoC antibiotics included vancomycin (66% of patients) with 41% on long‐term taper, fidaxomicin (33% of patients), and metronidazole (25% of patients). Nineteen (24%) patients received more than one SoC antibiotic during their treatment course, most commonly with metronidazole and another SoC antibiotic. Of the 78 patients with follow‐ up data, 17 (22%) developed rCDI with a mean time to recurrence of 33 ± 22 days. Risk factors for rCDI are shown in the table. The use of BEZ earlier in the disease course (first or second CDI episode) was associated with a decreased risk of rCDI (OR: 0.21 95% CI: 0.04‐0.98; P = 0.033). Conclusion. In highly comorbid patients with recurrent C. difficile infection, bezlotoxumab use was effective in prevention of recurrence at 90 days and consistent with that of the randomized trials. (Figure Preseted).

%Z Open forum infectious diseases

Journal article; Conference proceeding

%U https://cochrane.66557.net/central/doi/10.1002/central/CN-01989470/full

%0 Journal Article

%A De Cruz, P.

%A Hamilton, A. L.

%A Burrell, K. J.

%A Gorelik, A.

%A Liew, D.

%A Kamm, M. A.

%D 2022

%T Endoscopic Prediction of Crohn's Disease Postoperative Recurrence

%V 28

%N 5

%P 680‐688

%8 2022-01-01

%R 10.1093/ibd/izab134

%K *Crohn disease; *prediction; *prognosis; *treatment response; Adalimumab [therapeutic use]; Adult; Article; Clinical trial; Colonoscopy; Controlled study; Crohn Disease [drug therapy, surgery]; Drug therapy; Female; High risk patient; Human; Humans; Ileum [surgery]; Intensive care; Intestine resection; Jejunum ulcer; Major clinical study; Male; Randomized controlled trial; Receiver operating characteristic; Recurrence; Risk assessment; Sensitivity and specificity; Stenosis; Surgery; Ulcer [drug therapy]

%X BACKGROUND: The presence and severity of endoscopic recurrence after Crohn's disease intestinal resection predicts subsequent disease course. The Rutgeerts postoperative endoscopic recurrence score is unvalidated but has proven prognostically useful in many clinical studies. This study aimed to investigate the association between specific early endoscopic findings and subsequent disease course. METHODS: In the setting of a randomized controlled trial (the POCER study), 85 patients underwent colonoscopy at 6 and 18 months after intestinal resection. Patients received 3 months of metronidazole, and high‐risk patients received a thiopurine (or adalimumab if they were thiopurine intolerant). For endoscopic recurrence (Rutgeerts score ≥i2) at 6 months, patients stepped up to a thiopurine, fortnightly adalimumab with thiopurine, or weekly adalimumab. Central readers confirmed Rutgeerts, Simple Endoscopic Score for Crohn's Disease, Crohn's Disease Endoscopic Index of Severity scores, and 5 newly tested endoscopic parameters: anastomotic ulcer depth (superficial vs deep), number of ulcers (0, ≤2, >2), ulcer size (1‐5 mm, ≥6 mm), circumferential extent of ulceration (<25%, ≥25%), and the presence or absence of stenosis. The POCER index, based on the 6‐month postoperative findings, was then developed in relation to predicting the endoscopic outcome at 18 months. RESULTS: Of the 5 parameters, the combination of ulcer depth and circumference at the anastomosis at 6 months was associated with endoscopic recurrence at 18 months (odds ratio [OR], 1.6; 95% confidence interval [CI], 1.03‐2.50; P = 0.035) with an area under the receiver operating characteristic curve of 0.62 (95% CI, 0.5‐0.75). The combination of these 2 parameters formed the basis of the POCER index (range, 0‐4 with 0 denoting no ulcers and 4 denoting deep ulceration with >25% circumferential involvement). The new index had a strong correlation with the Rutgeerts score measured at the same time points: Spearmans' r = .80 at 6 months and r = .77 at 18 months (P < 0.001 at both time points). A POCER index of ≥2 and a Rutgeerts score of ≥i2 both had a sensitivity of 0.41 for recurrence; however, the POCER index had a higher specificity (0.8 and 0.67, respectively). The POCER index at 6 months was associated with endoscopic recurrence at 18 months (OR, 1.5; 95% CI, 1.2‐2.0; P = 0.002; area under the receiver operating characteristic curve of 0.70; 95% CI, 0.57‐0.82), but the Rutgeerts score was not (OR, 1.2; 95% CI, 0.8‐1.8; P = 0.402). CONCLUSIONS: The POCER postoperative index comprises 2 key endoscopic factors related to the anastomosis that are associated with subsequent disease progression. A higher score, comprising the adverse prognostic factors of deep or circumferentially extensive anastomotic ulceration, may help identify patients who require more intensive therapy.

%Z Inflammatory bowel diseases

Journal article

%U https://cochrane.66557.net/central/doi/10.1002/central/CN-02292264/full

%0 Journal Article

%A Camba, A. H.

%A Carrillo-Palau, M.

%A Ramos, L.

%A Alonso-Abreu, I.

%A Hernandez, N.

%A Alarcon, O.

%A Jimenez-Sosa, A.

%A Quintero, E.

%D 2013

%T E-mail reminders and adherence rates to medical treatment in patients with inflammatory bowel disease

%V 144

%N 5

%P S779

%8 2013-01-01

%K *enteritis; *e‐mail; *gastrointestinal disease; *human; *patient; *therapy; Biological therapy; Colitis; Consultation; Crohn disease; Diagnosis; Drug therapy; Female; Follow up; Health care cost; Hospital; Hospitalization; Informed consent; Internet; Medical information; Patient compliance; Pregnant woman; Prospective study; Quality of life; Questionnaire; Relapse; Risk; Steroid; Surgery; Ulcerative colitis; biological therapy; colitis; consultation; diagnosis; drug therapy; enteritis; e‐mail; female; follow up; gastrointestinal disease; health care cost; hospital; hospitalization; human; informed consent; medical information; patient; patient compliance; pregnant woman; prospective study; quality of life; questionnaire; relapse; risk; surgery; therapy; ulcerative colitis

%X BACKGROUND: Non‐adherence to medical treatment in patients with inflammatory bowel disease (IBD) is a well known fact and it is associated with an increased risk of relapse and health care costs. Usefulness of e‐mail reminders to improve patient compliance has not been reported previously. METHODS: We performed a prospective study at the Hospital Universitario de Canarias. Patients with IBD diagnosed at least 6 months before, 18 y or older were included. We excluded patients on steroids, pregnant women and those unable to sign the informed consent. Demographic data, including Internet use, were registered Medical data (years from diagnosis, type and extension of IBD, medication, related surgery Mayo or CDAI index and a quality of life questionnaire) were also recorded. Adherence to medical treatment was determined with a previously validated questionnaire. We included three groups of patients: group A Internet non users, group B Internet users without e‐mail reminders and group C Internet users with e‐mail reminders. Non Internet users were followed as usual. Patients in the Internet group users were randomized to standard followup appointments plus periodic e‐mail reminders or just to regular follow‐up consultations Adherence was determined at the beginning of the study and at six and twelve months RESULTS: From June 2011 to November 2011, 168 patients were included (79% F) and followed for one year. 86 had ulcerative colitis, 79 Crohn's disease and 3 indeterminate colitis. 131 patients (78%) were Internet users and 37 not users. 61 Internet users were randomized to the e‐mail remainder group. Initial global adherence was 34.3% in group A 27.8 % in group B and 26.2% in group C (p=n.s). At the end of the study, global adherence rates were 83.3% in group A, 71.8 % in group B and 73.2 % in group C (p=n.s). When analyzing the subtype of non‐adherence we did not find either any difference between groups. There were no differences between groups regarding complications, need to change to steroids or biological therapy, surgery, hospitalizations or any other complication. CDAI Mayo and quality of life scores were similar between the three groups at the end of the follow‐up. CONCLUSIONS: The use of e‐mail reminders do not improve adherence to medical treatment in patients with IBD when compared with standard follow‐up. Regular follow up with appropriate medical information seems to be enough to improve the adherence rate in IBD patients.

%Z Gastroenterology

Journal article; Conference proceeding

%U https://cochrane.66557.net/central/doi/10.1002/central/CN-01026051/full

%0 Journal Article

%A Hernandez Camba, A.

%A Carrillo, M.

%A Ramos, L.

%A Alonso, I.

%A Hernandez Alvarez-Buylla, N.

%A Alarcon Fernandez, O.

%A Jimenez Sosa, A.

%A Quintero, E.

%D 2013

%T E-mail reminders and adherence rates to medical treatment in patients with inflammatory bowel disease

%V 7

%P S112

%8 2013-01-01

%K *colitis; *enteritis; *e‐mail; *human; *patient; *therapy; Biological therapy; Consultation; Crohn disease; Diagnosis; Drug therapy; Female; Follow up; Health care cost; Hospital; Hospitalization; Informed consent; Internet; Medical information; Patient compliance; Pregnant woman; Prospective study; Quality of life; Questionnaire; Relapse; Risk; Steroid; Surgery; Ulcerative colitis; biological therapy; colitis; consultation; diagnosis; drug therapy; enteritis; e‐mail; female; follow up; health care cost; hospital; hospitalization; human; informed consent; medical information; patient; patient compliance; pregnant woman; prospective study; quality of life; questionnaire; relapse; risk; surgery; therapy; ulcerative colitis

%X Background: Non‐adherence to medical treatment in patients with inflammatory bowel disease (IBD) is a well known fact and it is associated with an increased risk of relapse and health care costs. Usefulness of e‐mail reminders to improve patient compliance has not been reported previously. Methods: We performed a prospective study at the Hospital Universitario de Canarias. Patients with IBD diagnosed at least 6 months before, 18 y or older were included. We excluded patients on steroids, pregnant women and those unable to sign the informed consent. Demographic data, including Internet use, were registered. Medical data (years from diagnosis, type and extension of IBD, medication, related surgery, Mayo or CDAI index and a quality of life questionnaire) were also recorded. Adherence to medical treatment was determined with a previously validated questionnaire. We included three groups of patients: group A Internet non users, group B Internet users without e‐mail reminders and group C Internet users with e‐mail reminders. Non Internet users were followed as usual. Patients in the Internet group users were randomized to standard follow‐up appointments plus periodic e‐mail reminders or just to regular follow‐up consultations. Adherence was determined at the beginning of the study and at six and twelve months. Results: From June 2011 to November 2011, 168 patients were included (79% F) and followed for one year. 86 had ulcerative colitis, 79 Crohn's disease and 3 indeterminate colitis. 131 patients (78%) were Internet users and 37 not users. 61 Internet users were randomized to the e‐mail remainder group. Initial global adherence was 34.3% in group A, 27.8% in group B and 26.2% in group C (p = n.s). At the end of the study, global adherence rates were 83.3% in group A, 71.8% in group B and 73.2% in group C (p = n.s). When analyzing the subtype of non‐adherence we did not find either any difference between groups. There were no differences between groups regarding complications, need to change to steroids or biological therapy, surgery, hospitalizations or any other complication. CDAI, Mayo and quality of life scores were similar between the three groups at the end of the follow‐up. Conclusions: The use of e‐mail reminders do not improve adherence to medical treatment in patients with IBD when compared with standard follow‐up. Regular follow up with appropriate medical information seems to be enough to improve the adherence rate in IBD patients.

%Z Journal of Crohn's & colitis

Journal article; Conference proceeding

%U https://cochrane.66557.net/central/doi/10.1002/central/CN-01028536/full

%0 Journal Article

%A Duan, M.

%A Cao, L.

%A Zhu, W.

%A Li, Y.

%D 2022

%T EFFICACY OF TOTAL ENTERAL NUTRITION COMBINED WITH AZATHIOPRINE IN MAINTAINING POSTOPERATIVE REMISSION IN CROHN'S DISEASE: a PARALLEL, RANDOMIZED CONTROLLED STUDY

%V 162

%N 7

%P S‐1084‐S‐1085

%8 2022-01-01

%R 10.1016/S0016-5085(22)62601-5

%K *Crohn disease; *enteric feeding; *postoperative complication; *remission; Adult; Anastomosis; Complication; Conference abstract; Controlled study; Feces; Female; Human; Inflammatory bowel disease; Intestine resection; Major clinical study; Male; Nutrition; Outcome assessment; Prospective study; Questionnaire; Randomized controlled trial; Recurrence risk; Recurrent disease; Side effect; Surgery

%X Objective: The incidence of postoperative recurrence of Crohn's disease (CD) remains high. To investigate whether postoperative exclusive enteral nutrition (EEN) could delay CD recurrence. Methods: CD patients who underwent intestinal resection and anastomosis were randomly divided into nutritional group (EEN 3 months immediately after operation) and control group (from EEN to normal diet within 1 month after operation). All patients received AZA (1.5‐2.5mg/kg/d) about 2 weeks postoperatively. The primary outcome was 1‐year postoperative endoscopic recurrence rate, and the secondary outcomes were 1‐year postoperative clinical recurrence rate, 3‐month postoperative endoscopic recurrence rate, fecal calprotectin (FC), Inflammatory Bowel Disease Questionnaire (IBDQ). Results: A total of 51 patients were included in the analysis. There was no difference in baseline data between the control group (n=26) and the nutritional group (n=25). 10 patients without primary outcome were excluded from the further analysis. The intent to treat analysis showed that the 1‐year endoscopic recurrence rate in the nutritional group was lower than that in the control group, but the difference was not statistically significant [33.3%(7/21) VS 60.0%(12/ 20), p=0.12]. The IBDQ score of the nutrition group was significantly lower than that of the control group 3 months after surgery (165.6±10.8 VS 197.3±6.9, p =0.02). In the perprotocol analysis, the 1‐year endoscopic recurrence rate in the nutrition group was lower than that in the control group, but the difference was not statistically significant [22.2%(4/ 18) VS 58.3%(7/12), p=0.06]. The IBDQ score of nutrition group was significantly lower than that of control group at 1 and 3 months after operation (174.8±6.2 VS 198.1±6.0, p= 0.02; 165.6±10.8 VS 206.8±6.7, p<0.01). In the further analysis, subjects were divided into two groups according to whether the time of postoperative EEN was over 3 months: the 1‐ month EEN group (n=19, average EEN time was 1.6 months) and the 4‐month EEN group (n=22, average EEN time was 4.4 months). Results showed that the 1‐year endoscopic recurrence rate of the 4‐month EEN group was significantly lower than that in the 1‐month EEN group [27.3%(6/22) VS 68.4%(13/19), p =0.02]. The IBDQ score of the 4‐month EEN group at 3 months after surgery was significantly lower than that of the 1‐month EEN group (169.6±9.1 VS 200.7±8.3, p=0.02), whereas no significant difference in IBDQ score between the two groups was observed at 1 year after surgery. 22 patients had adverse events (all were AZA side effects) and no serious adverse events happened. Conclusion: Over 3 months EEN following bowel resection and anastomosis may be associated with a decreased risk of postoperative recurrence of CD. Further prospective studies with large number of patients are warranted. (Table Presented) (Table Presented)

%Z Gastroenterology

Journal article; Conference proceeding

%U https://cochrane.66557.net/central/doi/10.1002/central/CN-02427374/full

%0 Journal Article

%A De Cruz, P.

%A Kamm, M. A.

%A Hamilton, A. L.

%A Ritchie, K. J.

%A Krejany, E. O.

%A Gorelik, A.

%A Liew, D.

%A Prideaux, L.

%A Lawrance, I. C.

%A Andrews, J. M.

%A Et, Al.

%D 2015

%T Efficacy of thiopurines and adalimumab in preventing Crohn's disease recurrence in high-risk patients - a POCER study analysis

%V 42

%N 7

%P 867‐879

%8 2015-01-01

%R 10.1111/apt.13353

%K Adalimumab [*therapeutic use]; Adult; Aged; Azathioprine [*administration & dosage, adverse effects]; Colonoscopy [methods]; Crohn Disease [diagnosis, *prevention & control, *surgery]; Drug Therapy, Combination; Female; Follow‐Up Studies; Humans; Male; Mercaptopurine [*administration & dosage, adverse effects]; Metronidazole [*administration & dosage, adverse effects]; Middle Aged; Postoperative Period; Recurrence; Risk Factors; Treatment Outcome; Tumor Necrosis Factor‐alpha [antagonists & inhibitors, immunology]

%X BACKGROUND: Crohn's disease recurs in the majority of patients after intestinal resection. AIM: To compare the relative efficacy of thiopurines and anti‐TNF therapy in patients at high risk of disease recurrence. METHODS: As part of a larger study comparing post‐operative management strategies, patients at high risk of recurrence (smoker, perforating disease, ≥2nd operation) were treated after resection of all macroscopic disease with 3 months metronidazole together with either azathioprine 2 mg/kg/day or mercaptopurine 1.5 mg/kg/day. Thiopurine‐intolerant patients received adalimumab induction then 40 mg fortnightly. Patients underwent colonoscopy at 6 months with endoscopic recurrence assessed blind to treatment. RESULTS: A total of 101 patients [50% male; median (IQR) age 36 (25‐46) years] were included. There were no differences in disease history between thiopurine‐ and adalimumab‐treated patients. Fifteen patients withdrew prior to 6 months, five due to symptom recurrence (of whom four were colonoscoped). Endoscopic recurrence (Rutgeerts score i2‐i4) occurred in 33 of 73 (45%) thiopurine vs. 6 of 28 (21%) adalimumab‐treated patients [intention‐to‐treat (ITT); P = 0.028] or 24 of 62 (39%) vs. 3 of 24 (13%) respectively [per‐protocol analysis (PPA); P = 0.020]. Complete mucosal endoscopic normality (Rutgeerts i0) occurred in 17/73 (23%) vs. 15/28 (54%) (ITT; P = 0.003) and in 27% vs. 63% (PPA; P = 0.002). The most advanced disease (Rutgeerts i3 and i4) occurred in 8% vs. 4% (thiopurine vs. adalimumab). CONCLUSIONS: In Crohn's disease patients at high risk of post‐operative recurrence adalimumab is superior to thiopurines in preventing early disease recurrence.

%Z Alimentary pharmacology & therapeutics

Journal article

%U https://cochrane.66557.net/central/doi/10.1002/central/CN-01170110/full

%0 Journal Article

%A Wegner, A.

%A Mierzwa, G.

%A Wiecek, S.

%A Korlatowicz- Bilar, A.

%A Dadalski, M.

%A Korczowski, B.

%A Kierkus, J.

%D 2016

%T Efficacy of a micronised, dispersible ferric pyrophosphate in children with anemia associated with inflammatory bowel disease

%V 62

%P 315‐316

%8 2016-01-01

%R 10.1097/01.mpg.0000484500.48517.e7

%K *European; *anemia; *child; *ferric pyrophosphate; *gastroenterology; *human; *inflammatory bowel disease; *nutrition; *society; Blood analysis; Boy; Constipation; Crohn disease; Disease activity; European; Female; Ferritin; Girl; Hematocrit; Hemoglobin; Inflammation; Intestinal tissue; Iron; Iron deficiency; Iron deficiency anemia; Male; Multicenter study; Nausea; Parameters; Patient; Relapse; Risk; Side effect; Statistical significance; Supplementation; Therapy; Ulcerative colitis; anemia; blood analysis; boy; child; constipation; disease activity; female; gastroenterology; girl; hematocrit; human; inflammation; inflammatory bowel disease; intestine tissue; iron deficiency; iron deficiency anemia; male; multicenter study; nausea; nutrition; parameters; patient; relapse; risk; side effect; society; statistical significance; supplementation; therapy; ulcerative colitis

%X Objectives and study: Anemia is one of the most frequent complications of inflammatory bowel disease (IBD) in children. Iron deficiency is the most important cause of anemia in Crohn's disease and ulcerative colitis patients. Oral iron supplementation has been the main form of the treatment for anemia in IBD. However, this treatment is associated with the high risk of side effects. In addition to that, oral iron may exacerbate the inflammation of the intestinal tissues. The aim of the study was to assess the therapy efficacy and the frequency of side effects in patients with the anemia during IBD, treated the ferric pyrophosphate. Methods: A prospective, randomized, multicenter trial was conducted in a group of 37 children (15 girls, 21 boys) aged 13.54±2.7 years (mean±SD), with inflammatory bowel disease (19 Crohn Disease, 18 ulcerative colitis) and the iron deficiency anemia. The patients were randomly assigned to start treatment with the micronised, dispersible ferric pyrophosphate in the dose 60 mg (group 1) or micronised, dispersible ferric pyrophosphate in the dose 120 mg (group 2) for a period of 90 days. Clinical disease activity assessment and blood analysis were performed on days 0 and 90 of the trial. Results: 34 /37 patients completed the study. During the trial the hemoglobin, hematocrit, ferritin and TIBC scores were increased in both groups with statistical significance (p<0.05). The iron level was increased with statistical significance in group 2 but not in group 1. There were no statistical significant differences in comparison this parameters from group 1 and 2 in the 90 day of the study. During the trial there were no SAE. 13 patients from group 1 reported the side effects ( 7‐ stomachache, 3‐ nauseas, 2‐ constipation). In group 2 ‐ 7 patients reported the side effects ( 3‐ stomachache, 3‐ nauseas, 1‐ constipation). The difference between groups was not statistically significant. 3 patients didn't complete the trial. Reasons for not completing the study were: withdrawal of consent (1 patient from group 2), relapse of the disease and stopped taking ferric pyrophosphate ( 1 patient from group 1, 1 patient from group 2). (Table presented) Conclusion: Ferric pyrophosphate is effective and safe in children with IBD anemia.

%Z Journal of pediatric gastroenterology and nutrition

Journal article; Conference proceeding

%U https://cochrane.66557.net/central/doi/10.1002/central/CN-01266629/full

%0 Journal Article

%A Wegner, A.

%A Mierzwa, G.

%A Wiecek, S.

%A Korlatowicz-Bilar, A.

%A Dadalski, M.

%A Korczowski, B.

%A Kierkus, J.

%D 2016

%T Efficacy of a micronised, dispersible ferric pyrophosphate in children with anaemia associated with inflammatory bowel disease

%V 10

%P S286‐S287

%8 2016-01-01

%R 10.1093/ecco-jcc/jjw019

%K *Crohn disease; *iron deficiency anemia; Adolescent; Adverse drug reaction; Blood analysis; Child; Clinical article; Clinical trial; Comparative effectiveness; Constipation; Controlled clinical trial; Controlled study; Drug therapy; Drug withdrawal; Female; Girl; Hematocrit; Human; Human tissue; Intestine tissue; Male; Multicenter study; Nausea; Randomized controlled trial; Relapse; Side effect; Statistical significance; Stomach pain; Ulcerative colitis

%X Background: Anaemia is one of the most frequent complications of inflammatory bowel disease (IBD) in children. Iron deficiency is the most important cause of anaemia in Crohn's disease and ulcerative colitis patients. Oral iron supplementation is the main form of the treatment for anaemia in IBD. However, this treatment is associated with the high risk of side effects. In addition to that, oral iron may exacerbate the inflammation of the intestinal tissues. Methods: The aim of the study was to assess the therapy efficacy and the frequency of side effects in patients with the anaemia during IBD, treated the ferric pyrophosphate. A prospective, randomised, multicentre trial was conducted in a group of 37 children (15 girls and 21 boys) aged 13.54 ± 2.7 years (mean ± SD), with inflammatory bowel disease (19 Crohn's disease and18 ulcerative colitis) and iron deficiency anaemia. The patients were randomly assigned to start treatment with the micronised, dispersible ferric pyrophosphate in the dose 60 mg (group 1) or micronised, dispersible ferric pyrophosphate in the dose 120 mg (group 2) for a period of 90 days. Clinical disease activity assessment and blood analysis were performed on days 0 and 90 of the trial. Results: In total, 34/37 patients completed the study. During the trial the haemoglobin, haematocrit, ferritin and TIBC scores were increased in both groups with statistical significance (p < 0.05). The iron level was increased with statistical significance in group 2 but not in group 1. There were no statistical significant differences in comparison this parameters from group 1 and 2 in the 90 trail day. During the trial there were no SAE. 13 patients from group 1 reported the side effects (7 stomach ache; 3 nauseas; and 2 constipation). In group 2, 7 patients reported the side effects (3 stomach ache; 3 nauseas; and 1 constipation). The difference between groups was not statistically significant. Further, 3 patients did not complete the trial. Reasons for not completing the study were withdrawal of consent (1 patient from group 2), relapse of the disease, and cessation of ferric pyrophosphate (1 patient from group 1 and 1 patient from group 2). Conclusions: Ferric pyrophosphate is effective and safe in children with IBD anaemia. (Figure Presented).

%Z Journal of Crohn's & colitis

Journal article; Conference proceeding

%U https://cochrane.66557.net/central/doi/10.1002/central/CN-01766490/full

%0 Journal Article

%A Kierkus, J.

%A Iwanczyk, B.

%A Wegner, A.

%A Dadalski, M.

%A Grzybowska-Chlebowczyk, U.

%A Lazowska, I.

%A Maslana, J.

%A Toporowska-Kowalska, E.

%A Czaja-Bulsa, G.

%A Mierzwa, G.

%A Et, Al.

%D 2013

%T Efficacy infliximab with immunomodulator and infliximab alone of maintenance therapy in children with Crohn's disease multicenter randomized study

%V 7

%P S220‐S221

%8 2013-01-01

%K *Crohn disease; *child; *colitis; *human; *immunomodulating agent; *infliximab; *maintenance therapy; Boy; Crohn disease; Deterioration; Female; Girl; Male; Patient; Registration; Relapse; Remission; Risk; Side effect; Therapy; boy; child; colitis; deterioration; female; girl; human; maintenance therapy; male; patient; registration; relapse; remission; risk; side effect; therapy

%X Background: Optimal use of biologics in Crohn's disease (CD) requires balance between benefits and risk, and concern of serious side effects may question the concomitant immunomodulators use in pediatric patients. The study (registration no NCT01559142) was conducted to compare the efficacy of two infliximab maintenance regiments: (1) infliximab alone and (2) infliximab with immunomodulator in pediatric patients with moderate to severe active CD. Methods: Ninety‐nine children (62 boys and 37 girls) aged 14.54±2.61 years with PCDAI >30 pts and endoscopic evaluation performed were involved to the study and received induction therapy with infliximab 5 mg/kg at weeks 0, 2, and 6. Clinical (PCDAI score) and endoscopical (SES CD score) evaluations were performed at week 10 and patients with clinical response (decrease of PCDAI ≥15 AND PCDAI <30) and/or remission (PCDAI ≤10) were randomized to Group l receiving infliximab 5 mg/kg every 8 weeks with immunomodulator until Week 54 (n = 45) or Group II receiving infliximab 5 mg/kg every 8 weeks with immunomodulator stopped at Week 26 (n = 39). Clinical (PCDAI score) and endoscopical (SES CD score) were performed at week 54 in both groups. Primary endpoint was loss of clinical response defined as increase of PCDAI >15 points or PCDAI >30. Secondary endpoints were: necessity to increase/ change maintenance therapy and relapse of mucosal response defined as increase of SES CD score. Results: 84 out of 99 (85%) pts had response, and 58 (59%) clinical remission. 78 out of 84 (93%) pts with clinical response had also mucosal response defined as decrease of SES CD score and 26 out of 84 (31%) mucosal remission defined as SES CD = 0 points. 2 out of 45 (4%) pts in Group I and 2 out of 39 pts (5%) in Group II had loss of clinical response at Week 54 (non significant NS). The increase/change of maintenance therapy was necessary in 13 out of 45 pts (29%) in Group I and 11 out of 39 (28%) in Group II (NS). Mucosal deterioration (increase of SES CD score) was found in 13 out of 45 pts in Group I (29%) and 11 out of 39 pts in Group II (28%) (NS). Conclusions: Both regimens of infliximab maintenance therapy ‐ with and without concomitant immunomodulator are ‐ equally efficient in the clinical and endoskcopic response rate in children with moderate to severe active CD.

%Z Journal of Crohn's & colitis

Journal article; Conference proceeding

%U https://cochrane.66557.net/central/doi/10.1002/central/CN-01028529/full

%0 Journal Article

%A Kim, K. O.

%A Jang, B. I.

%A Lee, Y. J.

%A Lee, H. S.

%A Kim, E. S.

%A Jung, Y. J.

%A Kim, E. Y.

%A Kim, S. K.

%A Yang, C. H.

%D 2022

%T Efficacy and safety of oral sulfate tablet for the bowel preparation in patients with inflammatory bowel disease: multicenter randomized controlled study

%V 16

%P i282‐i283

%8 2022-01-01

%R 10.1093/ecco-jcc/jjab232.360

%K *drug safety; *drug tolerability; *inflammatory bowel disease; *intestine preparation; Adult; Aphthous stomatitis; Bloating; Cleaning; Clinical trial; Colonoscopy; Comparative effectiveness; Conference abstract; Disease exacerbation; Drug dose regimen; Drug therapy; Electrolyte blood level; Endoscopist; Female; Human; Human tissue; Interview; Kidney function; Laboratory test; Major clinical study; Male; Mucosa; Multicenter study; Randomized controlled trial; Recurrent disease; Satisfaction; Single blind procedure; Visual analog scale; Vomiting

%X Background: Although high and low volume polyethylene glycol(PEG) bowel preparations are recommended for patients with inflammatory bowel disease(IBD) before colonoscopy, sometime it is limited due to poor compliance. So, studies regarding novel low volume preparations in IBD are need, but, there is limited data. We aimed to evaluated the safety, tolerability and efficacy of oral sulfate tablet (OST) by comparison with 2L PEG and ascorbate (PEG/Asc) in patients with IBD. Methods: One hundred ten patients with IBD were enrolled in this single‐blind, multicenter, non‐inferiority study. Patients were assigned either 2L PEG/Asc or OST to be administered in a split‐dose regimen. OST patients were administered 14 tablets with more than 1L of free water in the evening, and the following morning. Colonoscopies were performed by blinded investigators. The primary efficacy endpoint was successful bowel cleansing rate, defined as Harefield Cleansing Scale grade A or B as evaluated by blinded endoscopist in each hospital. Secondary endpoints included the presence of residual air bubbles. Adverse events and laboratory evaluations were monitored to assess safety. Flare up of IBD symptoms and mucosa change associated with preparation were also evaluated. Tolerability, in terms of taste and degree of resistance to drink was assessed with VAS score by interview before colnoscopy. Safety was assessed by spontaneously reported adverse events, solicited ratings of expected prep symptoms, and laboratory testing. Results: The cleansing score at all the segment was significantly higher in OST group. A high rate of cleansing success was seen with OST (98.1%), which was noninferior to 2L PEG/Asc (98.1%). Proportion of patients with bubble score 0 was significantly higher in OST than 2L PEPG/Asc group (94.5% vs. 50.0%, p<0.001). Satisfaction, in terms of easiness to drink (8.2 ± 1.6 vs. 6.0 ± 2.2, p<0.001) and taste(7.0 ± 2.8 vs. 5.4 ± 2.4, p=0.002) was significantly higher in OST group. More patients in OST group (94.5%) wanted to repeat the same preparation next colonoscopy. There was no significant difference in the development of vomiting and bloating between group. Aphthous ulcers were noted in the same number of patients in both group and symptom flare up were noted in 2 cases of OST group (p=1.000). No clinically significant differences were seen between preps for renal function and serum electrolyte. Conclusion: Oral sulfate tablets achieved a high level of cleansing comparable with 2L PEG/Asc in patients with IBD. The risk of mucosal change or symptom relapse were not higher, on the other hand the degree of satisfaction was higher than 2L PEG/Asc. OST split method could be considered as one of the preparation method for patients with inactive IBD.

%Z Journal of Crohn's & colitis

Journal article; Conference proceeding

%U https://cochrane.66557.net/central/doi/10.1002/central/CN-02374217/full

%0 Journal Article

%A Fan, H.

%A Du, J.

%A Liu, X.

%A Zheng, W. W.

%A Zhuang, Z. H.

%A Wang, C. D.

%A Gao, R.

%D 2019

%T Effects of pentasa-combined probiotics on the microflora structure and prognosis of patients with inflammatory bowel disease

%V 30

%N 8

%P 680‐685

%8 2019-01-01

%R 10.5152/tjg.2019.18426

%K *inflammatory bowel disease; *microflora; *prognosis; Adult; Anti‐Inflammatory Agents, Non‐Steroidal [*therapeutic use]; Article; Bacteroides; Bifidobacterium; Clinical article; Controlled study; Cytokines [analysis]; Drug Therapy, Combination; Drug combination; Drug therapy; Enterobacteriaceae; Feces; Feces [chemistry]; Female; Gastrointestinal Microbiome [*drug effects]; Gene expression; Human; Humans; Inflammatory Bowel Diseases [*drug therapy, *microbiology]; Lactobacillus; Male; Mesalamine [*therapeutic use]; Middle Aged; Nonhuman; Probiotics [*therapeutic use]; Prognosis; Protein expression; Randomized controlled trial; Recurrence risk; Saccharomyces; Treatment Outcome; Validation process

%X BACKGROUND/AIMS: The aim of the present study was to investigate the effects of the combination treatment of pentasa and probiotics on the microflora composition and prognosis in patients with inflammatory bowel disease (IBD). MATERIALS AND METHODS: A total of 40 patients with IBD (19 control group and 21 observation group) were randomized. Patients in the control group were given pentasa, and patients in the observation group were given probiotics along with pentasa. The microflora composition, biochemical indices, inflammatory markers, and activity scores of the two groups were analyzed. RESULTS: After treatment, the number of enterobacteria, enterococci, saccharomyces, and bacteroides; the levels of fecal lactoferrin, 1‐antitrypsin, β2‐microglobulin, high‐sensitivity C‐reactive protein, and interleukin (IL)‐6; activity scores; and recurrence rate in the observation group were significantly lower than those in the control group. Bifidobacterium and lactobacillus counts and IL‐4 levels were significantly higher in the observation group than in the control group. CONCLUSION: The combination of probiotics and pentasa can improve microflora composition in patients with IBD and reduce the level of inflammatory cytokines; therefore, it is worthy of further clinical validation.

%Z Turkish journal of gastroenterology

Journal article

%U https://cochrane.66557.net/central/doi/10.1002/central/CN-01986709/full

%0 Journal Article

%A NCT

%D 2023

%T Effectiveness of Telemedicine in Inflammatory Bowel Disease

%8 2023-01-01

%K Crohn Disease; Inflammatory Bowel Diseases; Intestinal Diseases

%X Number of volunteers: 60 patients with IBD Study duration: 1 year (6 months ‐ patient enrollment, 6 months ‐ telemedical intervention). Inclusion criteria: ‐ Age ≥18 years old. ‐ Signed informed consent. ‐ Diagnosis: Crohn disease (ICD‐10 codes K50.0, K50.1, K50.8, K50.9) and Ulcerative colitis (K51.0, K51.2, 51.3, K51.5, K51.8). Exclusion criteria: ‐ Severe cognitive dysfunction; ‐ Acute mental illness; ‐ Lack of technical ability to take part in telemedical intervention (does not have the skills to work with a smartphone, computer, tablet, there is no appropriate technical means); ‐ Participation in other clinical studies. ‐ Oncological diseases requiring active treatment. ‐ Patients who denied signing informed consent. Outpatient observation with connection to the telemonitoring program ‐ 30 participants. Outpatient monitoring without connecting to the telemonitoring program ‐ 30 participants. Study design: Stage 1: selection of the patients with IBD that meet the inclusion criteria after inpatient treatment in the gastroenterology department. Randomization of participants into two groups: face‐to‐face outpatient observation and observation using telemedicine technologies. Signing informed consent, filling out questionnaires: 1. Simple Clinical Colitis Activity Index (SCCAI) questionnaire for patients with ulcerative colitis / Harvey‐Bradshaw index (HBI) questionnaire for patients with Crohn disease; 2. World Health Organization's Quality of Life (WHOQOL‐26): questionnaire for assessing health‐related quality of life; 3. SIBDQ: Short Inflammatory Bowel Disease Questionnaire; 4. Work Productivity and Activity Impairment Questionnaire (WPAI): questionnaire used to evaluate work productivity and activity impairment; 5. Hospital Anxiety and Depression Scale (HADS); 6. Toronto Alexithymia Scale (TAS‐26): used to evaluate alexithymia (a risk factor for the development of psychosomatic diseases); 7. Visceral Sensitivity Index (VSI): Special gastroenterological questionnaire to determine the index of visceral sensitivity; 8. PSQ‐18: Patient Satisfaction Questionnaire; 9. General Medication Adherence Scale (GMAS) to determine the adherence to the therapy. Stage 2: for the outpatient observation group: one face‐to‐face appointment, general recommendations. For the telemonitoring group patients log in to the web platform at least once a month. When the patient log in to the telemedicine platform the following scorings must be filled out:1) SCCAI for Ulcerative colitis and Harvey‐Bradshaw index for Crohn disease. 2) IBD disk questionnaire to evaluate disease dynamics. The results of the scoring systems will appear to the health care professionals and patients in a traffic light manner (red, yellow and green). If patient has a recurrence of the disease, he will be instructed to contact a doctor. Patients from intervention group will have the possibility of online consultation with a gastroenterologist upon request via chat or phone call and access to educational information about IBD, necessary lifestyle changes, dietary recommendations. To be assessed in both groups during the entire observation period: ‐ Frequency of scheduled and unscheduled visits, hospitalizations in medical organizations, assistance within the statutory health insurance system; ‐ The number of surgical interventions associated with IBD; ‐ Activity impairment and work productivity; ‐ Mortality from complications of IBD; ‐ State and patient spending on healthcare; Stage 3: re‐hospitalization of both groups 6 months after randomization in order to evaluate IBD activity (laboratory and instrumental examinations). Re‐completion of all questionnaires for both groups. The purpose of this study is to evaluate telemedicine impact in IBD.

%Z https://clinicaltrials.gov/ct2/show/NCT05994716

Trial registry record; Clinical trial protocol

%U https://cochrane.66557.net/central/doi/10.1002/central/CN-02590060/full

%0 Journal Article

%A NCT

%D 2014

%T Effect of Soluble Dietary Fiber on Bacterial Translocation in Crohn's Disease

%8 2014-01-01

%K Crohn Disease; Pectin

%X Bacterial translocation (BT) is a proposed mechanism of CD. Microorganisms can be cultured from 18‐48% of draining mesenteric lymph nodes from CD patients. A breakdown in barrier function in "late stage" CD has been observed in patients requiring surgery. In addition, it was found that BT influences the response to biological therapy and clinical relapse in CD. Therefore, reducing BT may be of therapeutic importance in treatment for CD. The role of soluble dietary fiber in Crohn's disease (CD) is still inconclusive. Population based studies have shown that long‐term intake of dietary fiber is associated with lower risk of CD. However, meta‐analysis did not show benefit in inducing or maintaining remission. In addition, the possible mechanism of dietary fiber on CD is still unclear. The rationale relates to the beneficial effects of fiber may be due to the production of the fiber metabolites short‐chain fatty acids (SCFAs), particularly butyrate. Dietary substrates may modify the commensal microbiota or their metabolites or enhance epithelial barrier function. Recently, it was found that dietary fiber metabolites SCFA is regulatory of mucosal regulatory T cells. The current study is to examine the impact of dietary fiber on bacterial translocation,intestinal luminal microbiology, and mucosal immunology in CD patients.

%Z https://clinicaltrials.gov/show/NCT02164877

Trial registry record

%U https://cochrane.66557.net/central/doi/10.1002/central/CN-01546459/full

%0 Journal Article

%A NCT

%D 2016

%T Early Surgery Versus Conservative Treatment in Patients With Ileocaecal Crohn's Disease

%8 2016-01-01

%K Crohn Disease

%X Surgical therapy is currently indicated for Crohn's disease (CD) patients after conservative treatment becomes ineffective. The principles of so‐called step‐up therapy (STUP) where surgery represents the last therapeutical option are still followed. Early surgical intervention (Early Surgery ‐ ES) can be an alternative even in patients with uncomplicated type of CD before all medical therapy is used (Top‐down approach). Limited resection under these conditions will lead to immediate remission. Moreover, laparoscopic ileocaecal resection is safe with low morbidity and regarding potential complications of step‐up treatment might be beneficial for the patient. Before wide introduction of ES approach into clinical practice, it is necessary to perform a randomized trial comparing early resection with the standard step‐up medical therapy. The potential effect of early, intensive therapy (ileocaecal resection) on biological behavior of the disease has not been studied that is why patients with uncomplicated ileocaecal form are the most suitable for such a trial. Significant number of these patients will indeed progress into more unfavorable course of the disease (relapse, complicated form, early recurrence). Other potential benefit of early resection is the extended period without necessary medication. Even pharmacological recurrence prevention is not needed after surgery in uncomplicated CD patient if other risk factors are excluded. Rapid remission induced by surgery can lead to faster improvement of quality of life than long‐term medication.

%Z https://clinicaltrials.gov/show/NCT02716454

Trial registry record

%U https://cochrane.66557.net/central/doi/10.1002/central/CN-01556770/full

%0 Journal Article

%A Scapa, E.

%A Maharshak, N.

%A Kariv, Y.

%A Ben-Horin, S.

%A White, I.

%A Santo, E.

%A Tulchinsky, H.

%A Dotan, I.

%D 2015

%T Early initiation of adalimumab significantly diminishes post-operative Crohn's Disease recurrence, and is superior to immunomodulator therapy. Preliminary results from the POPART trial

%V 9

%P S303‐S304

%8 2015-01-01

%R 10.1093/ecco-jcc/jju027

%K *European; *adalimumab; *colitis; *immunomodulating agent; *organization; *recurrent disease; *therapy; Arm; C reactive protein; Calgranulin; Crohn disease; Donkey; European; Follow up; History; Human; Intestine resection; Mercaptopurine; Patient; Placebo; Population; Prevention; Quality of life; Remission; Risk; Smoking; Stratification; Tumor necrosis factor inhibitor; Ulcer; Weight; arm; colitis; donkey; follow up; history; human; intestine resection; organization; patient; population; prevention; quality of life; recurrent disease; remission; risk; smoking; stratification; therapy; ulcer; weight

%X Background: A sizable proportion of Crohn's disease (CD) patients will undergo intestinal resection, but most will experience early endoscopic and clinical disease recurrence. Prevention of post‐operative recurrence using a risk‐stratified approach was recently demonstrated to be superior when anti‐TNF agents were compared to placebo and immunomodulators, but the value of early anti‐TNF in an all‐comers patients population has not been examined to date. Aim: To evaluate the efficacy of an early unstratified approach comparing anti‐TNF (adalimumab) and thiopurine (6‐mercap‐topurine, 6MP) therapy on the natural history of post operative CD recurrence. Aim: To evaluate the efficacy of an early unstratified approach comparing anti‐TNF (adalimumab) and thiopurine (6‐mercaptopurine, 6MP) therapy on the natural history of post operative CD recurrence. Methods: All CD patients undergoing a first ileocecectomy for inflammatory complications were prospectively recruited to the Post OPerative Adalimumab Recurrence Trial (POPART). Patients were randomized within 45 days to receive either adalimumab 160mg/80mg and then 40 mg every other week, or 6MP 1.5mg/kg/day. All patients underwent ileocolonoscopy at 6 and 12 months to asses for endoscopic recurrence as defined by the Rutgeert's score. Endoscopic remission was defined as a Rutgeert's score of i0‐i1, while advanced lesions were defined as i2‐i4. Results: Nineteen patients have reached the 24 week time point. The mean ages of patients in the 6MP (n=8) and adalimumab (n=11) arms (30.5 ± 2.3 vs 34.4 ± 2.5 years, respectively) as well as the smoking status (1/11 vs3/8smokers/total) were comparable. Fifty percent (4/8) of the 6MP‐treated patients had advanced lesions (i2), compared to only 9% (1 of 11) of the adalimumab‐treated patients (p<0.05). Furthermore median fecal calprotectin levels were 186 in the 6MP (n=6) arm and 27 in the adalimumab arm (n=3), and adali‐mumab‐treated patients gained more weight (6.5kg vs 3.5kg increase in the 6MP group, p=0.06) during the 24 weeks follow‐up. CD activity index, histological scores, C‐reactive protein, and quality of life as represented by the IBDQ score were comparable. Importantly, a Rutgeerts score of i2 was the most advanced score recorded in both groups, mostly due to discrete perianastomotic ulcers. Conclusions: Early treatment of post operative CD patients with adalimumab is significantly superior to 6MP, regardless of risk stratification. Applying such therapy may modify the natural history of post‐operative CD.

%Z Journal of Crohn's & colitis

Journal article; Conference proceeding

%U https://cochrane.66557.net/central/doi/10.1002/central/CN-01130625/full

%0 Journal Article

%A Scapa, E.

%A Maharshak, N.

%A Kariv, Y.

%A Ben-Horin, S.

%A White, I. D.

%A Santo, E.

%A Tulchinsky, H.

%A Dotan, I.

%D 2015

%T Early initiation of adalimumab significantly diminishes post-operative crohn's disease recurrence, and is superior to immunomodulator therapy. Preliminary results from the POPART trial

%V 148

%N 4

%P S240‐S241

%8 2015-01-01

%K *adalimumab; *gastrointestinal disease; *immunomodulating agent; *recurrent disease; *therapy; Arm; C reactive protein; Calgranulin; Crohn disease; Donkey; Follow up; History; Human; Intestine resection; Mercaptopurine; Patient; Placebo; Population; Prevention; Quality of life; Remission; Risk; Smoking; Stratification; Tumor necrosis factor inhibitor; Ulcer; Weight; arm; donkey; follow up; gastrointestinal disease; history; human; intestine resection; patient; population; prevention; quality of life; recurrent disease; remission; risk; smoking; stratification; therapy; ulcer; weight

%X Background: A sizable proportion of Crohn's disease (CD) patients will undergo intestinal resection, but most will experience early endoscopic and clinical disease recurrence. Prevention of post‐operative recurrence using a risk‐stratified approach was recently demonstrated to be superior when anti‐TNF agents were compared to placebo and immunomodulators, but the value of early anti‐TNF in an all‐comers patients population has not been examined to date. Aim: To evaluate the efficacy of an early unstratified approach comparing anti‐TNF (adalimumab) and thiopurine (6‐mercaptopurine, 6MP) therapy on the natural history of post operative CD recurrence. Methods: All CD patients undergoing a first ileocecectomy for inflammatory complications were prospectively recruited to the Post OPerative Adalimumab Recurrence Trial (POPART). Patients were randomized within 45 days to receive either adalimumab 160mg/80mg and then 40 mg every other week, or 6MP 1.5mg/kg/day. All patients underwent ileocolonoscopy at 6 and 12 months to asses for endoscopic recurrence as defined by the Rutgeert's score. Endoscopic remission was defined as a Rutgeert's score of i0‐i1, while advanced lesions were defined as i2‐i4. Results: Nineteen patients have reached the 24 week time point. The mean ages of patients in the 6MP (n=8) and adalimumab (n=11) arms (30.5±2.3 vs 34.4±2.5 years, respectively) as well as the smoking status (1/11 vs3/8smokers/total) were comparable. Fifty percent (4/8) of the 6MP‐treated patients had advanced lesions (i2), compared to only 9% (1 of 11) of the adalimumab‐treated patients (p<0.05). Furthermore median fecal calprotectin levels were 186 in the 6MP (n=6) arm and 27 in the adalimumab arm (n=3), and adalimumab‐treated patients gained more weight (6.5kg vs 3.5kg increase in the 6MP group, p=0.06) during the 24 weeks follow‐up. CD activity index, histological scores, C‐reactive protein, and quality of life as represented by the IBDQ score were comparable. Importantly, a Rutgeerts score of i2 was the most advanced score recorded in both groups, mostly due to discrete perianastomotic ulcers. Conclusion: Early treatment of post operative CD patients with adalimumab is significantly superior to 6MP, regardless of risk stratification. Applying such therapy may modify the natural history of post‐operative CD.

%Z Gastroenterology

Journal article; Conference proceeding

%U https://cochrane.66557.net/central/doi/10.1002/central/CN-01089145/full

%0 Journal Article

%A Scapa, E.

%A Fliss-Isakov, N.

%A Tulchinsky, H.

%A Yanai, H.

%A White, I.

%A Dotan, I.

%A Maharshak, N.

%D 2018

%T Early initiation of adalimumab significantly diminishes endoscopic post-operative Crohn's disease recurrence, and is superior to immunomodulatory therapy, regardless of risk stratification. A randomized, controlled study

%V 6

%N 8

%P A458

%8 2018-01-01

%R 10.1177/2050640618792819

%K *Crohn disease; *recurrent disease; *stratification; Adult; Case report; Clinical article; Comparative effectiveness; Complication; Conference abstract; Crohn disease; Drug therapy; Female; Follow up; Human; Ileocolonoscopy; Male; Nonsmoker; Prospective study; Randomized controlled trial; Relapse; Risk assessment; Surgery; adult; case report; clinical article; comparative effectiveness; complication; conference abstract; drug therapy; female; follow up; human; ileocolonoscopy; male; non‐smoker; prospective study; randomized controlled trial; recurrent disease; relapse; risk assessment; stratification; surgery

%X Introduction: A sizable proportion of patients with Crohn's disease (CD) will undergo intestinal resection. Most will experience early post‐operative endoscopic and clinical disease recurrence. The superiority of anti‐TNF agents over both placebo and immunomodulators in the prevention of post‐operative recurrence using a risk‐stratified strategy was previously demonstrated. Aims and Methods: To evaluate the efficacy of an early unstratified approach comparing anti‐TNF (adalimumab) with thiopurine (6‐mercaptopurine, 6MP) therapy on post‐operative CD recurrence, as demonstrated by endoscopy at 24 weeks. All CD patients undergoing a first ileocecectomy for inflammatory complications were prospectively recruited to the Post Operative Adalimumab Recurrence Trial (POPART). Patients were randomized within 45 days to receive either adalimumab or 6MP. All patients underwent ileocolonoscopy at 24 weeks post operation to asses for endoscopic recurrence as defined by the Rutgeerts score. Endoscopic recurrence was defined as a Rutgeerts score of i2‐i4. Results: Fourty‐one patients were recruited to the study. Four patients were lost to follow‐up, and two who did not adhere to treatment were excluded from the final analysis. A total of 35 patients completed the study protocol ‐16 were treated with 6MP and 19 with adalimumab. Mean age 32.2±21.6, 62.9% males, 54.3% non‐smokers. Follow‐up endoscopy was performed at week 24. Baseline clinical parameters and smoking status were comparable between treatment arms (43.8% vs. 38.9% respectively, p=0.774). Endoscopic recurrence at week 24 was significantly lower in the adalimumab vs. the 6MP arm: 21.1% vs 56.2%, p=0.03. Other clinical and biologic indices at week 12 and 24 were comparable (Table 1). Table 1. Clinical and endoscopic parameters of Crohn's disease patients at post ileocecectomy weeks 12 and 24 according to medical therapy Conclusion: Early treatment of post‐operative CD patients with adalimumab is significantly superior to 6MP, regardless of risk stratification. Endoscopy is superior to clinical and biochemical indices, in early risk stratification of patients.

%Z United European gastroenterology journal

Journal article; Conference proceeding

%U https://cochrane.66557.net/central/doi/10.1002/central/CN-01792127/full

%0 Journal Article

%A Molander, P.

%A Färkkilä, M.

%A Ristimäki, A.

%A Salminen, K.

%A Kemppainen, H.

%A Blomster, T.

%A Koskela, R.

%A Jussila, A.

%A Rautiainen, H.

%A Nissinen, M.

%A Et, Al.

%D 2015

%T Does fecal calprotectin predict short-term relapse after stopping TNFα-blocking agents in inflammatory bowel disease patients in deep remission?

%V 9

%N 1

%P 33‐40

%8 2015-01-01

%R 10.1016/j.crohns.2014.06.012

%K Adolescent; Adult; Child; Colonoscopy; Enzyme‐Linked Immunosorbent Assay; Feces [*chemistry]; Female; Follow‐Up Studies; Gastrointestinal Agents [*therapeutic use]; Humans; Inflammatory Bowel Diseases [diagnosis, *drug therapy, metabolism]; Leukocyte L1 Antigen Complex [*analysis]; Male; Middle Aged; Prognosis; Recurrence; Remission Induction; Time Factors; Tumor Necrosis Factor‐alpha [*antagonists & inhibitors]; Young Adult

%X BACKGROUND AND AIMS: This prospective multicenter study examined whether elevated fecal calprotec tin (FC) concentrations after stopping TNFα‐blocking therapy can predict clinical or endoscopic relapse. In addition, we evaluated the impact of histological remission on the relapse risk. METHODS: We enrolled inflammatory bowel disease (IBD) patients who were in clinical, endoscopic, and FC‐based (< 100 μg/g) remission after a minimum 11 months of TNFα‐blocking therapy. The patients were followed‐up for 12 months after the discontinuation of TNFα‐blocking therapy. FC was collected monthly for the first 6 months and thereafter every second month. Ileocolonoscopy was performed at inclusion, at 4 months, at the study end, and at the time of clinical relapse. RESULTS: Of 52 enrolled patients, 49 (16 Crohn's disease, 33 ulcerative colitis/IBD unclassified) provided the stool samples requested and comprised the study group. During the follow‐up, 15/49 (31%) relapsed, whereas 34 (69%) remained in remission. Patients relapsing showed constantly elevated FC levels for a median of 94 (13‐317) days before the relapse. Significant increase in median FC levels was seen 2 (p = 0.0014), 4 (p = 0.0056), and 6 (p = 0.0029) months before endoscopic relapse. Constantly normal FC concentrations during the follow‐up were highly predictive for clinical and endoscopic remission. Normal FC concentrations in patients with remission were associated with histological remission. CONCLUSION: FC seems to increase and remain elevated before clinical or endoscopic relapse, suggesting that it can be used as a surrogate marker for predicting and identifying patients requiring close follow‐up in clinical practice.

%Z Journal of Crohn's & colitis

Journal article

%U https://cochrane.66557.net/central/doi/10.1002/central/CN-01114988/full

%0 Journal Article

%A Miele, E.

%A Giannetti, E.

%A Martinelli, M.

%A Buonavolontà, R.

%A Strisciuglio, C.

%A Sciorio, E.

%A Staiano, A.

%D 2011

%T Does cow's-milk protein elimination diet have a role in induction and maintenance of remission in children with Ulcerative Colitis?

%V 52

%P E151

%8 2011-01-01

%R 10.1097/MPG.0b013e318224e326

%K *child; *diet; *gastroenterology; *human; *milk; *nutrition; *remission; *society; *ulcerative colitis; Allergy; Atopy; Cow; Dairy product; Diagnosis; Disease activity; Enteritis; Etiology; Family history; Follow up; Lactose intolerance; Maintenance therapy; Patient; Physician; Population; Prevalence; Prick test; Questionnaire; Randomized controlled trial; Relapse; Risk; Skin; Therapy

%X Objectives and Study: Children with inflammatory bowel disease (IBD) avoid dairy products more than they would need to based on the prevalence of lactose malabsorption and/or allergy to milk proteins because of arbitrary advice from physicians. Aims of the present study were to evaluate the efficacy of a cow's milk protein elimination diet on induction and maintenance of remission and to define association with atopy in children with ulcerative colitis (UC). Methods: Twenty consecutive patients (mean age: 10 y and 5 months; range: 4 y and 7 months to 17 y; F/M: 11/9) with newly diagnosed UC were randomised either to receive a cow's milk protein elimination diet (n=10) or to continue a free diet (n=10) associated to concomitant steroid induction and mesalamine maintenance treatment. Children were prospectively evaluated at four time points: within 1 month, 2 months, 6 months and 1 year after diagnosis or at the time of relapse. Pediatric Ulcerative Colitis Activity Index (PUCAI) and a physician's global assessment were used to measure disease activity. At baseline and at 12 months or at the time of relapse all patients were assessed endoscopically and histologically. A questionnaire was completed including personal and family history of atopy. At diagnosis, patients were tested for atopy by specific serumIgE and/or skin‐prick tests. Results: All 20 patients responded to the IBD induction therapy. At 6 months remission was achieved in 5 patients (50%) treated with elimination diet and IBD therapy and in 4 patients (40%) treated with free diet and IBD therapy (P=0.31). Preliminary data showed that 5 of 10 (50%) patients treated with elimination diet and IBD therapy and 4 of 10 (40%) patients treated with free diet and IBD therapy relapsed within 6 months of follow‐up (P=0.5; OR=1.5; CI=0.25‐8.81). At time of enrollment and at time of relapse, endoscopic and histological scores were not statistically different comparing the exclusion diet and free diet groups (P=0.45; P=0.68). All 20 patients completed the questionnaire. The prevalence of atopy in our study population was 32%. Four children in the elimination diet group had a diagnosis of atopy compared with 3 patients in the free diet group (P=0.32). Conclusion: Even though allergy to milk protein has been reported to play a possible role in the etiopathogenesis of UC, preliminary data of this first pediatric, randomised, controlled trial suggest that cow's‐milk protein elimination diet does not modify the time of induction and the risk of relapse.

%Z Journal of pediatric gastroenterology and nutrition

Journal article; Conference proceeding

%U https://cochrane.66557.net/central/doi/10.1002/central/CN-01005895/full

%0 Journal Article

%A Martinelli, M.

%A Miele, E.

%A Giannetti, E.

%A Buonavolontà, R.

%A Strisciuglio, C.

%A Staiano, A.

%D 2011

%T Does cow's milk protein elimination diet have a role on induction and maintenance of remission in childrenwith ulcerative colitis?

%V 43

%P S434

%8 2011-01-01

%K *cow; *diet; *remission; *ulcerative colitis; Allergy; Atopy; Child; Dairy product; Diagnosis; Disease activity; Enteritis; Etiology; Family history; Follow up; Human; Lactose intolerance; Maintenance therapy; Patient; Physician; Population; Prevalence; Prick test; Questionnaire; Randomized controlled trial; Relapse; Risk; Serum; Skin; Therapy

%X Background & Aims: Children with inflammatory bowel disease (IBD) avoid dairy products more than they would need to based on the prevalence of lactose malabsorption and/or allergy to milk proteins because of arbitrary advice from physicians. Aims of the present study were to evaluate the efficacy of a cow's milk protein elimination diet on induction and maintenance of remission and to define association with atopy in children with ulcerative colitis (UC) Methods: 20 consecutive patients (mean age: 10 yrs and 5 months; range: 4 yrs and 7 months to 17 yrs; F/M: 11/9) with newly diagnosed UC were randomised either to receive a cow's milk protein elimination diet (n= 10) or to continue a free diet (n= 10) associated to concomitant steroid induction and mesalamine maintenance treatment. Children were prospectively evaluated at four time points: within 1 month, 2 months, 6 months and 1 year after diagnosis or at the time of relapse. Pediatric Ulcerative Colitis Activity Index (PUCAI) and a physician's global assessment were used to measure disease activity. At baseline and at 12 months or at the time of relapse all patients were assessed endoscopically and histologically. A questionnaire was completed including personal and family history of atopy. At diagnosis, patients were tested for atopy by specific serum IgE and/or skin‐prick tests. Results: All 20 patients responded to the IBD induction therapy. At six months remission was achieved in 5 patients (50%) treated with elimination diet and IBD therapy and in 4 patients (40%) treated with free diet and IBD therapy (p=0.31). Preliminary data showed that 5 of 10 (50%) patients treated with elimination diet and IBD therapy and 4 of 10 (40%) patients treated with free diet and IBD therapy relapsed within 6 months of follow‐up (p=0.5; OR=1.5; CI=0.25‐8.81). At time of enrollment and at time of relapse, endoscopic and histological scores were not statistically different comparing the exclusion diet and free diet groups (p=0.45; p=0.68). All 20 patients completed the questionnaire. The prevalence of atopy in our study population was 32%. Four children in the elimination diet group had a diagnosis of atopy compared with 3 patients in the free diet group (p=0.32). Conclusions: Even though allergy to milk protein has been reported to play a possible role in the etiopathogenesis of UC, preliminary data of this first pediatric, randomised, controlled trial suggest that cow's milk protein elimination diet does not modify the time of induction and the risk of relapse.

%Z Digestive and liver disease

Journal article; Conference proceeding

%U https://cochrane.66557.net/central/doi/10.1002/central/CN-01005808/full

%0 Journal Article

%A Miele, E.

%A Giannetti, E.

%A Martinelli, M.

%A Buonavolontà, R.

%A Strisciuglio, C.

%A Sciorio, E.

%A Staiano, A.

%D 2011

%T Does cow's milk protein elimination diet have a role on induction and maintenance of remission in children with ulcerative colitis?

%V 140

%N 5

%P S513

%8 2011-01-01

%R 10.1016/S0016-5085(11)62127-6

%K *child; *cow; *diet; *gastrointestinal disease; *remission; *ulcerative colitis; Allergy; Atopy; Dairy product; Diagnosis; Disease activity; Enteritis; Etiology; Family history; Follow up; Lactose intolerance; Maintenance therapy; Patient; Physician; Population; Prevalence; Prick test; Questionnaire; Randomized controlled trial; Relapse; Risk; Serum; Skin; Therapy

%X Background & Aims: Children with inflammatory bowel disease (IBD) avoid dairy products more than they would need to based on the prevalence of lactose malabsorption and/or allergy to milk proteins because of arbitrary advice from physicians. Aims of the present study were to evaluate the efficacy of a cow's milk protein elimination diet on induction and maintenance of remission and to define association with atopy in children with ulcerative colitis (UC) Methods: 20 consecutive patients (mean age: 10 yrs and 5 months; range: 4 yrs and 7 months to 17 yrs; F/M: 11/9) with newly diagnosed UC were randomised either to receive a cow's milk protein elimination diet (n=10) or to continue a free diet (n=10) associated to concomitant steroid induction and mesalamine maintenance treatment. Children were prospectively evaluated at four time points: within 1 month , 2 months , 6 months and 1 year after diagnosis or at the time of relapse. Pediatric Ulcerative Colitis Activity Index (PUCAI) and a physician's global assessment were used to measure disease activity. At baseline and at 12 months or at the time of relapse all patients were assessed endoscopically and histologically. A questionnaire was completed including personal and family history of atopy. At diagnosis, patients were tested for atopy by specific serum IgE and/or skin‐prick tests. Results: All 20 patients responded to the IBD induction therapy. At six months remission was achieved in 5 patients (50%) treated with elimination diet and IBD therapy and in 4 patients (40%) treated with free diet and IBD therapy (p=0.31). Preliminary data showed that 5 of 10 (50%) patients treated with elimination diet and IBD therapy and 4 of 10 (40%) patients treated with free diet and IBD therapy relapsed within 6 months of follow‐up (p=0.5; OR=1.5; CI=0.25‐8.81). At time of enrollment and at time of relapse, endoscopic and histological scores were not statistically different comparing the exclusion diet and free diet groups (p=0.45; p=0.68). All 20 patients completed the questionnaire. The prevalence of atopy in our study population was 32%. Four children in the elimination diet group had a diagnosis of atopy compared with 3 patients in the free diet group (p=0.32). Conclusions: . Even though allergy to milk protein has been reported to play a possible role in the etiopathogenesis of UC, preliminary data of this first pediatric, randomised, controlled trial suggest that cow's milk protein elimination diet does not modify the time of induction and the risk of relapse.

%Z Gastroenterology

Journal article; Conference proceeding

%U https://cochrane.66557.net/central/doi/10.1002/central/CN-01765898/full

%0 Journal Article

%A Florin, J.

%A Stalder, O.

%A Baumgartner, C.

%A Mean, M.

%A Rodondi, N.

%A Aujesky, D.

%D 2022

%T Do Patients with a Family or Personal History of Venous Thromboembolism have an Increased Risk of Recurrence?

%V 122

%N 6

%P 1017‐1026

%8 2022-01-01

%R 10.1055/s-0041-1740184

%K *family history; *medical history; *recurrence risk; *venous thromboembolism; Aged; Anticoagulants [adverse effects]; Anticoagulation; Antithrombin deficiency; Article; Blood Coagulation; Body mass; Cohort analysis; Compression ultrasonography; Computer assisted tomography; Confounding variable; Deep vein thrombosis; Female; Human; Human experiment; Humans; Inflammatory bowel disease; Interview; Lung embolism; Lung ventilation; Male; Multicenter study; Nuclear magnetic resonance imaging; Outcome assessment; Outpatient; Phlebography; Prospective Studies; Prospective study; Recurrence; Risk Factors; Risk factor; Sensitivity and specificity; Thorax pain; Venous Thromboembolism [chemically induced, diagnosis, epidemiology]

%X Background  A family (FH) and personal history (PH) of venous thromboembolism (VTE) are commonly evaluated risk factors for recurrence. We examined the association between FH/PH of VTE and the risk of recurrence and whether a stronger history status (i.e., both FH/PH vs. no FH/PH) carries an increased recurrence risk. Methods  We prospectively followed 813 patients aged ≥ 65 years with acute VTE from 9 Swiss hospitals. We classified patients into four groups: no FH/PH, FH only, PH only, and both FH/PH. The primary outcome was recurrent VTE during the full observation period. We examined the association between FH/PH status and the time to VTE recurrence using competing risk regression, adjusting for confounders and periods of anticoagulation. Results  Of 813 patients with VTE, 59% had no FH/PH, 11% a FH only, 24% a PH only, and 7% had both a FH and PH of VTE. Overall, 105 patients had recurrent VTE during the full observation period. After adjustment, patients with a FH only (subhazard ratio [SHR] 0.8, 95% confidence interval [CI] 0.4‐1.7), PH only (SHR 1.5, 95% CI 0.9‐2.5), and both FH/PH (SHR 1.4, 95% CI 0.6‐3.1) did not have an increased risk of recurrent VTE compared with those without FH/PH. When we considered the period after the completion of initial anticoagulation only, the results were similar. Conclusion  Our findings indicate that in patients with acute VTE, a FH and/or PH of VTE does not convey an increased risk of recurrent VTE. In particular, we did not find a dose‐effect relationship between FH/PH status and VTE recurrence.

%Z Thrombosis and haemostasis

Journal article

%U https://cochrane.66557.net/central/doi/10.1002/central/CN-02492317/full

%0 Journal Article

%A Pierre, N.

%A Huynh-Thu, V. A.

%A Baiwir, D.

%A Mazzucchelli, G.

%A Fleron, M.

%A Trzpiot, L.

%A Eppe, G.

%A Laharie, D.

%A Satsangi, J.

%A Colombel, J. F.

%A Et, Al.

%D 2023

%T Distinct biological profiles associated with the risk of short-term relapse and mid/long-term relapse in Crohn's disease patients stopping infliximab

%V 17

%P i3‐i6

%8 2023-01-01

%R 10.1093/ecco-jcc/jjac190.0002

%K *Crohn disease; *relapse; Adult; Australia; Blood clotting; Clinical trial; Conference abstract; Controlled study; Dendritic cell; Drug combination; Drug therapy; Europe; External validity; Female; Human; Human cell; Human tissue; Inflammation; Major clinical study; Male; Protein blood level; Randomized controlled trial; Remission; Risk assessment; Volcano

%X Background: In Crohn's disease (CD) patients stopping infliximab (IFX), the risk of short‐term relapse (<6 months) and mid/long‐term (>6 months) relapse were associated with distinct biological profiles (STORI trial). Herein, we aim to test the external validity of this finding Methods: The SPARE trial has included 211 CD patients (from 64 sites in Europe and Australia) in steroid‐free remission >6 months, receiving a combined therapy (IFX and immunosuppressant (IS)) >8 months and who were then randomised in three arms: Continuing combo, stopping IFX or stopping IS. The arm stopping IFX was used to externally validate our findings generated in the STORI trial. To this end, the measurement of 161 proteins obtained in the baseline serum of STORI was repeated in SPARE (arm stopping IFX) with the same technologies: Selected reaction ‐between serum protein levels and time to relapse (HR: Hazard ratio and its associated statistics) were determined by using univariable Cox model in the stratified (relapse <6 or >6 months) and non‐stratified cohort.Results: The blood proteins depicting the risk of short‐term or mid/long‐term relapse in STORI and SPARE were selected as shown Fig. 1 (SRM) and Fig. 2 (PEA). The representation of those markers in volcano ‐sent distinct biological profiles (Fig. 3‐4). This is also corroborated by mid/long‐term relapse (Fig. 1‐2). In STORI and SPARE, the risk of short‐term relapse was associated (p‐value<0.05) with a high serum level of inflammatory markers (IL6, CRP, HPR, ORM1, LRG1, HP, CP, APCS, ITIH3), complement components (C8B, C4B), blood coagulation pro‐teins (F9, SERPIND1), markers of dendritic cells (LAMP3, CLEC4C) and a low serum level of a complement component (MASP1) (Fig. 1‐2‐3). In STORI and SPARE, the risk of mid/long‐term relapse was associ‐ated (p‐value<0.05) with a high serum level of a complement component (Figure Presented) (CFB) and a low serum level of a protease inhibitor (SERPINA4), a growth factor (FGF2), an anti‐inflammatory cytokine (IL10) (Fig. 1‐2‐ 4). For other proteins, STORI and SPARE showed convergent tendencies (p‐value<0.01; Fig. 1‐2‐3‐4) or differences (Fig. 1‐2) Conclusion: We confirm that, after stopping IFX in CD patients, the risk of short‐term and mid/long‐term relapse are associated with distinct biological profiles. The risk of short‐term relapse is deeply associated with residual inflammation while the biological picture depicting the risk of mid/long‐term relapse seems less clear .

%Z Journal of Crohn's & colitis

Journal article; Conference proceeding

%U https://cochrane.66557.net/central/doi/10.1002/central/CN-02536204/full

%0 Journal Article

%A Buhl, S. S.

%A Steenholdt, C.

%A Brynskov, J.

%A Thomsen, O. Ø.

%A Bendtzen, K.

%A Ainsworth, M. A.

%D 2014

%T Discontinuation of infliximab therapy in patients with Crohn's disease in sustained complete remission (the STOP IT study): protocol for a double-blind, randomised, placebo-controlled, multicentre trial

%V 4

%N 12

%P e005887

%8 2014-01-01

%R 10.1136/bmjopen-2014-005887

%K Adolescent; Adult; Antibodies, Monoclonal [adverse effects, *therapeutic use]; Anti‐Inflammatory Agents, Non‐Steroidal [adverse effects, *therapeutic use]; Crohn Disease [*drug therapy, metabolism]; Double‐Blind Method; Female; Humans; Infliximab; Maintenance Chemotherapy [adverse effects, statistics & numerical data]; Male; Prospective Studies; Recurrence; Remission Induction; Research Design; Tumor Necrosis Factor‐alpha [metabolism]

%X INTRODUCTION: Infliximab (IFX), a monoclonal chimeric antibody against tumour necrosis factor (TNF) α, is effective for induction and maintenance of remission in moderate to severe Crohn's disease. Discontinuation of IFX maintenance therapy in patients in remission should be considered in order to reduce the potential long‐term side effects and lower costs. METHODS AND ANALYSIS: This is a prospective, double‐blind, randomised, placebo‐controlled, multicentre study of patients with luminal Crohn's disease who have been treated with IFX for at least 1 year and are in sustained complete clinical, biochemical and endoscopic remission (ie, Crohn's Disease Activity Index (CDAI) score <150, complete mucosal healing and biochemical markers of inflammation within the normal range). These patients are randomised to receive placebo infusions or continue IFX maintenance therapy. The primary end point is the proportion of patients in maintained remission after 48 weeks (def. CDAI <150). ETHICS AND DISSEMINATION: It is estimated that the knowledge gained about how to optimally handle patients with Crohn's disease in complete long‐term sustained remission on IFX is proportionate to the risks and inconveniences related to participation in this study. Prolonged exposure to IFX may cause severe side effects and increased risk of malignancies. Conversely, IFX discontinuation should not unnecessarily create a high risk of relapse. Thus, empirical evidence is needed concerning the safety of discontinuing IFX once a patient exhibits sustained remission. Study results will be published in an English language scientific medical journal. The study is approved by the Danish Medicines Agency (EudraCT‐number: 2012‐002702‐51) and the Regional Ethics Committee of Region Hovedstaden Denmark (Approval‐number: H‐4‐2012‐099). The project is reported to the Danish Data Protection Agency (ID‐number: 2007‐58‐0015/HEH.750.89‐27), registered at Clinicaltrials.gov, and monitored by independent GCP units for the University of Copenhagen, Odense and Aarhus. The current approved protocol is V.3.2, dated 1 June 2014. TRIAL REGISTRATION NUMBER: http://clinicaltrials.gov/show/NCT01817426.

%Z BMJ open

Journal article

%U https://cochrane.66557.net/central/doi/10.1002/central/CN-01111527/full

%0 Journal Article

%A Buhl, S.

%A Steenholdt, C.

%A Brynskov, J.

%A Christensen, K. R.

%A Dorn-Rasmussen, M.

%A Thomsen, O. O.

%A Bendtzen, K.

%A Klausen, T. W.

%A Dahlerup, J. F.

%A Thorsgaard, N.

%A Et, Al.

%D 2021

%T Discontinuation Of Infliximab Therapy In Patients With Crohn&Apos;S Disease In Sustained, Complete Clinical-Biochemical-Endoscopic Remission: a Double-Blinded, Placebo-Controlled, Randomized Clinical Trial

%V 160

%N 6

%P S‐151‐S‐152

%8 2021-01-01

%R 10.1016/S0016-5085(21)01115-X

%K *relapse; *remission; Adult; Capsule endoscopy; Clinical trial; Conference abstract; Controlled study; Crohn Disease Activity Index; Crohn disease; Diagnostic test accuracy study; Double blind procedure; Drug therapy; Drug withdrawal; Female; Human; Ileocolonoscopy; Maintenance therapy; Major clinical study; Multicenter study; Physician; Randomized controlled trial; Recurrence free survival; Sensitivity analysis; Survival analysis; Treatment duration

%X Background It Remains Unknown Whether Infliximab (Ifx) Successfully Can Be Discontinued Once Crohn’S Disease (Cd) Patients Have Attained Sustained, Complete Clinical, Biochemical, And Endoscopic Remission. No Randomized, Placebo (Pbo) Controlled Trial Has Previously Assessed This. Methods This Double‐Blind, Randomized, Pbo‐Controlled Multicenter Trial Enrolled Patients With Luminal Cd Who Had Been Treated With Standard Ifx Maintenance Therapy For At Least 1 Year, In Complete Remission At The Time Of Inclusion Defined As Cd Activity Index (Cdai) <150, Normal Biochemical Parameters (Crp, Wbc, Hgb, Albumin), And No Signs Of Inflammatory Activity As Assessed By Ileo‐Colonoscopy, Mri, And/Or Capsule Endoscopy. Patients Were Randomized 1:1 To Continue Ifx Therapy Or Matching Pbo For A Duration Of 48 Weeks. Continued Concomitant Therapy With Stable Doses Of Immunomodulators (Thiopurines, Methotrexate) Was Allowed (55% Of Patients). The Primary Endpoint Was Time To Relapse (Defined As Cdai >150 With An Increase In Cdai >70‐Point From Baseline Over Two Consecutive Weeks; Or Definitive Clinical Relapse Requiring Immediate Intervention As Judged By Treating Physician) In The Intention‐To‐Treat Population. Results The Study Population Comprised 115 Patients (N=54 Female; Age Median 34 Years [Iqr 26‐50]; Disease Duration Median 6 Years [3‐12]; Ifx Treatment Duration Median 23 Months [16‐39]). All Patients Were In Combined Clinical‐ (Cdai Median 41 [Iqr 15‐66]), Biochemical‐ (Crp Median 3mg/L [Iqr 2‐4]), And Endoscopic‐ (Simple Endoscopic Score For Cd Median 0, [Iqr 0‐0] (N=99)) Remission. Patients Were Randomized To Continued Ifx Therapy (N=59) Or To Start Pbo Infusions (N=56) (Figure 1). Time To Relapse Was Significantly Shorter Among Patients Who Discontinued Ifx As Compared To Those Continuing Ifx (P<0.001) (Figure 2). After One Year, Relapse‐Free Survival Was 51% In The Pbo Group And 100% In The Ifx Group (Kaplan Meier Survival Analysis). Concomitant Immunosuppressants Numerically Increased Time To Relapse For Placebo Treated Patients. However, Also For This Subgroup, Time To Relapse Remained Significantly Shorter Than In The Ifx Group. In Sensitivity Analysis Based On A Less Stringent Definition Of Relapse (One Cdai Assessment >150) The Figures Were 47% In The Pbo Group Vs. 98% In Ifx Group (P<0.001). Proportion Of Per‐Protocol Patients (I.E. Withdrawals And Dropouts Excluded) In Clinical Remission (Cdai <150) By The End Of Trial Was Significantly Higher In The Ifx Group (Remission In N=52 Of 54 (96%) Vs. N=23 Of 47 (49%); P<0.001). Conclusion This First Double‐Blinded Placebo‐Controlled Rct Of Ifx Withdrawal In Crohn’S Disease Patients Strongly Suggests That Discontinuation Of Ifx Leads To A Considerable Risk Of Relapse Despite Combined Clinical, Biochemical, And Endoscopic Remission (Figure Presented) (Figure Presented)

%Z Gastroenterology

Journal article; Conference proceeding

%U https://cochrane.66557.net/central/doi/10.1002/central/CN-02356145/full

%0 Journal Article

%A NCT

%D 2020

%T De-escalation of Anti-TNF Therapy in Inflammatory Bowel Disease

%8 2020-01-01

%K Adalimumab; Colitis; Colitis, Ulcerative; Crohn Disease; Inflammatory Bowel Diseases; Infliximab; Intestinal Diseases; Ulcer

%X STUDY DESIGN: International, multi‐centre, prospective, partially randomised patient‐preference trial. STUDY POPULATION: Study population: Eligible patients are aged 12‐25 years with luminal Crohn's disease (CD) or ulcerative colitis (UC), who have three consecutive faecal calprotectin (FC) results in the target range (i.e. <250 µg/g for CD patients; <150 µg/g for UC patients) over a period of 6 months at study entry or recently confirmed endoscopic remission. DE‐ESCALATION STRATEGY: In patients treated with adalimumab, the dosing interval will be lengthened from 2 to 3 weeks. In patients treated with infliximab, the dosing interval will be lengthened from 8 to 12 weeks. FC rapid tests will be performed every 4 weeks and rapid tests for anti‐TNF trough levels will be performed every 12 weeks. MAIN STUDY ENDPOINTS: The primary outcome is the cumulative incidence of out‐of‐range FC results at 48 weeks follow‐up. Secondary endpoints include time to get out‐of‐range FC results, cumulative incidence of anti‐TNF associated adverse effects, proportion of patients progressing from out‐of‐range FC to loss‐of‐response and identification of predictors of successful de‐escalation. ETHICAL CONSIDERATIONS: Patients with reduced anti‐TNF exposure may have a higher risk of out‐of‐range FC results and, on the other hand, may benefit from fewer hospital visits or injections and possibly a decrease in adverse effects of anti‐TNF therapy. Tight monitoring of FC levels (i.e. 4‐weekly) will allow institution of re‐escalation before the patient manifests clinical signs of relapse. This study cannot be conducted without the participation of minors and young adults, who typically have a short disease duration. Early treatment with anti‐TNF agents possibly modifies the course of their disease, which makes provision for safe deescalation.

%Z https://clinicaltrials.gov/show/NCT04646187

Trial registry record

%U https://cochrane.66557.net/central/doi/10.1002/central/CN-02205953/full

%0 Journal Article

%A Garcia-Olmo, D.

%A Panes, J.

%A Brayshaw, N.

%A Richard, M. P.

%A Agboton, C.

%D 2018

%T Cx601, a novel stem cell therapy for complex perianal fistulas in Crohn's disease has rapid onset of clinical remission and low relapse rate by week 52

%V 12

%P S460‐S461

%8 2018-01-01

%K *Crohn disease; *anus fistula; *cell therapy; *recurrence risk; *remission; *stem cell; Adult; Clinical assessment; Conference abstract; Controlled study; Female; Finger; Hazard ratio; Human; Human cell; Kaplan Meier method; Major clinical study; Male; Nuclear magnetic resonance imaging; Phase 3 clinical trial; Post hoc analysis; Proportional hazards model; Randomized controlled trial

%X Background: Cx601 is an expanded allogeneic adipose‐derived mesenchymal stem cell local therapy for complex perianal fistulas in patients with Crohn's disease (CD). In the Phase III, pivotal, ADMIRE‐CD study of patients with CD and treatment‐refractory, draining complex perianal fistulas, more patients receiving Cx601 achieved clinical remission at week 24 compared with standard of care (SoC). Here we describe post hoc analyses of time to clinical remission and relapse by week 52 in ADMIRE‐CD. Methods: Clinical remission was defined as closure of all treated external openings that were draining at baseline despite gentle finger pressure. Relapse by week 52 was defined as reopening of any treated external openings with active drainage as clinically assessed, and/or development of a perianal collection >2 cm of the treated perianal fistulas confirmed by centrally blinded MRI assessment in patients with combined remission at week 24 (i.e. clinical remission and no collections >2 cm). Confidence intervals for difference in rates were calculated by the asymptotic Wald method. Times to clinical remission and relapse were analysed by Cox regression with accompanying Kaplan‐Meier estimates. Analyses of subgroups with and without concomitant medication were also performed. Results: The intent‐to‐treat population comprised 212 randomised patients. At week 6, 51.4% of patients receiving Cx601 were in clinical remission vs. 34.3% in the SoC group (absolute difference 17.1 percentage points; 95% CI: 4.0‐30.2; relative difference 49.9%). The difference persisted across visits and at week 52 was 17.0 percentage points (57.0% vs. 40.0% for Cx601 and SoC, respectively; 95% CI: 3.8‐30.3; relative difference 42.5%). The median time to clinical remission at week 52 was 6.7 weeks (95% CI: 6.4‐11.9) in the Cx601 group vs. 14.6 weeks (95% CI: 11.9‐22.9) in the SoC group (hazard ratio [HR]=1.72 [95% CI: 1.25‐2.38]). By week 52, 25.0% (13/52) of patients receiving Cx601 relapsed vs. 44.1% (15/34) in the SoC group (difference of minus 19.1 percentage points; 95% CI:‐39.5 to 1.3). A longer time to relapse by week 52 was observed for patients receiving Cx601 compared with the SoC group (HR=0.47 [95% CI: 0.22‐1.00]). A similar trend in time to clinical remission was also observed in subgroups irrespective of concomitant therapy. Conclusions: Patients receiving Cx601 experienced a shorter time to clinical remission and a longer duration of remission compared with SoC. This was observed in subgroups of patients with/without concomitant medication. These results support the efficacy of Cx601 in patients with CD and treatment‐refractory, draining complex perianal fistulas.

%Z Journal of Crohn's & colitis

Journal article; Conference proceeding

%U https://cochrane.66557.net/central/doi/10.1002/central/CN-01466830/full

%0 Journal Article

%A Fornaro, R.

%A Frascio, M.

%A Stabilini, C.

%A Sticchi, C.

%A Barberis, A.

%A Denegri, A.

%A Ricci, B.

%A Azzinnaro, A.

%A Lazzara, F.

%A Gianetta, E.

%D 2008

%T Crohn's disease surgery: problems of postoperative recurrence

%V 60

%N 6

%P 761‐781

%8 2008-01-01

%K *Crohn disease /drug therapy /surgery; Adolescent; Adult; Aged; Antibodies, Monoclonal [administration & dosage, therapeutic use]; Anti‐Infective Agents [administration & dosage, therapeutic use]; Anti‐Inflammatory Agents [administration & dosage, therapeutic use]; Anti‐Inflammatory Agents, Non‐Steroidal [administration & dosage, therapeutic use]; Comparative study; Crohn Disease [*drug therapy, *surgery]; Evaluation study; Female; Follow up; Follow‐Up Studies; Forecasting; Gastrointestinal Agents [administration & dosage, therapeutic use]; Human; Humans; Immunosuppressive Agents [administration & dosage, therapeutic use]; Infliximab; Laparoscopy; Male; Mesalamine [administration & dosage, therapeutic use]; Metronidazole [administration & dosage, therapeutic use]; Middle Aged; Middle aged; Postoperative Care; Postoperative care; Randomized Controlled Trials as Topic; Randomized controlled trial; Recurrence; Recurrent disease; Reoperation; Retrospective Studies; Retrospective study; Review; Risk Factors; Risk factor; Time; Time Factors

%X Crohn's disease is associated with high rates of postoperative recurrence. By 10 years postoperatively a high percentage of patients suffer clinical relapses (as many as 75% and above) and many of these (up to 45%) require re‐intervention. The aim of the study was to identify, among the various potentially predictive factors, those which today can be considered real risk factors for postoperative recurrence. A review of the literature of the last two decades was carried out. Factors related to the patient, disease, type of surgery and pharmacological treatment were analysed. The relapse rate we recorded was 41.17% (28 of 62 patients operated on in the last 20 years and included in an average 6‐year follow‐up (range: 1‐19 years). Significant predictive factors, in adition to duration of the follow‐up and smoking, are also the location of the disease in the colon, extent more than 100 cm and the absence of postoperative pharmacological prophylaxis. The high incidence of postoperative recurrence in Crohn's disease mandates a strict follow‐up (clinical, laboratory and instrumental monitoring). Identifying patients at increased risk would enable physicians to implement a rational pharmacological prophylaxis.

%Z Chirurgia italiana

Journal article

%U https://cochrane.66557.net/central/doi/10.1002/central/CN-01743464/full

%0 Journal Article

%A De Cruz, P.

%A Kamm, M. A.

%A Hamilton, A. L.

%A Ritchie, K. J.

%A Krejany, E. O.

%A Gorelik, A.

%A Liew, D.

%A Prideaux, L.

%A Lawrance, I. C.

%A Andrews, J. M.

%A Et, Al.

%D 2015

%T Crohn's disease management after intestinal resection: a randomised trial

%V 385

%N 9976

%P 1406‐1417

%8 2015-01-01

%R 10.1016/S0140-6736(14)61908-5

%K Adalimumab; Adult; Antibodies, Monoclonal, Humanized [administration & dosage]; Azathioprine [therapeutic use]; Colonoscopy; Crohn Disease [pathology, surgery, *therapy]; Female; Humans; Male; Mercaptopurine [therapeutic use]; Metronidazole [therapeutic use]; Middle Aged; Recurrence; Treatment Outcome

%X BACKGROUND: Most patients with Crohn's disease need an intestinal resection, but a majority will subsequently experience disease recurrence and require further surgery. This study aimed to identify the optimal strategy to prevent postoperative disease recurrence. METHODS: In this randomised trial, consecutive patients from 17 centres in Australia and New Zealand undergoing intestinal resection of all macroscopic Crohn's disease, with an endoscopically accessible anastomosis, received 3 months of metronidazole therapy. Patients at high risk of recurrence also received a thiopurine, or adalimumab if they were intolerant to thiopurines. Patients were randomly assigned to parallel groups: colonoscopy at 6 months (active care) or no colonoscopy (standard care). We used computer‐generated block randomisation to allocate patients in each centre to active or standard care in a 2:1 ratio. For endoscopic recurrence (Rutgeerts score ≥i2) at 6 months, patients stepped‐up to thiopurine, fortnightly adalimumab with thiopurine, or weekly adalimumab. The primary endpoint was endoscopic recurrence at 18 months. Patients and treating physicians were aware of the patient's study group and treatment, but central reading of the endoscopic findings was undertaken blind to the study group and treatment. Analysis included all patients who received at least one dose of study drug. This trial is registered with ClinicalTrials.gov, number NCT00989560. FINDINGS: Between Oct 13, 2009, and Sept 28, 2011, 174 (83% high risk across both active and standard care groups) patients were enrolled and received at least one dose of study drug. Of 122 patients in the active care group, 47 (39%) stepped‐up treatment. At 18 months, endoscopic recurrence occurred in 60 (49%) patients in the active care group and 35 (67%) patients in the standard care group (p=0.03). Complete mucosal normality was maintained in 27 (22%) of 122 patients in the active care group versus four (8%) in the standard care group (p=0.03). In the active care arm, of those with 6 months recurrence who stepped up treatment, 18 (38%) of 47 patients were in remission 12 months later; conversely, of those in remission at 6 months who did not change therapy recurrence occurred in 31 (41%) of 75 patients 12 months later. Smoking (odds ratio [OR] 2.4, 95% CI 1.2‐4.8, p=0.02) and the presence of two or more clinical risk factors including smoking (OR 2.8, 95% CI 1.01‐7.7, p=0.05) increased the risk of endoscopic recurrence. The incidence and type of adverse and severe adverse events did not differ significantly between patients in the active care and standard care groups (100 [82%] of 122 vs 45 [87%] of 52; p=0.51) and (33 [27%] of 122 vs 18 [35%] of 52; p=0.36), respectively. INTERPRETATION: Treatment according to clinical risk of recurrence, with early colonoscopy and treatment step‐up for recurrence, is better than conventional drug therapy alone for prevention of postoperative Crohn's disease recurrence. Selective immune suppression, adjusted for early recurrence, rather than routine use, leads to disease control in most patients. Clinical risk factors predict recurrence, but patients at low risk also need monitoring. Early remission does not preclude the need for ongoing monitoring. FUNDING: AbbVie, Gutsy Group, Gandel Philanthropy, Angior Foundation, Crohn's Colitis Australia, and the National Health and Medical Research Council.

%Z Lancet (london, england)

Journal article

%U https://cochrane.66557.net/central/doi/10.1002/central/CN-01071267/full

%0 Journal Article

%A Cohen, Z.

%D 2000

%T Crohn's disease - Outcome of surgery

%V 36

%N SUPPL. G

%P 51‐57

%8 2000-01-01

%K *Crohn disease /diagnosis /drug therapy /surgery; Adult; Clinical trial; Conference paper; Controlled study; Disease activity; Endoscopy; Female; Human; Intestine anastomosis; Intestine resection; Large intestine; Major clinical study; Male; Onset age; Quality of life; Randomized controlled trial; Recurrent disease; Remission; Risk factor; Small intestine; Smoking; Stapler; Treatment outcome

%X Although medical therapy is the primary method of treatment for patients with Crohn's disease, approximately 80% of affected patients will require surgery at some time. Surgery is usually effective in eliminating the complications of the disease and improving quality of life. However, the disease often recurs in a large proportion of patients and requires further medical and even surgical therapy. In the literature, recurrence rates of 3.4‐16% per year have been reported depending on the criteria used to define recurrence. Various surgical maneuvers do not seem to decrease this recurrence rate and will be addressed below. Certain drugs may be effective as maintenance therapy and now such drugs as the 5‐aminosalicylic acid (5‐ASA) compounds and azathioprine have been shown to have some effect in maintaining remission following surgery. We undertook a randomized, multicenter, controlled trial to determine whether 5‐ASA was effective in reducing the risk of recurrent Crohn's disease in patients who had had a surgical resection. Within 8 weeks of surgery, 163 patients who had no evidence of gross residual disease were randomized to a group administered 1.5 g 5‐ASA twice daily, or to a placebo control group. All patients underwent follow‐up endoscopic or the results. The factors mentioned above, such as the use of prophylactic medication to delay the onset of recurrence, were not able to be studied in this analysis due to the lack of information available regarding use of medication in the population studied.

%Z Drugs of today (Barcelona, Spain : 1998)

Journal article; Conference proceeding

%U https://cochrane.66557.net/central/doi/10.1002/central/CN-01710584/full

%0 Journal Article

%A Karstensen, J. G.

%A Săftoiu, A.

%A Brynskov, J.

%A Hendel, J.

%A Klausen, P.

%A Cârtână, T.

%A Klausen, T. W.

%A Riis, L. B.

%A Vilmann, P.

%D 2016

%T Confocal laser endomicroscopy: a novel method for prediction of relapse in Crohn's disease

%V 48

%N 4

%P 364‐372

%8 2016-01-01

%R 10.1055/s-0034-1393314

%K Adult; Aged; Colon [pathology]; Colonoscopy [*methods]; Crohn Disease [*diagnosis]; Feasibility Studies; Female; Fluorescein [*pharmacology]; Fluorescent Dyes [pharmacology]; Humans; Ileum [*pathology]; Intestinal Mucosa [*pathology]; Male; Microscopy, Confocal [*methods]; Middle Aged; Predictive Value of Tests; Prospective Studies; Rectum [pathology]; Recurrence; Reproducibility of Results; Risk Factors

%X BACKGROUND AND STUDY AIMS: Confocal laser endomicroscopy (CLE) has been shown to predict relapse in ulcerative colitis in remission, but little is currently known about its role in Crohn's disease. The aim of this study was to identify reproducible CLE features in patients with Crohn's disease and to examine whether these are risk factors for relapse. PATIENTS AND METHODS: This was a single‐center prospective feasibility study of CLE imaging in patients with Crohn's disease. CLE imaging was performed in the terminal ileum and four colorectal sites, and was correlated with histopathology and macroscopic appearance. Clinical relapse, defined as the need for treatment escalation or surgical intervention, was recorded during follow‐up. RESULTS: The study included 50 patients: 39 with Crohn's disease (20 in remission), and 11 controls. Ileal fluorescein leakage and microerosions were significantly more frequent in patients with endoscopically active Crohn's disease compared with patients with inactive Crohn's disease and controls (P = 0.005 and (P = 0.006, respectively). The same applied to colorectal fluorescein leakage and vascular alterations ((P = 0.043 and (P = 0.034, respectively). During a 12‐month follow‐up period, ileal fluorescein leakage and microerosions were significant risk factors for relapse in the subgroup of patients in remission (log rank (P = 0.009 and (P = 0.007, respectively) as well as in the entire group of patients with Crohn's disease (log rank (P = 0.006 and (P = 0.01, respectively). Inter‐ and intraobserver reproducibility was almost perfect (κ > 0.80) or substantial (κ > 0.60) for the majority of CLE parameters. CONCLUSIONS: CLE can identify reproducible microscopic changes in the terminal ileum that are risk factors for relapse in patients with otherwise inactive Crohn's disease. TRIAL REGISTRATION: ClinicalTrials.gov (NCT01738529).

%Z Endoscopy

Journal article

%U https://cochrane.66557.net/central/doi/10.1002/central/CN-01264594/full

%0 Journal Article

%A Demetrashvili, Z.

%A Kenchadze, G.

%A Pipia, I.

%A Khutsishvili, K.

%A Loladze, D.

%A Ekaladze, E.

%A Merabishvili, G.

%A Kamkamidze, G.

%D 2019

%T Comparison of treatment methods of appendiceal mass and abscess: a prospective Cohort Study

%V 48

%P 48‐52

%8 2019-01-01

%R 10.1016/j.amsu.2019.10.016

%K *abscess /drug therapy /surgery; *acute appendicitis /drug therapy /surgery; *appendix disease /drug therapy /surgery; *conservative treatment; *emergency surgery; Abdominal abscess; Adult; Anastomosis leakage; Antibiotic therapy; Appendectomy; Appendix perforation /surgery; Article; Cecum cancer /surgery; Controlled study; Crohn disease; Female; Follow up; Hemicolectomy; Hospitalization; Human; Intermethod comparison; Length of stay; Major clinical study; Male; Operation duration; Percutaneous drainage; Postoperative complication; Priority journal; Prospective study; Recurrent disease; Surgical infection

%X Background: The aim of our prospective study is to compare and analyze the results of two treatment methods of appendiceal mass and abscess: emergency surgery and conservative treatment with and without interval surgery. Materials and methods: 74 Patients with the diagnosis of appendiceal mass or abscess were enrolled in this study. The patients were assigned into two groups: the emergency surgery group and the conservative management group. The conservative management group was subdivided into two groups: interval surgery group and the ambulatory follow‐up observation group without interval surgery. Several clinical characteristics were determined and compared between the groups. Among patients who underwent surgery, the surgical methods, operation time, postoperative hospitalization period, and post‐surgical complications were analyzed. In the ambulatory follow‐up observation group, recurrence of appendicitis was assessed. Results: Comparison of the emergency surgery group and interval surgery group revealed that the interval surgery group was characterized by shorter operation time (P = 0.008), a smallernumber of postoperative complications (P = 0.02) and also shorter postoperative hospital stay (P = 0.009). In the ambulatory follow‐up observation group, recurrence of appendicitis developed in 3 (13%) patients. US or CT‐guided PCD was performed in all 3 patients on the conservative treatment stage. Comparing the interval surgery and recurrent appendicitis groups revealed statistically significant difference: operation time (P = 0.04) as well as postoperative hospital stay (P = 0.04) were shorter in recurrent appendicitis group. In 3 (4.1%) patients, the cause of the appendiceal mass was caecal cancer (2 cases) and Crohn's disease. Conclusion: Conservative treatment without interval surgery seems to be the preferred method for treatment of appendiceal mass and abscess. Patients can be operated on only in case of recurrence of appendicitis. US or CT PCD of appendiceal abscess presents the risk‐factor for the development of recurrence of appendicitis. CT and colonoscopy within 4–6 weeks after completing the conservative treatment is recommended to be performed in all patients.

%Z Annals of medicine and surgery

Journal article

%U https://cochrane.66557.net/central/doi/10.1002/central/CN-02093086/full

%0 Journal Article

%A Tursi, A.

%A Elisei, W.

%A Picchio, M.

%A Zampaletta, C.

%A Pelecca, G.

%A Faggiani, R.

%A Brandimarte, G.

%D 2014

%T Comparison of the effectiveness of infliximab and adalimumab in preventing postoperative recurrence in patients with Crohn's disease: an open-label, pilot study

%V 18

%N 11

%P 1041‐1046

%8 2014-01-01

%R 10.1007/s10151-014-1177-1

%K *Crohn disease/co [Complication]; *Crohn disease/dt [Drug Therapy]; *Crohn disease/pc [Prevention]; *Crohn disease/su [Surgery]; *adalimumab/cm [Drug Comparison]; *adalimumab/dt [Drug Therapy]; *postoperative complication/co [Complication]; *postoperative complication/dt [Drug Therapy]; *postoperative complication/pc [Prevention]; *recurrent disease/co [Complication]; *recurrent disease/dt [Drug Therapy]; *recurrent disease/pc [Prevention]; *recurrent disease/su [Surgery]; Adalimumab; Adult; Antibodies, Monoclonal [*administration & dosage]; Antibodies, Monoclonal, Humanized [*administration & dosage]; Anti‐Inflammatory Agents, Non‐Steroidal [administration & dosage]; Article; Biopsy; Clinical article; Clinical effectiveness; Colon biopsy; Colon resection; Controlled study; Crohn Disease [diagnosis, *drug therapy, surgery]; Digestive System Surgical Procedures; Disease control; Dose‐Response Relationship, Drug; Drug efficacy; Drug response; Endoscopy, Digestive System; Female; Follow‐Up Studies; High risk population; Histopathology; Human; Human tissue; Humans; Infliximab; Infliximab/cm [Drug Comparison]; Injections, Subcutaneous; Male; Outcome assessment; Pilot Projects; Pilot study; Postoperative Period; Postoperative period; Prospective Studies; Prospective study; Recurrence; Risk assessment; Risk factor; Scoring system; Treatment Outcome; Treatment indication; Tumor Necrosis Factor‐alpha [antagonists & inhibitors]; Young Adult; Young adult

%X Background: Endoscopic and clinical recurrence of Crohn's disease (CD) appears in up to 80 and 30 % of patients, respectively, 1 year after surgery. Both infliximab (IFX) and adalimumab (ADA) have been demonstrated to be effective in reducing the possibility of recurrence after surgery, but head‐to‐head studies have not been performed so far. The aim of this open‐label prospective study was to compare endoscopic, histological and clinical recurrence after 1 year of treatment with IFX or ADA as postoperative prophylaxis in CD patients with a high risk of recurrence. Methods: Consecutive CD patients who underwent curative ileocolonic resection were randomized to receive IFX or ADA for 1 year. Co‐primary endpoints were endoscopic, histological and clinical recurrence after 12 months of therapy. Results: Twenty consecutive CD patients (9 males and 11 females; median age 32.5 years, range 20‐39 years) were enrolled after undergoing curative ileocolonic resection. Among the 10 patients treated with IFX, 2 (20 %) had endoscopic recurrence compared to 1 (10 %) in the group of 10 ADA patients (p = 1.0). Three out of 10 (30 %) IFX patients and 2 out of 10 (20 %) ADA patients had histological recurrence (p = 1.0). No significant clinical differences were found between the two groups. Conclusions: IFX and ADA were similar in preventing histological, endoscopic and clinical recurrence after curative ileocolonic resection in high risk CD patients.

%Z Techniques in coloproctology

Journal article

%U https://cochrane.66557.net/central/doi/10.1002/central/CN-01072416/full

%0 Journal Article

%A NCT

%D 2020

%T Comparative Study of Dietary and Immunological Management of Refractory Epilepsy in Children

%8 2020-01-01

%K Drug Resistant Epilepsy; Epilepsy; Rituximab; Trace Elements

%X 1. Definition of refractory epilepsy A task force of the International League Against Epilepsy proposed that drug‐resistant epilepsy be defined as failure of adequate trials of two tolerated and appropriately chosen and used antiseizure drug schedules (whether as monotherapies or in combination) to achieve sustained seizure freedom (1) . This requires application of the intervention at adequate strength/dosage for a sufficient length of time (2)..Terms refractory" intractable", drug‐resistant" pharmaco‐resistant" have been used interchangeably in several documented literature in different time periods (3). The first antiseizure drug fails in 20 to 40 percent of children with epilepsy; lack of efficacy and side effects contribute to treatment failure (4) Epidemiology About 10‐20 % children with epilepsy develop drug refractory epilepsy (5).In a systematic review of 35 observational studies that included over 13,000 patients with epilepsy and 3900 patients with drug resistant epilepsy( DRE), the pooled prevalence of (DRE) was 30 percent, and the pooled incidence proportion was 15 percent (6). Risk factors ‐ A high number of seizures prior to diagnosis and treatment ‐ Genetic or inherited syndromes, for both generalized and localization‐related epilepsy, have a better prognosis than symptomatic/cryptogenic epilepsy in both pediatric and adult populations (7). ‐ Localization‐related epilepsy underlies more than half of the cases of DRE in children (8,9). ‐ Other findings more variably associated with the risk of DRE include a presentation with status epilepticus , a longer duration of epilepsy , a family history of epilepsy , a history of febrile convulsions , abnormal ( ELECTROENCEPHALOGRAM ) EEG findings . An abnormal neurologic examination and/or developmental delays (10,11). ‐ Some pediatric studies have found that seizure onset in later childhood or adolescence appears to be more likely to be associated with DRE than seizures with onset between the ages of 5 and 10 years (12). Causes (13,14 ) Epilepsy syndromes Infancy • Epilepsy of infancy with migrating focal seizures ‐ West syndrome ‐ Dravet syndrome ‐ Doose syndrome Childhood ‐ Lennox‐Gastaut syndrome ‐ Epileptic encephalopathy with continuous spike and wave during sleep Metabolic • Pyridoxine dependent epilepsy • Biotinidase biosynthesis deficiency • Organic acidemia • Urea cycle disorders • Non‐ketotic hyperglycinemia ‐ Aminoacidopathies ‐ Mitochondrial disorders including Alpers syndrome ‐ Hashimoto encephalopathy Structural Abnormality • Malformations: Neuronal migration defects and neural tube defects • Neurocutaneous syndromes , Infectious/Inflammatory: , • Hypoxic ischemic encephalopathy, • Rasmussen encephalitis ‐ Stroke ‐ Tumors ‐ Mesial temporal sclerosis Autoimmune epilepsies : N‐methyl‐D‐aspartate receptor(NMDAR), Connective tissue disorders • Systemic lupus erythematosus, • Wegener's granulomatosis ‐ , sarcoidsosis, ‐ celiac disease, ‐ Crohn's disease, ‐ Behcet's ‐ Sjorgren's syndrome, EVALUATION A‐ History and presentation : The approach depends upon age of onset, accurate description: pre‐ictal, ictal, postictal events, precipitating events, seizure types, evolution relation to fever, previous history of febrile seizures (simple/complex), associated non‐epileptic events, intake of (ANTI EPILPTIC DRUGS )AEDs with dose, developmental history, sleep history, detailed birth and peri‐/antenatal history etc. B‐Clinical examination depends upon anthropometry, syndromic facial dysmorphism, neurocutaneous features and detailed neurological and systemic assessment C‐ Pseudo‐refractoriness indicates a condition in which seizures persist because the condition has not been adequately treated. The most common causes of pseudo refractory epilepsy include 1. Inappropriate diagnosis ‐Cardiogenic (arrhythmias) and vasovagal events (syncope) ‐ Parasomnias ‐ Movement disorders (paroxysmal dyskinesias, cataplexy) ‐ Psychogenic nonepileptic seizure (PNES) can mimic epileptic seizures. , do not respond to antiseizure drug therapy. While not without limitations, video‐EEG monitoring is the gold standard test for the diagnosis of PNES 2. Bad compliance ; Missed dose 3. Incorrect drug An incorrect diagnosis of seizure classification leading to incorrect drug choice It is not uncommon for diopathic generalized epilepsy syndromes to be unrecognized and inappropriately treated with antiseizure drugs that are more appropriate to localization‐related epilepsy . 4. Inadequate dosage 5. Inadequate frequency 6. Enzyme induction, especially if more than one antiepilptic drug AED or other medication is used 7. Inadequate anticonvulsant therapy D‐Investigation for the patients include; 1‐EEG Routine EEG is a must for the clinical diagnosis of epilepsy and associated syndrome. For the majority of patients with epilepsy, routine EEG is sufficient to classify seizure type and to start treatment But for refractory epilepsy ( RE) and a doubtful seizure, video‐EEG monitoring is the best diagnostic tool available. 2‐Drug level monitor 3‐Serum electrolyte, serum glucose 4‐MRI brain ; High‐resolution MRI helps in isolating the cause of focal epilepsies and to predict long‐term outcome and spontaneous remission in patients. In some cases, follow‐up MRI reveals an etiology for epilepsy (such as cerebral neoplasm, autoimmune encephalitis) that was not seen on the initial study and requires specific therapies in addition to antiseizure drugs . 5‐ Metabolic Work up Arterial blood gas (ABG ) Serum ammonia ,lactate Tandem mass screening 6‐Genetic Testing as karyotyping , gene sequence ,‐Fundus examination The presence of features like dysmorphism, growth retardation, intellectual disability and hypotonia may indicate an underlying genetic syndrome, 7‐CSF culture, analysis • Lumbar puncture of infectious process 8‐vascuilitis panel Complete blood cell count Erythrocyte sedimentation rate CRP ( C REACTIVE PROTEIN ) VWF Ag (VON WILLEBRAND FACTOR) C 3, 4 (COMPLEMENT 3, 4) Antinuclear AB (ANTIBODY) Antiphospholipid AB Antineutrophil cytoplasmic AB Anti‐DNA AB 9‐autoimmune encephalitis (Hashimotos ) antithyroid peroxidase AB, anti‐TSH receptor AB Anti‐NMDA‐R AB Anti‐MOG AB (if CNS demyelination) abdominal ultrasound and ‐MRI abdomen;To detect ovarian teratoma as in autoimmune encephalitis F‐Practice recommendations for AED trial: 1. Optimise the dose of each AED by increasing the dose incrementally. If the maximum dose is ineffective introduce a second AED while continuing on the first. If seizure control is achieved, consider tapering the first AED. The advice to "start low and go slow" is appropriate 2. If one or two AEDs are ineffective, rational polytherapy should be explored. 3. Consider using AEDs with different mechanisms of action 4. Avoid using an AED that may worsen or provoke seizures Carbamazepine (CBZ), Oxcarbazepine (OXC), Phenytoin (PHT), Vigabatrin (VGB) and Tiagabine (TGB) may worsen myoclonus and absence seizures ‐ Gabapentin (GBP) and Lamotrigine (LTG) may worsen myoclonus (Benzodiazepines given intravenously may worsen tonic seizures but may be very useful in treating Lennox‐Gastaut and does not contraindicate their use Background A‐Immunotherapy Evidence that the immune system is involved in the pathogenesis of epilepsy particularly, medically refractory epilepsy, has given rise to the use of adjunctive immunotherapy to slow or change the epileptogenic process. ‐ The presumed immune therapeutic mechanism (ACTH, corticosteroids, plasmapheresis human immunoglobulin G, rituximab, azathioprine, and cyclophosphamide) is the removal of various neuroimmunological mechanisms involved. However, new models suggest suppression of endogenous brain agent's proconvulsant (neuropeptides) There is limited data of these treatments outside of specific epileptic encephalopathies such as West syndrome, Rasmussen encephalitis, Landau Kleffner and specific antibody mediated encephalitis such as anti NMDA encephalitis a) Corticosteroids: Their immunological mechanisms are inhibition diapedesis and therefore infiltration of lymphocytes to the injured areas, attenuating production of inflammatory humoral mediators (IL‐2) and inhibition of leukocyte function (helper T lymphocytes mainly) as well as endothelial cells. Dose: Methylprednisolone 30 mg /kg /day for 5 days up to 1 gm /day ,pulsed monthly for 6 months (15). b) Plasmapheresis: the activity of auto‐Abs can be modulated by treatment with IVIGs and by plasma treatment, which consists of the mechanical removal of Abs (16) c) Human immunoglobulin G: . IgGIV inhibits activation of innate cells, suppression of the production of proinflammatory cytokines, and soluble factors as tumor necrosis factor and interleukins induced by this factor. In endothelial cells and macrophages, suggesting a mechanism antiepileptic Dose:400 mg.kg /day for 5 days D‐‐Rituximab: Rituximab, a chimeric monoclonal antibody that binds to CD20, was the first monoclonal antibody to be approved for clinical use in the therapy of cancer. It is approved for use against indolent B‐cell non‐Hodgkin's lymphoma (NHL), although its use has expanded significantly beyond that indication to virtually any CD20‐positive NHL, and more recently into other areas such as autoimmune disorders ) Rituximab targets CD20, a transmembrane protein present on virtually all B cells from the stage at which they become committed to B‐cell development until it is downregulated when they differentiate into antibody‐secreting plasma cells (17) Numerous review articles whose primary focus is the action of rituximab in the treatment of RA, systemic lupus erythematosus (SLE) and other autoimmune diseases were published in 2006. Although there is a clear consensus that substantial clinical benefits are associated with rituximab therapy, the exact mechanism(s) by which the B‐cell depletion promoted by rituximab ameliorates autoimmune disease activity remains an area of active discussion.(18, 19) The most likely mechanisms have naturally centered on the pathogenic role of B cells in autoimmune disease: these include the potential of B cells to give rise to plasma cells that secrete autoantibodies as well as the ability of autoantigen‐specific B cells to present antigens to autoreactive T cells, which leads to T‐cell activation, cytokine release and inflammation. In certain cases the results of clinical trials have demonstrated moderate correlations between reduction of autoantibody levels and lessening of disease symptoms, which has emphasized the role of autoantibodies in disease pathology. In other studies such correlations have not been observed, which has led investigators to favor B cell‐T cell interactions as the most important in the etiology of tissue injury in autoimmune diseases. (20 ,21) B‐Dietary Therapy 2. trace elements and antioxidants; The equilibrium of trace elements is essential for a healthy nervous system due to their key roles in activation of specific enzyme in many pathways of the central nervous system function metabolism ‐ Essential trace elements that include zinc, copper, magnesium, and selenium might play a role in the pathogenesis of seizures because of their possible influence on synaptogenesis, their actions on ligand‐ and voltage‐gated ion channels, their effects on membrane lipid peroxidation and turnover of some neurotransmitters, and their roles in immunity (26) . Glutathione peroxidase (GPx) and superoxide dismutase (SOD) are two major enzymes that are involved in antioxidative defense mechanisms. 1. ketogenic diet rationale; The common element of these different approaches is variable reduction in the amount of carbohydrate with appropriate increase in fat. Diets that produce a state of ketosis are referred to as ''ketogenic" When deprived of glucose through restriction of carbohydrate intake, the human body begins metabolizing fat.In doing so, ketone bodies (acetoacetate, acetone, and hydroxybutyrate) are produced . (22). KDT is associated with increased mitochondrial biogenesis, oxidative phosphorylation, enhanced gamma‐aminobutyric acid (GABA) levels, reduced neuronal excitability and firing, and stabilized synaptic function (23) Indicated Dietary therapy has been reported as effective in the treatment of seizures associated with glucose transporter 1 deficiency, pyruvate dehydrogenase deficiency, infantile spasms, absence epilepsy, myoclonic atonic epilepsy (Doose syndrome), Dravet syndrome, tuberous sclerosis complex, mitochondrial disorders, Lennox‐Gastaut syndrome, Sturge‐Weber syndrome, and Rett syndrome (24) Results from the randomized control trial indicated that 38% of children had more than 50% reduction in seizure frequency, and 7% had more than 90% reduction in seizure frequency three months after starting classic ketogenic diet. Retrospective studies reported a higher rate of seizure control (25). Contraindications Absolute contraindications for the use of diet therapy include carnitine deficiency (primary), carnitine palmitoyltransferase (CPT) I OR II deficiency, carnitine translocase deficiency, β‐oxidation defects, pyruvate carboxylase deficiency, and porphyria. Antioxidative defense mechanisms are important pathways involving trace elements. The accumulation of free radicals may lead to seizures and increases the risk o f their recurrence, because oxidative stress produces peroxidated membrane lipids and damages the cells Selenium (Se), zinc (Zn), and copper (Cu) are important trace elements that participate in the structure of these enzymes patients with intractable epilepsy had significantly decreased levels of serum Se and Zn in comparison to the controlled group(27)

%Z https://clinicaltrials.gov/show/NCT04542629

Trial registry record

%U https://cochrane.66557.net/central/doi/10.1002/central/CN-02163503/full

%0 Journal Article

%A Asati, P.

%A Tripathi, M.

%A Jain, A.

%A Shukla, S.

%A Dixit, V.

%A Budhiraja, S.

%D 2016

%T Comparative response of combination therapy vs. Mono therapy in steroid dependent ulcerative colitis patients

%V 22

%P S39

%8 2016-01-01

%R 10.1097/01.MIB.0000480201.06783.ab

%K *colitis; *human; *inflammatory bowel disease; *monotherapy; *non profit organization; *patient; *steroid; *therapy; *ulcerative colitis; Azathioprine; Biopsy; Clinical examination; Colonoscopy; Corticosteroid; Disease activity; Disease course; Follow up; Gastroenterology; Histology; India; Informed consent; Intention to treat analysis; Mesalazine; Prospective study; Quality of life; Questionnaire; Recurrence risk; Relapse; Remission; Side effect; Sigmoidoscopy; biopsy; clinical examination; colitis; colonoscopy; disease activity; disease course; follow up; gastroenterology; histology; human; inflammatory bowel disease; informed consent; intention to treat analysis; monotherapy; non profit organization; patient; prospective study; quality of life; questionnaire; recurrence risk; relapse; remission; side effect; sigmoidoscopy; therapy; ulcerative colitis

%X Background: Ulcerative colitis (UC) is an idiopathic inflammatory bowel disease (IBD) characterized by a chronic relapsing/intermittent clinical course. Steroids offer efficient and prompt relief of symptoms in a high proportion of ulcerative colitis (UC). Although most patients initially respond to corticosteroids, after 1 year, approximately 25% become steroid‐dependent. The aim of this prospective study was to assess whether the administration the efficacy of azathioprine (AZA) and mesalamine combination to AZA monotherapy in maintaining remission of steroid‐dependent ulcerative colitis (UC). Methods: The present study was carried out in the Department of Gastroenterology, IMS, BHU, Varanasi, India. A total of 136 patients age sex matched were included in the study after written informed consent fulfilling inclusion and exclusion criteria's. Patients with steroid‐dependent UC in remission were randomized to receive AZA alone (1.5 mg/kg) (group 1) or in combination with mesalamine (800 mg tid) (group 2). Each group had 59 patients. Patients were considered in remission when steroid was withdrawn, an Ulcerative Colitis Clinical Activity Index (UCCAI) score of <2, an Ulcerative Colitis Disease Activity Index (UCDAI) score of 0, and a negative colonoscopy and histology. Follow up was carried out every monthly for 2 year. Clinical examination, hematological and biochemical test were carried out routinely. Sigmoidoscopy and completion of inflammatory bowel disease quality‐of‐life questionnaire (IBD‐Q) and UCDAI every 3 months; and 3) total colonoscopy with biopsies at the end of the study. Results: Out of 136 patients 110 patients were analysed and rest 18 (13.23%) were lost in follow up and 4 patients in each group developed side effects or poor compliance with treatment and were withdrawn from the study. Five patients receiving monotherapy (group 1) relapsed after the first year of the study and 3 after the second year of the study (14.54%). In the combination group (group 2) 2 patients relapsed after the first year of study and 2 after the second year of the study (7.27%) having statistically significant when compared with group 1 (p<0.05). Relapse rates were significant whether analyzed by intention‐to‐treat or per protocol. There were no significant differences between groups in time to relapse or discontinuation of treatment, UCCAI, UCDAI, or IBD‐Q scores. Adverse events were similar both the groups and statistically non significant. Conclusions: Patients with steroid‐dependent UC successfully maintained in remission on AZA and mesalamine and however relapse rate were more common in patients on azathioprine alone.

%Z Inflammatory bowel diseases

Journal article; Conference proceeding

%U https://cochrane.66557.net/central/doi/10.1002/central/CN-01171801/full

%0 Journal Article

%A Chen, M.

%A Shen, B.

%D 2014

%T Comparable short- and long-term outcomes of colonoscopic balloon dilation of Crohn's Disease and benign non-Crohn's Disease strictures

%V 20

%N 10

%P 1739‐1746

%8 2014-01-01

%R 10.1097/MIB.0000000000000145

%K Adult; Catheterization; Colonoscopy; Constriction, Pathologic [mortality, *therapy]; Crohn Disease [mortality, *therapy]; Endoscopy, Gastrointestinal; Female; Follow‐Up Studies; Humans; Male; Middle Aged; Prognosis; Risk Factors; Survival Rate

%X BACKGROUND: The response of Crohn's disease (CD) stricture to endoscopic therapy compared with non‐CD stricture is unknown. Our aim was to compare the short‐ and long‐term outcomes of endoscopic management of those strictures. METHODS: All eligible patients with benign non‐CD strictures who underwent the endoscopic balloon dilation between January 2002 and September 2013 were included. Patients with CD strictures were randomly selected with a ratio (CD versus non‐CD strictures) of 2:1. RESULTS: A total of 90 patients were included, including 30 (33.3%) with non‐CD strictures and 60 (66.7%) with CD strictures. Patients with CD strictures were younger than those with non‐CD strictures at the time of disease diagnosis (25.8 ± 11.1 versus 50.5 ± 17.5; P < 0.001) and at the time of the first dilation (43.9 ± 12.4 versus 55.8 ± 13.9; P < 0.001). There were no significant differences in characteristics of strictures and their endoscopic treatments between the CD and non‐CD groups, except for the percentage of patients who were ever treated with intralesional corticosteroid injection (25.0% versus 6.7%, P = 0.046). Patients in the 2 groups had similar technical success rates (94.0% versus 93.9%, P = 1.00). Few patients in CD stricture group required emergency room visits due to obstructive symptoms from recurrence of disease (1.7% versus 16.7%, P = 0.02). There were on procedure‐related complications in either of the 2 groups. CONCLUSIONS: The efficacy and safety of endoscopic balloon dilation in the treatment of CD and non‐CD strictures seemed to be comparable.

%Z Inflammatory bowel diseases

Journal article

%U https://cochrane.66557.net/central/doi/10.1002/central/CN-01114260/full

%0 Journal Article

%A Zhu, P.

%A Yang, B. L.

%D 2018

%T Combination therapy for perianal fistulising Crohn's disease in biological era: what is the optimal time for surgical intervention?

%V 12

%N Supplement 1

%P S439‐S440

%8 2018-01-01

%K *Crohn disease; *biological product; Abscess; Adult; Chinese medicine; Cohort analysis; Conference abstract; Controlled clinical trial; Controlled study; Crohn disease; Drug combination; Drug therapy; Female; Follow up; Human; Infliximab; Infusion; Kaplan Meier method; Log rank test; Major clinical study; Male; Recurrence risk; Remission; Retrospective study; Risk factor; Stenosis; Surgery; abscess; adult; cohort analysis; conference abstract; controlled clinical trial; controlled study; drug combination; drug therapy; female; follow up; human; infusion; log rank test; major clinical study; male; recurrence risk; remission; retrospective study; risk factor; stenosis; surgery

%X Background: Management of complex perianal fistulising Crohn's disease (pCD) continues to be challenging. Recent studies have recognised that combined surgery and anti‐TNFa therapy could improve clinical outcomes in patients with pCD. However, the optimal timing of surgical intervention after infliximab infusion is still controversial. The aim of the present study was to determine the long‐term efficacy of the treatment strategy with early surgical intervention based on infliximab therapy for pCD. Methods: In this retrospective cohort study, consecutive patients seen at Affiliated Hospital of Nanjing University of Chinese Medicine, which received combined infliximab and surgical treatment between July 2010 and January 2017 for active pCD were included. Patients received surgical intervention during the induction infliximab therapy (time interval < 6 weeks) were grouped into the do‐not‐wait (DNW) cohort, while with time interval >6 weeks were grouped into the do‐wait (DW) cohort. The long‐term recurrence rate was compared using Kaplan‐Meier curves and log‐rank tests. Cox proportion hazard model was used to identify risk factors for the development of recurrence in DNW cohort. Results: One hundred and seventeen patients were included and followed‐up for a median of 36.0 (IQR 23.5‐58.5) months. Among them, 73 patients and 44 patients were grouped into DNW cohort and DW cohort, respectively. There was no significant difference in baseline characteristics between two cohorts. The median interval between surgery and initial infliximab infusion was 9.0 (IQR 5.5‐17.0) days in DNW cohort and 188.0 (IQR 102.25‐455.75) days in DW group. Greater percentages of patients in the DNW cohort had anorectal stenosis compared with patients in the DW cohort (37% vs. 20.5%, p = 0.061). At the end of follow‐up, 61.6% of patients in DNW group and 65.9% patients in DW group derived clinical remission respectively (p = 0.643). The cumulative recurrence rate after perianal surgery was 23%, 32%, 35% in DNW group and 16%, 24%, 24% in DW group, at 1, 2, and 3 years, respectively (p = 0.162). In DNW cohort, the presence of concurrent abscess at baseline (HR: 3.802; 95% CI 1.413‐10.229; p = 0.008) and maintenance infliximab therapy >3 infusions (HR: 2.356; 95% CI 1.012‐5.488; p = 0. 047) predicted the development of recurrence. (Figure presented) Conclusions: Based on infliximab therapy, early surgical intervention could provide long‐term benefits for pCD. The presence of concurrent abscess and maintenance infliximab therapy more than three times were risk factors for recurrence with early surgical intervention.

%Z Journal of Crohn's & colitis

Journal article; Conference proceeding

%U https://cochrane.66557.net/central/doi/10.1002/central/CN-01466783/full

%0 Journal Article

%A Bentz, S.

%A Hausmann, M.

%A Piberger, H.

%A Kellermeier, S.

%A Paul, S.

%A Held, L.

%A Falk, W.

%A Obermeier, F.

%A Fried, M.

%A Schölmerich, J.

%A Et, Al.

%D 2010

%T Clinical relevance of IgG antibodies against food antigens in Crohn's disease: a double-blind cross-over diet intervention study

%V 81

%N 4

%P 252‐264

%8 2010-01-01

%R 10.1159/000264649

%K Abdominal Pain [physiopathology]; Adolescent; Adult; Analysis of Variance; Antibodies, Anti‐Idiotypic [*immunology]; Crohn Disease [blood, *diet therapy, *immunology]; Cross‐Over Studies; Defecation [physiology]; Disease Progression; Double‐Blind Method; Enzyme‐Linked Immunosorbent Assay; Eosinophil‐Derived Neurotoxin [analysis, immunology]; Feces; Female; Food; Food Hypersensitivity [*immunology]; Health Status; Humans; Immunoglobulin G [*immunology]; Male; Middle Aged; Pilot Projects; Probability; Prognosis; Recurrence; Reference Values; Risk Assessment; Treatment Outcome; Young Adult

%X BACKGROUND: Environmental factors are thought to play an important role in the development of Crohn's disease (CD). Immune responses against auto‐antigens or food antigens may be a reason for the perpetuation of inflammation. METHODS: In a pilot study, 79 CD patients and 20 healthy controls were examined for food immunoglobulin G (IgG). Thereafter, the clinical relevance of these food IgG antibodies was assessed in a double‐blind cross‐over study with 40 patients. Based on the IgG antibodies, a nutritional intervention was planned. The interferon (IFN)gamma secretion of T cells was measured. Eosinophil‐derived neurotoxin was quantified in stool. RESULTS: The pilot study resulted in a significant difference of IgG antibodies in serum between CD patients and healthy controls. In 84 and 83% of the patients, respectively, IgG antibodies against processed cheese and yeast were detected. The daily stool frequency significantly decreased by 11% during a specific diet compared with a sham diet. Abdominal pain reduced and general well‐being improved. IFNgamma secretion of T cells increased. No difference for eosinophil‐derived neurotoxin in stool was detected. CONCLUSION: A nutritional intervention based on circulating IgG antibodies against food antigens showed effects with respect to stool frequency. The mechanisms by which IgG antibodies might contribute to disease activity remain to be elucidated.

%Z Digestion

Journal article

%U https://cochrane.66557.net/central/doi/10.1002/central/CN-00752303/full

%0 Journal Article

%A Cong, L-L

%A Yao, J-Y

%A Wu, Y-J

%A Lyu, Y-H

%D 2021

%T Clinical Effect of Changyanqing Mixture Combined with Mesalazine Enteric-coated Tablets for Maintenance Treatment of Ulcerative Colitis in Remission Period

%V 27

%N 3

%P 99‐104

%8 2021-01-01

%R 10.13422/j.cnki.syfjx.20202324

%K *enteric coated tablet; *feces; *histology; *maintenance therapy; *remission; *ulcerative colitis; Adult; Article; Controlled study; Drug safety; Female; Follow up; Histopathology; Human; Human tissue; Inflammatory bowel disease; Lifestyle; Major clinical study; Male; Quality of life; Recurrence risk; Simulation

%X Objective：This study aims to evaluate the clinical effect of Changyanqing mixture combined with mesalazine enteric‐coated tablets in the maintenance treatment of ulcerative colitis（UC）in remission period. Method： The 140 patients with UC in remission period were randomly divided into control group（70 cases） and observation group（70 cases）. The 61 patients in control group completed the therapy（6 cases lost or lost to follow‐up and 3 were eliminated），63 patients in observation group completed the therapy（5 cases lost or lost to follow‐up and 2 were eliminated）. Both groups ′patients got treatment of lifestyle adjustment，and they also took mesalazine enteric‐coated tablets orally， 0.5 g/time， 3 times/day. Patients in observation group took Changyanqing mixture orally for a month in the morning and evening every day，150 mL/time，and then changed to 150 mL/time，1 time/day，for 3 consecutive months，finally changed to once every other day for 8 months. Patients in control group took simulated medicine of Changyanqing mixture orally in the same way as observation group. The treatment was continued for 12 months. When UC recurred during the treatment，patients took mesalazine enteric‐coated tablets orally at 1 g/time， 3 times/day until remission， when the above intervention plan was continued to be adopted. The recurrence rate，first recurrence time within 12 months （duration from remission to Mayo≥3）and the degree of disease activity at recurrence were recorded. Scores of traditional Chinese medicine（TCM）syndrome and inflammatory bowel disease questionnaire（IBDQ）were evaluated once every 2 months. Before treatment，and at the 6th and 12th month after treatment，colonoscopy and mucosal histology were performed once，enteroscopic mucosal scores，Geboes index of mucosal histology were evaluated，and fecal calprotectin（FC）levels were detected. Also，safety evaluation was conducted. Result： During 12 months，the recurrence rate in observation group was 20.63%（13/63），lower than 39.34%（24/61）in control group（P<0.05），the frequency of recurrence and the first recurrence duration in observation group were all less than those in control group（P<0.01）. All these meant the disease activity of patients in observation group was lighter than that in control group（χ2=5.947，P<0.05）. After repeated measurements of variance analysis， scores of TCM syndrome，enteroscopic mucosal scores，Geboes index and FC levels in two groups gradually increased（P<0.05），and scores of IBDQ gradually decreased（P<0.05）during the 12‐month period. At the second，fourth，sixth，eighth，tenth and twelfth month，scores of TCM syndromes in observation group were lower than those in control group（P<0.01），and scores of IBDQ were higher than those in control group（P< 0.01）. At the sixth and twelfth month after treatment，intestinal endoscopic mucosal scores，Geboes index and FC levels in observation group were all lower than those in control group（P<0.01）. And there were no adverse reactions related to Changyanqing mixture. Conclusion： Changyanqing mixture combined with mesalazine enteric‐coated tablets in the maintenance treatment of patients with UC in remission can control the FC level， further reduce the recurrence rate，delay the recrudescence‐time，reduce the frequency of UC and the disease activity，maintain the good remission state of UC，stabilize the quality of life of patients，and ensure the safety of clinical use.

%Z Chinese journal of experimental traditional medical formulae

Journal article

%U https://cochrane.66557.net/central/doi/10.1002/central/CN-02567979/full

%0 Journal Article

%A Laharie, D.

%A Bourreille, A.

%A Branche, J.

%A Allez, M.

%A Bouhnik, Y.

%A Filippi, J.

%A Zerbib, F.

%A Savoye, G.

%A Nachury, M.

%A Moreau, J.

%A Et, Al.

%D 2012

%T Ciclosporin versus infliximab in patients with severe ulcerative colitis refractory to intravenous steroids: a parallel, open-label randomised controlled trial

%V 380

%N 9857

%P 1909‐1915

%8 2012-01-01

%R 10.1016/S0140-6736(12)61084-8

%K Acute Disease; Adult; Antibodies, Monoclonal [*therapeutic use]; Anti‐Inflammatory Agents, Non‐Steroidal [*therapeutic use]; Colitis, Ulcerative [*drug therapy]; Cyclosporine [*therapeutic use]; Drug Resistance; Female; Humans; Immunosuppressive Agents [*therapeutic use]; Infliximab; Infusions, Intravenous; Male; Middle Aged; Steroids [*administration & dosage]; Treatment Failure

%X BACKGROUND: Ciclosporin and infliximab are potential rescue treatments to avoid colectomy in patients with acute severe ulcerative colitis refractory to intravenous corticosteroids. We compared the efficacy and safety of these drugs for this indication. METHODS: In this parallel, open‐label, randomised controlled trial, patients were aged at least 18 years, had an acute severe flare of ulcerative colitis defined by a Lichtiger score greater than 10 points, and had been given an unsuccessful course of high‐dose intravenous steroids. None of the patients had previously received ciclosporin or infliximab. Between June 1, 2007, and Aug 31, 2010, patients at 27 European centres were randomly assigned (via computer‐derived permutation tables; 1:1) to receive either intravenous ciclosporin (2 mg/kg per day for 1 week, followed by oral drug until day 98) or infliximab (5 mg/kg on days 0, 14, and 42). In both groups, azathioprine was started at day 7 in patients with a clinical response. Neither patients nor investigators were masked to study treatment. The primary efficacy outcome was treatment failure defined by absence of a clinical response at day 7, a relapse between day 7 and day 98, absence of steroid‐free remission at day 98, a severe adverse event leading to treatment interruption, colectomy, or death. Analysis was by intention to treat. This trial is registered with EudraCT (2006‐005299‐42) and ClinicalTrials.gov (NCT00542152). FINDINGS: 115 patients were randomly assigned; 58 patients were allocated to receive ciclosporin and 57 to receive infliximab. Treatment failure occurred in 35 (60%) patients given ciclosporin and 31 (54%) given infliximab (absolute risk difference 6%; 95% CI ‐7 to 19; p=0·52). Nine (16%) patients in the ciclosporin group and 14 (25%) in the infliximab group had severe adverse events. INTERPRETATION: Ciclosporin was not more effective than infliximab in patients with acute severe ulcerative colitis refractory to intravenous steroids. In clinical practice, treatment choice should be guided by physician and centre experience. FUNDING: Association François Aupetit, Société Nationale Française de Gastroentérologie, and the International Organization for the study of Inflammatory Bowel Disease.

%Z Lancet (london, england)

Journal article

%U https://cochrane.66557.net/central/doi/10.1002/central/CN-00839275/full

%0 Journal Article

%A Kelly, C.

%A Wilcox, M.

%A Glerup, H.

%A Aboo, N.

%A Eves, K.

%A Mary Beth, D.

%D 2017

%T Characteristics and outcomes in patients with C. Difficile infection and inflammatory bowel disease: bezlotoxumab versus placebo

%V 5

%N 5

%P A306

%8 2017-01-01

%R 10.1177/2050640617725676

%K *Clostridium difficile infection; *Crohn disease; Adult; Cohort analysis; Controlled study; Diagnosis; Drug therapy; Feces analysis; Female; Human; Incidence; Infusion; Major clinical study; Male; Outpatient; Post hoc analysis; Prevention; Randomized controlled trial; Relapse; Ulcerative colitis

%X Introduction: Patients with inflammatory bowel disease (IBD) experience higher rates of C. difficile infection (CDI) than the overall population, often lack typical risk factors, and frequently experience severe and recurrent episodes. MODIFY I/II were global trials of the efficacy and safety of bezlotoxumab (bezlo: a human monoclonal antibody against C. difficile toxin B), in which bezlo was superior to placebo at preventing CDI recurrence (rCDI) in participants with primary or recurrent CDI given antibacterial drug treatment for CDI. Participants with IBD could be enrolled if, in the opinion of the investigator, symptoms were more likely due to CDI than IBD. Aims & Methods: The objective of this post‐hoc subgroup analysis was to summarize CDI‐related outcomes, including initial clinical cure and rCDI, through 12 weeks in participants with IBD enrolled in the MODIFY trials. CDI‐related outcomes through 12 weeks in the subset of IBD participants enrolled in the MODIFY trials included: initial clinical cure (no diarrhea during the 2 consecutive days following completion of ≤14 days of an antibacterial drug treatment for CDI); and rCDI (new episode of diarrhea associated with a positive stool test for toxigenic C. difficile in participants who had achieved initial clinical cure). for this post‐hoc analysis, participants randomized to bezlo or actoxumab+bezlo were pooled and are referred to as the “bezlo” group and participants randomized to placebo or actoxumab were pooled and are referred to as the “no bezlo” group. Results: Overall, 2559 participants were included in the mITT population; 1554 participants were randomized to a bezlo group and 1005 were randomized to a no bezlo group. There were 44 participants with IBD: 23 (52.3%) had ulcerative colitis; 18 (40.9%) had Crohn's disease; and 3 (6.8%) had non‐characterized IBD. Compared with participants without IBD, participants with IBD tended to be younger, were more often treated as outpatients, were more often immunocompromised, and a smaller percentage had severe CDI. Among IBD participants, a higher proportion had initial clinical cure in the no bezlo group compared with the bezlo group and there was a higher proportion of participants with rCDI in the no bezlo group compared with the bezlo group. In IBD participants who did not receive bezlo, most of the recurrences (5 of 7) occurred within 4 weeks after study infusion, while most of the recurrences among participants who received bezlo occurred after week 4 (3 of 4). Conclusion: Participants with IBD and CDI enrolled in the MODIFY trials were younger, more likely to be diagnosed with CDI as an outpatient, to be immunocompromised, and to develop rCDI compared with non‐IBD participants. Bezlo yielded a 27.2% absolute reduction (50% relative reduction) in the incidence of rCDI in participants with IBD. The efficacy of bezlo in preventing rCDI may extend to patients with IBD, but additional data are needed due to the limited cohort size.

%Z United European gastroenterology journal

Journal article; Conference proceeding

%U https://cochrane.66557.net/central/doi/10.1002/central/CN-01439106/full

%0 Journal Article

%A Kelly, C. P.

%A Wilcox, M.

%A Glerup, H.

%A Aboo, N.

%A Eves, K.

%A Dorr, M. B.

%D 2017

%T Characteristics and outcomes in patients with C. difficile infection (CDI) and inflammatory bowel disease: bezlotoxumab versus placebo

%V 152

%N 5

%P S340

%8 2017-01-01

%K *Clostridium difficile infection; *Crohn disease; Adult; Antibiotic therapy; Cohort analysis; Controlled study; Diagnosis; Drug therapy; Feces analysis; Female; Follow up; Hospital patient; Human; Incidence; Infusion; Major clinical study; Male; Outpatient; Post hoc analysis; Prevention; Randomization; Randomized controlled trial; Recipient; Ulcerative colitis

%X Background: Patients with inflammatory bowel disease (IBD) experience higher rates of CDI than the overall population, often lack typical risk factors, and frequently experience severe and recurrent episodes. MODIFY I/II were global trials of the efficacy and safety of bezlotoxumab (bezlo: a human monoclonal antibody against C. difficile toxin B), in which a single 10 mg/kg IV dose of bezlo was superior to placebo at preventing CDI recurrence (rCDI) among subjects with primary or recurrent CDI given standard of care (SoC) antibiotic therapy. Patients with IBD could be enrolled if, in the opinion of the investigator, symptoms were more likely due to CDI than IBD. A post hoc analysis of the IBD subset was conducted. Material/methods: Summary CDI‐related outcomes through 12 weeks in the subset of IBD patients enrolled in the MODIFY trials included: clinical cure (no diarrhoea during the 2 consecutive days following completion of ≤14 days of a SoC antibiotic); and rCDI (new episode of diarrhoea associated with a positive stool test for toxigenic C. difficile in patients who had achieved clinical cure). Results: Overall, 1554 subjects were included in the mITT population of which 21 had a history of IBD; 3 additional subjects received a new diagnosis of IBD on study. 15 had ulcerative colitis, 7 had Crohn's disease, and 2 had unclassified IBD. Characteristics and outcomes of the IBD subset were compared with those of the mITT population (Table). IBD subjects were younger and fewer were inpatients at randomization compared with the mITT population. A lower proportion of IBD subjects achieved clinical cure compared with the mITT population in the bezlo group. rCDI rates were higher in the IBD subset compared with the mITT population (in both placebo and bezlo recipients); notably, a higher proportion of IBD subjects receiving placebo vs bezlo had rCDI. Three placebo IBD subjects who experienced rCDI were hospitalized compared with no bezlo IBD subjects. One placebo‐treated subject with IBD died during 12‐weeks follow‐up. Conclusions: Consistent with prior reports, subjects with IBD and CDI were younger, more likely to be diagnosed as an out‐patient, less likely to respond to SoC antibiotic therapy and more likely to develop rCDI compared with non‐IBD cases. A single infusion of bezlo was associated with a 22.3% absolute reduction in the incidence of rCDI in IBD patients (40% relative reduction). The efficacy of bezlo in preventing rCDI may extend to patients with IBD, but additional data are needed due to the limited cohort size. (Table Presented).

%Z Gastroenterology

Journal article; Conference proceeding

%U https://cochrane.66557.net/central/doi/10.1002/central/CN-01422315/full

%0 Journal Article

%A Jones, G-R

%A Arnott, I.

%A Satsangi, J.

%D 2012

%T Challenges in recruiting to clinical trials in the UK: the toppic experience

%V 61

%P A389

%8 2012-01-01

%R 10.1136/gutjnl-2012-302514d.224

%K *United Kingdom; *clinical trial (topic); *digestive system function disorder; Controlled study; Crohn disease; Diagnosis; Follow up; Human; Hypersensitivity; Intestine resection; Ischemic heart disease; Medical research; Mercaptopurine; Mortality; Pancreatitis; Patient; Placebo; Prevention; Randomization; Risk; Screening; Small intestine; Stoma; Surgery; United Kingdom; clinical trial (topic); controlled study; diagnosis; digestive system function disorder; follow up; human; hypersensitivity; intestine resection; ischemic heart disease; medical research; mortality; pancreatitis; patient; prevention; randomization; risk; screening; small intestine; stoma; surgery

%X Introduction: The Trial of Prevention of Post‐operative Crohn's disease (TOPPIC) is a multi‐centre, randomised, Medical Research Council (MRC) funded, controlled trial of mercaptopurine (MP) vs placebo in preventing post‐operative recurrence. It is the largest and only double‐blinded trial conducted to date assessing thiopurine treatment in this setting. To complete the study in five centres we assumed that 60% of eligible patients would be enrolled into the study. Our experience of the challenges to recruitment has relevance to all other clinical trials in the area. Methods: Patients undergoing intestinal resection for Crohn's disease (CD) were prospectively recruited and randomised (1:1) within 3 months of surgery to either placebo or 1‐1.5 mg/kg/day MP. 234 patients are sought to detect a 20% difference with 80% power between the two groups. The primary outcome means to assess the ability of MP to delay or prevent post‐operative recurrence. This is assessed clinically at 12 study visits over 36 months and endoscopically at 12 and 36 months. Recruitment is due to finish in 2012. Results: The initial enrolment of patients was disappointing with only 60 patients recruited at 22 months (predicted 180 patients). Within the study centres anti‐TNF use increased over the same period fivefold (p The study was therefore extended for a further 18 months from 5 Scottish centres, to involve 25 new sites across England and Wales. As of February 2012 223 patients have been randomised representing 20.5% of the 1085 patients undergoing resection at participating centres. Of those not randomised, 530 (49%) were ineligible and 243 (22%) declined pre‐screening, 71 (6%) were ineligible/declined during screening. Of the 530 patients ineligible pre‐screening 158 (30%) had a stoma, 110 (21%) were not included for reasons not specified, 55 (10%) had a condition the clinician felt placed them at unacceptable risk, 53 (10%) had MP hypersensitivity, 32 (6%) had no known diagnosis of CD, 28 (5%) failed to have ileocolonic resection within 3 months, 19 (4%) had active or untreated malignancy. The remainder 14% consisted of multiple other factors; including previous pancreatitis, and length small bowel resected. Of the 44 randomised patients whom have since dropped out since randomisation; 23 withdrew early from the trial, 13 were lost to follow‐up, six other reasons not specified and one mortality from coronary heart disease. Conclusion: Our experience illustrates a number of challenges in investigator lead studies in IBD. Although we had accurately predicted the number of resections our initial projections had dramatically underestimated the proportion of patients willing or eligible to participate in a placebo controlled study. This study has however, highlighted the merits of multi‐centre collaboration, not least due to the acceptance onto the NIHR portfolio.

%Z Gut

Journal article; Conference proceeding

%U https://cochrane.66557.net/central/doi/10.1002/central/CN-01025974/full

%0 Journal Article

%A Schwartz, D.

%D 2005

%T Can we reset the immunostat? A randomized, double-blind, controlled withdrawal trial in Crohn's disease patients win long-term remission taking azathioprine

%V 6

%N 4

%P 114‐115

%8 2005-01-01

%R 10.1097/01.ebg.0000194916.36808.2e

%K *Crohn disease /drug therapy; *remission; Clinical trial; Controlled study; Crohn disease activity index score; Double blind procedure; Drug withdrawal; Human; Long term care; Major clinical study; Multicenter study; Note; Outcomes research; Randomized controlled trial; Relapse; Risk assessment; Scoring system

%Z Evidence-based gastroenterology

Journal article

%U https://cochrane.66557.net/central/doi/10.1002/central/CN-01740164/full

%0 Journal Article

%A EUCTR CZ

%D 2009

%T Biological therapy in the prevention of Crohn`s disease recurrence after resection in ileocecal region

%8 2009-01-01

%X INTERVENTION: Trade Name: Remicade Pharmaceutical Form: Powder for injection* INN or Proposed INN: INFLIXIMAB CAS Number: 170277313 Concentration unit: mg/kg milligram(s)/kilogram Concentration type: equal Concentration number: 5‐ Pharmaceutical form of the placebo: Solution for infusion Route of administration of the placebo: Intravenous use CONDITION: Prevention of Crohn`s disease recurrence after surgery in ileocecal region by postoperative application of infliximab (four infusions). ; MedDRA version: 9.1 Level: LLT Classification code 10011401 Term: Crohn's disease PRIMARY OUTCOME: Main Objective: Aim of the prospective randomized placebo controlled study is to prove the efficacy of biological therapy (Infliximab) in prevention of Crohn's disease recurrence in 24 months (defined as endoscopic recurrence ‐ in endoscopic score of i2 or more) after surgery in ileocecal region Primary end point(s): incidence of endoscopic recurrence in 24 months after surgery, rate of clinical relapse, risk factors for development of recurrence, quality of life, incidence of repeated hospitalization eventually another surgery Secondary Objective: Secondary aim is to compare the rate of clinical relapse in both groups (defined as a increase in CDAI score of 75 points). To design algorithm of disease recurrence monitoring in terms of multi specialty cooperation (surgeon, gastroenterologist, radiologist, immunologist) using new non‐invasive techniques (biological markers, biochemistry markers, NMR‐ enterography, ultrasound) including calculation of costs. INCLUSION CRITERIA: ‐ Suitable patients for the study are all patients admitted for surgery due to ileocecal Crohn's disease, who have never been treated by any biological therapy (naive) ‐ age 18‐65years ‐ no signs of system infection (viral hepatitis) ‐ negative chest X‐ray and Quantiferron test for TBC ‐ no signs of heart failure Are the trial subjects under 18? no Number of subjects for this age range: F.1.2 Adults (18‐64 years) yes F.1.2.1 Number of subjects for this age range F.1.3 Elderly (>=65 years) no F.1.3.1 Number of subjects for this age range

%Z https://trialsearch.who.int/Trial2.aspx?TrialID=EUCTR2008-005688-32-CZ

Trial registry record

%U https://cochrane.66557.net/central/doi/10.1002/central/CN-02067468/full

%0 Journal Article

%A Treton, X.

%A Bouhnik, Y.

%A Mary, J. Y.

%A Colombel, J. F.

%A Duclos, B.

%A Soule, J. C.

%A Lerebours, E.

%A Cosnes, J.

%A Lemann, M.

%D 2009

%T Azathioprine withdrawal in patients with Crohn's disease maintained on prolonged remission: a high risk of relapse

%V 7

%N 1

%P 80‐85

%8 2009-01-01

%R 10.1016/j.cgh.2008.08.028

%K Adult; Azathioprine [*therapeutic use]; Cohort Studies; Crohn Disease [*drug therapy]; C‐Reactive Protein [analysis]; Female; Follow‐Up Studies; Hemoglobins [analysis]; Humans; Immunosuppressive Agents [*therapeutic use]; Leukocyte Count; Male; Middle Aged; Recurrence; Risk Factors; Withholding Treatment

%X BACKGROUND & AIMS: Azathioprine (AZA) withdrawal in Crohn's disease after long‐term remission under treatment is controversial. In a Groupe d'Etude Thérapeutique des Affections Inflammatoires du tube Digestif randomized, double‐blind, placebo‐controlled trial, the hypothesis that AZA withdrawal was not inferior to AZA continuation in patients in prolonged clinical remission could not be shown. METHODS: A cohort of 66 patients in prolonged remission while being treated with AZA who stopped AZA, during or at the end of the randomized controlled trial, underwent long‐term follow‐up evaluation. The primary end point was clinical relapse. Prognostic factors of relapse were looked for through a proportional hazards model. RESULTS: Median durations of AZA therapy and of clinical remission were 68.4 months (interquartile range, 52.8‐85.2 mo) and 63.6 months (interquartile range, 48.0‐55.7 mo), respectively. The median follow‐up time after AZA interruption was 54.5 months; 32 of 66 patients had a relapse. The cumulative probabilities +/‐ SE of relapse at 1, 3, and 5 years were 14.0% +/‐ 4.3%, 52.8% +/‐ 7.1%, and 62.7% +/‐ 7.2%, respectively. C‐reactive protein concentration of 20 mg/L or greater (risk, 58.6; 95% confidence interval, 7.5‐457; P = .002), hemoglobin level less than 12 g/dL (risk, 4.8; 95% confidence interval, 1.7‐13.7; P = .04), and neutrophil count 4 x 10(9)/L or greater (risk, 3.2; 95% confidence interval, 1.6‐6.3; P = .003) were associated independently with an increased risk of relapse. Among the 32 relapsing patients, 23 were retreated by AZA alone, all but 1 up to successful remission. CONCLUSIONS: Our results confirm that AZA withdrawal is associated with a high risk of relapse, whatever the duration of remission under this treatment. These data suggest that if AZA is well tolerated, it should not be interrupted.

%Z Clinical gastroenterology and hepatology

Journal article

%U https://cochrane.66557.net/central/doi/10.1002/central/CN-00666625/full

%0 Journal Article

%A Orlando, A.

%A Mocciaro, F.

%A Ventimiglia, M.

%A Renna, S.

%A Scimeca, D.

%A Rispo, A.

%A Scribano, M. L.

%A Testa, A.

%A Aratari, A.

%A Bossa, F.

%A Et, Al.

%D 2019

%T Azathioprine vs. mesalamine for prevention of post-operative clinical relapse in Crohn's disease patients with severe endoscopic recurrence: data on efficacy and safety from an IG-IBD multi-centre randomised double-blind double-dummy trial

%V 13

%P S381

%8 2019-01-01

%R 10.1093/ecco-jcc/jjy222.659

%K *Crohn disease; *drug safety; *prognosis; *relapse; *visually impaired person; Adult; Clinical article; Conference abstract; Controlled study; Double blind procedure; Drug megadose; Drug therapy; Female; Human; Male; Patient history of surgery; Pharmacokinetics; Prevention; Prophylaxis; Randomization; Randomized controlled trial; Risk factor; Smoking; Surgery; Surgical risk

%X Background: More than 70% of patients with Crohn's disease (CD) require surgery at least once during the course of their disease. Unfortunately endoscopic recurrence (ER) is up to 100% at 5 years with a risk of six‐month severe ER (≥ i2) around 50% as showed in a previous Italian study. As well know symptomatic recurrence is strongly related to the severity of ER and ECCO guideline recommended prophylactic treatment after ileocolonic resection despite effectiveness of immunosuppressants remain debated. Methods: We performed a multi‐centre randomised double‐blind double‐dummy trial to assess the role of azathioprine (AZA) vs. high dose of mesalamine (5‐ASA) as treatment of early severe POR (Rutgeerts' score ≥ i2) and as prophylaxis for clinical relapse (eligible patients and treatment allocation are showed in Table 1). Primary outcomes: endoscopic improvement and clinical relapse after 12 months from randomisation. Post‐trial analysis: data on clinical and endoscopic outcomes up to 10 years from T0. Results: According to inclusion/exclusion criteria 46 patients were randomised (characteristics of screened patients are showed in Table 1): 65% males, overall median age at diagnosis and at surgery of 29.5 and 36.5 years, respectively. At the final analysis 17% of patients experienced a clinical relapse within 12 months from randomisation without differences between AZA and 5‐ASA groups. Considering POR after 12 months of treatment no significant improvement were observed from T0 in both groups (p = ns). At the post‐trial analysis, 53% of patients experienced a clinical relapse without differences between those previously treated with AZA or 5‐ASA (p = ns). Smoking and previous surgery at T0 were risk factors for clinical relapse (p = 0.031 and 0.003). No significant AE were recorded. Conclusions: This multi‐centre RCT does not show efficacy of AZA or 5‐ASA in the treatment of severe POR or as prophylaxis for clinical relapse. In the post‐trial analysis, in those with POR at 6‐month from surgery, risk factors for severe CD (smoking and multiple surgery) could help to identify patients with worse prognosis to start biological therapy. (Table Presented) .

%Z Journal of Crohn's & colitis

Journal article; Conference proceeding

%U https://cochrane.66557.net/central/doi/10.1002/central/CN-01959185/full

%0 Journal Article

%A Reinisch, W.

%A Angelberger, S.

%A Petritsch, W.

%A Shonova, O.

%A Lukas, M.

%A Bar-Meir, S.

%A Teml, A.

%A Schaeffeler, E.

%A Schwab, M.

%A Dilger, K.

%A Et, Al.

%D 2010

%T Azathioprine versus mesalazine for prevention of postoperative clinical recurrence in patients with Crohn's disease with endoscopic recurrence: efficacy and safety results of a randomised, double-blind, double-dummy, multicentre trial

%V 59

%N 6

%P 752‐759

%8 2010-01-01

%R 10.1136/gut.2009.194159

%K Adult; Anti‐Inflammatory Agents, Non‐Steroidal [*therapeutic use]; Azathioprine [adverse effects, *therapeutic use]; Colonoscopy; Crohn Disease [*prevention & control, surgery]; Double‐Blind Method; Female; Humans; Immunosuppressive Agents [*therapeutic use]; Male; Mesalamine [adverse effects, *therapeutic use]; Methyltransferases [blood]; Middle Aged; Patient Selection; Secondary Prevention; Severity of Illness Index; Treatment Outcome; Young Adult

%X OBJECTIVE: The aim of the study was to compare azathioprine versus mesalazine tablets for the prevention of clinical recurrence in patients with postoperative Crohn's disease (CD) with moderate or severe endoscopic recurrence. METHODS: This was a 1 year, double‐blind, double‐dummy, randomised study which took place in 21 gastroenterology centres in Austria, the Czech Republic, Germany and Israel. The study participants were 78 adults with CD who had undergone resection with ileocolonic anastomosis in the preceding 6‐24 months without subsequent clinical recurrence and with a Crohn's disease activity index (CDAI) score <200, but with moderate or severe endoscopic recurrence. The study drugs were azathioprine 2.0‐2.5 mg/kg/day or mesalazine 4 g/day over 1 year. The primary end point was therapeutic failure during 1 year, defined as a CDAI score > or = 200 and an increase of > or = 60 points from baseline, or study drug discontinuation due to lack of efficacy or intolerable adverse drug reaction. RESULTS: Treatment failure occurred in 22.0% (9/41) of azathioprine‐treated patients and 10.8% (4/37) of mesalazine‐treated patients, a difference of 11.1% (95% CI ‐5.0% to 27.3%, p=0.19). Clinical recurrence was significantly less frequent with azathioprine versus mesalazine (0/41 (0%) vs 4/37 (10.8%), p=0.031), whereas study drug discontinuation due to adverse drug reactions only occurred in azathioprine‐treated patients (9/41 (22.0%) vs 0%, p=0.002). The proportion of patients showing > or = 1 point reduction in Rutgeerts score between baseline and month 12 was 63.3% (19/30) and 34.4% (11/32) in the azathioprine and mesalazine groups, respectively (p=0.023). CONCLUSIONS: In this population of patients with postoperative CD at high risk of clinical recurrence, superiority for azathioprine versus mesalazine could not be demonstrated for therapeutic failure.

%Z Gut

Journal article

%U https://cochrane.66557.net/central/doi/10.1002/central/CN-00749652/full

%0 Journal Article

%A Ardizzone, S.

%A Maconi, G.

%A Sampietro, G. M.

%A Russo, A.

%A Radice, E.

%A Colombo, E.

%A Imbesi, V.

%A Molteni, M.

%A Danelli, P. G.

%A Taschieri, A. M.

%A Et, Al.

%D 2004

%T Azathioprine and mesalamine for prevention of relapse after conservative surgery for Crohn's disease

%V 127

%N 3

%P 730‐740

%8 2004-01-01

%R 10.1053/j.gastro.2004.06.051

%K Adolescent; Adult; Aged; Anti‐Inflammatory Agents, Non‐Steroidal [*administration & dosage]; Azathioprine [*administration & dosage]; Crohn Disease [*prevention & control, surgery]; Digestive System Surgical Procedures [methods]; Female; Humans; Immunosuppressive Agents [*administration & dosage]; Male; Mesalamine [*administration & dosage]; Middle Aged; Postoperative Period; Prospective Studies; Secondary Prevention; Treatment Outcome

%X BACKGROUND & AIMS: Because the reoperation rate for Crohn's disease is high after resective surgery, use of conservative surgery has increased. Mesalamine was investigated for the prevention of postoperative relapse, with disappointing results. The role of azathioprine in the postoperative setting is unknown. We aimed to compare the efficacy and safety of azathioprine and mesalamine in the prevention of clinical and surgical relapse in patients who have undergone conservative surgery for Crohn's disease. METHODS: In a prospective, open‐label, randomized study, 142 patients received azathioprine (2 mg. kg ‐1. day ‐1 ) or mesalamine (3 g/day) for 24 months. Clinical relapse was defined as the presence of symptoms with a Crohn's Disease Activity Index score >200 and surgical relapse as the presence of symptoms refractory to medical treatment or complications requiring surgery. RESULTS: After 24 months, the risk of clinical relapse was comparable in the azathioprine and mesalamine groups, both on intention‐to‐treat (odds ratio [OR], 2.04; 95% confidence interval [CI], 0.89‐4.67) and per‐protocol analyses (OR, 1.79; 95% CI, 0.80‐3.97). No difference was observed with respect to surgical relapse at 24 months between the 2 groups. In a subgroup analysis, azathioprine was more effective than mesalamine in preventing clinical relapse in patients with previous intestinal resections (OR, 4.83; 95% CI, 1.47‐15.8). More patients receiving azathioprine withdrew from treatment due to adverse events than those receiving mesalamine (22% vs. 8%; P = 0.04). CONCLUSIONS: While no difference was observed in the efficacy of azathioprine and mesalamine in preventing clinical and surgical relapses after conservative surgery, azathioprine is more effective in those patients who have undergone previous intestinal resection.

%Z Gastroenterology

Journal article

%U https://cochrane.66557.net/central/doi/10.1002/central/CN-00491333/full

%0 Journal Article

%A Chen, S. Q.

%A Liu, W. C.

%A Zhang, Z. Z.

%A Lin, L. Y.

%A Chen, S. M.

%A Huang, G. L.

%A Lin, C. Z.

%A Wang, L.

%D 2019

%T Application of closed negative pressure irrigation and suction device in the treatment of high perianal abscess

%V 22

%N 4

%P 364‐369

%8 2019-01-01

%R 10.3760/cma.j.issn.1671-0274.2019.04.009

%K *perianal abscess; *suction pump; *therapy effect; Abscess [complications, *surgery]; Adult; Anus Diseases [complications, *surgery]; Anus fistula; Article; Cancer patient; Cancer recurrence; Cancer surgery; Chi square test; Clinical article; Complication; Controlled study; Crohn disease; Curettage; Diabetes mellitus; Drainage; Female; Follow up; Graft recipient; Human; Humans; Incision; Leukemia; Male; Middle Aged; Morbidity; Negative‐Pressure Wound Therapy; Operative blood loss; Physiotherapy; Postoperative pain; Prospective Studies; Prospective study; Randomized controlled trial; Rectal Diseases [complications, surgery]; Rectal Fistula [etiology, prevention & control]; Recurrence risk; Skin; Suction [instrumentation]; Surgery; Therapeutic Irrigation; Treatment Outcome; Vacuum; Visual analog scale; Wound dressing

%X Objective: To explore the efficacy of closed negative pressure irrigation and suction device (Patent number: Z200780013509.8) in the treatment of high perianal abscess. Methods: From January 2015 to December 2016, ≥18‐year‐old patients with primary high perianal abscess who were treated at our department were prospectively enrolled. Exclusion criteria: (1) recurrent perianal abscess; (2) complicated with anal fistula formation; (3) preoperative, intraoperative or postoperative physical therapy, and curettage treatment, negative pressure irrigation; (4) Crohn's disease‐related perianal abscess; (5) with immunosuppressive status, such as transplant recipients; (6) co‐existence of malignant tumors, such as leukemia; (7) with diabetes; (8) those who could not receive long‐term follow‐up and were not suitable to participate in this study. According to the random number table method, the patients were randomly divided into negative pressure irrigation and suction group and routine drainage group. All patients were clearly diagnosed and the location and size of the perianal abscess were marked before surgery. These two groups were treated as follows: (1) Negative pressure irrigation and suction group: the skin was incised at a diameter of 1‐2 cm at the site where the abscess fluctuated most obviously. After the abscess was removed, a closed negative pressure irrigation and suction device was installed and the pressure of ‐200 to ‐100 mmHg (1 mmHg=0.133 kPa) was maintained to keep the abscess cavity collapsed. Generally, the irrigation was stopped 5 days later or when the drainage was clear. The closed vacuum suction was maintained for 2 additional days, before the wound was sutured. (2) Conventional drainage group: conventional incision and drainage was carried out. The skin was cut at a diameter of 8 to 10 cm at the site of abscess with most obvious fluctuation. After the abscess was removed, normal saline gauze was used for dressing. Dressing was changed regularly until the wound healed. The efficacy, operative time, intraoperative bleeding, incision length, frequency of dressing change, pain index (visual analogue score, VAS score), postoperative healing time, complications, recurrence rate of perianal abscess, anal fistula formation rate were observed. The t test and χ2 test were used for comparison between the 2 groups. Results: There were both 40 patients in the negative pressure irrigation and suction group and the conventional drainage group. There were 28 males and 12 females in negative pressure irrigation and suction group with a mean age of (38.3±12.0) years and mean disease course of (6.6±2.1) days. The abscess in pelvic‐rectal space accounted for 50.0% (20/40) and the mean diameter of abscess was (8.0±3.7) cm. There were 26 males and 14 females in the conventional drainage group with a mean age of (37.1±11.8) years and mean disease course of (6.4±2.5) days. The abscess in pelvic‐rectal space accounted for 55.0% (22/40) and the diameter of abscess was (8.2±3.5) cm. The differences in baseline data between two groups were not statistically significant (all P>0.05). Both groups successfully completed the operation. There was no significant difference in operative time between two groups (P>0.05). As compared to conventional drainage group, intraoperative blood loss in negative pressure irrigation and suction group was less [(12.1±5.5) ml vs. (18.3±4.4) ml, t=5.606, P<0.001], incision length was shorter [(2.3±0.8) cm vs. (7.6±1.7) cm, t=17.741, P<0.001], postoperative VAS pain scores at 1‐, 3‐, 7‐, and 14‐day after operation were lower [3.7±1.4 vs. 7.6±1.8, t=10.816, P<0.001; 3.0±1.3 vs. 6.8±1.6, t=11.657, P<0.001; 2.7±0.9 vs. 5.1±1.1, t=10.679, P<0.001; 1.2±0.3 vs. 1.6±0.4, t=5.060, P=0.019], the dressing change within 7 days after operation was less (3.5±1.2 vs. 12.6±2.7, t=19.478, P<0.001), postoperative healing time was shorter [(10.4±3.0) d vs. (13.5±3.8) d, t=4.049, P<0.001] and postoperative complication rate was lower [17.5% (7/40) vs. 2.5% (1/40), χ2=5.000, P=0.025]. During follow‐up of 12 to 36 (24±5) months, the recurrence rate of perianal abscess within 1 year after operation and anal fistula formation rate in negative pressure irrigation and suction group were lower than those in conventional drainage group [5.0% (2/40) vs. 20.0% (8/40), χ2=4.114, P=0.042 and 2.5% (1/40) vs. 17.5% (7/40), χ2=5.000, P=0.025, respectirely]. The one‐time cure rate of negative pressure irrigation and suction group and conventional drainage group was 92.5% (37/40) and 62.5%(25/40), respectirely (χ2=10.323, P=0.001). Conclusions: The application of the negative pressure irrigation and suction device in the treatment of high perianal abscess can improve the efficiency of one‐time cure, reduce postoperative pain, accelerate healing time, decrease the morbidity of postoperative complication and the rates of abscess recurrence and anal fistula formation, indicating an improvement of the treatment.

%Z Zhonghua wei chang wai ke za zhi [Chinese journal of gastrointestinal surgery]

Journal article

%U https://cochrane.66557.net/central/doi/10.1002/central/CN-01937455/full

%0 Journal Article

%A Steinhart, A. H.

%A O'Rourke, K.

%A Wolff, B. G.

%A McLeod, R. S.

%D 1992

%T Application of a stopping rule based on total treatment failures: the postoperative Crohn's disease trial

%V 45

%N 5

%P 495‐504

%8 1992-01-01

%R 10.1016/0895-4356(92)90098-8

%K Aminosalicylic Acids [*therapeutic use]; Computer Simulation; Crohn Disease [*prevention & control, surgery]; Double‐Blind Method; Humans; Mesalamine; Multicenter Studies as Topic; Randomized Controlled Trials as Topic [economics, *methods]; Risk Factors; Time Factors

%X The Postoperative Crohn's Disease Trial (PCDT), a placebo‐controlled randomized trial of Rowasa I in the prevention of postoperative recurrence of Crohn's disease, is used as an example of how a stopping rule based on total endpoint occurrences can provide considerable advantage over standard fixed sample size methods. It can be used when the primary outcome is occurrence or time to occurrence and does not raise the troublesome issues regarding the unblinding of group differences that other sequential methods create. The main advantage of the total endpoint stopping rule is that it provides set power. Standard fixed sample size designs provide a given power only on average. The power actually achieved in a particular fixed sample size trial is largely determined by the overall observed rate of endpoint occurrences. This claim about the total endpoint stopping rule is well established in the statistical literature and, as well as outlining the mathematical details in an Appendix, we use computer simulation of the PCDT to demonstrate that use of the stopping rule will allow termination of the trial while maintaining power and type I error at a predetermined level.

%Z Journal of clinical epidemiology

Journal article

%U https://cochrane.66557.net/central/doi/10.1002/central/CN-00197147/full

%0 Journal Article

%A EUCTR DK

%D 2018

%T Antibiotic treatment (Rifaxamin) of a chronic inflammatory diarrhoeal disease (Collagenous Colitis)

%8 2018-01-01

%X INTERVENTION: Trade Name: Xifaxan Product Name: Xifaxan Pharmaceutical Form: Coated tablet Pharmaceutical form of the placebo: Coated tablet Route of administration of the placebo: Oral use CONDITION: Collagenous Colitis Therapeutic area: Diseases [C] ‐ Digestive System Diseases [C06] PRIMARY OUTCOME: Main Objective: The aim of this study is to assess if 4 weeks treatment with Rifaximin as a supplement to a standard course of Budesonide against active Collagenous Colitis can reduce the risk of relapse after treatment cessation. Primary end point(s): The number of patients in clinical remission 12 weeks after cessation of Budesonide in the Rifaximin group compared to the placebo group Secondary Objective: The number of patients in clinical remission 12 weeks after cessation of Budesonide in the Rifaximin group compared to the placebo group. Remission defined as < 3 daily bowel movements or < 1 watery stool per day measured as a mean during the last week.; Reduction in MCDAI score from baseline to week 18. Time to relapse. ; Quality of life assessed by short health scale (SHS) 12 weeks after completed Budesonide‐course.; Number of patients in histolocigal remission 12 weeks after cessation of treatment. ; Changes is gut microbiome measured by 16SrDNA‐analysis on stool samples.; Proportion of patients in clinical remission and difference in quality of life 6 and 12 month after treatment cessation.; ; Timepoint(s) of evaluation of this end point: 12 weeks SECONDARY OUTCOME: Secondary end point(s): Reduction in MCDAI score from baseline to week 18. Time to relapse. ; Quality of life assessed by short health scale (SHS) 12 weeks after completed Budesonide‐course.; Number of patients in histolocigal remission 12 weeks after cessation of treatment. ; Changes is gut microbiome measured by 16SrDNA‐analysis on stool samples.; Proportion of patients in clinical remission and difference in quality of life 6 and 12 month after treatment cessation.; Timepoint(s) of evaluation of this end point: 12 weeks INCLUSION CRITERIA: • Age >18 years • Written informed consent • Histological findings fulfill criteria for CC: o A thickenened subepithelial collagenous band >10 µm in veloriented colonic biopsies and o Increased count of inflammatory cells in lamina propria • Diagnostic biopsies are a maximum of two years old • A history of nonbloody, watery diarrhea for more than two weeks prior to screening in patients with recently diagnosed CC or a history of clinical relapse for more than a week in patients with known CC • Active disease: > 3 stools/day or >1 watery stool/day measured as a mean during a week prior to baseline Are the trial subjects under 18? no Number of subjects for this age range: F.1.2 Adults (18‐64 years) yes F.1.2.1 Number of subjects for this age range 14 F.1.3 Elderly (>=65 years) no F.1.3.1 Number of subjects for this age range 34

%Z https://trialsearch.who.int/Trial2.aspx?TrialID=EUCTR2018-001891-39-DK

Trial registry record

%U https://cochrane.66557.net/central/doi/10.1002/central/CN-01907060/full

%0 Journal Article

%A NCT

%D 2017

%T Addressing Depression and Anxiety Symptoms in Patients With Inflammatory Bowel Disease

%8 2017-01-01

%K Anxiety Disorders; Colitis, Ulcerative; Crohn Disease; Depression; Depressive Disorder; Inflammatory Bowel Diseases; Intestinal Diseases

%X Inflammatory Bowel Diseases (IBD) are chronic debilitating disorders of the gastrointestinal tract that comprise two subtypes; Crohn's Disease (CD) and Ulcerative Colitis (UC). Canada has among the highest incidence rates of CD and UC in the world, as high as 20.2 and 19.5 per 100,000 respectively. Although, IBD can occur at any age, it is frequently diagnosed in the second and third decades of life, at a time when vulnerable individuals are entering the prime years of their lives. This age of onset, coupled with the recurrent and frequently relapsing nature of these disorders, can significantly impair the psychological well‐being of patients. Therefore, it's not surprising that patients with IBD report a higher burden of depression and anxiety in comparison to the general population. The prevalence of depression and anxiety in patients with IBD have previously been linked to the following: (1) Increased risk of surgery; (2) Increased number of relapses; (3) Clinical recurrence; (4) Treatment failure and earlier retreatment; (5) Lower self‐reported quality of life, satisfaction, and medication adherence; (6) and Increased health care utilization. Although, depression and anxiety are highly treatable conditions, they are often under‐recognized and under‐ treated in patients with IBD. The most common treatments for these disorders are pharmacological agents and psychological treatments. Psychological treatments like Cognitive Behavioral Therapy (CBT) have extensive support for treatment of depression and anxiety. The major advantage of psychological treatments over pharmacological agents is their ability to sustain improved depression and anxiety symptoms in patients post‐treatment. As part of this study, we aim to evaluate the following: Specific Aim #1: Determine whether a psychological intervention, involving web‐based CBT, is effective in ameliorating depression and anxiety symptoms in a cohort of adult IBD patients. Specific Aim #2: Determine the durability effect of the intervention on sustaining improved psychiatric symptoms. Specific Aim #3: Determine the impact of a psychological on IBD‐specific and psychiatric‐specific health care utilization.

%Z https://clinicaltrials.gov/show/NCT03327038

Trial registry record

%U https://cochrane.66557.net/central/doi/10.1002/central/CN-01577566/full

%0 Journal Article

%A Mañosa, M.

%A Cabré, E.

%A Bernal, I.

%A Esteve, M.

%A Garcia-Planella, E.

%A Ricart, E.

%A Peñalva, M.

%A Cortes, X.

%A Boix, J.

%A Piñol, M.

%A Et, Al.

%D 2013

%T Addition of metronidazole to azathioprine for the prevention of postoperative recurrence of Crohn's disease: a randomized, double-blind, placebo-controlled trial

%V 19

%N 9

%P 1889‐1895

%8 2013-01-01

%R 10.1097/MIB.0b013e31828ef13f

%K *Crohn disease/co [Complication]; *Crohn disease/dt [Drug Therapy]; *Crohn disease/pc [Prevention]; *Crohn disease/su [Surgery]; *azathioprine/ae [Adverse Drug Reaction]; *azathioprine/cb [Drug Combination]; *azathioprine/cm [Drug Comparison]; *azathioprine/ct [Clinical Trial]; *azathioprine/dt [Drug Therapy]; *metronidazole/ae [Adverse Drug Reaction]; *metronidazole/cb [Drug Combination]; *metronidazole/cm [Drug Comparison]; *metronidazole/ct [Clinical Trial]; *metronidazole/dt [Drug Therapy]; *recurrent disease/dt [Drug Therapy]; *recurrent disease/pc [Prevention]; Acute pancreatitis/si [Side Effect]; Adolescent; Adult; Anti‐Infective Agents [therapeutic use]; Article; Azathioprine [*therapeutic use]; Controlled study; Crohn Disease [complications, *surgery]; Double blind procedure; Double‐Blind Method; Drug Therapy, Combination; Drug efficacy; Drug safety; Dysgeusia/si [Side Effect]; Endoscopy; Fatigue/si [Side Effect]; Female; Follow‐Up Studies; Gastrointestinal symptom/si [Side Effect]; Headache/si [Side Effect]; Human; Humans; Hypertransaminasemia/si [Side Effect]; Immunosuppressive Agents [therapeutic use]; Intestine resection; Lymphocytopenia/si [Side Effect]; Major clinical study; Male; Metronidazole [*therapeutic use]; Molluscum contagiosum/si [Side Effect]; Multicenter study; Paresthesia/si [Side Effect]; Patient compliance; Pilot Projects; Placebo; Postoperative Complications [*prevention & control]; Priority journal; Prognosis; Randomized controlled trial; Risk reduction; Secondary Prevention; Surgical technique; Young Adult

%X BACKGROUND: Endoscopic recurrence occurs in up to 80% of patients with Crohn's disease 1 year after intestinal resection. Imidazole antibiotics, thiopurines, and particularly their combination have proven efficacy in preventing endoscopic recurrence. The aim of the study was to compare the efficacy of the addition of metronidazole (for 3 months after the surgical treatment) to azathioprine for the prevention of postsurgical endoscopic recurrence. METHODS: A pilot study was made of 50 patients with Crohn's disease undergoing intestinal resection with ileocolic anastomosis and treated with 2 to 2.5 mg/kg of azathioprine per day for 1 year. The patients were randomized to receive additional 15 to 20 mg/kg of metronidazole per day or placebo for the first 3 months (n = 25 per arm). Endoscopic assessment was performed 6 and 12 months after the surgical resection. The primary end point was the prevention of endoscopic recurrence as defined by a Rutgeerts score of <2 at 6 months. The initial sample size had an 80% statistical power in detecting an absolute risk reduction of ≥30%. RESULTS: Endoscopic recurrence occurred in 28% and 44% of the patients at 6 months (P = 0.19) and in 36% and 56% (P = 0.15) at 12 months in the metronidazole and placebo groups, respectively. No statistically significant differences were found between the treatment groups regarding severe endoscopic recurrence (Rutgeerts score ≥ 3) at 6 and 12 months. Likewise, there were no differences in the rate of adverse events between the treatment groups. CONCLUSIONS: The addition of metronidazole to azathioprine did not significantly reduce the risk of endoscopic recurrence beyond azathioprine alone in this study but does not worsen its safety profile.

%Z Inflammatory bowel diseases

Journal article

%U https://cochrane.66557.net/central/doi/10.1002/central/CN-00916036/full

%0 Journal Article

%A Cañete, F.

%A Mañosa, M.

%A Casanova, M. J.

%A González-Sueyro, R. C.

%A Barrio, J.

%A Bermejo, F.

%A Nos, P.

%A Iglesias-Flores, E.

%A García-Planella, E.

%A Pérez-Calle, J. L.

%A Et, Al.

%D 2019

%T Adalimumab or Infliximab for the Prevention of Early Postoperative Recurrence of Crohn Disease: results From the ENEIDA Registry

%V 25

%N 11

%P 1862‐1870

%8 2019-01-01

%R 10.1093/ibd/izz084

%K *Crohn disease; *relapse; Adalimumab [*therapeutic use]; Adult; Article; Clinical assessment; Clinical practice; Colonoscopy; Crohn Disease [*drug therapy, *prevention & control, surgery]; Drug efficacy; Drug therapy; Female; Human; Humans; Immunosuppressive Agents [therapeutic use]; Infliximab [*therapeutic use]; Intestinal Mucosa [pathology]; Logistic Models; Major clinical study; Male; Middle Aged; Multivariate Analysis; Prevention; Randomized controlled trial; Recurrence; Registries; Retrospective Studies; Risk assessment; Risk factor; Secondary Prevention; Spain; Surgery; Tumor Necrosis Factor‐alpha [antagonists & inhibitors]

%X BACKGROUND: Anti‐tumor necrosis factor agents (anti‐TNFs) are efficacious at preventing the postoperative recurrence (POR) of Crohn disease, as demonstrated in 2 randomized controlled trials. However, real‐life data for infliximab or adalimumab in this setting are scarce. Our aim was to assess both the efficiency of anti‐TNFs at preventing early POR of Crohn disease in clinical practice and the associated risk factors for POR. METHODS: Patients in whom anti‐TNFs were prescribed for the prevention of POR within 3 months after ileocolonic resection and who had an endoscopic assessment within 18 months were identified from the ENEIDA registry. Clinical and endoscopic features were collected within 18 months after surgery. RESULTS: In total, 152 patients were included (55 treated with infliximab, 97 with adalimumab, and 39% with concomitant immunosuppressants). Anti‐TNF treatment was started after a median time of 29 days (IQR 13‐44) after surgery. Eighty‐two percent of patients had at least one risk factor for POR, and 82% had been exposed to anti‐TNFs before the index surgery. Overall, 34% had endoscopic POR (as defined using a Rutgeerts endoscopic score > i1); 14% had advanced endoscopic POR (>i2); and 20% had clinical POR, with no differences between infliximab and adalimumab. In the multivariate analysis, only perianal disease (odds ratio 2.73, 95% confidence interval [CI] 1.26‐5.91) and rectal involvement (odds ratio 2.79, 95% CI 1.09‐7.14) were independent predictors of endoscopic POR. CONCLUSIONS: In clinical practice, anti‐TNFs for the prevention of POR of Crohn disease are frequently used in patients experienced with anti‐TNFs and with concomitant immunosuppressants. The efficacy of infliximab and adalimumab for POR prevention is similar and in accordance with the results obtained in randomized controlled trials.

%Z Inflammatory bowel diseases

Journal article

%U https://cochrane.66557.net/central/doi/10.1002/central/CN-01937259/full

%0 Journal Article

%A Papamichael, K.

%A Archavlis, E.

%A Lariou, C.

%A Mantzaris, G. J.

%D 2012

%T Adalimumab for the prevention and/or treatment of post-operative recurrence of Crohn's disease: a prospective, two-year, single center, pilot study

%V 6

%N 9

%P 924‐931

%8 2012-01-01

%R 10.1016/j.crohns.2012.02.012

%K Adalimumab; Adolescent; Adult; Antibodies, Monoclonal [therapeutic use]; Antibodies, Monoclonal, Humanized [administration & dosage, *therapeutic use]; Anti‐Inflammatory Agents, Non‐Steroidal [administration & dosage, *therapeutic use]; Azathioprine [therapeutic use]; Crohn Disease [*drug therapy, *prevention & control, surgery]; Endoscopy, Gastrointestinal; Female; Humans; Immunosuppressive Agents [therapeutic use]; Infliximab; Intestinal Mucosa [pathology]; Male; Mesalamine [therapeutic use]; Middle Aged; Pilot Projects; Postoperative Care; Secondary Prevention; Statistics, Nonparametric; Wound Healing; Young Adult

%X BACKGROUND: Infliximab has shown efficacy at preventing post operative recurrence (POR) of Crohn's disease (CD). This study aimed at evaluating whether adalimumab can prevent and treat POR of CD. METHODS: This prospective, single‐center, open‐label, two‐year study included 23 patients who had undergone ileocecal resection for refractory or complicated CD and were at high‐risk for POR. Patients received adalimumab from post operative day 14 (Group I, n=8) or at 6 months post operatively after confirmation of endoscopic recurrence (PO‐ER) despite treatment with azathioprine, infliximab, or 5‐ASA (patients intolerant to infliximab and azathioprine, Group II, n=15). Symptom assessment and laboratory tests were performed at monthly visits. Endoscopic findings were graded using the Rutgeerts score (RS) at 6 and 24 months after initiation of adalimumab. Primary end‐points were maintenance (group I) or achievement of mucosal healing (Group II). Secondary end‐points were prevention of post operative clinical recurrence (PO‐CR) (Group I) and endoscopic and clinical improvement (group II). RESULTS: In Group I, PO‐ER (RS≥i2) was seen in one patient at 6 months PO, whereas a second patient developed PO‐ER and PO‐CR after 24 months of treatment. In Group II, all patients had PO‐ER whereas 9 (60%) patients had PO‐CR at study enrolment; after 24 months of treatment 9/15 (60%) patients achieved complete (RS‐i0, n=3) or near complete (RS‐i1, n=6) mucosal healing and 5/9 (56%) clinical remission. No serious adverse events were reported. CONCLUSIONS: This pilot study suggests that adalimumab may prevent PO‐ER and treat PO‐ER/CR in high risk patients for POR of CD.

%Z Journal of Crohn's & colitis

Journal article

%U https://cochrane.66557.net/central/doi/10.1002/central/CN-00854269/full

%0 Journal Article

%A NCT

%D 2020

%T Adalimumab Dose Reduction Aiming Low Serum Concentration With Control of Disease Activity

%8 2020-01-01

%K Adalimumab; Arthritis; Arthritis, Rheumatoid

%X Biological agents are frequently prescribed to optimize rheumatoid arthritis care. In order to prevent joint destruction it is necessary to maintain remission or low disease activity. Up to now clinicians used to continue the initial treatment regimen to maintain remission or low disease activity. Since biologic therapy is expensive, and is associated with patient burden as dose dependant risk for serious infections, multiple studies have been performed to show that a large proportion of patients with rheumatoid arthritis with stable low disease activity can reduce their dose without relapse of disease. In addition the latest European and American recommendations pose to reduce the dosage or to discontinue the bDMARDs in case of persistent remission or low disease activity. Yet, there are no recommendations on how this should be carried out. Currently, most clinicians use Disease Activity Score in 28 joints (DAS28) and the Clinical Disease Activity Index (CDAI) to monitor dose reduction strategies. Although disease activity guided dose reduction is safe and cost‐effective, a relatively novel strategy is dose reduction using serum drug concentrations (therapeutic drug monitoring). Most biologics are characterized by wide variation in pharmacokinetics between patients, resulting in wide range of drug concentrations when administered at the labeled dose. Therapeutic drug monitoring can be a valuable tool for optimizing the dosage of biopharmaceuticals and improving patient care on individual level. In other autoimmune diseases, such as inflammatory bowel disease, it is thought to be superior to empirical dose reduction and is already applied in clinical practice. The rationale behind therapeutic drug monitoring is that medication dose correlates with serum drug levels and drug concentration correlates with therapeutic effect. The latter notion is demonstrated for adalimumb by Pouw et al. Adalimumab serum concentration in a range 5‐8 mg/L is sufficient for adequate response. In the first phase of treatment, drug concentration must be high enough to control immunogenicity. To control disease activity in the 2nd phase (after 28 weeks), lower concentrations than 5 mg/L are probably sufficient. Our study group illustrated in 2018 that reducing adalimumab dose by prolonging the dosing interval with 50%, is non‐inferior to continuation in patients with adalimumab levels > 8mg/L. In addition, recent published data suggest that concentrations of 0.1‐0.5 mg/L are enough to control TNF in this phase. Since around 70% of the patients have adalimumab concentration above 5 mg/l, while an adalimumab concentration of 5 mg/L is enough for adequate response (7), a large extent of patients might thus be overexposed. The hypothesis is that 1/ tapering adalimumab in RA patient doing well after 28 weeks using TDM aiming at 5mg/l (and disease activity measurement) results in maintenance of disease control and lower adalimumab use, and that 2/ tapering to a lower target drug level of 2 mg/L is non inferior to the higher 5mg/l target with respect to disease activity control and safety, and superior in adalimumab reduction. Tapering to achieve these lower targets (for example direct doubling of interval in patients with levels > 10 mg/L) might result in the lowest effective drug dose. Data regarding disease status, functioning, adalimumab serum concentrations, anti‐drug antibodies and medical costs will be collected during this study. Disease activity after dose reduction, aiming adalimumab concentration of 2 mg/L or 5 mg/L, in rheumatoid arthritis patients treated with adalimumab for at least 28 weeks and a serum adalimumab concentration above 5 mg/L, will be evaluated in this multi‐centre, randomized, single blinded trail. Patients with an adalimumab concentration above 5mg/L will be randomly assigned to dose reduction by extending their dosing interval aiming a drug level of 2 mg/L or aiming a drug level of 5 mg/L. A newly developed algorithm is used to determine the interval prolongation for each patient

%Z https://clinicaltrials.gov/show/NCT04222920

Trial registry record

%U https://cochrane.66557.net/central/doi/10.1002/central/CN-02079211/full

%0 Journal Article

%A Bao, C.

%A Wu, L.

%A Wang, D.

%A Chen, L.

%A Jin, X.

%A Shi, Y.

%A Li, G.

%A Zhang, J.

%A Zeng, X.

%A Chen, J.

%A Et, Al.

%D 2022

%T Acupuncture improves the symptoms, intestinal microbiota, and inflammation of patients with mild to moderate Crohn's disease: a randomized controlled trial

%V 45

%P 101300

%8 2022-01-01

%R 10.1016/j.eclinm.2022.101300

%K *Crohn disease; *acupuncture; *alternative medicine; *inflammation; *inflammatory bowel disease; *intestine flora; Adult; Article; Basic research; China; Clinical trial; Controlled study; Crohn Disease Activity Index; Crohn disease [therapy]; Faecalibacterium prausnitzii; Female; Follow up; Gene expression; Han Chinese; Histopathology; Human; Human tissue; Major clinical study; Male; Moxibustion; Nonhuman; Operational taxonomic unit; Outcome assessment; Outpatient department; Parallel design; Randomized controlled trial; Recurrence risk; Remission; Roseburia; Roseburia faecis; Th1 cell; Th17 cell; acupuncture; adolescent; adult; aged; article; colonoscopy; controlled study; female; follow up; hemostasis; histopathology; human; human tissue; inflammation; intestine flora; major clinical study; male; moxibustion; nonhuman; operational taxonomic unit; outpatient department; parallel design; population abundance; protein blood level; randomized controlled trial; recurrence risk; remission; sham procedure; symptom; tertiary care center

%X Background: The efficacy and mechanisms of acupuncture for Crohn's disease (CD) are not well understood. We investigated its effects on symptoms, intestinal microbiota, and circulating inflammatory markers in CD patients. Methods: This 48‐week, randomized, sham controlled, parallel‐group clinical trial was performed at a tertiary outpatient clinic in China. From April 2015 to November 2019, 66 patients (mean age 40·4, 62·1% were male, all were Han Chinese) with mild to moderate active CD and unresponsive to drug treatment were enrolled and randomly assigned equally to an acupuncture group or a sham group. The treatment group received 3 sessions of acupuncture plus moxibustion per week for 12 weeks and a follow‐up of 36 weeks. Clinicaltrials.gov: NCT02559037. Findings: At week 12, the clinical remission rate (the primary outcome) and clinical response rate of acupuncture group were significantly higher than that of sham group, with a difference of 42·4% (95% CI: 20·1%‐64·0%) and 45·5% (95% CI: 24·0%‐66·9%), respectively, both of which maintained at week 48. The acupuncture group had significantly lower CD activity index and C‐reactive protein level at week 12, which maintained at 36‐week follow‐up. The CD endoscopic index of severity, histopathological score, and recurrence rate at week 48 were significantly lower in acupuncture group. The number of operational taxonomic unit of intestinal microbiota and relative abundance of Faecalibacterium prausnitzii and Roseburia faecis were increased. Plasma diamine oxidase, lipopolysaccharide, and Th1/Th17 related cytokines were decreased in 12‐week after acupuncture. Interpretation: Acupuncture was effective in inducing and maintaining remission in patients with active CD, which was associated with increased abundance of intestinal anti‐inflammatory bacteria, enhanced intestinal barrier, and regulation of circulating Th1/Th17‐related cytokines. Funding: National Key Basic Research Program of China (2015CB554500 and 2009CB522900), Shanghai Rising‐Star Program (19QA1408100).

%Z EClinicalMedicine

Journal article

%U https://cochrane.66557.net/central/doi/10.1002/central/CN-02372747/full

%0 Journal Article

%D 2023

%T Abstracts of the 18th Congress of ECCO

%V 17

%8 2023-01-01

%K Adult; Bone marrow cell; Clinical trial; Cohort analysis; Colectomy; Complication; Conference review; Controlled study; Crohn disease; DNA methylation; Double blind procedure; Drug efficacy; Drug therapy; Epigenome; Epithelium; Female; Ferroptosis; Gene network analysis; Gene sequence; Genetic susceptibility; Human; Human cell; Hungarian (citizen); Ileitis; Inflammatory bowel disease; Kidney; Male; Phase 3 clinical trial; Post hoc analysis; Randomized controlled trial; Risk factor; Single cell analysis; Surgery; Ulcerative colitis

%X The proceedings contain 1084 papers. The topics discussed include: Sequencing‐based gene network analysis reveals a profound role for ferroptosis key gene GPX4 in post‐operative endoscopic recurrence in Crohn's disease; distinct biological profiles associated with the risk of short‐term relapse and mid/long‐term relapse in Crohn's disease patients stopping infliximab; highly stable epigenome‐wide peripheral blood DNA methylation signatures accurately predict endoscopic response to adalimumab, vedolizumab and Ustekinumab in Crohn's disease patients: The epic‐cd study; efficacy of Upadacitinib in ulcerative colitis: A phase 3 post hoc analysis of biologic‐ and anti‐TNF‐inadequate response patients; high‐dimensional single‐cell analysis identifies cellular signatures associated with response to vedolizumab therapy in ulcerative colitis; multiomic interrogation of the epithelium in active ulcerative colitis reveals dysregulated myeloid cells associated with non‐response to anti‐tumor necrosis factor therapy; absolute and relative risks of kidney and urological complications in patients with inflammatory bowel disease; mucosal healing with vedolizumab in inflammatory bowel disease patients with chronic pouchitis: Evidence from earnest, a randomized, double‐blind, placebo‐controlled trial; and long‐term colectomy rates of ulcerative colitis over 40‐year of different therapeutic eras ‐ results from a western Hungarian population‐based inception cohort between 1977‐2020.

%Z Journal of Crohn's & colitis

Journal article; Conference proceeding

%U https://cochrane.66557.net/central/doi/10.1002/central/CN-02526728/full

%0 Journal Article

%A NTR

%D 2017

%T A trial to compare if the diseased area beween the colon and the small bowel of patients with Crohn's disease stays free of disease after surgery between a group of patients whom receive no medication versus a group whom receive vedolizumab

%8 2017-01-01

%X INTERVENTION: Vedolizumab 300 milligram 8 weekly (4 doses) or placebo 8 wekkly (4 doses) CONDITION: Crohn's disease ; ; PRIMARY OUTCOME: The proportion of patients with clinically significant endoscopic recurrence (Rutgeerts i2b, i3 or i4) at week 26. SECONDARY OUTCOME: ‐ Proportion of patients without endoscopic recurrence (i0) ; ; ‐ Symptomatic recurrence (CDAI increase >70 points compared to baseline) ; ; ‐ Proportion of patients with normalized serum CRP at all time points and CRP at all visits ; ; ‐ Proportion of patients with normal fecal calprotectin (<50) at all visits ; ; ‐ Quality of life measured by IBDQ and SF‐36 ; ; ‐ Serum concentrations of vedolizumab and antibodies to vedolizumab before every infusion ; INCLUSION CRITERIA: 1. In the opinion of the investigator, the subject is capable of understanding and complying with protocol requirements. 2. The subject signs and dates a written, informed consent form and any required privacy authorization prior to the initiation of any study procedures. 3. Established Crohn’s disease as the indication for ileocolonic resection 4. Age > 18 5. Ileocolonic resection with ileocolonic anastomosis and removal of all tissue macroscopically affected by CD according to the surgeon 6. Presence of at least 1 risk factor for recurrence: • Active smoking > 10 cigarettes/day • 2nd, 3rd or later resection • Surgery for perforating complication (abscess, fistula) • Previous exposure to anti‐TNF antibodies 7. Male or non‐pregnant, non‐lactating females. Females of child bearing potential must have a negative serum pregnancy test prior to randomization, and must use a hor

%Z https://trialsearch.who.int/Trial2.aspx?TrialID=NTR6385

Trial registry record

%U https://cochrane.66557.net/central/doi/10.1002/central/CN-01885413/full

%0 Journal Article

%A NCT

%D 2007

%T A Study of Infliximab for Treatment Resistant Major Depression

%8 2007-01-01

%K Depression; Depressive Disorder; Depressive Disorder, Major; Infliximab

%X Major depression has become a health crisis of epidemic proportions in the modern world. The prevalence of major depression has risen over the last several generations in every country examined, and age of symptom onset has decreased. Currently the fourth leading health burden worldwide, major depression will rank second after cardiac disease as a cause of international medical morbidity by the year 2020. One in six individuals in the United States will experience an episode of major depression in his or her lifetime, and the risk of subsequent episodes rises dramatically once a person has been depressed. Indeed, depression is now recognized to be a highly chronic and recurrent illness. On average, patients with major depression are symptomatic 60% of the time, even when receiving community‐standard antidepressant treatment. Recent estimates place the economic burden of depression in the United States at 83 billion dollars a year. Depression is associated with greater disability than are most other chronic illnesses and is a risk factor for mortality. Suicide ranks among the top ten causes of death in the United States, and best estimates suggest that 60‐70% of people who kill themselves are clinically depressed. Between 10‐15% of severely depressed people eventually commit suicide. In addition, many studies indicate that depression significantly increases all‐cause mortality independently of suicide. Depression predicts the later development of a number of medical conditions, including cardiac and cerebrovascular disease, hypertension,diabetes,obesity and the metabolic syndrome,dementia, and cancer. Depression also markedly increases mortality in patients who are medically ill and has been associated with decreased responses to pharmacological treatments for cancer and hepatitis C. Unfortunately, most patients with depression do not experience a complete resolution of symptoms with antidepressant treatment and 10‐20% of patients are refractory to all currently available modalities, including electroconvulsive shock (ECT) therapy. ECT is often effective in patients who have failed adequate trials of multiple antidepressants, but is associated with the risk of anesthesia and with significant short term memory impairment. Responses to ECT are short‐lived, and many patients who respond subsequently relapse, even when on maintenance antidepressants. In addition to efficacy issues, many patients are unable to tolerate side effects associated with antidepressants or ECT. The risks of not responding to (or tolerating) treatment have been highlighted by recent studies documenting that partial—but incomplete—response is associated with an increased risk of full symptomatic relapse (even when on therapy) and a worse long term disease course, as well as with significantly impaired quality of life. Treatment resistance also results in a six times increase in direct health care costs. These factors highlight the tremendous need to identify novel treatment strategies, especially for depressed patients who are unresponsive to conventional therapies. One possible mechanism that may contribute to treatment resistance is increased proinflammatory cytokine production and release. Several lines of evidence indicate that proinflammatory cytokines participate in the pathophysiology of major depression and may thus represent a novel target for the pharmacological treatment of the disorder. First, a high percentage of patients who receive cytokine therapies (such as interferon‐alpha for malignant melanoma or hepatitis C infection) develop depressive symptoms, and many patients meet full criteria for major depression. Interferon‐alpha‐induced depressive symptoms can be ameliorated by pre‐treatment with an antidepressant and respond to antidepressants once they have emerged. Second, many studies report that, as a group, medically healthy patients with depression exhibit elevated measures of proinflammatory cytokines, including tumor necrosis factor (TNF)‐alpha, interleukin (IL)‐1 and IL‐6. Moreover, a positive relationship between serum concentrations of proinflammatory cytokines and severity of depressive symptoms has been recently reported. Third, antidepressants have been shown to have anti‐inflammatory activity and may work—at least in part—by reducing inflammatory activity, given evidence that clinical response is associated with reductions in cytokine levels. These data raise the possibility that cytokine antagonists, such as the chimeric anti‐TNF‐alpha antibody infliximab, might have antidepressant efficacy. Of special relevance to this proposal, patients who are treatment resistant have been shown to exhibit increased inflammatory activity (as reflected by increased plasma concentrations of interleukin [IL]‐6 and the soluble IL‐6 receptor [sIL‐6R]), suggesting that cytokine antagonists might be especially effective in these patients. Providing care to patients with inflammatory bowel disease has given us the clear clinical impression that infliximab rapidly improves mood and energy levels in many patients prior to any demonstrable changes in bowel pathology. This impression is in line with a growing body of evidence suggesting that TNF‐alpha antagonists improve emotional functioning and fatigue in patients receiving these agents for rheumatoid arthritis and inflammatory bowel disease. These findings in patients with inflammatory diseases are consistent with the notion that TNF‐alpha antagonists such as infliximab might provide acute symptomatic relief for medically healthy patients with treatment‐resistant major depression and that symptom improvement might result from decreased inflammatory activity. Moreover, medically healthy depressed patients with increased inflammatory activity may be most likely to benefit from anti‐TNF‐alpha therapy.

%Z https://clinicaltrials.gov/show/NCT00463580

Trial registry record

%U https://cochrane.66557.net/central/doi/10.1002/central/CN-02042717/full

%0 Journal Article

%A De Vos, M.

%A Maertens, V.

%A Maes, L.

%A Fierens, K.

%A Van Kerschaver, O.

%A Kint, M.

%A Van Outryve, L.

%A Onghena, T.

%D 2018

%T A single-centre analysis on Hemorrhoidal Artery Ligation (HAL) and Recto-Anal Repair (RAR) after ten years

%V 24

%N 2

%P 28

%8 2018-01-01

%K *artery ligation; *rectal artery; Adult; Aged; Anus disease; Anus sphincter; Belgium; Bleeding; Chi square test; Colon motility; Colorectal tumor; Conference abstract; Controlled study; Data analysis software; Female; General hospital; Human; Inflammatory bowel disease; Major clinical study; Male; Medical record; Neurologic disease; Outpatient department; Patient satisfaction; Portal hypertension; Postoperative pain; Pregnancy; Prolapse; Prospective study; Questionnaire; Randomized controlled trial; Recurrence risk; Suture; Thrombosis; Urine retention; Visual analog scale; Wilcoxon signed ranks test

%X Introduction: Hemorrhoidal disease is a typical “civilization” disease with a multifactorial pathogenesis and a symptomatology that poorly correlates to clinical findings. Treatment options are abundant and rangebetween conservative measures and surgical hemorrhoidectomy. Hemorrhoidal Artery Ligation (HAL) with orwithout Recto‐Anal Repair (RAR) is a minimally invasive surgical technique for all grades of hemorrhoidal disease. Materials and Methods: This study analyzed the outcomes of 274 consecutive HAL procedures with or without RAR between January 2004 and August 2014, at Sint‐Lucas General Hospital in Ghent, Belgium. Initially 365 patients were selected. Because of co‐existent anal pathologies, Inflammatory Bowel Disease, a history of colorectal tumors, portal hypertension, neurologic disease that affect the colonic motility and/or anal sphincter and pregnancy, 91 were excluded. The patient population consisted of 159 males and 115 females between 18 and 75 years of age (mean 51 yrs) with grade I ‐IV hemorrhoidal disease. Data was collected through a questionnaire and the patient records. All data was analyzed with SPSS 24.0 using the Wilcoxon matched pairs signed ranks test (p<0.05), the Mann‐Whitney U test (p<0.05) and the Chi‐square test (p<0.05). Results: The initial, predominant symptomatology was discomfort in daily life, anal blood loss, anal pain andhemorrhoidal prolapse. Hemorrhoidal thrombosis occurred in 61.1%. Before seeking medical treatment, 61.1%were symptomatic for years and 73.8% were treated before the HAL procedure was performed. Patients were treated by HAL or combined HAL and RAR. During a HAL procedure, a mean of 5 sutures were placed (range 2‐8). For RAR, a mean of 2 mucopexies were performed (range 1‐3). 74.8% was treated on a day‐clinic basis.Mean Visual Analog Scale (VAS) for postoperative pain is 5.28 (HAL 4.17, HALRAR 6.06). The postoperative complication rate was 11.3%: anal pain (3.6%), hemorrhoidal thrombosis (3.3%) and urinary retention (2.6%). Symptoms of hemorrhoidal disease were all significantly less apparent after HAL or HALRAR. But 44.4% of patients needed further treatment of their hemorrhoidal disease. Satisfaction after HAL or HALRAR was 84.4%. Conclusion: These long‐term results after HALRAR show a significant decrease in symptomatology and a high patient satisfaction but confirmed concerns about high recurrence rates. More prospective randomized trials are needed to evaluate long term results compared to other surgical techniques.

%Z Ambulatory surgery

Journal article; Conference proceeding

%U https://cochrane.66557.net/central/doi/10.1002/central/CN-01731744/full

%0 Journal Article

%A Consigny, Y.

%A Modigliani, R.

%A Colombel, J. F.

%A Dupas, J. L.

%A Lémann, M.

%A Mary, J. Y.

%D 2006

%T A simple biological score for predicting low risk of short-term relapse in Crohn's disease

%V 12

%N 7

%P 551‐557

%8 2006-01-01

%R 10.1097/01.ibd.0000225334.60990.5b

%K Adult; Blood Sedimentation; Crohn Disease [*diagnosis, pathology, *therapy]; C‐Reactive Protein [metabolism]; Disease‐Free Survival; Double‐Blind Method; Erythrocytes [metabolism]; Female; Humans; Male; Proportional Hazards Models; Recurrence; Remission Induction; Risk; Sensitivity and Specificity; Time Factors

%X BACKGROUND: In Crohn's disease, studies have evaluated the ability of biological markers to predict relapse in the next 12 to 18 months, without differentiating early from late relapses. The aim of this study was to look for biological markers of short‐term relapse. MATERIALS AND METHODS: In a previous therapeutic trial, patients with a medically induced clinical remission had biological markers evaluated and updated every 6 weeks. A Cox model with time‐dependent covariates was used for analysis. RESULTS: Among the 71 patients, 38 had a relapse. Multivariate analysis selected 2 markers predictive of relapse: C‐reactive protein >20 mg/L and erythrocyte sedimentation rate >15 mm. A binary biological predictive score was derived: "negative" when both were lower than their limits, "positive" when otherwise. The relative risk of short‐term relapse for patients with a positive score compared to those with a negative score was 8.0 (95% confidence interval 2.8‐22.9). Sensitivity of the score was 89% and specificity was 43%. Assuming a 10% relapse rate every 6 weeks, negative and positive predictive values were 97% and 15%, respectively. CONCLUSIONS: This simple biological score can predict short‐term maintenance of remission in Crohn's disease and may help physicians in the follow‐up of patients in clinical remission.

%Z Inflammatory bowel diseases

Journal article

%U https://cochrane.66557.net/central/doi/10.1002/central/CN-00570939/full

%0 Journal Article

%A Radford-Smith, D.

%A Satsangi, J.

%A Louis, E.

%A Laharie, D.

%A Ding, N.

%A Siegmund, B.

%A D'Haens, G.

%A Erik, H.

%A Yates, A.

%A Anthony, D.

%A Et, Al.

%D 2022

%T A serum metabolite panel predicts Crohn's disease relapse in patients discontinuing infliximab and continuing antimetabolite therapy: sub-analysis of the SPARE Trial

%V 16

%P i458‐i459

%8 2022-01-01

%R 10.1093/ecco-jcc/jjab232.621

%K *Crohn disease; *recurrent disease; *relapse; *remission; Adult; Cohort analysis; Conference abstract; Controlled study; Cross validation; Diagnostic test accuracy study; Discriminant analysis; Drug combination; Drug therapy; Drug withdrawal; Feces; Female; Gene expression; Human; Human tissue; Major clinical study; Male; Metabolic fingerprinting; Metabolomics; Monotherapy; Nuclear magnetic resonance spectroscopy; Partial least squares regression; Predictive value; Protein blood level; Randomized controlled trial; Receiver operating characteristic; Validation process

%X Background: Combination therapy with infliximab and anti‐metabolites is an effective strategy for maintaining remission in patients with Crohn's disease (CD); however, the implications of long‐term combination therapy has led clinicians to consider discontinuing one of the two agents after sustained remission has been achieved. The SPARE investigators have addressed this in an unblinded clinical trial in which 211 patients were randomised to either continuation of combination therapy, withdrawal of infliximab, or withdrawal of antimetabolite therapy. Here, we aimed to determine whether nuclear magnetic resonance (NMR)‐based metabolomics analysis of patient sera at baseline could determine which patients randomised to infliximab withdrawal would relapse over 2‐years on anti‐metabolite monotherapy. Methods: The study cohort comprised patients randomised to infliximab cessation who had a serum aliquot available for analysis (n=63). All patients had been treated with a combination therapy of infliximab (IFX) and anti‐metabolite >8 months and had been in sustained steroid‐free remission >6 months. Disease relapse was defined by CDAI>250 (or between 150‐250 with 70 points increase over two consecutive weeks) alongside serum CRP and faecal calprotectin levels. Untargeted metabolomic profiling was carried out using NMR spectroscopy. Key metabolites were identified by their variable importance in projection (VIP) score using orthogonal partial least squares discriminant analysis (OPLS‐DA) with 10‐fold cross‐validation on independent data. Multiple logistic regression on the metabolites with highest VIP score was used to report a receiver operator characteristic (ROC) curve integral (AUC) value after collinear variables were removed. Results: Of 63 patients, 21 (33%) relapsed. The mean time to relapse after infliximab discontinuation was 7.9 months. Four independent (multicollinearity R2<0.4) serum metabolites (alanine, glutamine, acetoacetate, glucose) identified relapse with an AUC of 0.84 (95% confidence interval 0.74‐0.94, p<0.0001). The positive predictive value was 66.7%, and the negative predictive value was 77.1%. All serum metabolites identified were independent of baseline CRP level (p>0.05, Pearson correlation). Patients who relapsed following infliximab discontinuation had significantly elevated serum acetoacetate and decreased alanine and glutamine levels (post‐hoc students t‐test, p<0.05) at baseline. Conclusion: Here, we identify a panel of serum metabolites independent of baseline serum CRP, which predict high risk of disease relapse after infliximab discontinuation. These data may help in stratifying patients suitable for drug withdrawal, together with other clinical and multiomic parameters.

%Z Journal of Crohn's & colitis

Journal article; Conference proceeding

%U https://cochrane.66557.net/central/doi/10.1002/central/CN-02385351/full

%0 Journal Article

%A Lémann, M.

%A Mary, J. Y.

%A Colombel, J. F.

%A Duclos, B.

%A Soule, J. C.

%A Lerebours, E.

%A Modigliani, R.

%A Bouhnik, Y.

%D 2005

%T A randomized, double-blind, controlled withdrawal trial in Crohn's disease patients in long-term remission on azathioprine

%V 128

%N 7

%P 1812‐1818

%8 2005-01-01

%R 10.1053/j.gastro.2005.03.031

%K *Crohn disease/dt [Drug Therapy]; *azathioprine/ae [Adverse Drug Reaction]; *azathioprine/ct [Clinical Trial]; *azathioprine/dt [Drug Therapy]; Adult; Article; Azathioprine [*administration & dosage, adverse effects]; Biomarkers [blood]; Bone marrow disease/si [Side Effect]; C reactive protein; Chromosome aberration/si [Side Effect]; Clinical trial; Controlled clinical trial; Controlled study; Crohn Disease [*drug therapy, *pathology]; C‐Reactive Protein [analysis]; Double blind procedure; Double‐Blind Method; Drug withdrawal; Female; Hemoglobin; Human; Humans; Immunosuppressive Agents [*administration & dosage, adverse effects]; Kaplan Meier method; Karyotype; Leukopenia/si [Side Effect]; Long term care; Major clinical study; Male; Middle Aged; Multicenter study; Multivariate analysis; Myelodysplastic syndrome/si [Side Effect]; Placebo; Predictive Value of Tests; Priority journal; Protein blood level; Randomized controlled trial; Rash/si [Side Effect]; Recurrence; Remission; Steroid; Time Factors; Time series analysis; Treatment Outcome

%X BACKGROUND & AIMS: An open study reported that patients with Crohn's disease in remission who have taken azathioprine for longer than 3.5 years are at low risk of relapse when azathioprine is discontinued. To confirm this observation, we performed a multicenter, double‐blind, noninferiority withdrawal study. METHODS: Patients who were in clinical remission on azathioprine for > or = 42 months were randomized to continue azathioprine or to receive an equivalent placebo for 18 months. The primary end point was clinical relapse at 18 months. RESULTS: Forty patients were randomly assigned to receive azathioprine and 43 to receive placebo. Characteristics of patients at entry were similar in the 2 study groups. At 18 months, 3 patients had a relapse in the azathioprine group, and 9 had a relapse in the placebo group. Kaplan‐Meier estimates of the relapse rate at 18 months were 8% +/‐ 4% and 21% +/‐ 6%, respectively. The hypothesis that placebo was inferior to azathioprine was not rejected (P = .195). Among the baseline variables, C‐reactive protein level > 20 mg/L, time without steroids < 50 months, and hemoglobin level < 12 g/dL were found to be predictive of relapse in the multivariate analysis. CONCLUSIONS: This study shows that azathioprine withdrawal is not equivalent to continued therapy with azathioprine for maintenance of remission in patients with Crohn's disease who have been in remission on azathioprine for > or = 3.5 years. Thus, azathioprine maintenance therapy should be continued beyond 3.5 years.

%Z Gastroenterology

Journal article

%U https://cochrane.66557.net/central/doi/10.1002/central/CN-00511673/full

%0 Journal Article

%A Lindman, H.

%A Nygren, P.

%A Valachis, A.

%D 2023

%T A randomized double-blind placebo controlled phase 3 trial on the effect of Salovum™ and SPCFlakes™ on abemaciclib-induced gastrointestinal toxicity in early breast cancer - the ASF-BC study

%V 83

%N 5

%8 2023-01-01

%R 10.1158/1538-7445.SABCS22-OT3-30-01

%K *breast cancer; *gastrointestinal toxicity; Adult; Adverse drug reaction; Breast cancer recurrence; Cancer adjuvant therapy; Cancer patient; Cancer recurrence; Cancer survival; Carcinoid; Cholera; Clinical trial; Conference abstract; Controlled study; Crohn disease; Diarrhea; Disease free survival; Double blind procedure; Drug safety; Drug therapy; Egg powder; Electronic medical record; Female; Fruit juice; Hormonal therapy; Human; Inflammation; Ingestion; Major clinical study; Medical leave; Multicenter study; Nonhuman; Oat; Outcome assessment; Patient‐reported outcome; Phase 3 clinical trial; Prevention; Quality of life; Questionnaire; Randomized controlled trial; Side effect; Sweden; Systemic therapy; Treatment interruption; Ulcerative colitis

%X Background: In patients with high‐risk luminal breast cancer, the addition of CDK 4/6‐inhibitor abemaciclib to adjuvant endocrine therapy for two years has been associated with improved disease‐free survival and is now recommended as the preferred treatment strategy for this patient group. However, patients treated with abemaciclib frequently (82%) experience diarrhea which primarily occurs during the first three months from treatment initiation and seems to impact patients' quality of life. No proactive strategy to reduce the occurrence of abemaciclib‐induced diarrhea is proposed but patients are recommended to start with loperamide upon the occurrence of diarrhea to be combined with treatment interruption and dose adjustment as needed. Cholera induced diarrhea, as well as other forms of diarrhea‐inducing agents, has been shown to elicit a stimulated, endogenous production of a protein, named “antisecretory factor”, ASF, which acts by modulating secretion of water and ions but also counteracts inflammatory processes. ASF is commercialized as Salovum® and registered by the EU authorities as “Food for specific medical purposes”. Another way to increase ASF and, thus, to achieve benefit, is to induce its production/conversion by ingestion of malted oat flakes (SPC‐flakes®) which has been recommended or considered for several secretory pathological conditions. Salovum has been shown to rapidly, ie within hours to a few days, antagonize diarrheal diseases of various etiologies. It has also been used against high fluid passages and inflammation in Crohn disease, Colitis ulcerosa and carcinoids in adults. SPC flakes have similar effects but need weeks of administration to emerge. Importantly, to raise body ASF, by Salovum or SPC‐flakes, for the above indications has not been associated with adverse effects. Methods: This is a randomized double‐blind multicenter phase III study aiming to investigate a proactive strategy including Salovum and SPC‐flakes to prevent the occurrence of abemaciclibinduced diarrhea in patients with early breast cancer treated with abemaciclib. A total of 100 patients will be randomized, in a 1:1 manner, between 13 weeks with Salovum/SPC flakes (A) or placebo (B). The study will be conducted in up to ten different oncology departments in Sweden starting in Q3 2022. Primary objective: Occurrence of any‐grade (mild, moderate, severe) diarrhea according to the Systemic Treatment‐Induced Diarrhea Assessment Tool (STIDAT; patient‐reported outcome). Secondary objectives: Occurrence of any‐grade diarrhea according to CTCAE v. 5.0, health‐related quality of life (QoL), other adverse events related to abemaciclib, adherence to planned abemaciclib treatment, adherence to and pharmacodynamic effect from study products, safety of investigational products, sick leave duration, breast cancer recurrence. Investigational product, dosage and mode of administration: Salovum/placebo egg powder high in antisecretory factor, 4 g. Four sachets, ie 16 g q 8 h for 5 ‐ 7 days before start of abemaciclib. The appropriate amount of Salovum is mixed with 100 ‐ 200 ml of suitable liquid, e.g., fruit juice, and ingested orally. SPC‐flakes/placebo flat dose of 75 g/d divided in 2 ‐ 4 doses started in parallel with Salovum/placebo to be continued during the first 12 weeks of treatment with abemaciclib. End of study: All included patients will be followed using questionnaires up to 12 weeks from initiation of abemaciclib. After 12 weeks, all the patients will be followed through electronic medical records until the end of abemaciclib treatment (up to two years) for collecting potential adverse events and information on sick leave. Data from electronical medical records regarding recurrence and subsequent therapy will be collected until breast cancer recurrence or up to 5 years from initiation of abemaciclib.

%Z Cancer research

Journal article; Conference proceeding

%U https://cochrane.66557.net/central/doi/10.1002/central/CN-02537599/full

%0 Journal Article

%A Mantzaris, G. J.

%A Sfakianakis, M.

%A Archavlis, E.

%A Petraki, K.

%A Christidou, A.

%A Karagiannidis, A.

%A Triadaphyllou, G.

%D 2004

%T A prospective randomized observer-blind 2-year trial of azathioprine monotherapy versus azathioprine and olsalazine for the maintenance of remission of steroid-dependent ulcerative colitis

%V 99

%N 6

%P 1122‐1128

%8 2004-01-01

%R 10.1111/j.1572-0241.2004.11481.x

%K Adult; Aminosalicylic Acids [*administration & dosage]; Analysis of Variance; Azathioprine [*administration & dosage]; Colitis, Ulcerative [*diagnosis, *drug therapy]; Dose‐Response Relationship, Drug; Drug Administration Schedule; Drug Therapy, Combination; Female; Follow‐Up Studies; Humans; Male; Middle Aged; Prospective Studies; Remission Induction; Risk Assessment; Severity of Illness Index; Single‐Blind Method; Statistics, Nonparametric; Steroids [therapeutic use]; Treatment Outcome

%X OBJECTIVE: The aim of this prospective study was to assess whether the coadministration of azathioprine (AZA) and olsalazine is superior to AZA monotherapy in maintaining remission of steroid‐dependent ulcerative colitis (UC). METHODS: Patients with steroid‐dependent UC in remission were randomized to receive AZA alone (2.2 mg/kg) or in combination with olsalazine (0.5 g tid). Remission was defined as steroid withdrawal, an Ulcerative Colitis Clinical Activity Index (UCCAI) score of <2, an Ulcerative Colitis Disease Activity Index (UCDAI) score of 0, and a negative colonoscopy and histology. Patients were followed in the outpatient clinic every month for 2 yr. The study protocol included 1) monthly clinical examination, assessment of UCCAI, hematological and biochemical tests, and compliance with treatment; 2) a sigmoidoscopy and completion of inflammatory bowel disease quality‐of‐life questionnaire (IBD‐Q) and UCDAI every 3 months; and 3) total colonoscopy with biopsies at the end of the first and second year of the trial. RESULTS: Seventy patients were randomized to receive AZA alone (n = 34) or with olsalazine (n = 36). Three patients in each group developed side effects or could not comply with treatment and were withdrawn from the study. Three patients receiving AZA relapsed after the first year of the study and three after the second year of the study (19%). In the combination therapy group four patients relapsed after the first year of study and two after the second year of the study (18%). Relapse rates were not significant whether analyzed by intention‐to‐treat or per protocol. There were no significant differences between groups in time to relapse or discontinuation of treatment, UCCAI, UCDAI, or IBD‐Q scores. However, the number of adverse events and the cost of treatment were significantly higher, whereas compliance with treatment was poorer in the combination therapy. CONCLUSION: Patients with steroid‐dependent UC successfully maintained in remission on AZA are not in need of 5‐aminosalicylic acid compounds.

%Z American journal of gastroenterology

Journal article

%U https://cochrane.66557.net/central/doi/10.1002/central/CN-00482689/full

%0 Journal Article

%A EUCTR NL

%D 2010

%T A Multicenter Trial Comparing REMICADE (infliximab) and Placebo in the Prevention of Recurrence in Crohn's Disease Patients Undergoing Surgical Resection Who Are at an Increased Risk of Recurrence

%8 2010-01-01

%X INTERVENTION: Trade Name: Remicade Product Name: REMICADE Pharmaceutical Form: Powder for concentrate for solution for infusion INN or Proposed INN: INFLIXIMAB CAS Number: 170277‐31‐3 Current Sponsor code: INFLIXIMAB Other descriptive name: n/a Concentration unit: mg milligram(s) Concentration type: equal Concentration number: 100‐ Pharmaceutical form of the placebo: Powder for concentrate for solution for infusion Route of administration of the placebo: Intravenous use CONDITION: Crohn’s disease (CD) ; MedDRA version: 16.1 Level: PT Classification code 10011401 Term: Crohn's disease System Organ Class: 10017947 ‐ Gastrointestinal disorders Therapeutic area: Diseases [C] ‐ Digestive System Diseases [C06] SECONDARY OUTCOME: Secondary end point(s): Compare the efficacy of infliximab with that of placebo in the prevention of endoscopic recurrence of CD : Presence or absence of mucosal inflammation and ulceration will be evaluated via video ileocolonoscopy at Week 76, and to ensure that there is no recurrence of Crohn's Disease in the colon Timepoint(s) of evaluation of this end point: Week 76 INCLUSION CRITERIA: Have a documented diagnosis of CD confirmed by endoscopic, histologic and/or radiologic studies prior to resection or by tissue obtained at resection; Have undergone an ileocolonic surgical resection; Patients must also be at an increased risk of recurrence of active CD; Patients must not have previously discontinued infliximab as a result of tolerability issues or they must be naive to treatment with infliximab. Provided patients meet the above criteria pertaining to infliximab, they are eligible to enroll if they received prior treatment with adalimumab and/or certolizumab. Patients must undergo screening for HBV; Baseline CDAI < 200; Have adequate blood and liver test values. Are the trial subjects under 18? no Number of subjects for this age range: 0 F.1.2 Adults (18‐64 years) yes F.1.2.1 Number of subjects for this age range 290 F.1.3 Elderly (>=65 years) no F.1.3.1 Number of subjects for this age range PRIMARY OUTCOME: Main Objective: The primary objective of this study will be to compare the efficacy of infliximab with that of placebo in the prevention of clinical recurrence of CD through Week 76, defined as a composite endpoint that requires endoscopic confirmation of recurrence, in patients who are at an increased risk of active CD recurrence following ileocolonic resection. Primary end point(s): The primary endpoint is clinical recurrence of CD prior to or at Week 76. Clinical recurrence is a composite endpoint defined by the following: ; • A = 70‐point increase from baseline in CDAI score; and ; • A CDAI score of = 200; and ; • Evidence of endoscopic recurrence. Endoscopic recurrence is defined as a Rutgeerts score of = i2 at the anastomotic site or its equivalent elsewhere in the GI tract; and ; • If in the opinion of the investigator, the patient’s symptoms are predominantly diarrheal, a negative stool test for C. difficile must be present to confirm the true CD nature of the flare. ; or ; • Developing a new draining external fistula. ; or ; • Re‐opening and draining of a previously existing external fistula. or • Developing a new internal fistula. ; or ; • Developing a new perianal abscess. ; or ; • Developing a new intra‐abdominal abscess more than 3 months after the date of the index surgery. Note that “baseline” CDAI refers to the CDAI collected during the screening period that qualified the patient for the study. ; Patients who meet the criteria for clinical recurrence at Week 76 or at any time point prior to Week 76 will be considered to have clinical recurrence through Week 76. ; Patients who initiate a prohibited CD‐related medication or have a prohibited use of a CD medication prior to Week 76 will be considered to have had clinical recurrence. ; Patients who have surgery for CD prior to Week 76 will be considered to have had clinical recurrence ; Secondary Objective: The major secondary objective will be to compare the efficacy of infliximab with that of placebo in the prevention of endoscopic recurrence of CD through Week 76, defined as a Rutgeerts score = i2 either at the anastomosis or elsewhere in the gastrointestinal [GI] tract, in patients who are at an increased risk of active CD recurrence following ileocolonic resection. Timepoint(s) of evaluation of this end point: Week 76

%Z https://trialsearch.who.int/Trial2.aspx?TrialID=EUCTR2010-018431-18-NL

Trial registry record

%U https://cochrane.66557.net/central/doi/10.1002/central/CN-01866557/full

%0 Journal Article

%A Grosse, C.

%A Christophersen, C.

%A Thin, L.

%A Lightowler, D.

%A Lo, J.

%A Devine, A.

%A Lawrance, I.

%D 2021

%T A lacto-ovo vegetarian diet in patients with mild to moderate ulcerative colitis improves clinical response: a pilot study

%V 36

%N SUPPL 3

%P 161

%8 2021-01-01

%R 10.1111/jgh.15616

%K *cancer patient; *lactoovovegetarian diet; *pilot study; *relapse; *ulcerative colitis; Adjuvant chemotherapy; Adult; Body mass; Clinical article; Conference abstract; Controlled study; Dietitian; Feces; Female; Fish; Gastrointestinal tract; Gene expression; Human; Male; Metabolome; Microbiome; Middle aged; Nonhuman; Proof of concept; Protein expression; Quality of life; Randomized controlled trial; Remission; Sample size

%X Background and Aim: Inflammatory bowel disease (IBD) imposes a significant burden on an individual's quality of life and the health care system. Most patients use dietary modifications to manage their symptoms, despite limited research to support these changes. AWesternized diet has been associated with increased IBD risk and relapse. There are emerging data to suggest a plant‐based diet will be beneficial to patients with IBD through improving clinical response, reducing inflammation, and restoring symbiosis. The aim of this pilot study was to perform a proof‐of‐concept evaluation of this hypothesis. Methods: Patients with mild to moderate ulcerative colitis were enrolled in an 8‐week dietary trial. The patients were educated on following a lacto‐ovo vegetarian diet by an accredited practicing dietitian and provided with a vegetarian meal kit for five dinners per week. A partial Mayo score, Pro 6 score, IBD Quality of Life (IBD‐Q) score, and Food‐related Quality of Life (FRQoL) score were recorded at baseline and Weeks 2, 4, and 8. Fecal calprotectin (FCP) and C‐reactive protein (CRP) levels were recorded at baseline and Weeks 4 and 8. Stool samples for microbiome and metabolome analysis were collected at baseline and completion of the study. Data were analyzed using general linear modeling and were adjusted for body mass index and age. Results: Fourteen patients (mean age, 50.8 years; 58% women) were enrolled. Two patients withdrew from the study due to the severity of their disease within the first 2 weeks. Eleven patients (92%) reported 100% compliance with the diet. One patient consumed fish on two celebratory occasions. Clinical response was achieved as seen by a significant drop in the partial Mayo score (mean at Week 0, 4.17 vs Week 8, 2.36; P = 0.003) (Table 1) and the Pro 6 score (mean at Week 0, 2.83 vs Week 8, 1.64; P = 0.004). Quality‐of‐life measures significantly improved, as seen in the IBD‐Q scores (mean at Week 0, 139.2, vs Week 8, 167.3; P = 0.002) and FRQoL scores (mean at Week 0, 74.2, vs Week 8, 90.6; P = 0.002), while there was no significant improvement in biochemical remission measured by FCP level (mean at Week 0, 448 μg/g, vs Week 8, 419 μg/g; P = 0.391) and CRP level (mean at Week 0, 4.89 μg/mL, vs Week 8, 1.64 μg/mL; P = 5.76). Clinical response and improved qualityof‐ life measures occurred within the first 2 weeks, and these responses were maintained for the rest of the study. Dietary and microbiome analysis is in progress. Conclusion: In this pilot study, we showed a clinically significant improvement in the partial Mayo, Pro 6, IBD‐Q, and FRQoL scores. Microbiome and metabolite analysis is expected to demonstrate a favorable composition change in the gut microbiome and increased levels of beneficial metabolites after following a plant‐based diet. The results of this study suggest that a plant‐based diet may have clinical implications as an adjunct therapy in patients with mild to moderate ulcerative colitis. Although this study is limited by its sample size, the concept warrants a larger randomized study, with a direct comparison to an omnivorous diet.

%Z Journal of gastroenterology and hepatology

Journal article; Conference proceeding

%U https://cochrane.66557.net/central/doi/10.1002/central/CN-02337855/full

%0 Journal Article

%A Albenberg, L.

%A Brensinger, C.

%A Wu, Q.

%A Gilroy, E.

%A Kappelman, M.

%A Sandler, R.

%A Lewis, J.

%D 2018

%T A diet low in red and processed meats does not reduce the rate of Crohn's disease flares in a randomized controlled trial: results of the food and Crohn's disease exacerbation study (FACES)

%V 154

%N 1

%P S3

%8 2018-01-01

%K *Crohn disease; *diet; *disease exacerbation; *processed meat; Adult; Cohort analysis; Conference abstract; Controlled study; Crohn Disease Activity Index; E‐mail; Feeding behavior; Female; Human; Internet; Major clinical study; Male; Prospective study; Questionnaire; Randomized controlled trial; Recurrent disease; Red meat; Relapse; Remission; Surgery

%X BACKGROUND: Diet may be an important factor in the natural history of Crohn's disease (CD). We sought to determine whether exclusion of red and processed meats reduces the risk of CD relapse in a prospective randomized controlled trial. METHODS: Adults with CD were recruited from CCFA Partners, an Internet‐based cohort of IBD patients. Individuals who were in remission, defined as abbreviated Crohn's Disease Activity Index (aCDAI) 150, on a biannual survey and who reported consumption of red meat at least once weekly were randomized to consume a minimum of 2 servings/week (Arm A) or not more than 1 serving per month (Arm B) of red or processed meat for 48 weeks. Eligible subjects then received an email invitation with a link to a study arm‐specific consent. The primary outcome was relapse of CD, defined as increase in aCDAI by 70 points and to >150. The secondary outcome wasmoderate/severe relapse, defined as an increase in aCDAI to >219 or a need for CD surgery or new CD medication. RESULTS: 659 individuals were randomized of whom 214 consented (n=118 in arm A and n=96 in Arm B). Baseline characteristics were similar in each arm and among those who consented vs. non‐participants. Comparison of baseline nutrient intake on Diet History Questionnaire II, using PERMANOVA, showed no differences between the two groups (p=0.152). Adherence to the high meat diet, determined by % weeks consuming 2+ servings of red or processed meat, was 98.5% compared to 57% adherence to the low meat diet. At least a mild relapse occurred in 59% of patients during the study. There were no significant differences in time to overall (Fig 1, p=0.61) or moderate/severe (Fig 2, p=0.50) relapse. CONCLUSIONS: In patients with CD in remission, there was no difference in time to relapse in patients assigned to a high vs. low red and processed meat diet, although adherence was only modest in the low meat group. (Figure Presented).

%Z Gastroenterology

Journal article; Conference proceeding

%U https://cochrane.66557.net/central/doi/10.1002/central/CN-01570856/full

%0 Journal Article

%A Albenberg, L.

%A Brensinger, C.

%A Wu, Q.

%A Gilroy, E.

%A Kappelman, M.

%A Sandler, R.

%A Lewis, J.

%D 2018

%T A diet low in red and processed meats does not reduce the rate of Crohn's disease flares in a randomized controlled trial: results of the food and Crohn's disease exacerbation study (faces)

%V 24

%P S2‐S3

%8 2018-01-01

%R 10.1093/ibd/izy019

%K *Crohn disease; *diet; *disease exacerbation; *processed meat; Adult; Cohort analysis; Conference abstract; Controlled study; Crohn Disease Activity Index; E‐mail; Feeding behavior; Female; Human; Internet; Major clinical study; Male; Prospective study; Questionnaire; Randomized controlled trial; Recurrent disease; Red meat; Relapse; Remission; Surgery

%X Background: Diet may be an important factor in the natural history of Crohn's disease (CD). We sought to determine whether exclusion of red and processed meats reduces the risk of CD relapse in a prospective randomized controlled trial. Methods: Adults with CD were recruited from CCFA Partners, an Internet‐based cohort of IBD patients. Individuals who were in remission, defined as abbreviated Crohn's Disease Activity Index (aCDAI) ≤ 150, on a biannual survey and who reported consumption of red meat at least once weekly were randomized to consume a minimum of 2 servings/week (Arm A) or not more than 1 serving per month (Arm B) of red or processed meat for 48 weeks. Eligible subjects then received an email invitation with a link to a study arm‐specific consent. The primary outcome was relapse of CD, defined as increase in aCDAI by 70 points and to >150. The secondary outcome was moderate/severe relapse, defined as an increase in aCDAI to >219 or a need for CD surgery or new CD medication. (Figure presented) Results: 659 individuals were randomized of whom 214 consented (n=118 in arm A and n=96 in Arm B). Baseline characteristics were similar in each arm and among those who consented vs. non‐participants. Comparison of baseline nutrient intake on Diet History Questionnaire II, using PERMANOVA, showed no differences between the two groups (p=0.152). Adherence to the high meat diet, determined by % weeks consuming 2+ servings of red or processed meat, was 98.5% compared to 57% adherence to the low meat diet. At least a mild relapse occurred in 59% of patients during the study. There were no significant differences in time to overall (Fig 1, p=0.61) or moderate/severe (Fig 2, p=0.50) relapse. Conclusions: In patients with CD in remission, there was no difference in time to relapse in patients assigned to a high vs. low red and processed meat diet, although adherence was only modest in the low meat group.

%Z Inflammatory bowel diseases

Journal article; Conference proceeding

%U https://cochrane.66557.net/central/doi/10.1002/central/CN-01571654/full

%0 Journal Article

%A Albenberg, L.

%A Brensinger, C. M.

%A Wu, Q.

%A Gilroy, E.

%A Kappelman, M. D.

%A Sandler, R. S.

%A Lewis, J. D.

%D 2019

%T A Diet Low in Red and Processed Meat Does Not Reduce Rate of Crohn's Disease Flares

%V 157

%N 1

%P 128‐136.e5

%8 2019-01-01

%R 10.1053/j.gastro.2019.03.015

%K *Crohn disease; *diet; *disease exacerbation; *processed meat; Adult; Article; Cohort analysis; Controlled study; Crohn Disease Activity Index; Crohn Disease [*diet therapy, prevention & control]; Diet; Disease Progression; Female; Human; Humans; Internet; Kaplan‐Meier Estimate; Major clinical study; Male; Meat Products; Middle Aged; Principal Component Analysis; Proportional Hazards Models; Randomized controlled trial; Recurrence; Red Meat; Red meat; Relapse; Remission; Surgery

%X BACKGROUND & AIMS: Diet may be an important factor in the progression of Crohn's disease (CD). We performed a randomized controlled trial to determine whether reduced consumption of red and processed meats decreases the risk of symptomatic relapse of CD, analyzing results from the Food and Crohn's Disease Exacerbation Study (FACES) trial. METHODS: Adults with CD were recruited into the FACES trial from IBD Partners, an Internet‐based cohort of patients with inflammatory bowel disease, from November 2013 through June 2015. Individuals who were in remission (CD activity index [sCDAI] scores of ≤150), had completed a biannual survey, and reported consumption of red meat at least once weekly were randomly assigned to groups that consumed a minimum of 2 servings/week of red or processed meat (high meat, n = 118) or not more than 1 serving per month (low meat, n = 96) for 49 weeks. The primary outcome was relapse of CD, defined as increase in sCDAI score by ≥70 points and to >150 or a need for CD surgery or new CD medication. A secondary outcome, moderate or severe relapse, was based on an increase in sCDAI to >219. RESULTS: During the trial, the high‐meat groups reported consumption of 2 or more servings of red or processed meat during 98.5% of observed weeks compared with 18.8% of weeks for the low‐meat group. Any and moderate to severe relapse occurred in 62% of participants in the high‐meat group and 42% of participants in the low‐meat group. There were no significant differences in time to any (P = .61) or moderate/severe (P = .50) relapse. CONCLUSIONS: In an analysis of data from the FACES trial, we found that among patients with CD in remission, level of red and processed meat consumption was not associated with time to symptomatic relapse. ClinicalTrials.gov, Number: NCT0192673.

%Z Gastroenterology

Journal article

%U https://cochrane.66557.net/central/doi/10.1002/central/CN-01936494/full

%0 Journal Article

%A NCT

%D 2011

%T A Comparison of Narrow Band Imaging (NBI) and Standard White Light Laporoscopy to Detect Endometriosis

%8 2011-01-01

%K Endometriosis

%X Introduction: Endometriosis is a relatively common chronic gynecological condition that affects approximately 10% of all women of reproductive age. It is a pelvic inflammatory disease that is characterized by the presence of endometrial glands and stroma outside of the uterine cavity. Typical symptoms of endometriosis include dysmenorrhea, pelvic pain, and infertility; the severity of pain associated with this disease often leads to a considerable decrease in quality of life. The standard treatment for severe pelvic pain and infertility is to surgically remove endometriotic areas. Identifying all endometriotic lesions is paramount to "optimal endometriosis debulking." The inability to see all endometriosis lesions has been thought to be a factor for patients with little or no relief following surgery. Using the Narrow Band Imaging (NBI) method has the potential to improve visualization of endometriosis lesions, assist in debulking and thus, result in improved clinical outcomes. NBI is a technique that uses a specific narrow wavelength of light to change the normal color contrasts of the endoscopic image and improve detection of neovascularization, which is the pathological feature of endometriosis for both superficial and deeper vascularization. This type of imaging has the potential to offer improved discrimination of lesions, increasing diagnostic yield as well as resulting in more complete debulking. The results of a pilot study of 21 patients conducted at Mercy Medical Center demonstrated that use of NBI resulted in the diagnosis of pathologically‐proven endometriosis among 4 patients whose lesions were missed with visible white light only examination. In addition, the number of confirmed endometriosis lesions increased with the use of NBI in addition to white light. The study also suggested that NBI may assist in discriminating endometriosis from other lesions, thus reducing false positives: one patient had no lesions detected with NBI and the lesions biopsied based on visible white light examination were not endometriosis at pathologic examination. The investigators will conduct a multi‐center study with the following primary aims to determine the degree to which NBI improves the detection and diagnosis of endometriosis lesions. 1. Determine the degree to which NBI improves the diagnosis of endometriosis (diagnostic yield) of laparoscopic examinations compared to use of visible white light‐only laparoscopy. 2. Determine if NBI improves sensitivity in the detection of potential endometriosis lesions and reduces false positives compared to visible white light. Secondary aim: Determine the impact of the use of NBI, and presumed more complete removal of endometriotic lesions, compared to use of white light examination on reported severity of pain at 6‐weeks, 3‐months, and 6 months following surgery. The results of this study would significantly advance the state of art for the diagnosis and treatment of endometriosis. Improved detection and treatment of endometrial lesions should translate to a significant health benefit to women suffering from endometriosis. Potential health benefits include improved diagnosis of the etiology of pelvic pain, therefore guiding appropriate treatment management for women with pelvic pain due to endometriosis and improved debulking of lesions. Study Aims Hypotheses: The use of NBI in addition to white light examination will improve the diagnostic yield of endometriotic lesions at the time of laparoscopy compared to only using white light examination. Study Outcomes: Methods: Investigator Training: The investigators will be trained on the visualization of endometriosis under NBI ‐ Viewing WL and NBI images of potential endometrial lesions of various types Lesion discrimination under white light examination based on lesion color has been previously described in the literature. Red (also known as clear and/or pink) lesions are considered to be active and early lesions on endometriosis. White lesions may indicate areas of fibrosis and may be latent or dormant lesion of endometriosis. The red and white lesions are also known as the "subtle" lesions of endometriosis. Black (also known as brown, chocolate green, purple or blue) represent a more severe stage of the disease. Black lesions are also known as "gunshot" or "powder‐burn" lesions and are considered the "typical" lesions of endometriosis. Mixed color lesions can include a mixture of any color. It has been speculated that the subtle lesions of endometriosis are more likely to be positive lesions and to be missed under white light examination. The red lesions are also considered to have more vessels of smaller diameter and may also include peritoneal microlesions and therefore may be more easily detected under NBI. The proposed study design is a multisite randomized controlled device trial conducted in two clinical centers. Women will be randomized to have laparoscopic examination with white light followed by NBI (white light/NBI) examination or white light followed by repeat white light examination (white light/white light). Women of reproductive age (<50 years of age) undergoing diagnostic laparoscopy for pelvic pain, suspected endometriosis, or infertility will be eligible. A systematic examination covering the four quadrants of the pelvis will be conducted using white light followed by either NBI or white light to identify and document endometriotic lesions. The use of NBI in addition to white light examination will improve the sensitivity of detecting endometriotic lesions and reduce false positives at laparoscopy compared to only using white light examination. Secondarily, the use of NBI will be associated with a greater reduction in pain at the 6‐week, 3‐month, and 6‐month follow‐up compared to the use of white light examination alone because of improved lesion identification and debulking. Pass/Fail Criteria for Primary Aims. In this randomized clinical trial, failure is defined as no statistically significant difference in the primary outcomes of diagnostic yield or sensitivity between the white only examination arm and the white light followed by NBI examination arm. Study Design and Methods The investigators will test the hypotheses by conducting a multicenter device trial. Participants will be randomized to either white light/NBI or white light/white light at a 3:1 ratio. In both study groups, each of the quadrants of the pelvis will first be examined by white light. Once a full exam is performed using the white light with lesion location documented with photographs, a randomization envelope will be opened to reveal the patient's second modality (NBI or white light). Each area will then be inspected by this second modality with photographs taken for each quadrant, documenting lesion location. All identified lesions in areas amenable to resection will then be resected, labeled by method of detection, and sent for pathologic examination. ‐ The procedural use of NBI ‐ Completion of study forms to ensure data integrity Study Population: The population will include women of reproductive age undergoing diagnostic laparoscopy for suspected endometriosis. The study population may include: ‐ Patients undergoing initial diagnostic laparoscopy for pelvic pain, suspected endometriosis or infertility ‐ Patients previously evaluated with laparoscopy undergoing repeat laparoscopy for suspected recurrence or onset of endometriosis Recruitment: Equipment: ‐ SONY HD recorder PDW‐70MD The primary outcomes are the comparison of the diagnostic yield, sensitivity, and false positive rate of visible white light (white light/white light) versus NBI (white light/NBI) based on pathology gold standard. The participating surgeons and assistants from each site will be trained on the procedural use of NBI by Olympus and the coordinating center. A minimum of three cases of patients suspected to be positive for endometriosis will be watched by surgeons and coordinators from remaining sites. The training surgeon will perform the sweep through the quadrants of the pelvis. Each area will be viewed under white light. The training surgeon will ask the surgeons to identify suspicious areas that they would biopsy. Then the training surgeon will switch to NBI and once again ask the training surgeons to identify suspicious areas that they would biopsy. This will be done to ensure that all participating surgeons are able to identify suspicious areas under both imaging modalities to learn what potential endometrial lesions look like under NBI. Olympus will visit each site prior to the first procedure and train the assistant(s) on the completion of the study case report forms. Olympus will also monitor adherence to the protocol and the completion of the first study case report forms during the time of study start (first patient enrollment) to ensure the surgeon is performing the procedure according to the protocol and that the assistant is accurately capturing all of the required information. Women will be recruited through the gynecologic practices of the participating physicians. At the pre‐surgery visit, patients will be informed of the purpose of the study as well as the risks and benefits of the procedure and will be asked to sign an informed consent. After signing the informed consent, women will be asked to complete a baseline questionnaire to obtain information on demographic characteristics, medication history, and symptoms. A 10‐centimeter (cm) visual analog scale and a validated endometriosis symptom questionnaire [the Endometriosis Health Profile‐30 (EHP‐30)] will also be administered to assess pain. The EHP‐30 is an instrument specifically designed to measure pain and quality of life among women diagnosed with endometriosis. The EHP‐30 consists of a core questionnaire with 30 items and five scales and six modular parts consisting of 23 questions. The EHP‐30 has demonstrated both high reliability and validity. ‐ OLYMPUS HD EndoEYE Video Telescope (Models WA50011A, WA50013A, WA50013L, WA50013T, WA50015L) or OLYMPUS HD EndoEYE Laparo‐Thoraco Videoscope LTF‐VHOLYMPUS CLV‐180 EVIS EXERA II Xenon Light Source OLYMPUS CV‐180 EVIS EXERA II Video System Center OLYMPUS HD LCD Surgical Monitor ‐ Printers OEP‐4 Examination Procedures: The laparoscopic examination will be conducted in a standardized fashion with visible white light (standard), followed by an additional sweep with either white light (control) or NBI. The second look examination will be determined by random assignment after completion of the first examination with white light. The examination will include visualizing each quadrant of the pelvis (Right Anterior, Right Posterior, Left Posterior, Left Anterior). Each area will first be viewed and photographed under white light. The absence or presence of suspected endometrial lesions will be noted. If suspected lesions are seen, the number of lesions, the extent of the lesions and the surgeon's confidence in the lesion being endometriosis will be recorded. Photographs will be labeled by quadrant and all lesions will be circled and numbered. Clinicians will record their degree of confidence on a 5 point likert scale that the lesion to be excised is a lesion of endometriosis. Following visualization, photographic identification and documentation of the quadrants under white light has been completed, the study coordinator will open the randomization envelope and reveal the second modality to be used. The physician will then repeat the exam and photographs with either white light (control) or under NBI (switching on NBI). Again, under the second modality, the absence or presence of suspected endometrial lesions will be noted. If suspected lesions are seen, the number of lesions, the extent of the lesions, the lesion color and the surgeon's confidence in the lesion being endometriosis will be recorded. A photograph will be taken, labeled and all lesions will be circled and numbered. Clinicians will record their degree of confidence on a 5 point likert scale that the lesion to be excised is a lesion of endometriosis. The surgeon will record if the lesion was also visualized under the 1st imaging modality or under only one of the modalities. The order at which the areas are examined through the sweep will be at the surgeon's discretion. Examination by both sweeps will be photographed. Photos of all areas and detected lesions will be printed, labeled and lesions will be circled and numbered. All suspected endometrial lesions will be included in the analysis, even lesions that may be latent or healed (otherwise known as white lesions). Once both exams are complete, additional areas of the abdomen can be evaluated at the surgeon's discretion; those randomized to white light only will continue with only white light examination. All lesions detected by either method that are amenable to resection will be numbered and biopsied. Data collected will include location, method of detection and clinical diagnosis confidence score. Pathologists will be masked to method of detection (NBI versus white light). Lesions will be recorded according as falling within three categories: visible white light‐detected only; NBI‐detected only; or both NBI‐ and visible white light‐detected. Pathology will be performed at each center following a standardized protocol for the diagnosis of endometriosis. Pathologists at each institution will be masked to the method of examination used to identify the specimen. All discordant lesions (visualized under only one imaging modality), a random 10% sample of specimens and a random 10% of images from each center will be re‐reviewed by a central independent reviewer at the coordinating center. Lesions that are not amendable to resection will be photographed only and will not be included in the data analysis. Data Collection: 1. Patient baseline questionnaire: symptoms, medication history, demographic information, pain measurement (including 10‐cm visual analog scale and EHP‐30) 2. Photo documentation of each light examination 4. Pathology reports 5. Medical record review 6. Provider questionnaire: planned follow‐up treatment after surgery 7. Follow‐up questionnaires for pain at 6‐weeks, 3‐months and 6‐months; follow‐up surveys will include both the 10‐cm visual analog scale and the EHP‐30 Quality Control: Sample size Sample size calculations from original protocol, dated 5/14/2010 Data used for sample size calculations: 3. Case report forms: ‐ Recording of location of lesion by method of examination ‐ Notation of lesion color ‐ Clinical impression of detected lesions ‐ degree of confidence on a scale of 1 to 5 that the lesions observed are endometriosis ‐ Notation of lesions excised ‐ Photographic documentation of lesions ‐ Change in surgical treatment (larger excision/smaller excision/organ removal) based on results of visualization under the different imaging modalities. Quality control will be maintained by standardizing the protocols across the centers. As stated above, in each patient, the locations of all lesions suspected to be endometriosis detected with each modality will be documented with photographs. All discordant lesions (seen under only one imaging modality) and a random 10% sample of the images and specimen samples from each center will be re‐reviewed by a central independent reviewer at the coordinating center. The results between the site physician and the central reviewer will be compared. If the site and central reviewer have disagreement on the photographed lesion or pathology, adjudication will be performed between the site and the central reviewer. If a consensus still cannot be reached, a third party (surgeon and/or pathologist (from the remaining site) will be used as the tie breaker. Randomization Procedure Subjects will be assigned to one of two groups in a stratified randomized design. All subjects will undergo a white light examination followed by either a second examination with white light or an examination using NBI. Investigators will be blinded to second look imaging method until after the initial white light examination is complete. Randomization to either white light/NBI or white light/white light will occur at a 3:1 ratio. Each site is treated as independent and subjects will be combined across sites for analyses. A randomization table will be generated using Microsoft Excel at the beginning of study recruitment. The randomization number will be a three digit number and will form the last 3 digits of the study ID. Sealed envelopes will be provided to each site by the coordinating center to be opened after the initial white light examination. Sample size in the original protocol for this study was calculated as described in section 12.1 below. Because data from a third site of this multicenter trial (Atlanta Northside) will not be used, the total sample size goal (N = 170) in the original protocol will not be reached. Mercy was approved by the IRB in September 2012 to enroll 100 patients total; the Chicago site is approved to enroll 57 patients. Thus, the total sample size goal has been revised to 157; minimal detectable differences under the conditions of 90% power are described in section 12.2 below. Sample size calculations were based on the primary outcomes of diagnostic yield and sensitivity as well as the secondary outcome of impact on pain symptoms. The sample size is largely driven by the primary outcome of diagnostic yield. A sample size of 170 will provide 90% power to detect an improvement in diagnostic yield of 20% (95% CIs 72%‐85% white light and 95%‐100% NBI), based on a randomization scheme of 3:1 for white light/NBI versus white light/white light examination. This sample size takes into consideration a 25% drop out/not evaluable rate for the following reasons: incomplete visualization of the pelvis, loss to follow‐up for pain assessment or patients not evaluable for pain assessment (a subset of infertility patient who may not have pain as a presenting symptom). Sensitivity and false positive rate: Pain difference: Protocol Deviations The IRB (and other review boards as required for this study) will be notified of any protocol deviations. This sample size provides 90% power to detect an average increase of one additional endometriosis lesion with the NBI examination (white light/NBI group) compared to white light only examination (white light/white light group). To note, this power calculation was based on data from the pilot study that showed an average of 2 lesions (per patient) found using white light and 3 lesions (per patient) detected using NBI; the calculation assumes a common standard deviation of 1. Diagnostic yield: The calculations for sample size were based on information obtained from a pilot study that examined patients using white light followed by NBI during diagnostic laparoscopy. In that study, 4 additional patients were diagnosed with endometriosis with the addition of NBI to white light examination for an absolute improvement of 27% (4/15 patients with endometriosis were found with NBI examination only) (Barrueto and Audlin, 2008). Using these data, sample size calculations were conducted using nQuery 6.01 (Elashoff, 2005) for a two group chi‐square test with unequal Ns (3:1 ratio). A two group chi‐square test with a 0.05 two‐sided significance level will have 90% power to detect the difference between a Group 1 (white light/white light) diagnostic yield of 0.79 and a Group 2 (white light/NBI) diagnostic yield of 0.99 when the sample sizes in each group are 34 and 101, respectively (a total sample size of 135). In the pilot study, 15 of 20 patients examined were diagnosed with endometriosis on histology. All 15 patients were detected with NBI and white light. Four patients were diagnosed by lesions that were detected only with NBI. Based on these data, the estimated sensitivities of white light and NBI are 73% and 100% respectively. Given these sensitivities and the small total sample size, 95% confidence intervals are wide (48%‐90% white light and 80%‐100% NBI). At the lesion level, pilot data have shown an average of 2 lesions seen under white light compared to an average of 3 lesions using NBI. Using a common standard deviation of 1, the current sample size has at least 90% power to detect an average difference of at least one additional lesion. Hard copies of questionnaires and interview records will be locked in file cabinet with limited keyed access to research personnel. Computer data files are also kept on the Mercy Medical Center Intranet in a password protected file that is accessible only by research staff. White light examination is the current standard of care. NBI itself does not incur any additional risk. However, the use of NBI may result in the identification of additional lesions and thus incur the risk of additional resection at the time of laparoscopy. Additional sites identified for biopsy using NBI have no additional risks for removal as compared to white light. Lesions identified which are associated with higher risk structures such as the bowel and the diaphragm will not be removed. It is uncertain to what extent participants may benefit clinically and this will be clearly stated in the consent form. If diagnosis of endometriosis is improved, then patients may benefit from improved medical management. If additional lesions are identified and resected, patients may benefit from an improved clinical outcome. The study will also consider pain reduction as a clinically relevant outcome. A recently published study by Abbott et al (2003) reported that participants undergoing laparoscopy for potential endometriosis on average rated non‐menstrual pelvic pain prior to surgery as 6 on a 10‐point visual analogue scale, with 10 being the worst pain. In the Abbott et al (2003) and other similar studies, laparoscopic surgery using white light was shown to be associated with a 30% reduction in pain at 6 month follow up and 50%‐75% at 12 month follow up with increasing variance (Abbott et al., 2004, Jarrell et al., 2005). An RCT from Jarrell et al. (2005) showed a reduction in pain score of 1.9 (SD 0.5) at 6 month follow up and 2.6 (SD 0.5) at 12 month follow up, although the sample size of this study was small (N=16 overall, N=7 for laparoscopy arm). This study will test the hypothesis that NBI will result in an additional 20% decrease in pain compared to what has been observed in these previously published studies examining the reduction in pain using white light laparoscopy. Using the unbalanced randomization design with 1 patient randomized to white light/white light for every 3 patients randomized to white light/NBI , a two group t‐test with a 0.050 two‐sided significance level will have 90% power to detect the difference in pain change with a Group 1 (white light/white light) mean change of 1.9 and a Group 2 (white light/NBI) mean of 2.28, a difference in means of 0.38 with a common SD of 0.5, when the sample sizes in the two groups are 25 and 75, respectively (a total sample size of 100). We project that the majority of patients enrolled (over 70%) will be symptomatic with pelvic pain despite the indication for surgery (i.e. pelvic pain or infertility). Only 1 of the 2 study center sites has a significant population of women undergoing laparoscopy for evaluation and treatment of infertility. Amended sample size The sample size of the study has been adjusted downward to 157 due to the loss of one of the study centers for accrual. The revised sample size considers the outcomes of diagnostic yield and sensitivity of clinical determination of identified lesions at the time of surgery, by modality. A review of the first 69 patients enrolled and assigned to the combined white light/NBI arm shows an average detection rate for any lesion to be 4.2 per patient using NBI versus 2.9 per patient with white light. The minimal detectable difference in diagnostic yield and sensitivity was determined using nQuery 6.01 (Elashoff, 2005). With the current projected sample size of 157 patients, the minimal detectable difference between the two modalities, with 90% power, in diagnostic yield proportions and sensitivity estimates is 12.2%. Thus, the power of the study remains sufficient to detect a clinically meaningful difference in detection of endometriosis by white light and NBI. Data Management A copy of the consent form will be sent to the Data Coordinating Center (DCC). Report forms will be sent by fax or electronically (secured/encrypted) to the DCC data entry. The data will be checked for consistency by using selected double entry where 1/10th of the entered data will be re‐entered and checked for errors. Data cleaning procedures will be done using SAS with extensive program files to ensure the correctness of skip patterns and responses. After baseline information is collected and entered, an analysis will be done to confirm comparable baseline characteristics across the two groups. Confidentiality: Any results from this study will be published using data from the group as a whole. No individual will be identified in any reports. Risks/Benefits Assessment Risks/Discomforts: Benefits: Modifications to the Protocol Any modifications to the protocol will be sent to the IRB for approval. Data Analysis The investigators hypothesize that NBI will improve detection of endometriosis lesions thus improving the diagnosis of endometriosis. For the primary outcome of diagnostic yield, the reference standard will be the pathologic confirmation of excised lesions where any one lesion within an individual detected by either method will confirm the diagnosis of endometriosis. The investigators will address the detection of endometriosis by comparing the diagnostic yield of the white light/white light group versus the white light/NBI group. The investigators will use a McNemar's test for the proportion comparison, which accounts for the pairing of assessment within a participant and makes no assumptions about the distribution of samples. At the lesion level, the investigators will assess the average change in the number of lesions detected between the white light/white light group to the white light/NBI group using independent samples t‐test. The investigators will also calculate and compare the sensitivities and false positive rates for the white light/white light group and the white light/NBI group. Pain across the study period will be assessed by comparing average change in pain score (using both the VAS and the EHP‐30 scores) between the two groups. The difference in average pain change scores will be assessed using an independent samples t‐test. Differences in average pain between baseline and follow‐up will be assessed using paired t‐tests. Withdrawal from protocol All participants are informed that taking part in this study is completely voluntary and they have the right to not participate. If they do not want to join the study, or if they wish to withdraw from the study, they will still receive the same quality of medical care. Reporting of Serious Adverse Events and Unanticipated Problems The protocol has minimal additional risks beyond the usual risks associated with diagnostic laparoscopy. Should any unanticipated problems develop, the investigators will attend to/address the problem and notify institutional IRBs and the sponsor.

%Z https://clinicaltrials.gov/show/NCT01464775

Trial registry record

%U https://cochrane.66557.net/central/doi/10.1002/central/CN-01533692/full

%0 Journal Article

%A Feagan, B. G.

%A Fedorak, R. N.

%A Irvine, E. J.

%A Wild, G.

%A Sutherland, L.

%A Steinhart, A. H.

%A Greenberg, G. R.

%A Koval, J.

%A Wong, C. J.

%A Hopkins, M.

%A Et, Al.

%D 2000

%T A comparison of methotrexate with placebo for the maintenance of remission in Crohn's disease. North American Crohn's Study Group Investigators

%V 342

%N 22

%P 1627‐1632

%8 2000-01-01

%R 10.1056/NEJM200006013422202

%K Adult; Crohn Disease [*drug therapy]; Double‐Blind Method; Female; Glucocorticoids [therapeutic use]; Humans; Immunosuppressive Agents [adverse effects, *therapeutic use]; Injections, Intramuscular; Male; Methotrexate [adverse effects, *therapeutic use]; Prednisone [therapeutic use]; Recurrence; Remission Induction

%X BACKGROUND: Patients with Crohn's disease often have relapses. Better treatments are needed for the maintenance of remission. Although methotrexate is an effective short‐term treatment for Crohn's disease, its role in maintaining remissions is not known. METHODS: We conducted a double‐blind, placebo‐controlled, multicenter study of patients with chronically active Crohn's disease who had entered remission after 16 to 24 weeks of treatment with 25 mg of methotrexate given intramuscularly once weekly. Patients were randomly assigned to receive either methotrexate at a dose of 15 mg intramuscularly once weekly or placebo for 40 weeks. No other treatments for Crohn's disease were permitted. We compared the efficacy of treatment by analyzing the proportion of patients who remained in remission at week 40. Remission was defined as a score of 150 or less on the Crohn's Disease Activity Index. RESULTS: Forty patients received methotrexate, and 36 received placebo. At week 40, 26 patients (65 percent) were in remission in the methotrexate group, as compared with 14 (39 percent) in the placebo group (P=0.04; absolute reduction in the risk of relapse, 26.1 percent; 95 percent confidence interval, 4.4 percent to 47.8 percent). Fewer patients in the methotrexate group than in the placebo group required prednisone for relapse (11 of 40 [28 percent] vs. 21 of 36 [58 percent], P=0.01). None of the patients who received methotrexate had a severe adverse event; one patient in this group withdrew because of nausea. CONCLUSIONS: In patients with Crohn's disease who enter remission after treatment with methotrexate, a low dose of methotrexate maintains remission.

%Z New England journal of medicine

Journal article

%U https://cochrane.66557.net/central/doi/10.1002/central/CN-00277986/full

%0 Journal Article

%A Feagan, B. G.

%A Fedorak, R. N.

%A Irvine, E. J.

%A Wild, G.

%A Sutherland, L.

%A Steinhart, A. H.

%A Greenberg, G. R.

%A Koval, J.

%A Wong, C. J.

%A Hopkins, M.

%A Et, Al.

%D 2000

%T A comparison of methotrexate with placebo for the maintenance of remission in Crohn's disease

%V 342

%N 22

%P 1627‐1632

%8 2000-01-01

%R 10.1056/NEJM200006013422202

%K *Crohn disease/di [Diagnosis]; *Crohn disease/dt [Drug Therapy]; *methotrexate/ae [Adverse Drug Reaction]; *methotrexate/cm [Drug Comparison]; *methotrexate/ct [Clinical Trial]; *methotrexate/dt [Drug Therapy]; *methotrexate/im [Intramuscular Drug Administration]; Adult; Article; Clinical article; Clinical trial; Controlled clinical trial; Controlled study; Data analysis; Disease activity; Dose response; Drug withdrawal; Human; Nausea/si [Side Effect]; Placebo; Prednisone/do [Drug Dose]; Prednisone/dt [Drug Therapy]; Priority journal; Randomized controlled trial; Remission; Wellbeing

%X Background: Patients with Crohn's disease often have relapses. Better treatments are needed for the maintenance of remission. Although methotrexate is an effective short‐term treatment for Crohn's disease, its role in maintaining remissions is not known. Methods: We conducted a double‐blind, placebo‐controlled, multicenter study of patients with chronically active Crohn's disease who had entered remission after 16 to 24 weeks of treatment with 25 mg of methotrexate given intramuscularly once weekly. Patients were randomly assigned to receive either methotrexate at a dose of 15 mg intramuscularly once weekly or placebo for 40 weeks. No other treatments for Crohn's disease were permitted. We compared the efficacy of treatment by analyzing the proportion of patients who remained in remission at week 40. Remission was defined as a score of 150 or less on the Crohn's Disease Activity Index. Results: Forty patients received methotrexate, and 36 received placebo. At week 40, 26 patients (65 percent) were in remission in the methotrexate group, as compared with 14 (39 percent) in the placebo group (P=0.04; absolute reduction the risk of relapse, 26.1 percent; 95 percent confidence interval, 4.4 percent to 47.8 percent). Fewer patients in the methotrexate group than in the placebo group required prednisone for relapse (11 of 40 [28 percent] vs. 21 of 36 [58 percent], P=0.01). None of the patients who received methotrexate had a severe adverse event; one patient in this group withdrew because of nausea. Conclusions: In patients with Crohn's disease who enter remission after treatment with methotrexate, a low dose of methotrexate maintains remission.

%Z New England journal of medicine

Journal article

%U https://cochrane.66557.net/central/doi/10.1002/central/CN-00424036/full

%0 Journal Article

%A Stevens, T.

%A Haasnoot, L.

%A D'Haens, G. R.

%A Buskens, C. J.

%A de Groof, E. J.

%A Eshuis, E. J.

%A Gardenbroek, T. J.

%A Mol, B.

%A Stokkers, P.

%A Bemelman, W. A.

%A Et, Al.

%D 2020

%T 284 EARLY LAPAROSCOPIC ILEOCECAL RESECTION DECREASES NEED FOR MEDICAL TREATMENT AND SURGERY IN INFLAMMATORY ILEOCECAL CROHN'S DISEASE: LONG-TERM FOLLOW UP OF THE LIR!C TRIAL

%V 158

%N 6

%P S‐55‐S‐56

%8 2020-01-01

%R 10.1016/S0016-5085(20)30821-0

%K *Crohn disease; *follow up; Adult; Clinical trial; Conference abstract; Controlled study; Drug therapy; Female; Human; Kaplan Meier method; Major clinical study; Male; Multicenter study; Observational study; Randomized controlled trial; Retrospective study; Treatment duration; Treatment failure

%X Background: The LIR!C trial showed that laparoscopic ileocecal resection is cost‐effective and at least as good in terms of quality of life when compared to infliximab for ileocecal Crohn’s disease. Current aims were to compare outcomes of both treatment strategies beyond the one‐year endpoint of the trial and identify predictive factors associated with failure of the initial strategy. Methods: In this long‐term observational study data was retrospectively collected for patients that were included in the LIR!C trial; a multicenter, randomized controlled trial that compared a laparoscopic ileocecal resection with infliximab for adult patients with non‐stricturing and immunomodulator refractory ileocecal Crohn’s disease. Outcomes of interest were need for (re‐) surgery or anti‐TNF, time until need for first additional Crohn’s treatment as well as the identification of factors associated with the start of additional treatment. Time to additional treatment was assessed using Kaplan‐Meier analyses. Potential factors were defined a priori and analyzed by multivariable cox regression analysis. Results: Data were available for 134/143 (93.7%) patients of the LIR!C trial (resection, n=69; infliximab, n=65). Median [IQR] follow up time was 63.5 [39 – 94.5] months. In the resection group, 18 (26.1%) patients started anti‐TNF treatment after a median [IQR] of 25.5 [13 – 46.5] months. Importantly, none of the patients required a second resection (figure 1). 29 (42%) patients did not require any additional Crohn’s related treatment, although 37.7% did receive a prophylactic immunomodulator from enrollment onwards. Conversely, in the infliximab group, 31 (47.7%) patients underwent a Crohn’s related resection after a median of 17 [6 – 34] months. Duration of treatment effect was similar with a median (95% CI) time without need for additional treatment of 33 (15.1 – 50.9) and 34 (0 – 69.3) months in the resection and infliximab group respectively (log‐rank P = 0.521). In both groups prophylactic immunomodulators decreased the risk of additional treatment (HR 0.34 95%CI (0.16 – 0.69) and HR 0.49 95%CI (0.26‐0.93) respectively). Conclusion: This long term follow up study of the LIR!C trial showed that after a median follow up of 5 years the majority of patients who underwent a resection were free of anti‐TNF treatment whereas almost half of the infliximab group moved on to a resection. Importantly, almost half of the patients in the resection group did not have endoscopic or clinical recurrence requiring additional medical treatment and none required a second resection. These data support early ileocecal resection in inflammatory ileocecal Crohn’s disease failing conventional treatment.

%Z Gastroenterology

Journal article; Conference proceeding

%U https://cochrane.66557.net/central/doi/10.1002/central/CN-02132695/full
