# Supplementary material for: Risk factors for endoscopic postoperative recurrence in patients with Crohn’s Disease: a protocol for systematic review and meta-analysis
Source: BMC Gastroenterol. 2024 Jun 25;24:211. doi: 10.1186/s12876-024-03301-z (PMC11197377; doi:10.1186/s12876-024-03301-z)
Supplement: Supplementary file 2 — Supplementary Material 2 [file 12876_2024_3301_MOESM2_ESM.docx]

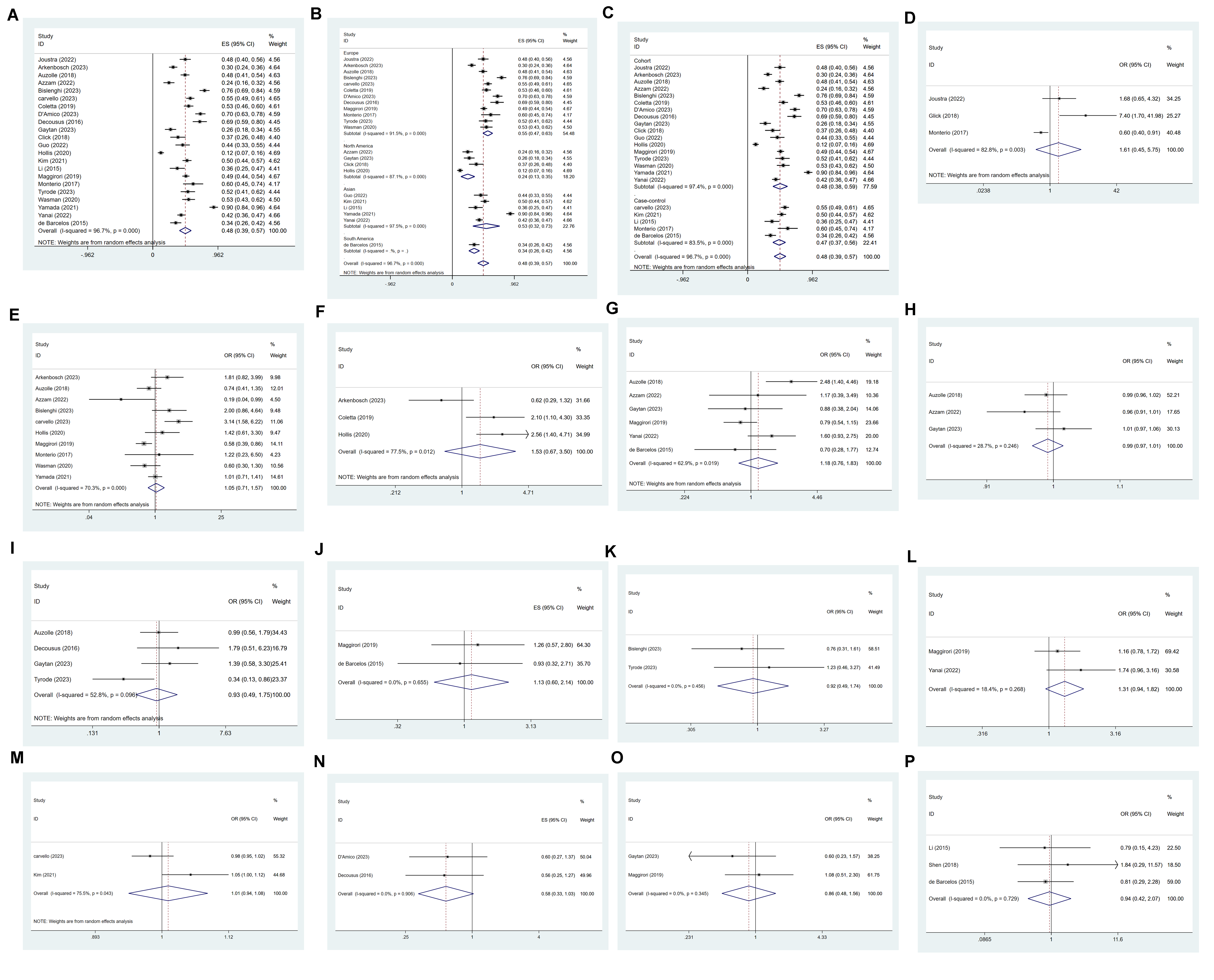


Table S1 Meta-analysis forest plot of incidence


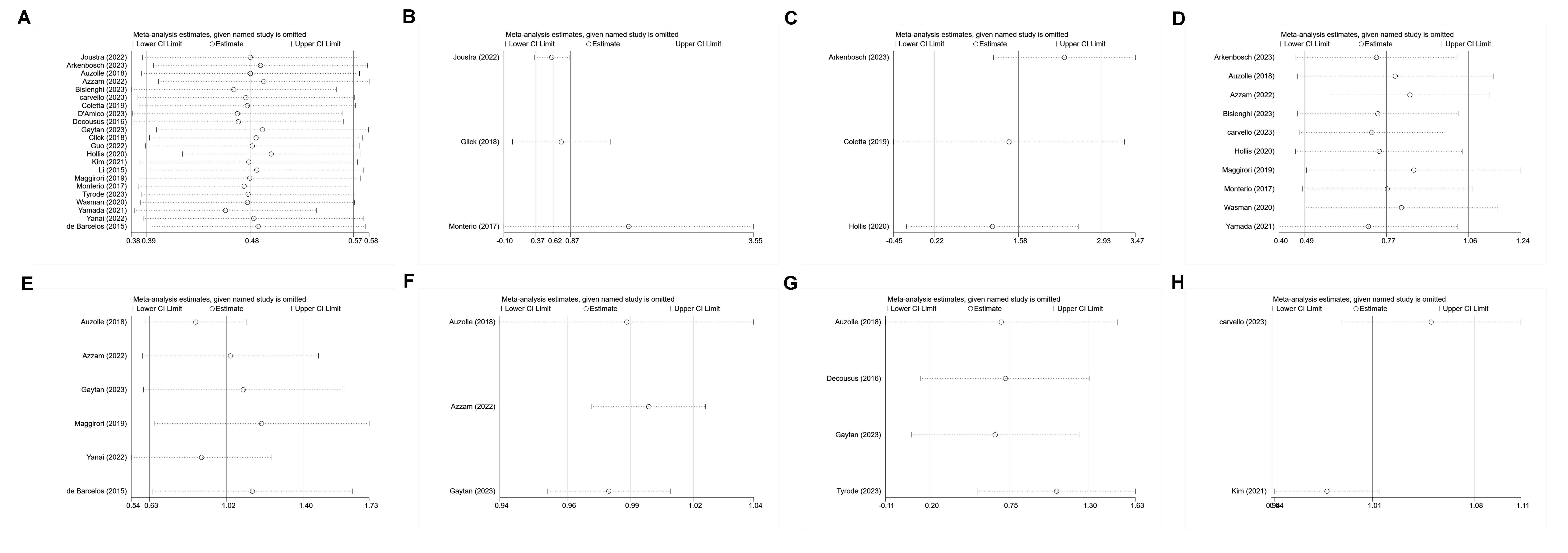


Table S2 Sensitivity analysis of incidence


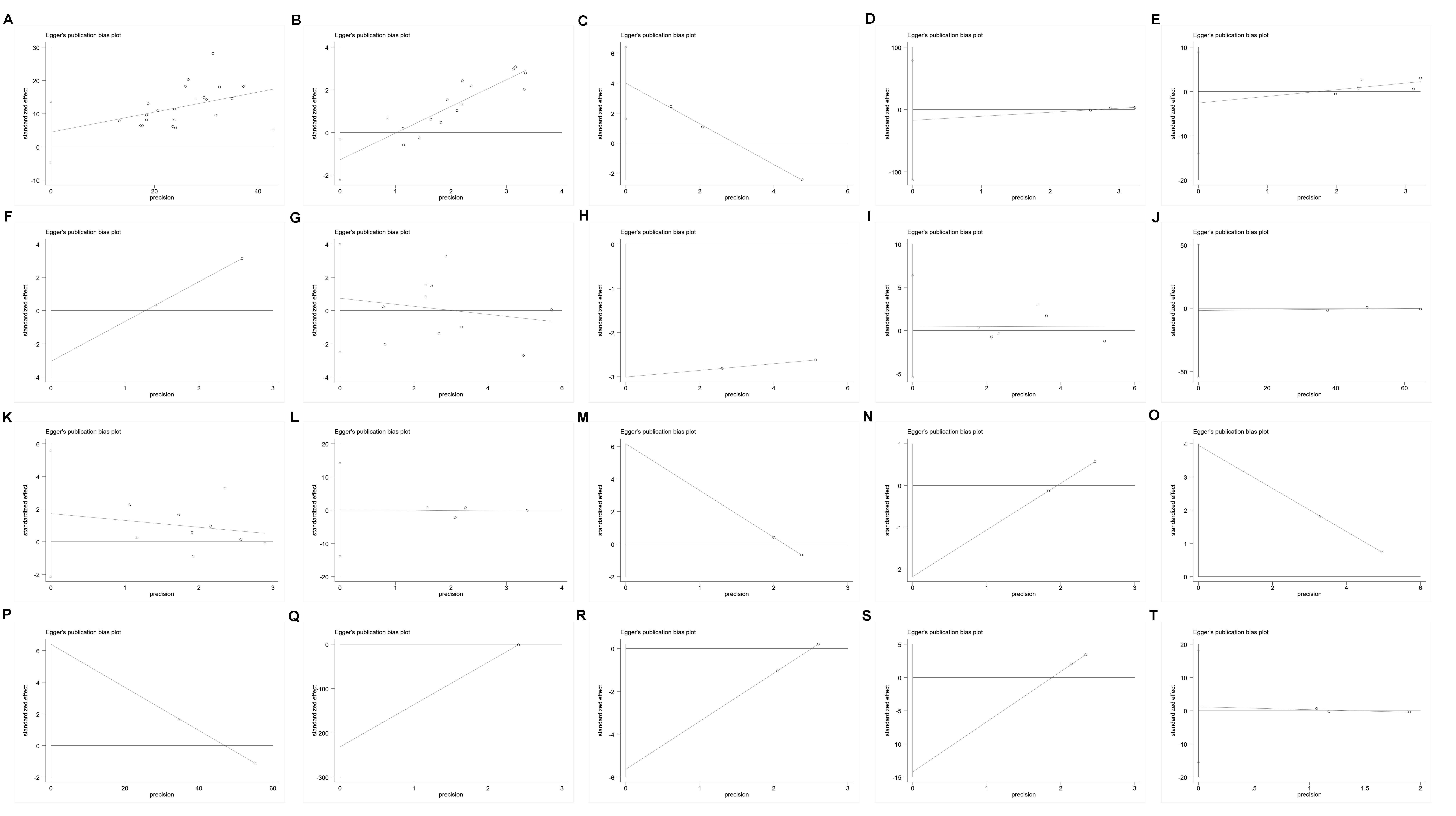


Table S3 Egger test of incidence
